# Tail-Approach-Based Design and Synthesis of Coumarin-Monoterpenes as Carbonic Anhydrase Inhibitors and Anticancer Agents

**DOI:** 10.1021/acsomega.2c07459

**Published:** 2023-02-03

**Authors:** Belma Zengin Kurt, Gulsen Celebi, Dilek Ozturk Civelek, Andrea Angeli, Atilla Akdemir, Fatih Sonmez, Claudiu T. Supuran

**Affiliations:** † Faculty of Pharmacy, Department of Pharmaceutical Chemistry, Bezmialem Vakif University, Istanbul 34093, Türkiye; ‡ Faculty of Medicine, Department of Pharmacology, Kocaeli University, Kocaeli 41001, Türkiye; § Faculty of Pharmacy, Department of Pharmacology, Bezmialem Vakif University, Istanbul 34093, Türkiye; ∥ Dipartimento Neurofarba, Sezione di Scienze Farmaceutiche e Nutraceutiche, Università degli Studi di Firenze, Via U. Schiff 6, Sesto Fiorentino, Florence 50019, Italy; ⊥ Faculty of Pharmacy, Department of Pharmacology, Computer-Aided Drug Discovery Laboratory, Bezmialem Vakif University, Istanbul 34093, Türkiye; # Pamukova Vocational School, Sakarya University of Applied Sciences, Sakarya 54055, Türkiye

## Abstract

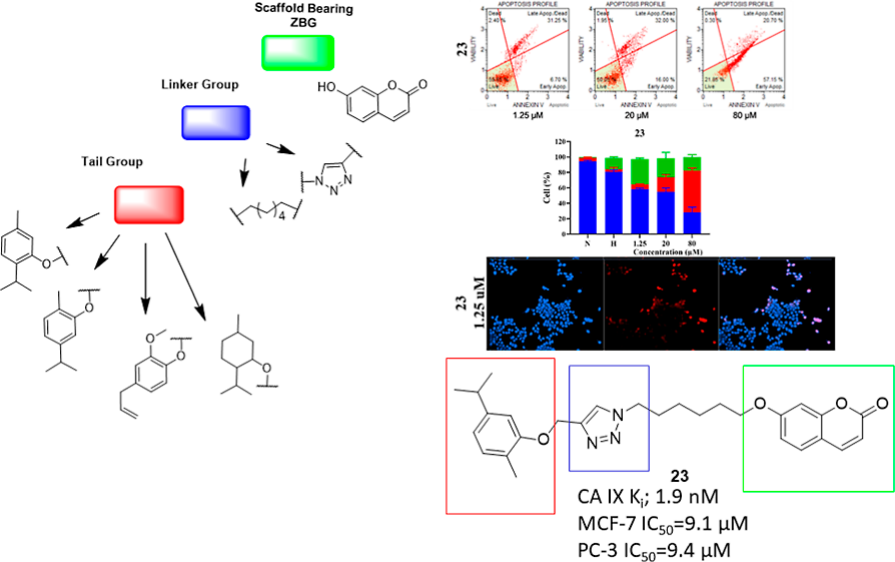

In this study, sixty novel coumarin-monoterpene compounds were synthesized in two series [thirty-two compounds (**12–43**) bearing a triazole ring in the first series, and twenty-eight compounds (**44–71**) bearing an alkyl chain in the second one]. Their inhibitory effects on the human carbonic anhydrase (hCA) isoforms I, II, IX, and XII and anticancer potentials were determined. All synthesized molecules selectively inhibited CA IX and XII. **23** and **42** were found to be the strongest inhibitors, with *K*_i_ values of 1.9 nM against hCA IX. Also, **70** showed the highest inhibitory activity with a *K*_i_ value of 4.9 nM against hCA XII. Moreover, their cytotoxic effects on colon adenocarcinoma (HT-29), prostate adenocarcinoma (PC-3), and breast adenocarcinoma (MCF-7) cell lines were evaluated. According to the cytotoxicity results, **14** (IC_50_ = 2.48 μM) and **63** (IC_50_ = 3.91 μM) exhibited the highest cytotoxicity on the MCF-7 cells, while **23** showed the strongest cytotoxic effect on both PC-3 (IC_50_ = 9.40 μM) and HT-29 (IC_50_ = 12.10 μM) cell lines. **14**, **23**, and **66** decreased CA IX and CA XII protein expression in HT-29 cells, while **23** and **66** showed the strongest reduction of both CA IX and CA XII in MCF-7 cells. All of the selected compounds increased total apoptosis in a concentration-dependent manner in HT-29 and MCF-7 cells. **14** has the strongest apoptotic effect in MCF-7 cells. **23** increased early apoptosis primarily, while **14** and **66** increased total apoptosis in HT-29. In addition, PI/Hoechst staining proves that apoptotic cells are increased in HT-29 with an effect of **14**, **23**, and **66**. As a result of the modeling studies, it has been shown that only the open coumarin form of the compounds can interact directly with the active-site Zn^2+^ ion. It has been shown that coumarin-monoterpene structures with different alkyl and monoterpene groups both specifically inhibit CA IX and XII and exhibit specific cytotoxicity in different cell lines.

## 1 Introduction

Cancer development in tissue develops through different mechanisms depending on the state of the tumor cells. Continuous division and growth of cells prevent access to oxygen through blood vessels in solid and metastatic tumors. In hypoxic conditions, cancer cells activate different metabolic pathways, such as general mitochondrial oxidative phosphorylation or anaerobic glycolysis, leading to the production of acidic metabolites. Increased acidity of the extracellular environment provides a clear selective advantage for tumor mass growth.^1^ Low pH levels can disrupt a variety of biological activities; moreover, extracellular and intracellular acidosis threaten cell viability. However, cancer cells can adapt to these changes by upregulating important pH-regulating factors. Carbonic anhydrases (CA) IX and CA XII, which are among these factors, are overexpressed under hypoxia.^2^ Necrosis around the tumor is explained by the overexpression of the CA IX isoenzyme increasing in this region and the dependence of pH control on this enzyme.^3^

The known basic function of CAs is hydration of the physiological reaction CO_2_ to bicarbonate and proton. CAs reversibly catalyze this reaction in all living organisms, including bacteria, archaea, and eukaryotes. CAs play a role in many pathological and physiological processes by providing pH and CO_2_ homeostasis as well as coping with the excess CO_2_ generated as a result of metabolic activities in all these organisms. Sixteen CA isoenzymes or CA-related proteins (CARPs) have been identified in mammals, and they differ in their catalytic activity, subcellular localization, and tissue distribution.^4^ CA I, CA II, CA III, CA VII, and CA XIII are cytosolic; CA IV, CA IX, CA XII, CA XIV, and CA XV are membrane-bound; CA VA and CA VB are mitochondrial; and CA VI shows intracellular localization as CA isoenzymes secreted in saliva, milk, and urine. Tissue distributions differ according to physio/pathological conditions, while there is an overproduction of CA IX and XII isoenzymes in hypoxic tumors, CA VA and XIV are predominant in the liver.^5^ Increased expression of CA IX is seen in most solid cancers including colorectal, breast, and pancreatic tumors.^6^ This overproduction of tumor-specific CA IX and XII isoenzymes has also been shown in cell culture studies. HT-29, MCF-7, and PC3 cell lines overexpressed, especially CA IX in hypoxic conditions.^7^ Studies have also shown that some natural/synthetic coumarin derivatives have high cytotoxicity on these cells.^8^

With the inhibition of the CA IX isoenzyme, the tumor growth activity of CA IX in hypoxic tumors is prevented, and the pH irregularity in tumors is controlled and allows for new applications in cancer diagnosis and treatment. CA IX, like other α-CAs, is inhibited by anionic inhibitors or sulfonamides and sulfamates that act by directly interacting with the Zn^+2^ ion in the active site space or by various interactions with hydrophilic/lipophilic amino acids in the active site.^9,10^ Among these inhibitors, it has been shown in vivo experiments that especially nonmembrane impermeable derivatives have selective inhibition of CA IX. In addition to derivatives that are membrane-impermeable, derivatives carrying a sugar structure also inhibit CA IX at low levels.^9,11^

**Figure 1:**
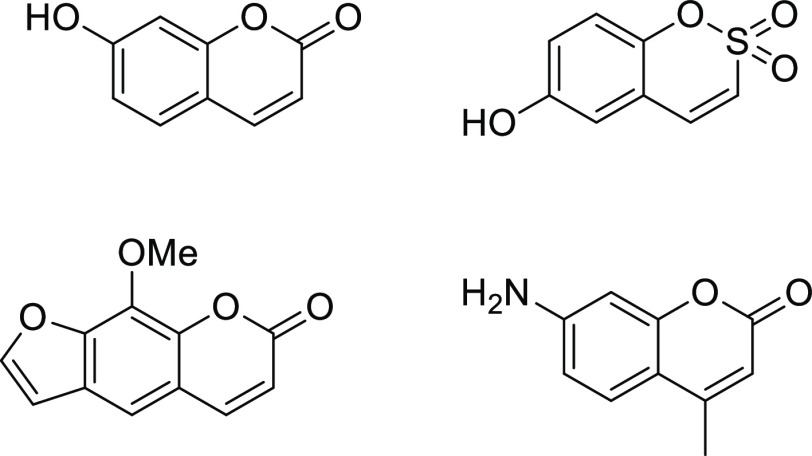
Some coumarin derivatives known as CAIs.

In addition to these CA inhibitors (CAIs), coumarin derivatives are important molecules that show selective inhibition of CA IX and CA XII at low nanomolar concentrations (Figure 1). Due to the nucleophilicity of zinc hydroxide, coumarins can easily be hydrolyzed to the substituted 2-hydroxycinnamic acid during the preincubation process, which is the most important step in providing this activity.^12^ Many studies have been conducted where coumarins have shown selective CA inhibition. Substituted coumarins and thiocoumarin derivatives also inhibited the CAs in the low nanomolar-micromolar range.^13^

**Figure 2:**
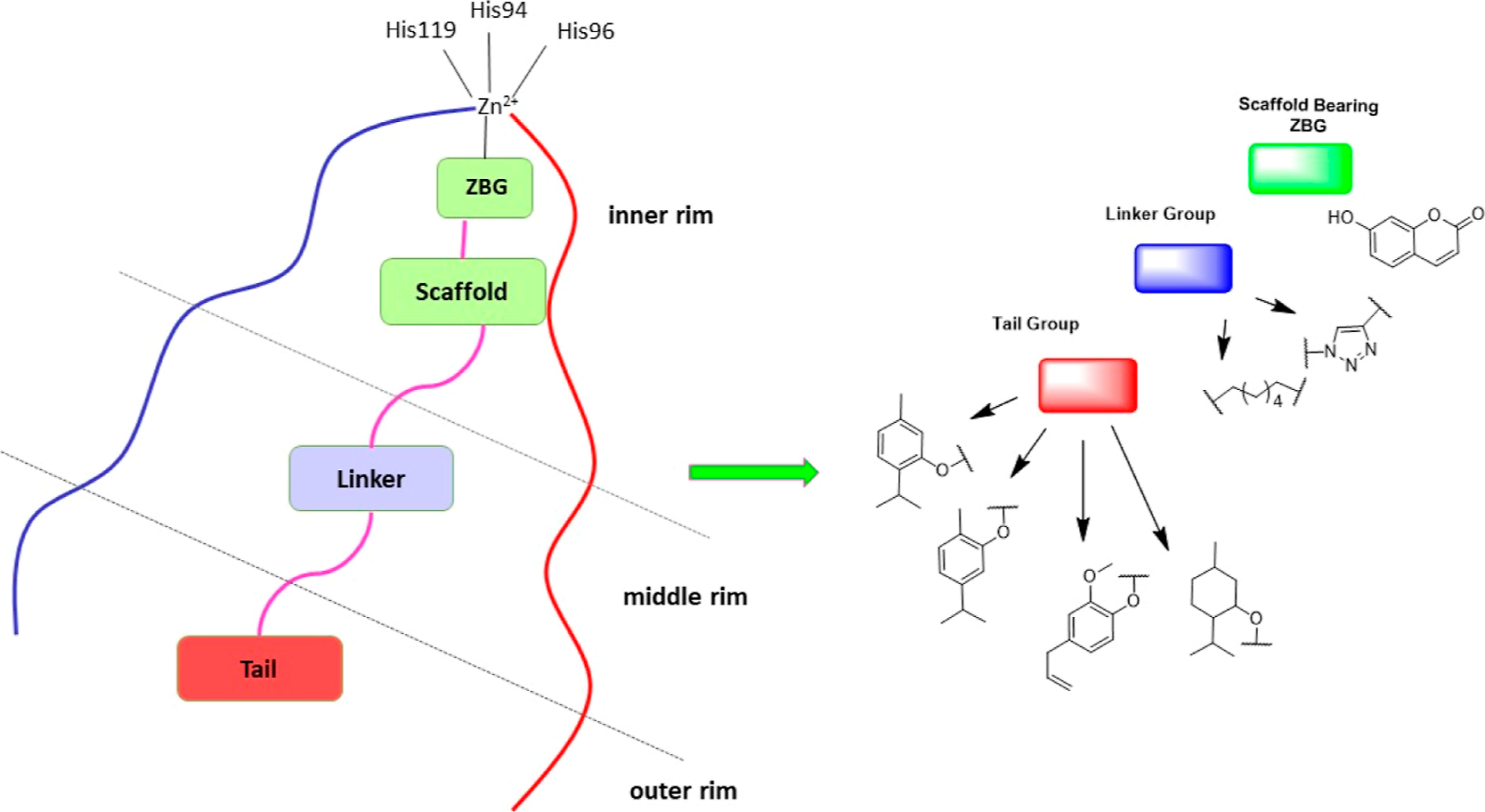
Tail-approach-based design of coumarin monoterpene derivatives.

The tail approach has been found to be the most successful design recently applied to obtain selective CAIs.^14^ Some researchers have also shown that this approach can increase the selective inhibition of CA IX and XII.^15,16^ The “tail approach” is a structure-based drug design approach and consists of adding “tail(s)” to an aromatic or heterocyclic scaffold equipped with a zinc linking group (ZBG), such as sulfonamides or their bioisosteres (Figure 2). Thus, it leads to an expanded molecule, and this structure can interact selectively between various isoforms (based on amino acid residues) with the middle or outer edge of the active site cavity. This affects the selectivity as well as the strength of the inhibitor.^15,17^

Based on these findings, we designed coumarins with different alkyl chain lengths as hybrid molecules with monoterpene compounds, such as thymol, carvacrol, and so forth, which are potential CAIs.^18^ After the CA inhibitions of the obtained compounds were determined, we examined the potential of the compounds to be developed as antitumor agents by selecting the cancer cell lines that were the most expressed CA IX and XII isoforms. The effects of compounds were first screened on the HT-29 cell line, and then, their effects on PC-3 and MCF-7 cell lines were examined by choosing a narrower compound scale according to enzyme inhibition results and HT-29 cytotoxicity results. Finally, apoptosis profiles and intracellular CA IX and XII protein levels of the most promising compounds were determined in HT-29 and MCF-7 cells. Furthermore, the binding interactions of these compounds with either hCA IX or XII were investigated with docking studies followed by molecular dynamics simulations.

## 2 Results and Discussion

### 2.1 Chemistry

**Scheme 1:**
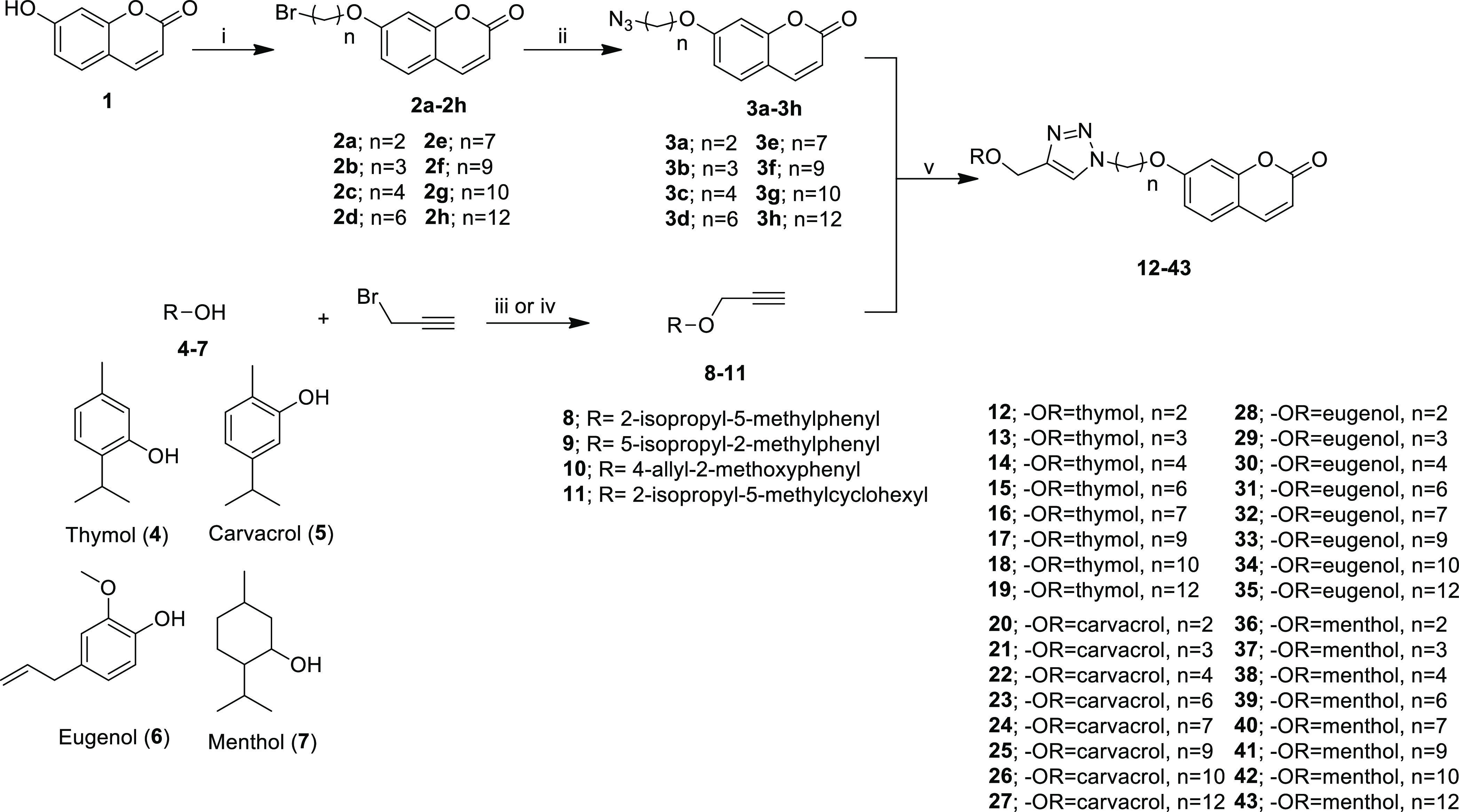
Synthesis of Bearing Triazole Ring Coumarin-Monoterpene Derivatives Reaction conditions: (i) dibromoalkane, K_2_CO_3_, and CH_3_CN, reflux, 2 h; (ii) NaN_3_ and DMF, rt, 16 h; (iii) propargyl bromide, DMF, and K_2_CO_3_, rt, 16 h; (iv) NaH, THF, and TBAI, propargyl bromide, 70 °C, 4 h; (v) PMDETA, CuBr, and DMF, rt.

**Scheme 2:**
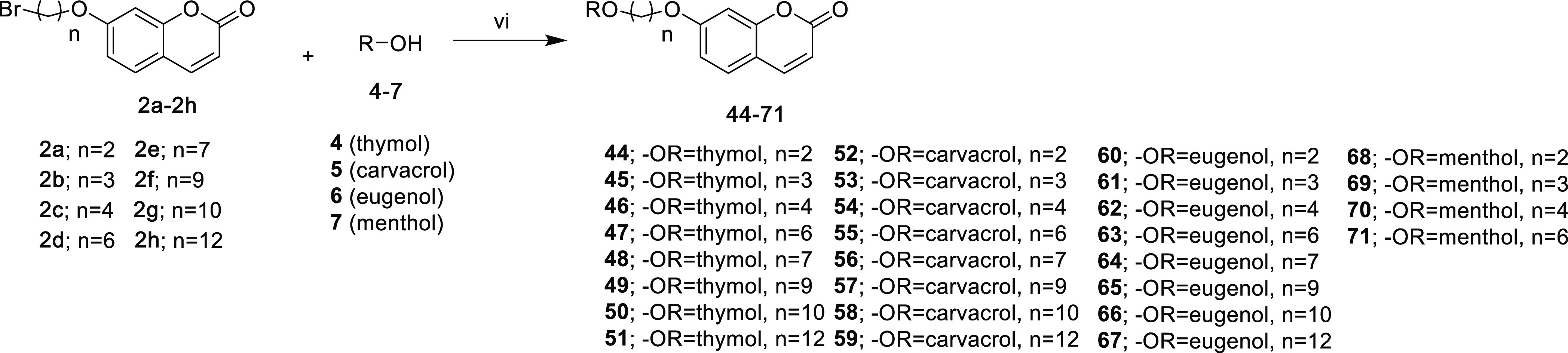
Synthesis of Coumarin-Monoterpene Derivatives Reaction conditions: (vi) KI, K_2_CO_3_, and DMF, 60 °C, 18 h for compounds **44–67**; NaI and DIPEA, 150 °C, 2 h for compounds **68–71**.

New coumarin derivatives containing monoterpene moieties were designed as two series. The first one has a triazole ring, and the second has an alkyl chain as linkers between coumarin and monoterpenes. The synthesis of these series is given in Schemes 1 and 2, respectively.

7-hydroxy coumarin (**1**) was reacted with dibromoalkane and then NaN_3_ to obtain coumarin derivatives bearing an azide moiety (**3a–3h**). On the other hand, monoterpenes [thymol (**4**), carvacrol (**5**), eugenol (**6**), and menthol (**7**)] were propargylated with propargyl bromide in DMF for binding the alkyne moiety to monoterpenes (**8–11**). The target compounds (**12–43**), bearing a triazole ring, in the first series were obtained by reacting **3a–3h** with **8–11** via the azide–alkyne Huisgen cycloaddition method (Scheme 1).

The synthesis of target compounds (**44–71**) bearing alkyl chains in the second series (Scheme 2) was achieved via the substitution reaction of monoterpenes (**4–7**) with coumarin derivatives containing an alkyl bromide moiety (**2a–2h**).

From the ^1^H NMR spectra of compounds **8–11**, the signals for propargyl protons were observed at 2.48–2.54, and 4.24–4.75 ppm. According to the IR spectra, the stretch signal of the alkyne group is seen around 2120 cm^–1^.

From the ^1^H NMR spectra of compounds **12–43**, the coumarin and monoterpene ring protons as well as a proton signal belonging to the triazole ring formed were observed in the aromatic region. In general, aromatic protons signaled in approximately the same areas for thymol, carvacrol, and eugenol. The signals for aromatic protons were observed between 6.24 and 8.28 ppm, while aliphatic proton signals were observed about at 1.10–5.21 ppm. Among these signals, the (CH_3_)_2_CH– doublet seen around 1.10–1.20 ppm, the Ar–CH_3_ singlet seen around 2.20–2.30 ppm, and the (CH_3_)_2_CH– multiplet seen in the range of 3.00–3.30 ppm are signals of specific aliphatic protons belonging to thymol/carvacrol derivatives (**12–27**). For compounds **28–35** containing the eugenol ring, specific signals belonging to the allyl group of eugenol and the methoxy group were seen instead of these peaks. The signals of methoxy protons were determined at 3.90 ppm, and the signals of terminal alkene protons were observed between 5.00 and 5.10 ppm. The signal of the −CH proton of the allyl group was observed in the range of 5.80–6.00 ppm, while −CH_2_ proton signal was seen around 3.36 ppm. For compounds **36–43** bearing a menthol ring, specific signals of the menthol ring were seen in the range of 0.63–3.20 ppm. From the ^13^C NMR spectra, the signals of the aliphatic and aromatic carbons were observed at 16–80 and 101.5–162.7 ppm, respectively.

From the ^1^H NMR spectra of compounds **44–71**, in the second series; the signals for aromatic protons were seen between 6.13 and 7.96 ppm, while the signals of aliphatic protons were observed between 0.69 and 4.25 ppm. From the ^13^C NMR spectra of them, the signals of aromatic carbon were observed in the range of 100.3–162.6 ppm, while the signals of aliphatic carbon were detected between 21.5 and 69.5 ppm.

### 2.2 CA Inhibition

The *K*_i_ values of the synthesized compounds **12–43** and **44–71** against hCA IX and hCA XII isoforms are given in Tables 1 and 2, respectively. Generally, all synthesized compounds (**12–71**) selectively inhibited the hCA IX and hCA XII (the tumor-associated isoforms) with *K*_i_ values in the range of 1.9–3507.0 nM, while they inhibited the hCA I and II isoforms in the micromolar level (*K*_i_ > 10,000 nM, therefore not given in the Tables 1 and 2).

(i) The *K*_i_ values of compounds **12–43**, bearing a triazole moiety in the first series, were determined in the range of 1.9–1871.0 and 8.2–664.5 nM against hCA IX and hCA XII, respectively (Table 1). In the first series, seven compounds [**12** (*K*_i_ = 2.9 nM), **23** (*K*_i_ = 1.9 nM), **29** (*K*_i_ = 2.4 nM), **30** (*K*_i_ = 3.1 nM), **37** (*K*_i_ = 2.2 nM), **42** (*K*_i_ = 1.9 nM), and **43** (*K*_i_ = 2.5 nM)] inhibited the tumor-associated isoform hCA IX approx. 10-fold stronger than acetazolamide (AAZ, *K*_i_ = 25.8 nM), used as a standard CAI. Moreover, eleven compounds (**15** (*K*_i_ = 25.2 nM), **19** (*K*_i_ = 21.8 nM), **20** (*K*_i_ = 12.6 nM), **22** (*K*_i_ = 28.7 nM), **24** (*K*_i_ = 32.7 nM), **27** (*K*_i_ = 30.4 nM), **28** (*K*_i_ = 31.9 nM), **31** (*K*_i_ = 31.5 nM), **34** (*K*_i_ = 28.1 nM), **35** (*K*_i_ = 29.9 nM), and **39** (*K*_i_ = 20.7 nM)) exhibited hCA IX inhibitory activity similar to or higher than AAZ. In this series, compounds **14**, **15**, and **40** strongly inhibited the other tumor-associated isoform hCA XII with *K*_i_ values of 9.3, 9.5, and 8.2 nM, respectively, which are close to that of AAZ (*K*_i_ of 5.7 nM). The cytosolic isoforms hCA I and hCA II (with *K*_i_ >10,000 nM) were weakly inhibited by compounds **12–43**.
(ii) From Table 2, the *K*_i_ values of compounds **44–71**, bearing alkyl chain in the second series, were defined in the range of 22.1–3507.0 and 4.9–83.6 nM against hCA IX and hCA XII, respectively. In the second series, only compound **63** (*K*_i_ = 22.1 nM) showed hCA IX inhibitory activity better than AAZ (*K*_i_ = 25.8 nM), whereas nine compounds (**48**, **51**, **52**, **55**, **59**, **61**, **62**, **69**, and **71**) inhibited hCA IX with *K*_i_ values of 31.3–38.3 nM, which are close to that of AAZ. On the other hand, the tumor-associated isoform hCA XII was strongly inhibited by twenty two of the twenty eight compounds with *K*_i_ values in the range from 4.9 to 9.4 nM. Among them, compound **70** (*K*_i_ = 4.9 nM) has the highest inhibitory activity against hCA XII. In this series, all synthesized compounds strongly inhibited hCA XII more than hCA IX.
(iii) Comparing both series (from Tables 1 and 2), most of the synthesized compounds bearing a triazole ring in the first series exhibited more potent inhibitory activity against hCA IX, while the compounds bearing an alkyl chain in the second series showed stronger inhibitory activity against hCA XII. Compounds **40** (*K*_i_ = 8.2 nM) and **70** (*K*_i_ = 4.9 nM), which are the most potent hCA XII inhibitors in both series, contain menthol as the monoterpene group, while the most effective hCA IX inhibitors include different monoterpene groups [−OR: carvacrol for compound **23** (*K*_i_ = 1.9 nM); −OR: menthol for compounds **37** (*K*_i_ = 2.2 nM) and **42** (*K*_i_ = 1.9 nM); −OR: eugenol for compounds **29** (*K*_i_ = 2.4 nM) and **63** (*K*_i_ = 22.1 nM)].

**Table 1:**
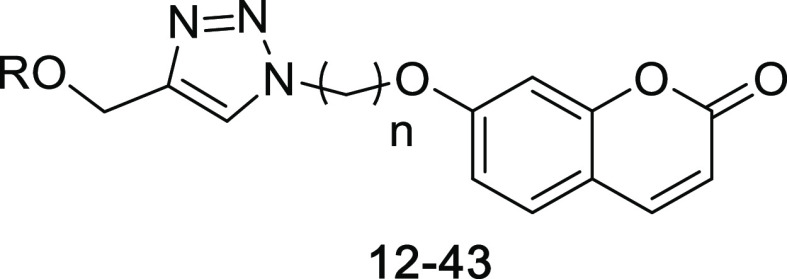

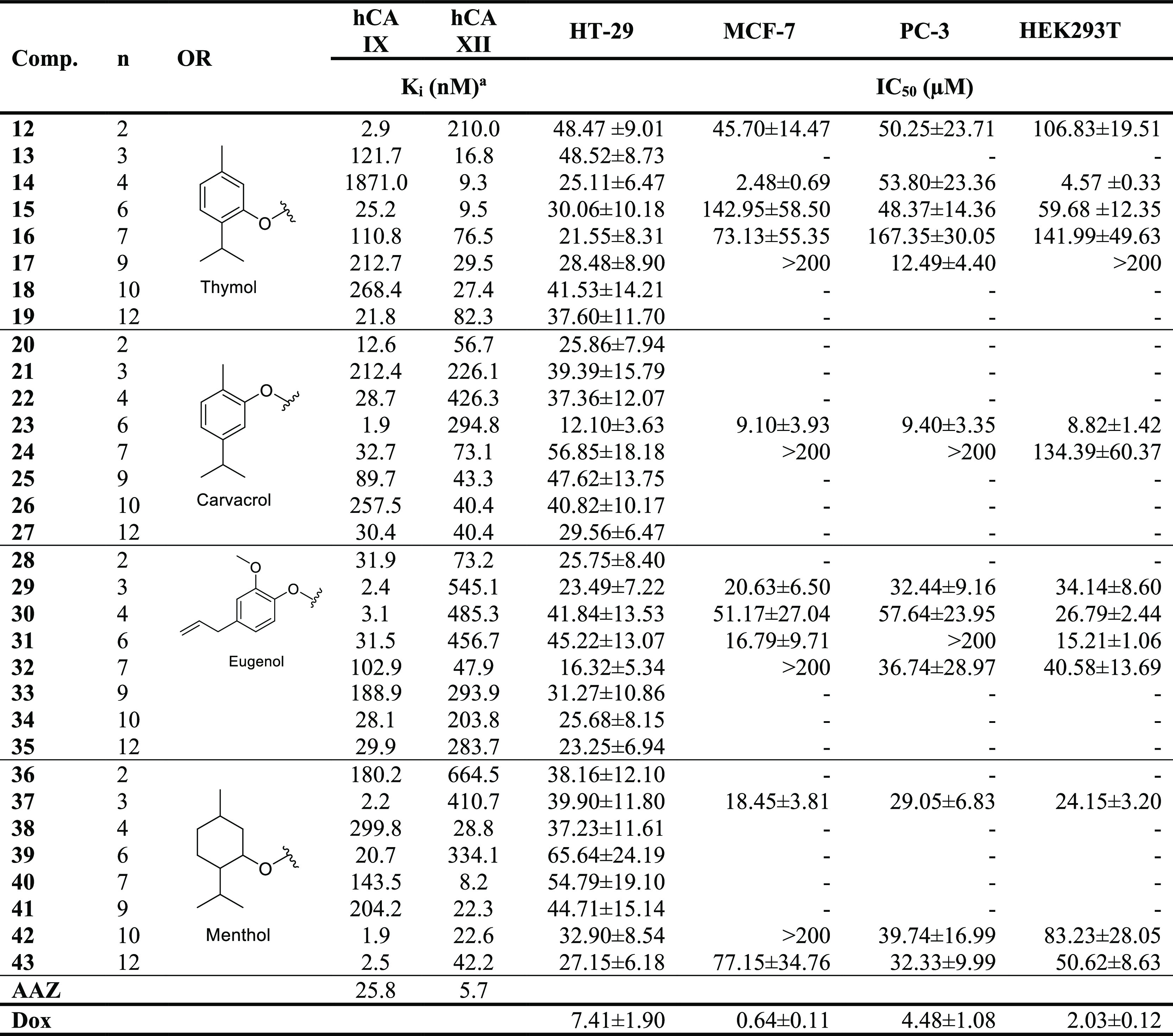
*K*_i_ (nM) for hCA IX and XII, IC_50_ (μM) Values for HT-29 Cell Line, and IC_50_ Values Calculated from Viability at MCF-7, PC-3, and HEK293T Cell Line Results of Selected Molecules (IC_50,_ μM) of **12–43**

a Mean from 3 different assays, by a stopped-flow technique (errors were in the range of ±5–10% of the reported values).

**Table 2:**
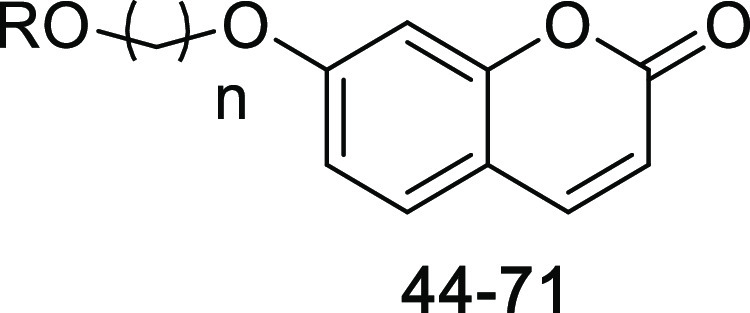

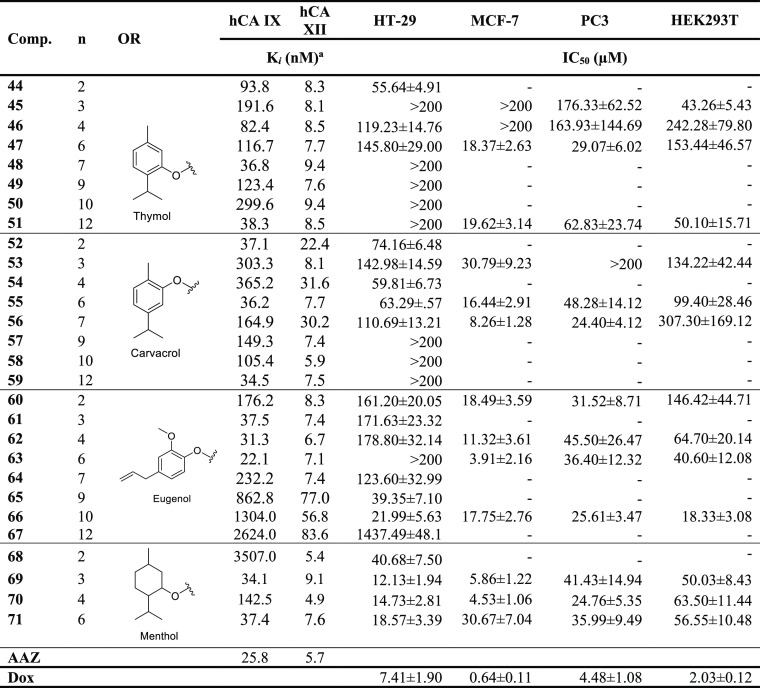
*K*_i_ (nM) for hCA I, II, IX, and XII, IC_50_ (μM) Values for HT-29 Cell Line, and IC_50_ Values Calculated from Viability at MCF-7, PC-3, and HEK293T Cell Line Results of Selected Molecules (IC_50,_ μM) of **44–71**

a Mean from 3 different assays, by a stopped-flow technique (errors were in the range of ±5–10% of the reported values).

Regardless of monoterpene groups, compounds containing only straight alkyl chains exhibited higher inhibition of CA XII than compounds containing triazole groups. This difference is due to the triazole group reducing the lipophilic character.^15,16,19^ However, the same effect was not seen for CA IX inhibition. Different monoterpene structures forming the tail part also affected CA inhibition to different degrees. Binding sites and aromatic and aliphatic structure interactions have brought about this difference.

### 2.3 Cell Viability against Normal and Cancer Cell Lines

IC_50_ values showing cytotoxic effects of newly synthesized compounds tested against HT-29, MCF-7, PC3, and HEK293T cell lines by MTT assay were compiled in Tables 1 and 2. The compounds **12–43** exhibited similar cytotoxicity against HT-29 cells that IC_50_ values ranged from 12.10–65.6 μM. The most promising compounds in the series were **14** (IC_50_ = 25.11 μM), **16** (IC_50_ = 21.55 μM), **23** (IC_50_ = 12.10 μM), **29** (IC_50_ = 23.49 μM), and **35** (IC_50_ = 23.25 μM) (Table 1). The compounds **44–71** generally showed weaker cytotoxic activity against HT-29 cells except **66** (IC_50_ = 21.99 μM), **69** (IC_50_ = 12.13 μM), **70** (IC_50_ = 14.73 μM), and **71** (IC_50_ = 18.57 μM) (Table 2). All compounds tested against HT-29 cells showed weaker cytotoxic effects than doxorubicin (IC_50_ = 7.41 μM). Considering the results of CA IX and XII enzyme inhibitions and cytotoxicity on HT-29 cells, no significant linear relationship was observed. It is possible that the cytotoxicity of the substances occurs via different pathways other than CA IX and XII inhibition.

For this reason, a narrower group of compounds was selected from the compounds exhibiting both high CA IX and XII inhibition and high HT-29 cytotoxicity, and cytotoxic evaluation was performed in MCF-7, PC3, and HEK293T cell lines. **14**, **23**, **56**, **63**, **69,** and **70** had lower IC_50_ values for cell cytotoxicity than others against MCF-7 cells (**14;** IC_50_ = 2.48 μM, **23**; IC_50_ = 9.10 μM, **56**; IC_50_ = 8.26 μM, **63**; IC_50_ = 3.91 μM, **69**; IC_50_ = 5.86 μM, **70**; and IC_50_ = 7.53 μM). Against PC3 cell lines, **17** (IC_50_ = 12.49 μM) and **23** (IC_50_ = 9.40 μM) showed the strongest cytotoxic activity. Although these values are lower when compared to the effect of doxorubicin (dox) on cancer cells, the synthesized compounds are less cytotoxic than dox in healthy cell lines. If we compare the selectivities of dox and active compounds on cancer and healthy cells, for example, the selectivity of **23** on HT-29 is 3-fold greater than dox. Similarly, the selectivity of **63** in MCF-7 is again 15-fold higher than dox.

### 2.4 Effects of Some Selected Compounds on Protein Levels of CA IX and CA XII

**Figure 3:**
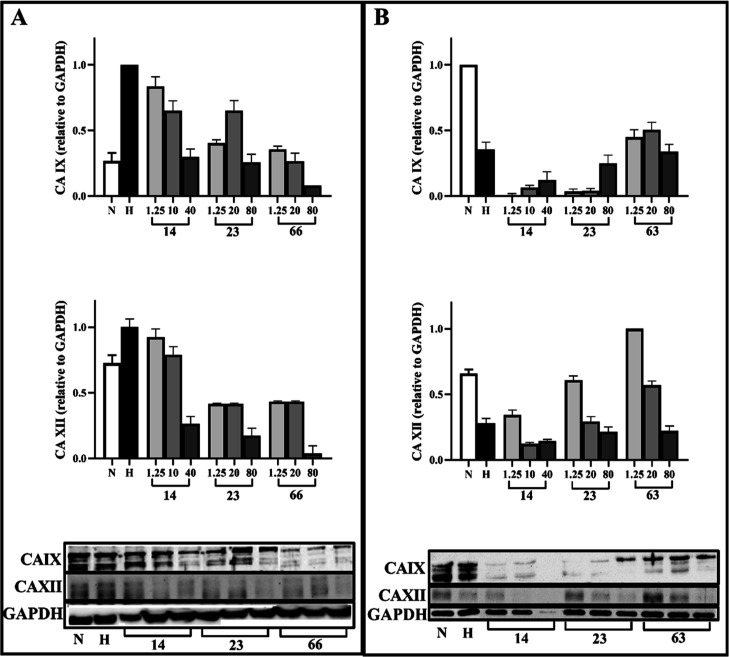
CA IX and CA II protein expression levels after the administration of compounds **14**, **23**, and **66** on HT-29 (A) and compounds **14**, **23**, and **63** on MCF-7 (B).

We performed a western blot experiment to understand the effect of selected compounds **14**, **23**, **63,** and **66** according to the results of experiments on protein expression of tumorigenic carbonic anhydrases CA IX and CA XII on two different cancer cell lines HT-29 and MCF-7. The effects of compounds **14**, **23**, and **66** on HT-29 cells and the effects of compounds **14**, **23**, and **63** on MCF-7 cells were evaluated. The results of western blot experiments showed that compounds **14, 23**, and **66** decreased the CA IX and CA XII expression in HT-29 cells. The highest decreases in CA IX and CA XII expression were determined at the highest dose of **66** (Figure 3A). On the other hand, **63** decreased the normal expression level of CA IX and XII in MCF-7 cells, while **14** and **23** decreased both the level of CAIX and CA XII (Figure 3B).

### 2.5 Apoptotic Effects of Some Selected Compounds

**Figure 4:**
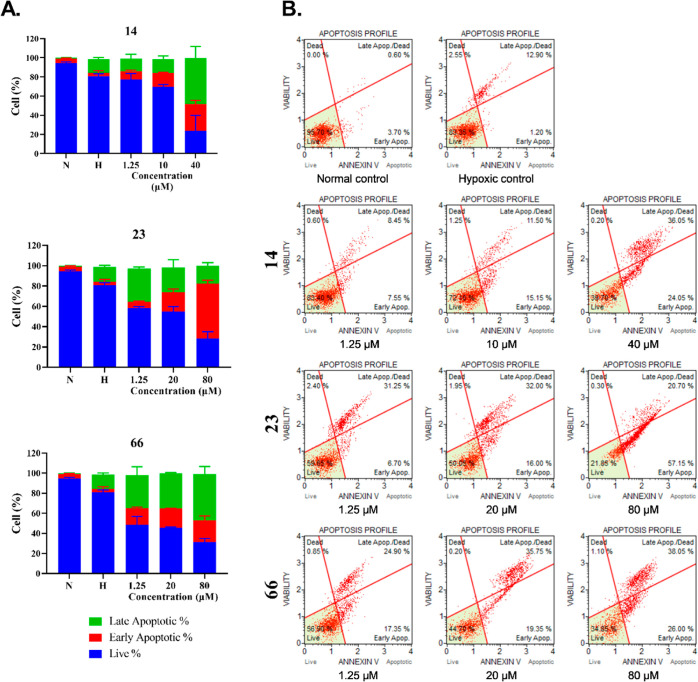
Evaluation of apoptotic cell profiles of normal, hypoxic controls, and treated cells. (A) Live, early, and late apoptotic cell percentages of **14**, **23**, and **66** treated HT-29 cells compared to normal and hypoxic controls. (B) Apoptotic profiles of normal, hypoxic controls, and treated HT-29 cells at three different concentrations.

Apoptotic profiles of selected compounds **14**, **23**, **63,** and **66** were evaluated in HT-29 (Figure 4) and MCF-7 cells (Figure 4). The results showed that **14**, **23**, and **66** generally increased late apoptotic cells (%) in HT-29, whereas **14**, **23**, and **63** mostly effected early apoptosis in MCF-7. A concentration-dependent increase in late apoptotic cells (%) in HT-29 depending on **14** and **66** was determined. On the other hand, treatment of **23** in HT-29 cells resulted in early apoptosis in a concentration-dependent manner (Figure 4).

**Figure 5:**
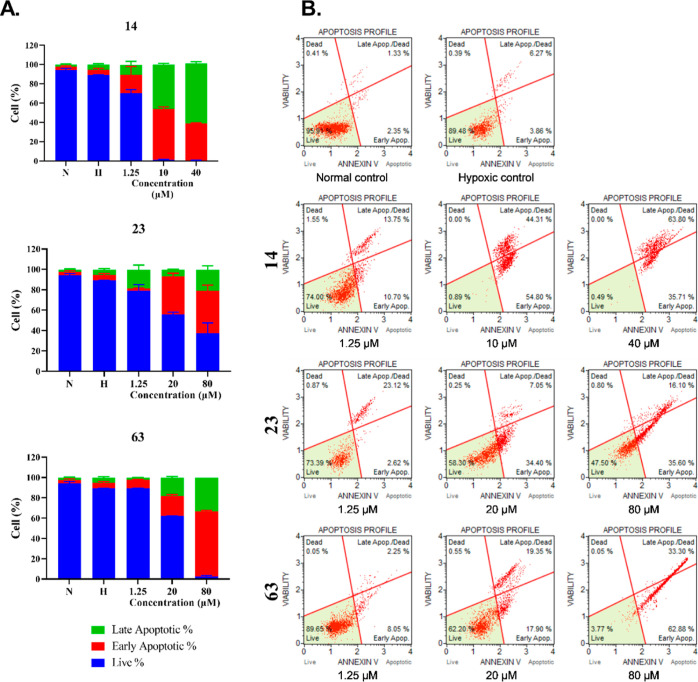
Evaluation of apoptotic cell profiles of normal, hypoxic controls, and treated cells. (A) Live, early, and late apoptotic cell percentages of **14**, **23**, and **63** treated MCF-7 cells compared to normal and hypoxic controls. (B) Apoptotic profiles of normal, hypoxic controls, and treated MCF-7 cells at three different concentrations.

Compound **14** exhibited a strong apoptotic effect on MCF-7 cells with a significant increase in both early and late apoptotic cells (%) at 10 and 40 μM. Both **23** and **63** increased the early and late apoptotic cells (%) in rising concentrations (Figure 5).

**Figure 6:**
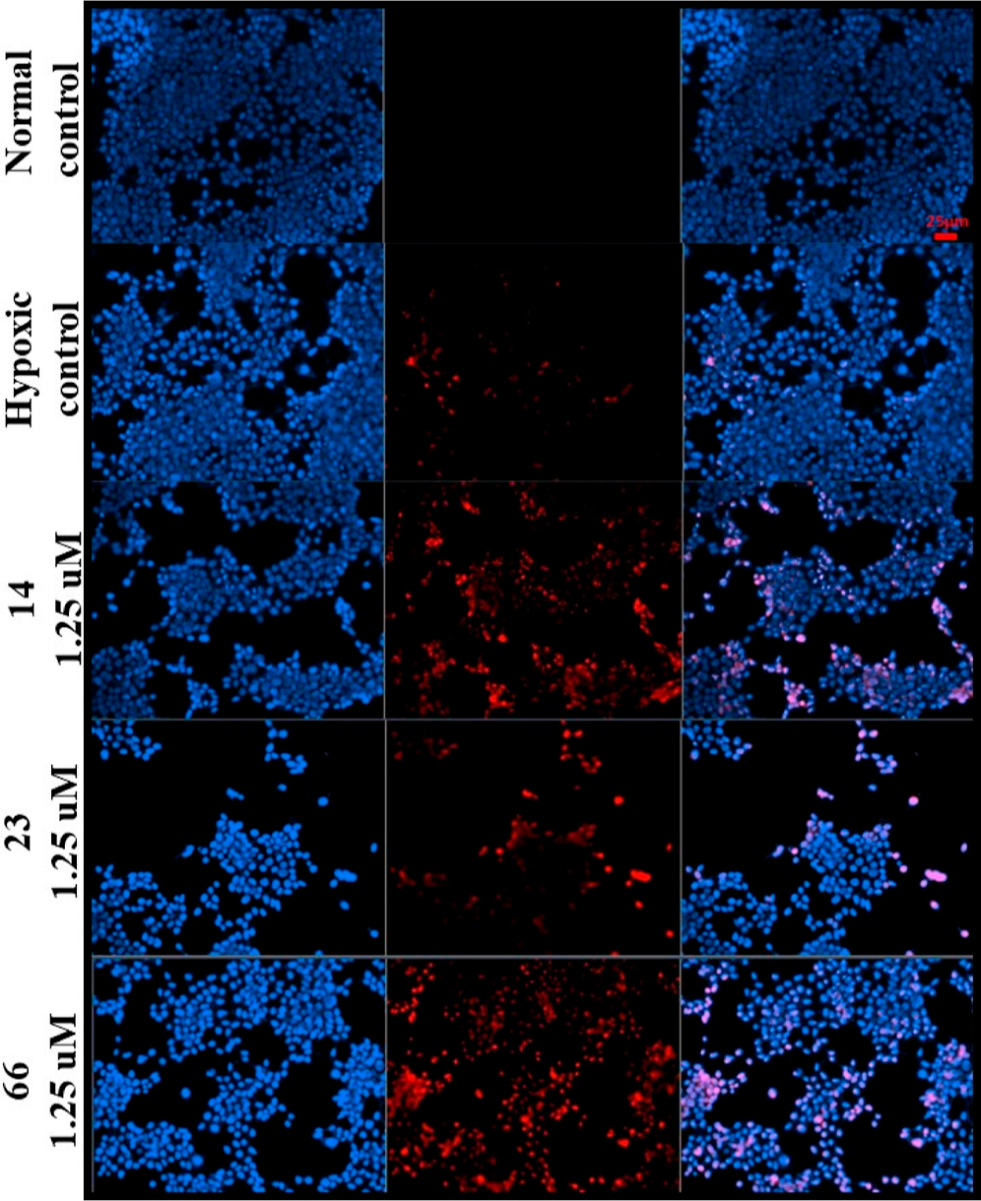
Fluorescence imaging of controls and treated HT-29 cells. ×20 magnification. The first column shows Hoechst, the second column shows PI, and the last column shows the merged images of the two dyes.

To further validate the cell viability and apoptotic effects of the compounds, HT-29 cells were examined under fluorescence microscopy. After incubation with **14**, **23**, and **66**, cells were stained with PI and Hoechst. As seen in Figure 6, PI did not penetrate normal control cells. However, it was determined that the cells stained with PI increased after hypoxic conditions. It can be said that compounds **14**, **23**, and **66** increased apoptotic/dead cells, as seen in Figure 6.

### 2.6 Molecular Modelling Studies

Compounds **23** and **70** showed the lowest *K*_i_ values for hCA IX (1.9 nM) and hCA XII (4.9 nM), respectively. The binding interactions of these compounds with either hCA IX or XII were investigated with docking studies followed by 50 ns molecular dynamics simulations.

#### 2.6.1 Investigation of Binding Interactions of Compound **23** with hCA IX

**Figure 7:**
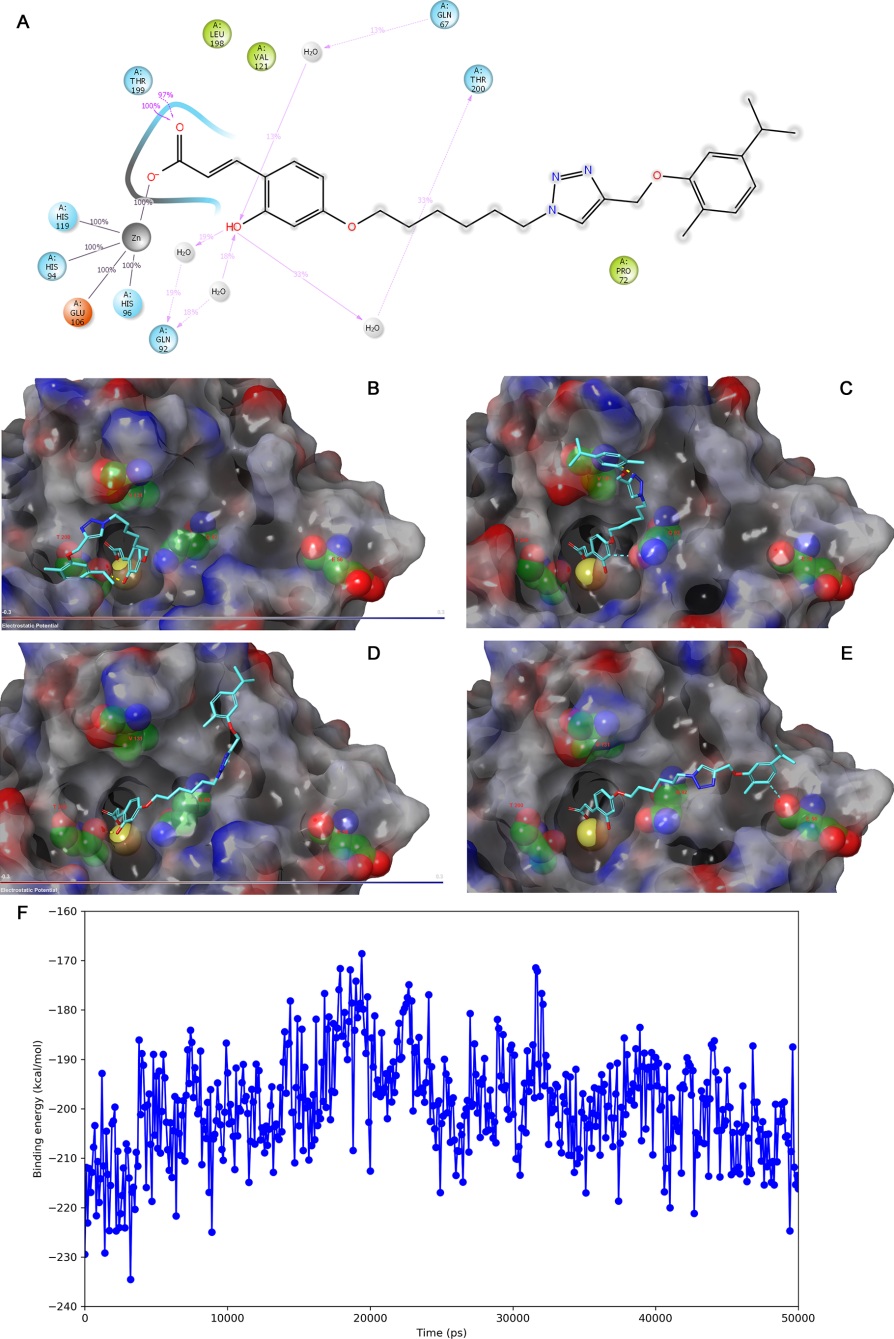
Interactions of the hCA IX—compound **23** complex during a 50 ns MD simulation. (A) Overview of the observed binding interactions. Snapshots of the ligand binding pose at 0 (B), 17.2 (C), 26.8 (D), and 34 ns (E). The MM–GBSA binding energy of the ligand-enzyme complex (F). The Zn^2+^ ion is shown in a large yellow sphere. Hydrogen bonds are indicated in yellow dashed lines. Aromatic hydrogen bonds are indicated in turquoise dashed lines.

Compound **23** can form an interaction with the active site Zn^2+^ ion, only when it is in the open-coumarin form (Figure 7). The docked pose shows a hydrogen bond of the ligand’s hydroxyl group with the sidechain of Thr200, while the carboxylic acid group interacts with the Zn^2+^ ion. The ligand is in a folded conformation and the terminal phenyl group forms hydrophobic interactions with the sidechains of Trp5 and Pro202.

During the entire simulation, the ligand’s carboxylic acid interacts with the Zn^2+^ ion and Thr199 (Figure 7A). The interaction of the hydroxyl group with Thr200 is not stable instead, it forms hydrogen bonds with bridging water molecules with Gln67, Gln92, and Thr200. Due to the flexible alkyl chain, the ligand adopts several conformations in the active site with a near-extended alkyl chain conformation. As a result, the triazole and phenyl groups of the ligand form occasionally hydrogen bonds or aromatic hydrogen bonds with the active site. The calculated MM–GBSA ligand-enzyme binding energy fluctuates roughly between −220 and 190 kcal/mol (Figure 7F).

No docked pose was observed of the compound was observed for the closed coumarin form in which the ligand forms an interaction with the zinc ion.

#### 2.6.2 Investigation of Binding Interactions of Compound **70** with hCA XII

**Figure 8:**
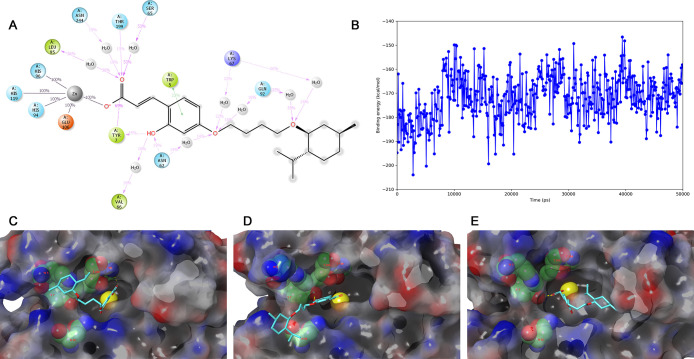
Interactions of the hCA XII—compound **70** complex during a 50 ns MD simulation. (A) Overview of the observed binding interactions. (B) MM–GBSA binding energy of the ligand-enzyme complex snapshots of the ligand binding pose at 0 (C), 10 (D), and 50 ns (E). The Zn^2+^ ion is shown in a large yellow sphere. Hydrogen bonds are indicated in yellow dashed lines. Aromatic hydrogen bonds are indicated in turquoise dashed lines.

Modeling studies indicated again that only the open-coumarin form of compound **70** was able to form a direct interaction with the active site Zn^2+^ ion, while the closed-coumarin form could not. The docked pose shows the interaction between the ligand’s carboxylic acid group and the Zn^2+^ ion as well as Thr199 (Figure 8). The ligand’s substituted cyclohexyl group is located close to Trp5 and His64 and forms hydrophobic interactions with these two amino acids.

During the 50 ns MD simulation, the interaction with Zn^2+^ is preserved, while the interaction with Thr199 is only present during 15% of the simulation time. However, the carboxylic acid forms interactions with Ser65, Leu95, and Asn244 via a bridging water molecule (Figure 8A). The ligand’s hydroxyl group can form direct interactions with Tyr7 and Asn62 and an interaction mediated by a bridging water molecule with Val66. The rest of the ligand is very flexible during the simulation and adopts different conformations in the active site. This part of the ligand mainly forms interactions via bridging water molecules. The calculated MM–GBSA binding energy between the ligand and active site ranges approximately from −190 to −160 kcal/mol.

## 3 Conclusions

In conclusion, 60 novel coumarin monoterpenes (**12–71**) were synthesized in two series (bearing a triazole ring and an alkyl chain) as CAIs. All the synthesized compounds in both series showed selective inhibitory activity at the nanomolar level against hCA IX and hCA XII (the tumor-associated isoforms). Compounds **23** and **42** exhibited the strongest inhibitory activity against hCA IX with *K*_i_ values of 1.9 nM, while compound **70** showed the highest hCA XII inhibition with a *K*_i_ of 4.9 nM. Moreover, seven compounds (**12**, **23**, **29**, **30**, **37**, **42**, and **43**) inhibited the tumor-associated isoform hCA IX with *K*_i_ values in the range 1.9–3.1 nM, which are approx. 10-fold lower than those of acetazolamide (AAZ, *K*_i_ = 25.8 nM). In addition, twenty-one compounds (**15**, **19**, **20**, **22**, **24**, **27**, **28**, **31**, **34**, **35**, **39**, **48**, **51**, **52**, **55**, **59**, **61**, **62**, **63**, **69**, and **71**; *K*_i_ = 12.6–38.3 nM) showed hCA IX inhibitory activity similar to or higher than AAZ. On the other hand, the tumor-associated isoform hCA XII was strongly inhibited by most synthesized compounds, with *K*_i_ values in the range from 4.9 to 9.5 nM. Among them, compound **70** (*K*_i_ = 4.9 nM) was found to be the most potent inhibitor against hCA XII. Docking studies in combination with molecular dynamics simulations have suggested the possible binding interactions of the target enzymes with compounds **23** and **70**.

The cytotoxic properties of all compounds were determined on HT-29, one of the cells in which CA IX and XII were overexpressed. After this initial cytotoxic screening, a narrower compound scale was selected according to the enzyme inhibition results and the HT-29 cytotoxicity results, and their cytotoxic effects were determined on PC-3, MCF-7, and HEK293T cells. According to the cytotoxicity results, **14** (IC_50_ = 2.48 μM) and **63** (IC_50_ = 3.91 μM) had the highest cytotoxicity on the MCF-7 cells. **23** showed the strongest cytotoxic effect on both PC-3 (IC_50_ = 9.40 μM) and HT-29 (IC_50_ = 12.10 μM) cell lines. In addition, **69** (IC_50_ = 12.13 μM) exhibited a strong cytotoxic effect on HT-29. The apoptotic properties of the most effective ones were evaluated on these cell lines because the selected compounds showed high cytotoxic effects on HT-29 and PC-3 cell lines. **14**, **23**, **63**, and **66** showed concentration-dependent apoptotic effects in HT-29 and MCF-7 cells. The most apoptotic compounds were **14** in MCF-7 and **23** in HT-29 cells. Additionally, both compounds decreased CA IX and CA XII protein expression in HT-29 cells, **23** and **66** showed the strongest decrement. However, **14** exhibited higher decrements of expression in MCF-7 cells.

It has been shown that hybrid molecules of specially designed coumarin-monoterpene structures with different linker and tail groups selectively inhibited the CA IX and XII and also exhibited specific cytotoxicity in different cell lines.

## 4 Experimental Section

### 4.1 Materials

The chemicals and solvents were bought from Fluka Chemie, Merck, Alfa Aesar, and Sigma-Aldrich. Melting points were determined on a STUART SMP40. IR spectra were measured on an Alfa Bruker spectrometer. ^1^H and ^13^C NMR spectra were acquired on a Varian spectrometer at 300 and at 75 Hz, respectively. Mass spectra were obtained using a Thermo Fisher Scientific LC–HRMS spectrometer. Spectrophotometric analyses were performed by a BioTek Power Wave XS (BioTek, USA). The cell line was purchased from American Type Culture Collection (ATCC). Dulbecco’s Modified Eagle’s Medium-F12, RPMI Medium, fetal calf serum, and PBS were bought from GIBCO BRL, InVitrogen (Carlsbad, CA). All compounds used for biological assays were >95% pure, as determined by the Shimadzu HPLC system with acetonitrile: 0.5% formic acid in water (70:30, v/v).

### 4.2 Methods

#### 4.2.1 General Procedures and Spectral Data

Compounds **2a–2h** and **3a–3h** were obtained using the synthesis procedures mentioned in previous studies.^20^

#### 4.2.2 Synthesis of Propargyl Monoterpenes (**8–11**)

For compounds **8–10**, the corresponding monoterpenes (thymol, carvacrol, and eugenol) (13 mmol), 1.48 mL of propargyl bromide (15.6 mmol), and 2.15 g of potassium carbonate (15.6 mmol) were taken into dry DMF and stirred at room temperature for 16 h. The mixture was poured onto crushed ice, and the residue was extracted with ethyl acetate (3 × 50 mL). The organic phase was washed with water (3 × 25 mL) and dried with sodium sulfate. The solvent was evaporated in a vacuum. Compounds **8–10** were purified by column chromatography (hexane/ethyl acetate).^20^

For compound **11**, 0.44 g of sodium hydride (10.98 mmol) was added to 10 mL of dry THF. A solution of 1.56 g menthol (9.98 mmol) dissolved in 20 mL dry THF was dropped into the sodium hydride solution in the ice bath. After the gas evolution was completed, the mixture was stirred at 70 °C for 1.5 h and cooled; then, propargyl bromide (0.90 mmol) and tetrabutylammonium iodide (TBAI, 0.20 mmol) were added to this mixture. It was stirred at room temperature for 2.5 h. At the end of the period, 4 mL of cold water was dropped, and THF was evaporated under a vacuum. The remaining oily substance was dissolved with 50 mL of ether and washed with 2 × 25 mL of water. The organic phase was dried with sodium sulfate and removed under a vacuum. The liquid residue was purified by column chromatography with petroleum ether/ether in a ratio of 1:1.^21^

##### 4.2.2.1 1-Isopropyl-4-methyl-2-(prop-2-yn-1-yloxy)benzene (**8**)

Yellow liquid, 80% yield. IR: 3292, 2960, 2122, 1612, 1505, 1244, 1035, 811, 629 cm^–1^; ^1^H NMR (CDCl_3_, 300 MHz): δ/ppm 1.26 (6H, d, *J* = 8.4 Hz), 2.39 (3H, s), 2.53–2.54 (1H, m), 3.32–3.41 (1H, m), 4.75 (2H, d, *J* = 2.3 Hz), 6.82–6.86 (2H, m), 7.17 (1H, d, *J* = 7.6 Hz); ^13^C NMR (CDCl_3_, 75 MHz): δ/ppm 21.6, 23.3, 26.6, 56.3, 75.2, 79.8, 113.2, 119.4, 125.7, 133.1, 136.0, 155.6.

##### 4.2.2.2 4-Isopropyl-1-methyl-2-(prop-2-yn-1-yloxy)benzene (**9**)

Yellow liquid, 82% yield. IR: 3291, 2959, 2122, 1613, 1511, 1239, 1033, 815, 639 cm^–1^; ^1^H NMR (CDCl_3_, 300 MHz): δ/ppm 1.24 (6H, d, *J* = 6.9 Hz), 2.20 (3H, s), 2.49–2.50 (1H, m), 2.80–2.92 (1H, m), 4.70 (2H, d, *J* = 2.3 Hz), 6.76–6.81 (2H, m), 7.06 (1H, d, *J* = 7.5 Hz); ^13^C NMR (CDCl_3_, 75 MHz): δ/ppm 16.0, 24.3, 34.2, 56.2, 75.3, 79.3,110.5, 119.3, 124.7, 130.8, 148.0, 155.9.

##### 4.2.2.3 4-Allyl-2-methoxy-1-(prop-2-yn-1-yloxy)benzene (**10**)

Yellow liquid, 76% yield. IR: 3289, 2936, 2121, 1592, 1509, 1259, 1139, 1024, 799, 640 cm^–1^; ^1^H NMR (CDCl_3_, 300 MHz): δ/ppm 2.48–2.49 (1H, m), 3.34 (2H, d, *J* = 6.4 Hz), 3.85 (3H, s, OCH_3_), 4.72 (2H, d, *J* = 2.3 Hz), 5.05–5.12 (2H, m), 5.89–6.02 (1H, m), 6.71–6.73 (2H, m), 6.97 (1H, d, *J* = 8.4 Hz); ^13^C NMR (CDCl_3_, 75 MHz): δ/ppm 40.0, 56.0, 57.1, 75.8, 79.0, 112.6, 115.0, 115.9, 120.5, 134.4, 137.7, 145.3, 149.9.

##### 4.2.2.4 1-Isopropyl-4-methyl-2-(prop-2-yn-1-yloxy)cyclohexane (**11**)

Yellow powder, 60% yield. IR: 3312, 2953, 2920, 2117, 1453, 1368, 1084, 1045, 1024, 661, 597 cm^–1^; ^1^H NMR (CDCl_3_, 300 MHz): δ/ppm 0.64 (3H, d, *J* = 6.7 Hz), 0.84–0.97 (8H, m), 1.19–1.24 (1H, m), 1.26–1.41 (1H, m), 1.55–1.67 (2H, m), 1.87 (1H, s, br), 2.12–2.21 (2H, m), 2.48–2.49 (1H, m), 3.22 (1H, td, *J* = 10.5, 4.1 Hz), 4.24 (2H, d, *J* = 2.1 Hz); ^13^C NMR (CDCl_3_, 75 MHz): δ/ppm 16.3, 21.1, 22.5, 23.5, 25.8, 31.6, 34.6, 40.4, 48.3, 49.5, 56.3, 75.7, 79.8.

#### 4.2.3 Synthesis of Coumarin-Monoterpene Derivatives Bearing a Triazole Ring (**12–43**)

1 mmol of coumarin azide (**3a–3h**) was dissolved in 4 mL of dry DMF, and 1 mmol of corresponding monoterpene propargyl derivatives (**8–11**) was added in a Schlenk tube. 2 mmol PMDETA (*N*,*N*,*N*′,*N*″,*N*″-pentamethyldiethylenetriamine) and 1 mmol CuBr were added to this mixture, and the Schlenk tube was closed. It was degassed three times. At the end of this period, THF was added to remove the copper and was filtered by the neutralized alumina. Excess THF was evaporated under a vacuum. The residue was dissolved in CH_2_Cl_2_ (50 mL) and washed with water (2 × 25 mL). The organic phase was dried with sodium sulfate, and the solvent was removed under a vacuum. The obtained compounds (**12–43**) were purified by column chromatography using hexane and ethyl acetate in a ratio of 2:1.^22^

##### 4.2.3.1 7-(2-(4-((2-Isopropyl-5-methylphenoxy)methyl)-1*H*-1,2,3-triazol-1-yl)ethoxy)-2*H*-chromen-2-one (**12**)

Whitish powder, 333 mg, starting from 200 mg of coumarin **3a**, 92% yield, mp 112–113 °C; IR: 3149, 3060, 2958, 2928, 1730, 1635, 1506, 1393, 1350, 1284, 1254, 1229, 1157, 1121, 892, 834 cm^–1^; ^1^H NMR (DMSO-*d*_6_, 300 MHz): δ/ppm 1.10 (6H, d, *J* = 9.3 Hz), 2.26 (3H, s), 3.11 (1H, m), 4.54 (2H, t, *J* = 4.6 Hz), 4.84 (2H, t, *J* = 4.6 Hz), 5.14 (2H, s), 6.30 (1H, d, *J* = 9.0 Hz), 6.71 (1H, d, *J* = 7.6 Hz), 6.88–7.05 (4H, m), 7.62 (1H, d, *J* = 8.7 Hz), 7.98 (1H, d, *J* = 9.3 Hz), 8.28 (1H, s); ^13^C NMR (DMSO-*d*_6_, 75 MHz): δ/ppm 21.6, 23.2, 26.6, 49.4, 62.1, 67.5, 102.1, 113.3, 113.4, 113.7, 122.0, 125.4, 126.3, 130.2, 134.0, 136.5, 143.9, 144.9, 155.7, 155.9, 160.8, 161.5. HRMS (ESI) *m*/*z*: calcd for C_24_H_25_N_3_O_4_ [M^+^ + Na], 442.1743; found, 442.1738.

##### 4.2.3.2 7-(3-(4-((2-Isopropyl-5-methylphenoxy)methyl)-1*H*-1,2,3-triazol-1-yl)propoxy)-2*H*-chromen-2-one (**13**)

White powder, 314 mg, starting from 200 mg of coumarin **3b**, 87% yield, mp 117–118 °C; IR: 3150, 3073, 2959, 2923, 1721, 1614, 1508, 1388, 1352, 1282, 1256, 1231, 1167, 1136, 1047, 838 cm^–1^; ^1^H NMR (CDCl_3_, 300 MHz): δ/ppm 1.15 (6H, d, *J* = 7.0 Hz), 2.31 (3H, s), 2.45–2.49 (2H, m), 3.20–3.25 (1H, m), 4.04 (2H, t, *J* = 5.8 Hz), 4.63 (2H, t, *J* = 6.7 Hz), 5.21 (2H, s), 6.26 (1H, d, *J* = 9.3 Hz), 6.76–6.83 (4H, m), 7.09 (1H, d, *J* = 8.2 Hz), 7.37 (1H, d, *J* = 8.4 Hz), 7.60 (1H, s), 7.64 (1H, d, *J* = 9.3 Hz); ^13^C NMR (CDCl_3_, 75 MHz): δ/ppm 21.5, 22.9, 26.7, 29.9, 47.2, 62.6, 64.8, 101.7, 112.7, 112.8, 113.1, 113.6, 122.0, 126.2, 129.1, 134.3, 136.7, 143.5, 155.4, 156.0, 161.3, 161.7. HRMS (ESI) *m*/*z*: calcd for C_25_H_27_N_3_O_4_ [M + Na]^+^, 456.1899; found, 456.1877.

##### 4.2.3.3 7-(4-(4-((2-Isopropyl-5-methylphenoxy)methyl)-1*H*-1,2,3-triazol-1-yl)butoxy)-2*H*-chromen-2-one (**14**)

Yellow liquid, 289 mg, starting from 200 mg of coumarin **3c**, 84% yield. IR: 3151, 3080, 2957, 2872, 1722, 1612, 1556, 1505, 1468, 1399, 1353, 1288, 1254, 1231, 1129, 1032, 836 cm^–1^; ^1^H NMR (CDCl_3_, 300 MHz): δ/ppm 1.19 (6H, d, *J* = 7.0 Hz), 1.82–1.91 (2H, m), 2.12–2.21 (2H, m), 2.32 (3H, s), 3.25–3.30 (1H, m), 4.04 (2H, t, *J* = 6.1 Hz), 4.47 (2H, t, *J* = 7.0 Hz), 5.22 (2H, s), 6.24 (1H, d, *J* = 6.6 Hz), 6.76–6.83 (4H, m), 7.10 (1H, d, *J* = 8.2 Hz), 7.36 (1H, d, *J* = 8.4 Hz), 7.60 (1H, s), 7.63 (1H, d, *J* = 9.6 Hz); ^13^C NMR (CDCl_3_, 75 MHz): δ/ppm 21.5, 23.0, 26.5, 26.8, 27.3, 30.5, 50.2, 62.7, 67.7, 101.5, 112.8, 112.9, 113.0, 113.4, 122.0, 122.4, 126.2, 129.0, 134.4, 136.7, 143.6, 145.3, 155.5, 156.0, 161.3, 162.1. HRMS (ESI) *m*/*z*: calcd for C_26_H_29_N_3_O_4_ [M + Na]^+^, 470.2056; found, 470.2034.

##### 4.2.3.4 7-((6-(4-((2-Isopropyl-5-methylphenoxy)methyl)-1*H*-1,2,3-triazol-1-yl)hexyl)oxy)-2*H*-chromen-2-one (**15**)

Yellow liquid, 412 mg, starting from 290 mg of coumarin **3d**, 85% yield. IR: 3150, 3080, 2942, 2866, 1710, 1609, 1506, 1394, 1350, 1283, 1230, 1122, 1016, 890, 810 cm^–1^; ^1^H NMR (CDCl_3_, 300 MHz): δ/ppm 1.18 (6H, d, *J* = 7.0 Hz), 1.22–1.46 (2H, m), 1.49–1.59 (2H, m), 1.76–1.86 (2H, m), 1.89–2.02 (2H, m), 2.32 (3H, s), 3.23–3.29 (1H, m), 3.99 (2H, t, *J* = 6.1 Hz), 4.39 (2H, t, *J* = 7.0 Hz), 5.21 (2H, s), 6.24 (1H, d, *J* = 9.6 Hz), 6.76–6.85 (4H, m), 7.10 (1H, d, *J* = 8.2 Hz), 7.35 (1H, d, *J* = 8.4 Hz), 7.56 (1H, s), 7.63 (1H, d, *J* = 9.3 Hz); ^13^C NMR (CDCl_3_, 75 MHz): δ/ppm 21.5, 23.0, 25.7, 25.9, 26.4, 26.8, 28.9, 30.4, 50.5, 62.7, 68.4, 101.5, 112.6, 113.0, 113.1, 113.2, 122.0, 126.2, 128.9, 134.4, 136.7, 143.6, 155.5, 156.1, 161.5, 162.4. HRMS (ESI) *m*/*z*: calcd for C_28_H_33_N_3_O_4_ [M + Na]^+^, 498.2369; found, 498.2354.

##### 4.2.3.5 7-((7-(4-((2-Isopropyl-5-methylphenoxy)methyl)-1*H*-1,2,3-triazol-1-yl)heptyl)oxy)-2*H*-chromen-2-one (**16**)

Yellow liquid, 292 mg, starting from 200 mg of coumarin **3e**, 92% yield. IR: 3150, 3060, 2924, 2856, 1720, 1607, 1504, 1391, 1347, 1277, 1250, 1227, 1118, 1093, 1015, 832, 811 cm^–1^; ^1^H NMR (CDCl_3_, 300 MHz): δ/ppm 1.18 (6H, d, *J* = 6.7 Hz), 1.38–1.43 (6H, m), 1.75–1.84 (2H, m), 1.90–1.99 (2H, m), 2.31 (3H, s), 3.25–3.30 (1H, m), 3.99 (2H, t, *J* = 6.4 Hz), 4.37 (2H, t, *J* = 7.0 Hz), 5.21 (2H, s), 6.23 (1H, d, *J* = 9.3 Hz), 6.76–6.84 (4H, m), 7.09 (1H, d, *J* = 7.6 Hz), 7.35 (1H, d, *J* = 8.4 Hz), 7.58 (1H, s), 7.63 (1H, d, *J* = 9.6 Hz); ^13^C NMR (CDCl_3_, 75 MHz): δ/ppm 20.3, 21.7, 24.7, 25.3, 25.5, 27.6, 27.7, 29.1, 29.2, 49.3, 61.4, 67.3, 100.2, 111.3, 111.7, 111.8, 111.8, 120.7, 121.1, 124.9, 127.7, 133.1, 135.4, 142.4, 143.8, 154.2, 154.8, 160.2, 161.2. HRMS (ESI) *m*/*z*: calcd for C_29_H_35_N_3_O_4_ [M + Na]^+^, 512.2525; found, 512.2501.

##### 4.2.3.6 7-((9-(4-((2-Isopropyl-5-methylphenoxy)methyl)-1*H*-1,2,3-triazol-1-yl)nonyl)oxy)-2*H*-chromen-2-one (**17**)

Yellow liquid, 226 mg, starting from 200 mg of coumarin **3f**, 72% yield. IR: 3149, 3048, 2925, 2854, 1725, 1607, 1556, 1505, 1458, 1402, 1280, 1253, 1228, 1156, 1017, 832, 809 cm^–1^; ^1^H NMR (CDCl_3_, 300 MHz): δ/ppm 1.18 (6H, d, *J* = 7.0 Hz), 1.22–1.33 (8H, m), 1.40–1.45 (2H, m), 1.74–1.81 (2H, m), 1.90–1.94 (2H, m), 2.31 (3H, s), 3.25–3.30 (1H, m), 3.99 (2H, t, *J* = 6.4 Hz), 4.36 (2H, t, *J* = 7.0 Hz), 5.21 (2H, s), 6.22 (1H, d, *J* = 9.3 Hz), 6.75–6.84 (4H, m), 7.09 (1H, d, *J* = 7.6 Hz), 7.34 (1H, d, *J* = 8.7 Hz), 7.58 (1H, s), 7.61 (1H, d, *J* = 9.6 Hz); ^13^C NMR (CDCl_3_, 75 MHz): δ/ppm 21.5, 23.0, 26.1, 26.6, 26.8, 29.1, 29.4, 29.5, 30.5, 50.6, 62.7, 68.7, 101.5, 112.5, 113.0, 113.1, 122.0, 122.4, 126.2, 129.0, 134.4, 136.6, 143.7, 144.9, 155.5, 156.1, 161.5, 162.6. HRMS (ESI) *m*/*z*: calcd for C_31_H_39_N_3_O_4_ [M + Na]^+^, 540.2838; found, 540.2830.

##### 4.2.3.7 7-((10-(4-((2-Isopropyl-5-methylphenoxy)methyl)-1*H*-1,2,3-triazol-1-yl)decyl)oxy)-2*H*-chromen-2-one (**18**)

White powder, 262 mg, starting from 200 mg of coumarin **3g**, 85% yield, mp 72–73 °C; IR: 3154, 3050, 2968, 2850, 1727, 1617, 1553, 1507, 1403, 1348, 1295, 1255, 1192, 1097, 1053, 1011, 855, 822 cm^–1^; ^1^H NMR (DMSO-*d*_6_, 300 MHz): δ/ppm 1.08 (6H, d, *J* = 7.0 Hz), 1.23–1.39 (12H, m), 1.69–1.73 (2H, m), 1.75–1.82 (2H, m), 2.26 (3H, s), 3.11–3.16 (1H, m), 4.04 (2H, t, *J* = 6.4 Hz), 4.36 (2H, t, *J* = 6.7 Hz), 5.11 (2H, s), 6.28 (1H, d, *J* = 9.3 Hz), 6.71 (1H, d, *J* = 7.6 Hz), 6.90–6.95 (3H, m), 7.03 (1H, d, *J* = 7.6 Hz), 7.60 (1H, d, *J* = 8.4 Hz), 7.97 (1H, d, *J* = 9.3 Hz), 8.18 (1H, s); ^13^C NMR (DMSO-*d*_6_, 75 MHz): δ/ppm 21.6, 23.2, 26.0, 26.4, 26.7, 29.0, 29.1, 29.3, 29.5, 30.3, 50.0, 62.2, 68.9, 101.7, 112.8, 113.0, 113.3, 113.8, 122.0, 124.7, 126.2, 130.1, 134.0, 136.5, 143.7, 145.0, 155.7, 156.1, 160.9, 1612.5. HRMS (ESI) *m*/*z*: calcd for C_32_H_41_N_3_O_4_ [M + Na]^+^, 554.2995; found, 554.2980.

##### 4.2.3.8 7-((12-(4-((2-Isopropyl-5-methylphenoxy)methyl)-1*H*-1,2,3-triazol-1-yl)dodecyl)oxy)-2*H*-chromen-2-one (**19**)

Yellow powder, 199 mg, starting from 200 mg of coumarin **3h**, 66% yield, mp 68–69 °C; IR: 3142, 3048, 2959, 2850, 1727, 1618, 1578, 1553, 1505, 1469, 1379, 1255, 1118, 1091, 1046, 807 cm^–1^; ^1^H NMR (CDCl_3_, 300 MHz): δ/ppm 1.18 (6H, d, *J* = 7.0 Hz), 1.27–1.46 (16H, m), 1.74–1.83 (2H, m), 1.85–1.93 (2H, m), 2.32 (3H, s), 3.25–3.30 (1H, m), 4.00 (2H, t, *J* = 6.4 Hz), 4.35 (2H, t, *J* = 7.3 Hz), 5.21 (2H, s), 6.23 (1H, d, *J* = 9.3 Hz), 6.76–6.84 (4H, m), 7.10 (1H, d, *J* = 7.9 Hz), 7.36 (1H, d, *J* = 8.4 Hz), 7.55 (1H, s), 7.63 (1H, d, *J* = 9.3 Hz); ^13^C NMR (CDCl_3_, 75 MHz): δ/ppm 21.6, 23.0, 26.1, 26.7, 26.8, 29.2, 29.5, 29.6, 29.7, 30.5, 50.6, 62.7, 68.8, 101.4, 112.9, 113.1, 113.2, 122.0, 122.3, 126.2, 128.9, 134.4, 136.7, 143.7, 145.0, 155.5, 156.1, 161.5, 162.6. HRMS (ESI) *m*/*z*: calcd for C_34_H_45_N_3_O_4_ [M + Na]^+^, 582.3308; found, 582.3282.

##### 4.2.3.9 7-(2-(4-((5-Isopropyl-2-methylphenoxy)methyl)-1*H*-1,2,3-triazol-1-yl)ethoxy)-2*H*-chromen-2-one (**20**)

Whitish powder, 246 mg, starting from 200 mg of coumarin **3a**, 68% yield, mp 105–106 °C; IR: 3157, 3052, 2964, 2873, 1708, 1612, 1581, 1508, 1402, 1350, 1285, 1254, 1234, 1204, 1126, 1050, 896, 836, cm^–1^; ^1^H NMR (CDCl_3_, 300 MHz): δ/ppm 1.21 (6H, d, *J* = 6.7 Hz), 2.19 (3H, s), 2.83–2.87 (1H, m), 4.45 (2H, t, *J* = 4.9 Hz), 4.82 (2H, t, *J* = 4.9 Hz), 5.24 (2H, s), 6.27 (1H, d, *J* = 9.3 Hz), 6.74–6.79 (4H, m), 7.06 (1H, d, *J* = 7.6 Hz), 7.37 (1H, d, *J* = 9.3 Hz), 7.63 (1H, d, *J* = 9.3 Hz), 7.78 (1H, s); ^13^C NMR (CDCl_3_, 75 MHz): δ/ppm 16.1, 24.3, 34.2, 49.6, 62.5, 66.9, 102.0, 110.2, 112.6, 113.5, 114.0, 119.0, 123.7, 124.4, 129.2, 130.8, 143.3, 145.4, 148.2, 155.9, 156.4, 160.9, 161.0. HRMS (ESI) *m*/*z*: calcd for C_24_H_25_N_3_O_4_ [M + Na]^+^, 442.1743; found, 442.1732.

##### 4.2.3.10 7-(3-(4-((5-Isopropyl-2-methylphenoxy)methyl)-1*H*-1,2,3-triazol-1-yl)propoxy)-2*H*-chromen-2-one (**21**)

White powder, 254 mg, starting from 200 mg of coumarin **3b**, 72% yield, mp 108–109 °C; IR: 3138, 3065, 2947, 2864, 1710, 1611, 1511, 1461, 1400, 1353, 1279, 1233, 1206, 1125, 1021, 938, 870, 834 cm^–1^; ^1^H NMR (CDCl_3_, 300 MHz): δ/ppm 1.22 (6H, d, *J* = 7.0 Hz), 2.14 (3H, s), 2.42–2.50 (2H, m), 2.83–2.88 (1H, m), 4.03 (2H, t, *J* = 5.5 Hz), 4.62 (2H, t, *J* = 7.0 Hz), 5.23 (2H, s), 6.25 (1H, dd, *J* = 4.9, 9.6 Hz), 6.74–6.87 (4H, m), 7.05 (1H, d, *J* = 7.6 Hz), 7.37 (1H, d, *J* = 8.2 Hz), 7.59 (1H, s), 7.63 (1H, d, *J* = 9.6 Hz); ^13^C NMR (CDCl_3_, 75 MHz): δ/ppm 16.0, 24.3, 29.8, 34.2, 47.2, 62.5, 64.8, 101.8, 110.2, 112.7, 113.1, 113.7, 118.9, 122.9, 124.3, 129.1, 130.8, 143.5, 145.3, 148.2, 156.0, 156.4, 161.7. HRMS (ESI) *m*/*z*: calcd for C_25_H_27_N_3_O_4_ [M + Na]^+^, 456.1899; found, 456.1878.

##### 4.2.3.11 7-(4-(4-((5-Isopropyl-2-methylphenoxy)methyl)-1*H*-1,2,3-triazol-1-yl)butoxy)-2*H*-chromen-2-one (**22**)

Yellow powder, 230 mg, starting from 200 mg of coumarin **3c**, 96% yield, mp 60–61 °C; IR: 3140, 3082, 2958, 2872, 1726, 1610, 1556, 1508, 1460, 1400, 1350, 1280, 1250, 1231, 1161, 1122, 1035, 892, 834 cm^–1^; ^1^H NMR (CDCl_3_, 300 MHz): δ/ppm 1.23 (6H, d, *J* = 7.0 Hz), 1.81–1.90 (2H, m), 2.11–2.16 (2H, m), 2.19 (3H, s), 2.81–2.91 (1H, m), 4.04 (2H, t, *J* = 6.1 Hz), 4.47 (2H, t, *J* = 7.0 Hz), 5.24 (2H, s), 6.24 (1H, d, *J* = 9.3 Hz), 6.75–6.83 (4H, m), 7.07 (1H, d, *J* = 7.3 Hz), 7.36 (1H, d, *J* = 8.4 Hz), 7.61 (1H, s), 7.64 (1H, s); ^13^C NMR (CDCl_3_, 75 MHz): δ/ppm 16.1, 24.3, 26.2, 27.3, 30.5, 34.3, 50.1, 62.7, 67.7, 101.5, 110.3, 112.8, 112.9, 113.4, 118.9, 122.4, 124.4, 129.0, 130.8, 143.6, 145.3, 148.3, 156.0, 156.5, 161.4, 162.1. HRMS (ESI) *m*/*z*: calcd for C_26_H_29_N_3_O_4_ [M + Na]^+^, 470.2056; found, 470.2034.

##### 4.2.3.12 7-((6-(4-((5-Isopropyl-2-methylphenoxy)methyl)-1*H*-1,2,3-triazol-1-yl)hexyl)oxy)-2*H*-chromen-2-one (**23**)

Yellow powder, 152 mg, starting from 200 mg of coumarin **3d**, 46% yield, mp 132–133 °C; IR: 3130, 3079, 2948, 2874, 1710, 1609, 1556, 1507, 1393, 1351, 1284, 1236, 1202, 1160, 1124, 1020, 855, 811 cm^–1^; ^1^H NMR (CDCl_3_, 300 MHz): δ/ppm 1.23 (6H, d, *J* = 7.0 Hz), 1.35–1.59 (4H, m), 1.80 (2H, t, *J* = 6.7 Hz), 1.96 (2H, t, *J* = 7.3 Hz), 2.19 (3H, s), 2.81–2.88 (1H, m), 3.99 (2H, t, *J* = 6.1 Hz), 4.38 (2H, t, *J* = 7.3 Hz), 5.23 (2H, s), 6.23 (1H, d, *J* = 9.3 Hz), 6.74–6.85 (4H, m), 7.06 (1H, d, *J* = 7.6 Hz), 7.35 (1H, dd, *J* = 3.5; 8.4 Hz), 7.57–7.64 (2H, m); ^13^C NMR (CDCl_3_, 75 MHz): δ/ppm 16.1, 24.3, 25.6, 26.4, 28.9, 30.4, 34.2, 50.4, 62.7, 68.4, 101.5, 110.3, 112.6, 113.1, 118.9, 122.4, 124.4, 129.0, 130.7, 143.7, 145.1, 148.2, 156.1, 156.5, 161.4, 162.4. HRMS (ESI) *m*/*z*: calcd for C_28_H_33_N_3_O_4_ [M + Na]^+^, 498.2369; found, 498.2350.

##### 4.2.3.13 7-((7-(4-((5-Isopropyl-2-methylphenoxy)methyl)-1*H*-1,2,3-triazol-1-yl)heptyl)oxy)-2*H*-chromen-2-one (**24**)

White powder, 243 mg, starting from 200 mg of coumarin **3e**, 75% yield, mp 83–84 °C; IR: 3112, 3071, 2947, 2869, 1730, 1610, 1559, 1510, 1398, 1351, 1286, 1245, 1230, 1155, 997, 830 cm^–1^; ^1^H NMR (CDCl_3_, 300 MHz): δ/ppm 1.23 (6H, d, *J* = 7.3 Hz), 1.38–1.43 (6H, m), 1.75–1.82 (2H, m), 1.89–1.96 (2H, m), 2.19 (3H, s), 2.84–2.88 (1H, m), 3.99 (2H, t, *J* = 6.4 Hz), 4.37 (2H, t, *J* = 7.3 Hz), 5.24 (2H, s), 6.24 (1H, d, *J* = 9.3 Hz), 6.75–6.84 (4H, m), 7.06 (1H, d, *J* = 7.6 Hz), 7.35 (1H, d, *J* = 8.4 Hz), 7.57 (1H, s), 7.63 (1H, d, *J* = 9.6 Hz); ^13^C NMR (CDCl_3_, 75 MHz): δ/ppm 16.1, 24.3, 26.0, 26.6, 28.9, 29.0, 30.4, 34.3, 50.5, 62.7, 68.6, 101.4, 110.2, 112.6, 113.2, 118.9, 122.3, 124.4, 128.9, 130.7, 143.7, 145.1, 148.2, 156.1, 156.5, 161.5, 162.5. HRMS (ESI) *m*/*z*: calcd for C_29_H_35_N_3_O_4_ [M + Na]^+^, 512.2525; found, 512.2504.

##### 4.2.3.14 7-((9-(4-((5-Isopropyl-2-methylphenoxy)methyl)-1*H*-1,2,3-triazol-1-yl)nonyl)oxy)-2*H*-chromen-2-one (**25**)

White powder, 194 mg, starting from 200 mg of coumarin **3f**, 62% yield, mp 69–70 °C; IR: 3114, 3073, 2923, 2872, 1733, 1611, 1509, 1473, 1397, 1287, 1247, 1229, 1177, 1057, 1015, 856 cm^–1^; ^1^H NMR (CDCl_3_, 300 MHz): δ/ppm 1.23 (6H, d, *J* = 7.0 Hz), 1.33–1.38 (8H, m), 1.40–1.45 (2H, m), 1.77–1.82 (2H, m), 1.89–1.94 (2H, m), 2.19 (3H, s), 2.84–2.88 (1H, m), 4.00 (2H, t, *J* = 6.4 Hz), 4.36 (2H, t, *J* = 7.3 Hz), 5.24 (2H, s), 6.23(1H, d, *J* = 9.3 Hz), 6.75–6.84 (4H, m), 7.06 (1H, d, *J* = 7.6 Hz), 7.36 (1H, d, *J* = 8.4 Hz), 7.57 (1H, s), 7.63 (1H, d, *J* = 9.3 Hz); ^13^C NMR (CDCl_3_, 75 MHz): δ/ppm 16.1, 24.3, 26.1, 26.6, 29.1, 29.4, 29.5, 30.5, 34.3, 50.6, 62.7, 68.7, 101.5, 110.3, 112.5, 113.1, 113.2, 118.9, 122.3, 124.4, 128.9, 130.7, 143.7, 145.1, 148.2, 156.1, 156.5, 161.5, 162.6. HRMS (ESI) *m*/*z*: calcd for C_31_H_39_N_3_O_4_ [M + Na]^+^, 540.2838; found, 540.2826.

##### 4.2.3.15 7-((10-(4-((5-Isopropyl-2-methylphenoxy)methyl)-1*H*-1,2,3-triazol-1-yl)decyl)oxy)-2*H*-chromen-2-one (**26**)

Yellow powder, 300 mg, starting from 200 mg of coumarin **3g**, 97% yield, mp 73–74 °C; IR: 3163, 3070, 2953, 2851, 1725, 1616, 1553, 1510, 1468, 1403, 1295, 1254, 1132, 1050, 1012, 891, 835 cm^–1^; ^1^H NMR (CDCl_3_, 300 MHz): δ/ppm 1.15 (6H, d, *J* = 6.7 Hz), 1.17–1.24 (8H, m), 1.32–1.40 (4H, m), 1.70–1.77 (2H, m), 1.81–1.86 (2H, m), 2.12 (3H, s), 2.74–2.81 (1H, m), 3.92 (2H, t, *J* = 6.4 Hz), 4.28 (2H, t, *J* = 7.3 Hz), 5.16 (2H, s), 6.17 (1H, d, *J* = 9.3 Hz), 6.67–6.77 (4H, m), 7.0 (1H, d, *J* = 7.6 Hz), 7.28 (1H, d, *J* = 8.4 Hz), 7.49 (1H, s), 7.55 (1H, d, *J* = 9.3 Hz); ^13^C NMR (CDCl_3_, 75 MHz): δ/ppm 14.9, 23.0, 24.8, 25.4, 27.9, 28.2, 28.3, 29.2, 33.0, 49.3, 61.4, 67.5, 100.2, 109.0, 111.3, 111.8, 111.9, 117.6, 121.0, 123.1, 127.6, 129.5, 142.5, 143.8, 147.0, 154.8, 155.2, 160.3, 161.3. HRMS (ESI) *m*/*z*: calcd for C_32_H_41_N_3_O_4_ [M + Na]^+^, 554.2995; found, 554.2971.

##### 4.2.3.16 7-((12-(4-((5-Isopropyl-2-methylphenoxy)methyl)-1*H*-1,2,3-triazol-1-yl)dodecyl)oxy)-2*H*-chromen-2-one (**27**)

Whitish powder, 168 mg, starting from 200 mg of coumarin **3h**, 56% yield, mp 80–81 °C; IR: 3163, 3060, 2918, 2850, 1726, 1618, 1553, 1511, 1467, 1403, 1296, 1254, 1237, 1133, 1049, 833 cm^–1^; ^1^H NMR (CDCl_3_, 300 MHz): δ/ppm 1.17–1.46 (22H, m), 1.76–1.93 (4H, m), 2.19 (3H, s), 2.84–2.88 (1H, m), 4.01 (2H, t, *J* = 6.4 Hz), 4.35 (2H, t, *J* = 7.0 Hz), 5.23 (2H, s), 6.23 (1H, d, *J* = 9.3 Hz), 6.75–6.84 (4H, m), 7.06 (1H, d, *J* = 7.6 Hz), 7.36 (1H, d, *J* = 8.4 Hz), 7.57 (1H, s), 7.63 (1H, d, *J* = 9.3 Hz); ^13^C NMR (CDCl_3_, 75 MHz): δ/ppm 16.1, 24.3, 26.1, 26.7, 29.2, 29.5, 29.6, 29.7, 30.5, 34.3, 50.6, 62.7, 68.8, 101.5, 110.2, 112.5, 113.1, 113.2, 118.9, 122.3, 124.4, 128.9, 130.7, 143.7, 145.0, 148.2, 156.1, 156.5, 161.5, 162.6. HRMS (ESI) *m*/*z*: calcd for C_34_H_45_N_3_O_4_ [M + Na]^+^, 582.3308; found, 582.3282.

##### 4.2.3.17 7-(2-(4-((4-Allyl-2-methoxyphenoxy)methyl)-1*H*-1,2,3-triazol-1-yl)ethoxy)-2*H*-chromen-2-one (**28**)

Whitish powder, 194 mg, starting from 200 mg of coumarin **3a**, 52% yield, mp 118–119 °C; IR: 3149, 3083, 2957, 2877, 1708, 1612, 1518, 1456, 1399, 1348, 1281, 1234, 1155, 1126, 1038, 997, 836 cm^–1^; ^1^H NMR (CDCl_3_, 300 MHz): δ/ppm 3.32 (2H, d, *J* = 6.7 Hz), 3.84 (3H, s), 4.42 (2H, t, *J* = 5.2 Hz), 4.79 (2H, t, *J* = 4.6 Hz), 5.03–5.10 (2H, m), 5.27 (2H, s), 5.86–5.98 (1H, m), 6.27 (1H, d, *J* = 9.6 Hz), 6.66–6.88 (4H, m), 6.95 (1H, d, *J* = 8.3 Hz), 7.36 (1H, d, *J* = 7.9 Hz), 7.63 (1H, d, *J* = 9.3 Hz), 7.84(1H, s); ^13^C NMR (CDCl_3_, 75 MHz): δ/ppm 40.0, 49.6, 56.0, 63.4, 66.9, 101.9, 112.4, 112.6, 113.4, 113.9, 114.4, 116.0, 120.6, 124.2, 129.2, 134.0, 137.7, 143.4, 144.9, 146.0, 149.6, 155.8, 160.9, 161.1. HRMS (ESI) *m*/*z*: calcd for C_24_H_23_N_3_O_5_ [M + Na]^+^, 456.1535; found, 456.1516.

##### 4.2.3.18 7-(3-(4-((4-Allyl-2-methoxyphenoxy)methyl)-1*H*-1,2,3-triazol-1-yl)propoxy)-2*H*-chromen-2-one (**29**)

Whitish powder, 240 mg, starting from 200 mg of coumarin **3b**, 66% yield, mp 88–89 °C; IR: 3139, 3078, 2964, 2876, 1723, 1614, 1591, 1467, 1396, 1351, 1265, 1227, 1137, 1056, 1011, 858, 832 cm^–1^; ^1^H NMR (CDCl_3_, 300 MHz): δ/ppm 2.40–2.48 (2H, m), 3.32 (2H, d, *J* = 6.7 Hz), 3.84 (3H, s), 4.03 (2H, t, *J* = 5.5 Hz), 4.58 (2H, t, *J* = 6.7 Hz), 5.04–5.10 (2H, m), 5.26 (2H, s), 5.89–5.98 (1H, m), 6.27 (1H, d, *J* = 9.6 Hz), 6.67–6.87 (4H, m), 6.94 (1H, d, *J* = 7.9 Hz), 7.37 (1H, d, *J* = 8.4 Hz), 7.63 (1H, d, *J* = 9.6 Hz), 7.65 (1H, s); ^13^C NMR (CDCl_3_, 75 MHz): δ/ppm 29.8, 40.0, 47.2, 56.0, 63.5, 64.9, 101.8, 112.4, 112.7, 113.1, 113.6, 114.3, 116.0, 120.7, 123.4, 129.1, 134.0, 137.7, 143.5, 144.9, 146.0, 149.6, 156.0, 161.3, 161.7. HRMS (ESI) *m*/*z*: calcd for C_25_H_25_N_3_O_5_ [M + Na]^+^, 470.1692; found, 470.1673.

##### 4.2.3.19 7-(4-(4-((4-Allyl-2-methoxyphenoxy)methyl)-1*H*-1,2,3-triazol-1-yl)butoxy)-2*H*-chromen-2-one (**30**)

Yellow powder, 255 mg, starting from 200 mg of coumarin **3c**, 72% yield, mp 73–74 °C; IR: 3150, 3072, 2960, 2866, 1723, 1613, 1553, 1515, 1471, 1400, 1292, 1256, 1233, 1190, 1133, 1097, 1057, 1011, 836 cm^–1^; ^1^H NMR (CDCl_3_, 300 MHz): δ/ppm 1.80–1.89 (2H, m), 2.09–2.18 (2H, m), 3.32 (2H, d, *J* = 6.7 Hz), 3.86 (3H, s), 4.03 (2H, t, *J* = 5.8 Hz), 4.47 (2H, t, *J* = 7.0 Hz), 5.04–5.10 (2H, m), 5.27 (2H, s), 5.87–5.99 (1H, m), 6.25 (1H, d, *J* = 9.3 Hz), 6.68–6.83 (4H, m), 6.96 (1H, d, *J* = 7.9 Hz), 7.36 (1H, d, *J* = 8.4 Hz), 7.63 (1H, d, *J* = 9.6 Hz), 7.68 (1H, s); ^13^C NMR (CDCl_3_, 75 MHz): δ/ppm 26.2, 27.3, 40.0, 50.1, 56.0, 63.5, 67.7, 101.5, 112.3, 112.8, 112.9, 113.4, 114.4, 115.9, 120.7, 129.0, 134.0, 137.7, 143.6, 146.0, 149.6, 156.0, 161.4, 162.1. HRMS (ESI) *m*/*z*: calcd for C_26_H_27_N_3_O_5_ [M + Na]^+^, 484.1848; found, 484.1826.

##### 4.2.3.20 7-((6-(4-((4-Allyl-2-methoxyphenoxy)methyl)-1*H*-1,2,3-triazol-1-yl)hexyl)oxy)-2*H*-chromen-2-one (**31**)

Whitish liquid, 272 mg, starting from 200 mg of coumarin **3d**, 80% yield. IR: 3149, 3074, 2942, 2865, 1746, 1611, 1554, 1509, 1464, 1405, 1338, 1290, 1258, 1126, 1025, 837 cm^–1^; ^1^H NMR (CDCl_3_, 300 MHz): δ/ppm 1.39–1.54 (4H, m), 1.75–1.84 (2H, m), 1.92–1.97 (2H, m), 3.32 (2H, d, *J* = 6.7 Hz), 3.85 (3H, s), 3.99 (2H, t, *J* = 6.4 Hz), 4.35 (2H, t, *J* = 7.0 Hz), 5.04–5.10 (2H, m), 5.26 (2H, s), 5.90–5.99 (1H, m), 6.24 (1H, d, *J* = 9.3 Hz), 6.68–6.72 (2H, m), 6.78–6.84 (2H, m), 6.95 (1H, d, *J* = 7.9 Hz), 7.35 (1H, d, *J* = 8.4 Hz), 7.62 (1H, s), 7.63 (1H, d, *J* = 9.3 Hz); ^13^C NMR (CDCl_3_, 75 MHz): δ/ppm 24.4, 25.1, 27.7, 29.1, 38.7, 49.2, 54.8, 62.3, 67.2, 100.2, 111.1, 111.4, 111.8, 111.9, 113.2, 114.7, 119.4, 121.6, 127.7, 132.7, 136.4, 142.4, 143.4, 144.8, 148.3, 154.8, 160.2, 161.2. HRMS (ESI) *m*/*z*: calcd for C_28_H_31_N_3_O_5_ [M + Na]^+^, 512.2161; found, 512.2146.

##### 4.2.3.21 7-((7-(4-((4-Allyl-2-methoxyphenoxy)methyl)-1*H*-1,2,3-triazol-1-yl)heptyl)oxy)-2*H*-chromen-2-one (**32**)

Yellow powder, 300 mg, starting from 200 mg of coumarin **3e**, 90% yield, mp 101–102 °C; IR: 3145, 3070, 2966, 2858, 1726, 1609, 1512, 1471, 1426, 1398, 1283, 1251, 1229, 1137, 1122, 1026, 864, 824 cm^–1^; ^1^H NMR (CDCl_3_, 300 MHz): δ/ppm 1.25–1.47 (6H, m), 1.74–1.79 (2H, m), 1.82–1.94 (2H, m), 3.32 (2H, d, *J* = 6.7 Hz), 3.86 (3H, s), 3.99 (2H, t, *J* = 6.4 Hz), 4.34 (2H, t, *J* = 7.3 Hz), 5.02–5.11 (2H, m), 5.27 (2H, s), 5.90–5.99 (1H, m), 6.23 (1H, d, *J* = 9.3 Hz), 6.68–6.72 (2H, m), 6.78–6.84 (2H, m), 6.95 (1H, d, *J* = 7.9 Hz), 7.35 (1H, d, *J* = 8.4 Hz), 7.62 (1H, s), 7.63 (1H, d, *J* = 8.4 Hz); ^13^C NMR (CDCl_3_, 75 MHz): δ/ppm 26.0, 26.6, 29.0, 30.4, 30.5, 40.0, 50.5, 56.0, 63.6, 68.6, 101.4, 112.3, 112.6, 113.2, 114.4, 115.9, 120.7, 122.8, 125.7, 128.9, 133.9, 137.7, 143.7, 144.7, 146.1, 149.6, 156.1, 161.5, 162.5. HRMS (ESI) *m*/*z*: calcd for C_29_H_33_N_3_O_5_ [M + Na]^+^, 526.2318; found, 526.2295.

##### 4.2.3.22 7-((9-(4-((4-Allyl-2-methoxyphenoxy)methyl)-1*H*-1,2,3-triazol-1-yl)nonyl)oxy)-2*H*-chromen-2-one (**33**)

Yellow powder, 193 mg, starting from 200 mg of coumarin **3f**, 60% yield, mp 83–84 °C; IR: 3136, 3072, 2995, 2850, 1730, 1611, 1512, 1473, 1398, 1350, 1287, 1258, 1231, 1036, 1015, 857, 826 cm^–1^; ^1^H NMR (CDCl_3_, 300 MHz): δ/ppm 1.32–1.47 (10H, m), 1.75–1.87 (4H, m), 3.32 (2H, d, *J* = 6.4 Hz), 3.85 (3H, s), 4.00 (2H, t, *J* = 6.7 Hz), 4.32 (2H, t, *J* = 7.3 Hz), 5.04–5.11 (2H, m), 5.26 (2H, s), 5.87–6.01 (1H, m), 6.23 (1H, d, *J* = 9.6 Hz), 6.67–6.72 (2H, m), 6.79–6.85 (2H, m), 6.96 (1H, d, *J* = 7.9 Hz), 7.36 (1H, d, *J* = 8.4 Hz), 7.63 (1H, d, *J* = 9.3 Hz), 7.65 (1H, s); ^13^C NMR (CDCl_3_, 75 MHz): δ/ppm 26.1, 26.6, 29.1, 29.3, 29.4, 30.4, 40.0, 50.6, 56.0, 63.6, 68.7, 101.5, 112.4, 112.5, 113.1, 113.2, 114.4, 115.9, 120.7, 122.8, 128.9, 133.9, 137.7, 143.7, 144.6, 146.1, 149.6, 156.1, 161.5, 162.6. HRMS (ESI) *m*/*z*: calcd for C_31_H_37_N_3_O_5_ [M + Na]^+^], 554.2634; found, 554.2606.

##### 4.2.3.23 7-((10-(4-((4-Allyl-2-methoxyphenoxy)methyl)-1*H*-1,2,3-triazol-1-yl)decyl)oxy)-2*H*-chromen-2-one (**34**)

Yellow powder, 273 mg, starting from 200 mg of coumarin **3g**, 86% yield, mp 48–49 °C; IR: 3130, 3080, 2942, 2851, 1725, 1614, 1510, 1468, 1395, 1294, 1258, 1230, 1194, 1131, 1099, 1035, 1015, 835 cm^–1^; ^1^H NMR (CDCl_3_, 300 MHz): δ/ppm 1.12–1.34 (8H, m), 1.40–1.48 (4H, m), 1.71–1.91 (4H, m), 3.32 (2H, d, *J* = 6.4 Hz), 3.86 (3H, s), 4.00 (2H, t, *J* = 6.4 Hz), 4.32 (2H, t, *J* = 7.3 Hz), 5.02–5.11 (2H, m), 5.27 (2H, s), 5.90–5.99 (1H, m), 6.23 (1H, d, *J* = 9.6 Hz), 6.68–6.74 (2H, m), 6.79–6.85 (2H, m), 6.95 (1H, d, *J* = 7.9 Hz), 7.35 (1H, d, *J* = 8.4 Hz), 7.61 (1H, s), 7.63 (1H, d, *J* = 9.6 Hz); ^13^C NMR (CDCl_3_, 75 MHz): δ/ppm 24.8, 25.4, 27.9, 28.2, 28.2, 28.3, 29.2, 29.2, 38.7, 49.3, 54.8, 62.4, 67.5, 100.0, 111.1, 111.3, 111.8, 111.9, 113.2, 114.7, 119.4, 121.5, 127.6, 132.6, 136.4, 142.4, 143.4, 144.8, 148.3, 154.8, 160.3, 161.3. HRMS (ESI) *m*/*z*: calcd for C_32_H_39_N_3_O_5_ [M + Na]^+^, 568.2787; found, 568.2762.

##### 4.2.3.24 7-((12-(4-((4-Allyl-2-methoxyphenoxy)methyl)-1*H*-1,2,3-triazol-1-yl)dodecyl)oxy)-2*H*-chromen-2-one (**35**)

Yellow powder, 258 mg, starting from 200 mg of coumarin **3h**, 84% yield, mp 72–74 °C; IR: 3137, 3084, 2917, 2849, 1710, 1617, 1589, 1556, 1402, 1373, 1296, 1229, 1195, 1134, 1051, 994, 850 cm^–1^; ^1^H NMR (DMSO-*d*_6_, 300 MHz): δ/ppm 1.28–1.45 (16H, m), 1.74–1.86 (4H, m), 3.34 (2H, d, *J* = 6.7 Hz), 3.77 (3H, s), 4.10 (2H, t, *J* = 6.4 Hz), 4.39 (2H, t, *J* = 7.0 Hz), 5.06–5.14 (2H, m), 5.11 (2H, s), 5.94–6.03 (1H, m), 6.33 (1H, d, *J* = 9.3 Hz), 6.72 (1H, d, *J* = 7.9 Hz), 6.84 (1H, s), 6.92–7.08 (4H, m), 7.66 (1H, d, *J* = 8.7 Hz), 8.03 (1H, d, *J* = 9.3 Hz), 8.24 (1H, s); ^13^C NMR (DMSO-*d*_6_, 75 MHz): δ/ppm 26.0, 26.4, 29.0, 29.3, 29.5, 29.6, 30.3, 31.0, 35.0, 50.0, 55.9, 62.5, 68.9, 101.7, 112.8, 112.9, 113.0, 113.3, 114.6, 116.2, 120.7, 125.0, 130.1, 133.5, 138.5, 143.4, 145.0, 146.3, 149.7, 156.0, 161.0, 162.5. HRMS (ESI) *m*/*z*: calcd for C_34_H_43_N_3_O_5_ [M + Na]^+^, 596.3100; found, 596.3070.

##### 4.2.3.25 7-(2-(4-(((2-isopropyl-5-methylcyclohexyl)oxy)methyl)-1*H*-1,2,3-triazol-1-yl)ethoxy)-2*H*-chromen-2-one (**36**)

Yellow powder, 316 mg, starting from 200 mg of coumarin **3a**, 86% yield, mp 122–123 °C; IR: 3136, 3065, 2956, 2867, 1729, 1619, 1557, 1455, 1404, 1298, 1242, 1140, 1083, 1037, 834 cm^–1^; ^1^H NMR (CDCl_3_, 300 MHz): δ/ppm 0.63 (3H, d, *J* = 6.7 Hz), 0.84–0.97 (8H, m), 1.19–1.23 (1H, m), 1.26–1.41 (1H, m), 1.57–1.67 (2H, m), 1.87 (1H, s, br), 2.12–2.21 (2H, m), 3.20 (1H, td, *J* = 10.5, 4.1 Hz), 4.43 (2H, t, *J* = 5.2 Hz), 4.57 (2H, t, *J* = 12.0 Hz), 4.79 (1H, d, *J* = 11.7 Hz), 4.81 (2H, t, *J* = 4.3 Hz), 6.27 (1H, d, *J* = 9.3 Hz), 6.79–6.83 (2H, m), 7.38 (1H, d, *J* = 8.2 Hz), 7.63 (1H, d, *J* = 9.6 Hz), 7.73 (1H, s); ^13^C NMR (CDCl_3_, 75 MHz): δ/ppm 16.3, 21.1, 22.5, 23.5, 25.8, 31.6, 34.6, 40.4, 48.3, 49.5, 62.1, 67.0, 79.0, 101.9, 112.6,113.4, 114.0, 123.6, 129.2, 143.4, 146.7, 155.9, 161.0, 161.1. HRMS (ESI) *m*/*z*: calcd for C_24_H_31_N_3_O_4_ [M + Na]^+^, 448.2212; found, 448.2192.

##### 4.2.3.26 7-(3-(4-(((2-Isopropyl-5-methylcyclohexyl)oxy)methyl)-1*H*-1,2,3-triazol-1-yl)propoxy)-2*H*-chromen-2-one (**37**)

Yellow powder, 308 mg, starting from 200 mg of coumarin **3b**, 86% yield, mp 78–79 °C; IR: 3133, 3067, 2944, 2866, 1710, 1616, 1557, 1510, 1463, 1400, 1352, 1281, 1233, 1159, 1130, 1071, 1047, 832 cm^–1^; ^1^H NMR (CDCl_3_, 300 MHz): δ/ppm 0.65 (3H, d, *J* = 6.7 Hz), 0.76–1.14 (7H, m), 1.18–1.37 (3H, m), 1.57–1.66 (2H, m), 1.82 (1H, s, br), 2.04–2.21 (2H, m), 2.40–2.49 (2H, m), 3.19 (1H, td, *J* = 10.5, 4.3 Hz), 4.05 (2H, t, *J* = 5.8 Hz), 4.53–4.61 (3H, m), 4.77 (1H, d, *J* = 12.0 Hz), 6.26 (1H, d, *J* = 9.6 Hz), 6.78–6.84 (2H, m), 7.38 (1H, d, *J* = 8.4 Hz), 7.55 (1H, s), 7.63 (1H, d, *J* = 9.3 Hz); ^13^C NMR (CDCl_3_, 75 MHz): δ/ppm 16.3, 21.1, 22.5, 23.5, 25.8, 29.9, 31.6, 34.6, 40.4, 47.0, 48.3, 62.1, 64.9, 79.0, 101.8, 112.7, 113.1, 113.6, 122.8, 129.1, 143.5, 146.5, 155.9, 161.2, 161.7. HRMS (ESI) *m*/*z*: calcd for C_25_H_33_N_3_O_4_ [M + Na]^+^, 462.2369; found, 462.2349.

##### 4.2.3.27 7-(4-(4-(((2-Isopropyl-5-methylcyclohexyl)oxy)methyl)-1*H*-1,2,3-triazol-1-yl)butoxy)-2*H*-chromen-2-one (**38**)

White powder, 322 mg, starting from 200 mg of coumarin **3c**, 92% yield, mp 75–76 °C; IR: 3135, 3068, 2948, 2867, 1707, 1615, 1556, 1468, 1403, 1354, 1289, 1255, 1131, 1084, 1050, 862 cm^–1^; ^1^H NMR (CDCl_3_, 300 MHz): δ/ppm 0.68 (3H, d, *J* = 7.0 Hz), 0.82–0.99 (8H, m), 1.20–1.60 (3H, m), 1.62–1.66 (2H, m), 1.67–1.91 (2H, m), 2.09–2.21 (4H, m), 3.20 (1H, td, *J* = 10.5, 4.3 Hz), 4.04 (2H, t, *J* = 6.1 Hz), 4.45 (2H, t, *J* = 7.0 Hz), 4.57 (1H, d, *J* = 12.0 Hz), 4.78 (1H, d, *J* = 12.3 Hz), 6.25 (1H, d, *J* = 9.3 Hz), 6.77–6.84 (2H, m), 7.37 (1H, d, *J* = 8.4 Hz), 7.57 (1H, s), 7.64 (1H, d, *J* = 9.3 Hz); ^13^C NMR (CDCl_3_, 75 MHz): δ/ppm 16.4, 21.2, 22.5, 23.4, 25.8, 26.2, 27.3, 31.6, 34.7, 40.4, 48.3, 50.0, 62.2, 67.7, 79.0, 101.5, 112.8, 112.9, 113.3, 122.4, 129.0, 143.6, 146.6, 156.0, 161.4, 162.1. HRMS (ESI) *m*/*z*: calcd for C_26_H_35_N_3_O_4_ [M + Na]^+^, 476.2525; found, 476.2504.

##### 4.2.3.28 7-((6-(4-(((2-Isopropyl-5-methylcyclohexyl)oxy)methyl)-1*H*-1,2,3-triazol-1-yl)hexyl)oxy)-2*H*-chromen-2-one (**39**)

Yellow liquid, 275 mg, starting from 200 mg of coumarin **3d**, 82% yield. IR: 3134, 3048, 2947, 2865, 1729, 1610, 1556, 1508, 1456, 1400, 1394, 1279, 1229, 1199, 1120, 1047, 1021, 833 cm^–1^; ^1^H NMR (CDCl_3_, 300 MHz): δ/ppm 0.67 (3H, d, *J* = 6.7 Hz), 0.82–0.98 (8H, m), 1.20–1.25 (2H, m), 1.26–1.67 (7H, m), 1.77–1.86 (2H, m), 1.90–1.98 (2H, m), 2.14–2.22 (2H, m), 3.20 (1H, td, *J* = 10.5, 4.1 Hz), 4.00 (2H, t, *J* = 6.4 Hz), 4.37 (2H, t, *J* = 7.0 Hz), 4.57 (1H, d, *J* = 12.3 Hz), 4.78 (1H, d, *J* = 12.3 Hz), 6.24 (1H, d, *J* = 9.6 Hz), 6.78–6.84 (2H, m), 7.37 (1H, d, *J* = 8.4 Hz), 7.53 (1H, s), 7.64 (1H, d, *J* = 9.3 Hz); ^13^C NMR (CDCl_3_, 75 MHz): δ/ppm 16.3, 21.2, 22.5, 23.4, 25.7, 25.8, 26.4, 28.9, 30.4, 31.6, 34.7, 40.4, 48.3, 50.0, 62.2, 68.4, 78.9, 101.5, 112.6, 113.1, 113.1, 122.3, 128.9, 143.7, 146.4, 156.0, 161.5, 162.4. HRMS (ESI) *m*/*z*: calcd for C_28_H_39_N_3_O_4_ [M + Na]^+^, 504.2834; found, 504.2812.

##### 4.2.3.29 7-((7-(4-(((2-Isopropyl-5-methylcyclohexyl)oxy)methyl)-1*H*-1,2,3-triazol-1-yl)heptyl)oxy)-2*H*-chromen-2-one (**40**)

Greenish liquid, 302 mg, starting from 200 mg of coumarin **3e**, 92% yield. IR: 3132, 3040, 2923, 2864, 1729, 1610, 1556, 1508, 1456, 1393, 1349, 1279, 1229, 1199, 1120, 1048, 833 cm^–1^; ^1^H NMR (CDCl_3_, 300 MHz): δ/ppm 0.67 (3H, d, *J* = 6.7 Hz), 0.82–0.98 (8H, m), 1.20–1.60 (8H, m), 1.61–1.67 (2H, m), 1.76–1.85 (3H, m), 1.87–1.94 (2H, m), 2.14–2.22 (2H, m), 3.20 (1H, td, *J* = 10.5, 4.3 Hz), 3.99 (2H, t, *J* = 6.4 Hz), 4.35 (2H, t, *J* = 7.3 Hz), 4.56 (1H, d, *J* = 12.0 Hz),4.78 (1H, d, *J* = 12.3 Hz), 6.25 (1H, d, *J* = 9.3 Hz), 6.78–6.84 (2H, m), 7.36 (1H, d, *J* = 8.4 Hz), 7.51 (1H, s), 7.63 (1H, d, *J* = 9.6 Hz); ^13^C NMR (CDCl_3_, 75 MHz): δ/ppm 16.3, 21.2, 22.5, 23.4, 25.8, 26.0, 26.6, 28.9, 29.0, 30.4, 31.7, 34.7, 40.4, 48.4, 50.4, 62.2, 68.6, 78.9, 101.5, 112.6, 113.1, 113.1, 122.3, 128.9, 143.7, 146.4, 156.1, 161.5, 162.5. HRMS (ESI) *m*/*z*: calcd for C_29_H_41_N_3_O_4_ [M + Na]^+^, 518.2995; found, 518.2972.

##### 4.2.3.30 7-((9-(4-(((2-Isopropyl-5-methylcyclohexyl)oxy)methyl)-1*H*-1,2,3-triazol-1-yl)nonyl)oxy)-2*H*-chromen-2-one (**41**)

Yellow liquid, 197 mg, starting from 200 mg of coumarin **3f**, 62% yield. IR: 3140, 3070, 2923, 2856, 1730, 1611, 1556, 1508, 1457, 1393, 1349, 1279, 1230, 1199, 1120, 1047, 1020, 833 cm^–1^; ^1^H NMR (CDCl_3_, 300 MHz): δ/ppm 0.67 (3H, d, *J* = 7.0 Hz), 0.81–0.98 (8H, m), 1.20–1.46 (12H, m), 1.58–1.67 (2H, m), 1.75–1.92 (5H, m), 2.14–2.22 (2H, m), 3.20 (1H, td, *J* = 10.5, 4.1 Hz), 4.00 (2H, t, *J* = 6.4 Hz), 4.33 (2H, t, *J* = 7.0 Hz), 4.57 (1H, d, *J* = 12.3 Hz), 4.77 (1H, d, *J* = 12.3 Hz), 6.24 (1H, d, *J* = 9.3 Hz), 6.79–6.85 (2H, m), 7.36 (1H, d, *J* = 8.4 Hz), 7.51 (1H, s), 7.63 (1H, d, *J* = 9.3 Hz); ^13^C NMR (CDCl_3_, 75 MHz): δ/ppm 16.3, 21.2, 22.5, 23.4, 25.8, 26.1, 26.6, 29.1, 29.4, 29.5, 30.5, 31.7, 34.7, 40.4, 48.4, 50.5, 62.2, 68.7, 78.9, 101.5, 112.5, 113.1, 113.2, 122.3, 128.9, 143.7, 146.3, 156.1, 161.5, 162.6. HRMS (ESI) *m*/*z*: calcd for C_31_H_45_N_3_O_4_ [M + Na]^+^, 546.3308; found, 546.3282.

##### 4.2.3.31 7-((10-(4-(((2-Isopropyl-5-methylcyclohexyl)oxy)methyl)-1*H*-1,2,3-triazol-1-yl)decyl) oxy)-2*H*-chromen-2-one (**42**)

White powder, 263 mg, starting from 200 mg of coumarin **3g**, 84% yield, mp 58–59 °C; IR: 3147, 3040, 2916, 2850, 1725, 1614, 1553, 1510, 1469, 1402, 1350, 1294, 1236, 1192, 1133, 1090, 1052, 1038, 835 cm^–1^; ^1^H NMR (CDCl_3_, 300 MHz): δ/ppm 0.66 (3H, d, *J* = 7.0 Hz), 0.79–0.98 (8H, m), 1.20–1.45 (14H, m), 1.58–1.67 (2H, m), 1.76–1.92 (5H, m), 2.14–2.21 (2H, m), 3.20 (1H, td, *J* = 10.5, 4.1 Hz), 4.00 (2H, t, *J* = 6.4 Hz), 4.33 (2H, t, *J* = 7.0 Hz), 4.57 (1H, d, *J* = 12.3 Hz), 4.77 (1H, d, *J* = 12.3 Hz), 6.23 (1H, d, *J* = 9.3 Hz), 6.79–6.85 (2H, m), 7.36 (1H, d, *J* = 8.4 Hz), 7.51 (1H, s), 7.64 (1H, d, *J* = 9.3 Hz); ^13^C NMR (CDCl_3_, 75 MHz): δ/ppm 16.3, 21.2, 22.5, 23.4, 25.7, 26.1, 26.7, 29.1, 29.2, 29.4, 29.5, 29.6, 30.5, 31.6, 34.7, 40.4, 48.3, 50.5, 62.2, 68.8, 78.9, 101.5, 112.5, 113.1, 113.2, 122.3, 128.9, 143.7, 146.3, 156.1, 161.5, 162.6. HRMS (ESI) *m*/*z*: calcd for C_32_H_47_N_3_O_4_ [M + Na]^+^, 560.3464; found, 560.3445.

##### 4.2.3.32 7-((12-(4-(((2-Isopropyl-5-methylcyclohexyl)oxy)methyl)-1*H*-1,2,3-triazol-1-yl)dodecyl) oxy)-2*H*-chromen-2-one (**43**)

White powder, 268 mg, starting from 200 mg of coumarin **3h**, 88% yield, mp 59–60 °C; IR: 3147, 3040, 2917, 2849, 1726, 1617, 1554, 1510, 1468, 1403, 1350, 1296, 1193, 1134, 1100, 1050, 1005, 832 cm^–1^; ^1^H NMR (CDCl_3_, 300 MHz): δ/ppm 0.66 (3H, d, *J* = 7.0 Hz), 0.81–0.98 (8H, m), 1.20–1.46 (17H, m), 1.58–1.67 (3H, m), 1.76–1.91 (5H, m), 2.15–2.21 (2H, m), 3.20 (1H, td, *J* = 10.5, 4.1 Hz), 4.00 (2H, t, *J* = 6.4 Hz), 4.33 (2H, t, *J* = 7.3 Hz), 4.57 (1H, d, *J* = 12.3 Hz), 4.77 (1H, d, *J* = 12.3 Hz), 6.23 (1H, d, *J* = 9.3 Hz), 6.79–6.85 (2H, m), 7.36 (1H, d, *J* = 8.4 Hz), 7.51 (1H, s), 7.63 (1H, d, *J* = 9.3 Hz); ^13^C NMR (CDCl_3_, 75 MHz): δ/ppm 16.3, 21.2, 22.5, 23.4, 25.7, 26.1, 26.7, 26.9, 29.1, 29.2, 29.3, 29.5, 29.6, 29.7, 30.5, 31.6, 34.7, 40.4, 48.4, 50.5, 62.2, 68.8, 78.9, 101.5, 112.5, 113.0, 113.2, 122.3, 128.9, 143.7, 146.3, 156.1, 161.5, 162.6. HRMS (ESI) *m*/*z*: calcd for C_34_H_51_N_3_O_4_ [M + Na]^+^, 588.3777; found, 588.3752.

#### 4.2.4 Synthesis of Coumarin-Monoterpene Derivatives Bearing Alkyl Chain (**44–71**)

For compounds **44–67**, the corresponding coumarin alkyl bromides (**2a–2h**; 1 mmol) and monoterpene derivatives (**4–7**; 1 mmol) were dissolved in dry DMF (5 mL). K_2_CO_3_ (2 mmol) and KI (0.1 mmol) were added to this solution, and the mixture was stirred at 60 °C for 18 h. It was poured into ice containing 10% HCl. The aqueous mixture was extracted with CH_2_Cl_2_ (3 × 50 mL). The organic phase was dried with sodium sulfate, and the solvent was removed under a vacuum. The compounds **44–67** were purified by column chromatography using hexane: ethyl acetate in a ratio of 4:2.^23^

For compounds **68–71**, 1 mmol of coumarin alkyl bromide derivatives, 1 mmol menthol, 0.1 mmol NaI, and 1.5 mmol *N*,*N*-diisopropylethylamine (DIPEA) were mixed and stirred at 150 °C for 2 h. It was cooled and 10 mL of 10% sodium bisulfate was added to this mixture. It was extracted with ethyl acetate (3 × 50 mL). The organic phase was dried with sodium sulfate, and the solvent was removed under a vacuum. The compounds **68–71** were purified by column chromatography using hexane/ethyl acetate in a ratio of 10:1.^21^

##### 4.2.4.1 7-(2-(2-Isopropyl-5-methylphenoxy)ethoxy)-2*H*-chromen-2-one (**44**)

White powder, 256 mg, starting from 300 mg of coumarin **2a**, 68% yield, mp 181–182 °C; IR: 3040, 2963, 1732, 1614, 1405, 1289, 1232, 1134, 1097, 832, 753 cm^–1^; ^1^H NMR (CDCl_3_, 300 MHz): δ/ppm 1.17 (6H, d, *J* = 6.7 Hz), 2.33 (3H, s), 3.20–3.27 (1H, m), 4.33–4.35 (2H, m), 4.36–4.61 (2H, m), 6.25–6.30 (1H, m), 6.70 (1H, s), 6.78 (1H, d, *J* = 7.6 Hz), 6.88–6.92 (2H, m), 7.10 (1H, d, *J* = 7.6 Hz), 7.37–7.42 (1H, m), 7.64 (1H, d, *J* = 9.3 Hz); ^13^C NMR (CDCl_3_, 75 MHz): δ/ppm 21.5, 22.9, 26.8, 66.7, 67.4, 101.8, 112.8, 113.2, 113.5, 122.0, 126.3, 129.0, 129.1, 134.6, 136.6, 143.6, 155.7, 156.0, 161.4, 162.1. HRMS (ESI) *m*/*z*: calcd for C_21_H_22_O_4_ [M + Na]^+^, 361.1416; found, 361.1394.

##### 4.2.4.2 7-(3-(2-Isopropyl-5-methylphenoxy)propoxy)-2*H*-chromen-2-one (**45**)

White liquid, 99 mg, starting from 200 mg of coumarin **2b**, 40% yield. IR: 3038, 2958, 1725, 1609, 1505, 1401, 1255, 1119, 831, 615 cm^–1^; ^1^H NMR (CDCl_3_, 300 MHz): δ/ppm 1.18 (6H, d, *J* = 7.0 Hz), 1.21–1.25 (2H, m), 2.31 (3H, s), 3.24–3.29 (1H, m), 4.16 (2H, t, *J* = 5.8 Hz), 4.25 (2H, t, *J* = 6.1 Hz), 6.24(1H, d, *J* = 9.3 Hz), 6.69 (1H, s), 6.75 (1H, d, *J* = 8.2 Hz), 6.83–6.87 (2H, m), 7.08 (1H, d, *J* = 7.6 Hz), 7.36 (1H, d, *J* = 8.7 Hz), 7.63 (1H, d, *J* = 9.6 Hz); ^13^C NMR (CDCl_3_, 75 MHz): δ/ppm 21.6, 23.0, 26.8, 29.4, 64.0, 65.4, 69.5, 101.5, 112.3, 112.7, 113.1, 113.3, 121.5, 126.1, 129.0, 134.1, 136.6, 143.7, 155.9, 156.0, 161.5, 162.3. HRMS (ESI) *m*/*z*: calcd for C_22_H_24_O_4_ [M + Na]^+^, 375.1572; found, 375.1551.

##### 4.2.4.3 7-(4-(2-Isopropyl-5-methylphenoxy)butoxy)-2*H*-chromen-2-one (**46**)

White liquid, 128 mg, starting from 200 mg of coumarin **2c**, 52% yield. IR: 3040, 2957, 1728, 1609, 1505, 1402, 1229, 1194, 994, 831, 730 cm^–1^; ^1^H NMR (CDCl_3_, 300 MHz): δ/ppm 1.22 (6H, d, *J* = 7.0 Hz), 2.04 (4H, s, br), 2.33 (3H, s), 3.29–3.33 (1H, m), 4.04 (2H, t, *J* = 5.5 Hz), 4.10 (2H, t, *J* = 5.8 Hz), 6.23 (1H, d, *J* = 9.3 Hz), 6.68 (1H, s), 6.74–6.86 (3H, m), 7.10 (1H, d, *J* = 7.6 Hz), 7.35 (1H, d, *J* = 8.4 Hz), 7.60 (1H, d, *J* = 9.6 Hz); ^13^C NMR (CDCl_3_, 75 MHz): δ/ppm 21.6, 23.0, 26.3 × 2, 26.8, 67.4, 68.4, 101.5, 112.3, 112.7, 113.1, 113.1, 121.3, 126.1, 129.1, 134.1, 136.5, 143.7, 156.1, 156.1, 161.4, 162.5. HRMS (ESI) *m*/*z*: calcd for C_23_H_26_O_4_ [M + Na]^+^, 389.1729; found, 389.1707.

##### 4.2.4.4 7-((6-(2-Isopropyl-5-methylphenoxy)hexyl)oxy)-2*H*-chromen-2-one (**47**)

Yellow liquid, 140 mg, starting from 200 mg of coumarin **2d**, 58% yield. IR: 3040, 2939, 1730, 1609, 1505, 1402, 1255, 1119, 1093, 831, 615 cm^–1^; ^1^H NMR (CDCl_3_, 300 MHz): δ/ppm 1.24 (6H, d, *J* = 7.0 Hz), 1.59–1.61 (4H, m), 1.83–1.89 (4H, m), 2.34 (3H, s), 3.30–3.34 (1H, m), 3.96–4.04 (4H, m), 6.23 (1H, d, *J* = 9.3 Hz), 6.68 (1H, s), 6.73–6.85 (3H, m), 7.11 (1H, d, *J* = 7.6 Hz), 7.35 (1H, d, *J* = 8.4 Hz), 7.60 (1H, d, *J* = 9.3 Hz); ^13^C NMR (CDCl_3_, 75 MHz): δ/ppm 21.6, 23.0, 26.0, 26.3, 26.9, 29.2, 29.6, 67.7, 68.7, 101.5, 112.3, 112.6, 113.0, 113.1, 121.2, 126.0, 129.0, 134.1, 136.4, 143.7, 156.1, 156.3, 161.4, 162.6. HRMS (ESI) *m*/*z*: calcd for C_25_H_30_O_4_ [M + Na]^+^, 417.2042; found, 417.2019.

##### 4.2.4.5 7-((7-(2-Isopropyl-5-methylphenoxy)heptyl)oxy)-2*H*-chromen-2-one (**48**)

White liquid, 150 mg, starting from 200 mg of coumarin **2e**, 62% yield. IR: 3038, 2934, 1731, 1610, 1505, 1392, 1256, 1119, 831, 615 cm^–1^; ^1^H NMR (CDCl_3_, 300 MHz): δ/ppm 1.20 (6H, d, *J* = 6.7 Hz), 1.41–1.56 (6H, m), 1.77–1.82 (4H, m), 2.31 (3H, s), 3.23–3.30 (1H, m), 3.92–4.03 (4H, m), 6.23 (1H, d, *J* = 9.3 Hz), 6.65 (1H, s), 6.72 (1H, d, *J* = 7.6 Hz), 6.78–6.84 (2H, m), 7.07 (1H, d, *J* = 7.6 Hz), 7.35 (1H, d, *J* = 8.7 Hz), 7.62 (1H, d, *J* = 9.3 Hz); ^13^C NMR (CDCl_3_, 75 MHz): δ/ppm 21.6, 23.0, 26.2, 26.4, 26.8, 29.1, 29.3, 29.6, 67.8, 68.8, 101.5, 112.3, 112.6, 113.1, 113.2, 121.1, 126.0, 128.9, 134.2, 136.4, 143.7, 156.1, 156.3, 161.5, 162.6. HRMS (ESI) *m*/*z*: calcd for C_26_H_32_O_4_ [M + Na]^+^, 431.2198; found, 431.2176.

##### 4.2.4.6 7-((9-(2-Isopropyl-5-methylphenoxy)nonyl)oxy)-2*H*-chromen-2-one (**49**)

White liquid, 120 mg, starting from 200 mg of coumarin **2f**, 48% yield. IR: 3040, 2926, 1731, 1610, 1505, 1349, 1256, 1119, 832, 615 cm^–1^; ^1^H NMR (CDCl_3_, 300 MHz): δ/ppm 1.20 (6H, d, *J* = 7.0 Hz), 1.23–1.48 (10H, m), 1.75–1.83 (4H, m), 2.31 (3H, s), 3.24–3.31 (1H, m), 3.91–4.01 (4H, m), 6.23 (1H, d, *J* = 9.3 Hz), 6.65 (1H, s), 6.72 (1H, d, *J* = 7.0 Hz), 6.78–6.84 (2H, m), 7.08 (1H, d, *J* = 7.6 Hz), 7.33 (1H, d, *J* = 8.7 Hz), 7.60 (1H, d, *J* = 9.3 Hz); ^13^C NMR (CDCl_3_, 75 MHz): δ/ppm 21.6, 23.0, 26.2, 26.4, 26.8, 29.2, 29.5, 29.6, 29.7, 67.9, 68.8, 101.5, 112.3, 112.6, 113.0, 113.2, 121.0, 126.0, 129.0, 134.2, 136.4, 143.8, 156.1, 156.4, 161.7, 162.6. HRMS (ESI) *m*/*z*: calcd for C_28_H_36_O_4_ [M + Na]^+^, 459.2511; found, 459.2487.

##### 4.2.4.7 7-((10-(2-Isopropyl-5-methylphenoxy)decyl)oxy)-2*H*-chromen-2-one (**50**)

White liquid, 122 mg, starting from 200 mg of coumarin **2g**, 52% yield. IR: 3038, 2924, 1731, 1610, 1506, 1280, 1230, 1120, 1017, 831, 753 cm^–1^; ^1^H NMR (CDCl_3_, 300 MHz): δ/ppm 1.11 (6H, d, *J* = 7.0 Hz), 1.25–1.40 (12H, m), 1.66–1.77 (4H, m), 2.22 (3H, s), 3.15–3.24 (1H, m), 3.83–3.94 (4H, m), 6.13 (1H, d, *J* = 9.3 Hz), 6.56 (1H, s), 6.63 (1H, d, *J* = 7.6 Hz), 6.70–6.75 (2H, m), 6.99 (1H, d, *J* = 7.6 Hz), 7.25 (1H, d, *J* = 8.4 Hz), 7.53 (1H, d, *J* = 9.3 Hz); ^13^C NMR (CDCl_3_, 75 MHz): δ/ppm 21.6, 23.0, 26.2, 26.4, 26.8, 29.2, 29.5, 29.6, 29.7, 68.0, 68.8, 101.5, 112.4, 112.6, 113.1, 113.2, 121.0, 126.0, 129.0, 134.2, 136.4, 143.8, 156.1, 156.4, 161.5, 162.6. HRMS (ESI) *m*/*z*: calcd for C_29_H_38_O_4_ [M + Na]^+^, 473.2668; found, 473.2645.

##### 4.2.4.8 7-((12-(2-Isopropyl-5-methylphenoxy)dodecyl)oxy)-2*H*-chromen-2-one (**51**)

White liquid, 130 mg, starting from 200 mg of coumarin **2h**, 56% yield. IR: 3038, 2923, 1732, 1611, 1506, 1279, 1119, 1094, 832, 753 cm^–1^; ^1^H NMR (CDCl_3_, 300 MHz): δ/ppm 1.12 (6H, d, *J* = 6.9 Hz), 1.18–1.34 (16H, m), 1.66–1.78 (4H, m), 2.23 (3H, s), 3.16–3.23 (1H, m), 3.84–3.95 (4H, m), 6.15 (1H, d, *J* = 9.3 Hz), 6.57 (1H, s), 6.63 (1H, d, *J* = 7.6 Hz), 6.72–6.77 (2H, m), 7.00 (1H, d, *J* = 7.6 Hz), 7.27 (1H, d, *J* = 8.4 Hz), 7.55 (1H, d, *J* = 9.3 Hz); ^13^C NMR (CDCl_3_, 75 MHz): δ/ppm 21.5, 22.9, 26.1, 26.4, 26.8, 29.2, 29.5, 29.6, 29.7 × 3, 68.0, 68.8, 101.5, 112.3, 112.5, 113.1, 113.2, 121.0, 125.9, 128.9, 134.2, 136.4, 143.6, 156.1, 156.4, 161.5, 162.6. HRMS (ESI) *m*/*z*: calcd for C_31_H_42_O_4_ [M + Na]^+^, 501.2981; found, 501.2957.

##### 4.2.4.9 7-(2-(5-Isopropyl-2-methylphenoxy)ethoxy)-2*H*-chromen-2-one (**52**)

White powder, 180 mg, starting from 200 mg of coumarin **2a**, 72% yield, mp 172–173 °C; IR: 3050, 2955, 1726, 1608, 1508, 1396, 1228, 1109, 1058, 818, 614 cm^–1^; ^1^H NMR (DMSO-*d*_6_, 300 MHz): δ/ppm 1.15 (6H, d, *J* = 7.0 Hz), 2.00 (3H, s), 2.77–2.81 (1H, m), 4.29–4.32 (2H, m), 4.41–4.43 (2H, m), 6.26 (1H, d, *J* = 9.3 Hz), 6.68 (1H, d, *J* = 7.6 Hz), 6.80 (1H, s), 6.95–7.00 (3H, m), 7.60 (1H, dd, *J* = 8.4, 2.0 Hz), 7.96 (1H, d, *J* = 9.6 Hz); ^13^C NMR (DMSO-*d*_6_, 75 MHz): δ/ppm 16.2, 24.6, 34.0, 67.1, 67.9, 102.0, 110.7, 113.1, 113.2, 113.5, 118.8, 123.8, 130.2, 130.9, 145.0, 148.2, 156.0, 156.9, 161.0, 162.3. HRMS (ESI) *m*/*z*: calcd for C_21_H_22_O_4_ [M + Na]^+^, 361.1416; found, 361.1393.

##### 4.2.4.10 7-(3-(5-Isopropyl-2-methylphenoxy)propoxy)-2*H*-chromen-2-one (**53**)

White liquid, 150 mg, starting from 200 mg of coumarin **2b**, 60% yield. IR: 3052, 2958, 1725, 1609, 1508, 1400, 1229, 1120, 995, 831, 751 cm^–1^; ^1^H NMR (CDCl_3_, 300 MHz): δ/ppm 1.15 (6H, d, *J* = 7.0 Hz), 2.10 (3H, s), 2.21–2.25 (2H, m), 2.75–2.79 (1H, m), 4.09 (2H, t, *J* = 5.8 Hz), 4.16 (2H, t, *J* = 6.1 Hz), 6.15 (1H, d, *J* = 9.3 Hz), 6.63–6.67 (2H, m), 6.74–6.78 (2H, m), 6.97 (1H, d, *J* = 7.3 Hz), 7.27 (1H, d, *J* = 8.2 Hz), 7.55 (1H, d, *J* = 9.3 Hz); ^13^C NMR (CDCl_3_, 75 MHz): δ/ppm 14.8, 23.1, 28.1, 33.0, 62.7, 64.1, 100.3, 108.2, 111.4, 111.7, 111.9, 117.1, 122.9, 127.7, 129.4, 142.4, 146.9, 154.8, 155.6, 160.2, 161.0. HRMS (ESI) *m*/*z*: calcd for C_22_H_24_O_4_ [M + Na]^+^, 375.1572; found, 375.1550.

##### 4.2.4.11 7-(4-(5-Isopropyl-2-methylphenoxy)butoxy)-2*H*-chromen-2-one (**54**)

White powder, 216 mg, starting from 200 mg of coumarin **2c**, 88% yield, mp 74–75 °C; IR: 3040, 2957, 1724, 1608, 1556, 1396, 1230, 1124, 1027, 1000, 836, 615 cm^–1^; ^1^H NMR (CDCl_3_, 300 MHz): δ/ppm 1.24 (6H, d, *J* = 7.0 Hz), 1.97–2.07 (4H, m), 2.18 (3H, s), 2.83–2.88 (1H, m), 4.05 (2H, t, *J* = 5.5 Hz), 4.11 (2H, t, *J* = 5.8 Hz), 6.24 (1H, d, *J* = 9.0 Hz), 6.69 (1H, s), 6.73 (1H, d, *J* = 8.7 Hz), 6.81–6.85 (2H, m), 7.03 (1H, d, *J* = 7.3 Hz), 7.36 (1H, d, *J* = 8.2 Hz), 7.63 (1H, d, *J* = 9.3 Hz); ^13^C NMR (CDCl_3_, 75 MHz): δ/ppm 16.1, 24.4, 26.2, 34.3, 67.3, 68.4, 101.5, 109.5, 112.6, 113.1, 113.2, 118.2, 124.2, 128.9, 130.6, 143.7, 148.1, 156.1, 157.1, 161.5, 162.5. HRMS (ESI) *m*/*z*: calcd for C_23_H_26_O_4_ [M + Na]^+^, 389.1729; found, 389.1708.

##### 4.2.4.12 7-((6-(5-Isopropyl-2-methylphenoxy)hexyl)oxy)-2*H*-chromen-2-one (**55**)

White liquid, 150 mg, starting from 200 mg of coumarin **2d**, 62% yield. IR: 3040, 2944, 1721, 1610, 1509, 1391, 1289, 1123, 1014, 833, 634 cm^–1^; ^1^H NMR (CDCl_3_, 300 MHz): δ/ppm 1.16 (6H, d, *J* = 7.0 Hz), 1.48–1.51 (4H, m), 1.75–1.78 (4H, m), 2.10 (3H, s), 2.73–2.80 (1H, m), 3.89–3.96 (4H, m), 6.16 (1H, d, *J* = 9.3 Hz), 6.61 (1H, s), 6.64 (1H, d, *J* = 7.6 Hz), 6.72–6.77 (2H, m), 6.97 (1H, d, *J* = 7.3 Hz), 7.27 (1H, d, *J* = 8.4 Hz), 7.56 (1H, d, *J* = 9.3 Hz); ^13^C NMR (CDCl_3_, 75 MHz): δ/ppm 16.1, 24.4, 26.0, 26.2, 29.2, 29.5, 34.4, 67.7, 68.7, 101.4, 109.5, 112.6, 113.1, 113.2, 118.0, 124.2, 128.9, 130.6, 143.8, 148.1, 156.1, 157.2, 161.6, 162.6. HRMS (ESI) *m*/*z*: calcd for C_25_H_30_O_4_ [M + Na]^+^, 417.2042; found, 417.2020.

##### 4.2.4.13 7-((7-(5-Isopropyl-2-methylphenoxy)heptyl)oxy)-2*H*-chromen-2-one (**56**)

White liquid, 207 mg, starting from 200 mg of coumarin **2e**, 86% yield. IR: 3038, 2933, 1731, 1610, 1508, 1279, 1229, 1110, 1018, 831, 615 cm^–1^; ^1^H NMR (CDCl_3_, 300 MHz): δ/ppm 1.23 (6H, d, *J* = 7.0 Hz), 1.43–1.56 (6H, m), 1.79–1.84 (4H, m), 2.18 (3H, s), 2.80–2.87 (1H, m), 3.94–4.02 (4H, m), 6.22 (1H, d, *J* = 9.3 Hz), 6.68–6.72 (2H, m), 6.77–6.83 (2H, m), 7.03 (1H, d, *J* = 7.6 Hz), 7.33 (1H, d, *J* = 8.7 Hz), 7.60 (1H, d, *J* = 9.3 Hz); ^13^C NMR (CDCl_3_, 75 MHz): δ/ppm 16.1, 24.4, 26.2, 26.3, 29.1, 29.3, 29.6, 34.4, 67.9, 68.8, 101.5, 109.6, 112.6, 113.1, 113.2, 118.0, 124.2, 129.0, 130.6, 143.7, 148.0, 156.1, 157.3, 161.5, 162.6. HRMS (ESI) *m*/*z*: calcd for C_26_H_32_O_4_ [M + Na]^+^, 431.2198; found, 431.2177.

##### 4.2.4.14 7-((9-(5-Isopropyl-2-methylphenoxy)nonyl)oxy)-2*H*-chromen-2-one (**57**)

White liquid, 147 mg, starting from 200 mg of coumarin **2f**, 62% yield. IR: 3040, 2926, 1732, 1610, 1509, 1279, 1229, 1120, 995, 832, 615 cm^–1^; ^1^H NMR (CDCl_3_, 300 MHz): δ/ppm 1.23 (6H, d, *J* = 6.7 Hz), 1.37–1.49 (10H, m), 1.75–1.83 (4H, m), 2.18 (3H, s), 2.80–2.87 (1H, m), 3.93–4.02 (4H, m), 6.23 (1H, d, *J* = 9.3 Hz), 6.67–6.70 (2H, m), 6.72–6.84 (2H, m), 7.04 (1H, d, *J* = 7.3 Hz), 7.34 (1H, d, *J* = 8.4 Hz), 7.62 (1H, d, *J* = 9.3 Hz); ^13^C NMR (CDCl_3_, 75 MHz): δ/ppm 16.1, 24.4, 26.2, 26.4, 29.2, 29.5 × 2, 29.6, 29.7, 34.3, 68.0, 68.8, 101.5, 109.6, 112.5, 113.1, 113.2, 117.9, 124.3, 128.9, 130.5, 143.7, 148.0, 156.1, 157.3, 161.6, 162.6. HRMS (ESI) *m*/*z*: calcd for C_28_H_36_O_4_ [M + Na]^+^, 459.2511; found, 459.2489.

##### 4.2.4.15 7-((10-(5-Isopropyl-2-methylphenoxy)decyl)oxy)-2*H*-chromen-2-one (**58**)

White powder, 203 mg, starting from 200 mg of coumarin **2g**, 86% yield, mp 50–51 °C; IR: 3052, 2955, 1731, 1619, 1556, 1293, 1131, 1014, 839, 723 cm^–1^; ^1^H NMR (CDCl_3_, 300 MHz): δ/ppm 1.22 (6H, d, *J* = 7.0 Hz), 1.29–1.62 (12H, m), 1.74–1.85 (4H, m), 2.18 (3H, s), 2.80–2.90 (1H, m), 3.93–4.02 (4H, m), 6.23 (1H, d, *J* = 9.3 Hz), 6.68 (1H, s), 6.70 (1H, d, *J* = 7.9 Hz), 6.80–6.84 (2H, m), 7.04 (1H, d, *J* = 7.6 Hz), 7.34 (1H, d, *J* = 8.4 Hz), 7.62 (1H, d, *J* = 9.3 Hz); ^13^C NMR (CDCl_3_, 75 MHz): δ/ppm 16.0, 24.3, 26.1, 26.3, 29.1, 29.5 × 2, 29.6, 29.7, 34.3, 68.0, 68.8, 101.5, 109.6, 112.5, 113.1, 113.2, 117.9, 124.3, 128.9, 130.5, 143.6, 148.0, 156.1, 157.3, 161.5, 162.6. HRMS (ESI) *m*/*z*: calcd for C_29_H_38_O_4_ [M + Na]^+^, 473.2668; found, 473.2645.

##### 4.2.4.16 7-((12-(5-Isopropyl-2-methylphenoxy)dodecyl)oxy)-2*H*-chromen-2-one (**59**)

White powder, 186 mg, starting from 200 mg of coumarin **2h**, 80% yield, mp 58–59 °C; IR: 3040, 2917, 1727, 1616, 1510, 1397, 1237, 1120, 1025, 835, 720 cm^–1^; ^1^H NMR (CDCl_3_, 300 MHz): δ/ppm 1.23 (6H, d, *J* = 7.0 Hz), 1.30–1.62 (16H, m), 1.74–1.86 (4H, m), 2.18 (3H, s), 2.83–2.88 (1H, m), 3.93–4.03 (4H, m), 6.24 (1H, d, *J* = 9.3 Hz), 6.68 (1H, s), 6.71 (1H, d, *J* = 7.6 Hz), 6.80–6.85 (2H, m), 7.04 (1H, d, *J* = 7.6 Hz), 7.36 (1H, d, *J* = 8.4 Hz), 7.63 (1H, d, *J* = 9.6 Hz); ^13^C NMR (CDCl_3_, 75 MHz): δ/ppm 16.1, 24.4, 26.1, 26.4, 29.2, 29.5, 29.6, 29.8, 34.3, 68.0, 68.8, 101.5, 109.6, 112.5, 113.1, 113.2, 117.9, 124.3, 128.9, 130.5, 143.7, 148.0, 156.1, 157.3, 161.6, 162.6. HRMS (ESI) *m*/*z*: calcd for C_31_H_42_O_4_ [M + Na]^+^, 501.2981; found, 501.2955.

##### 4.2.4.17 7-(2-(4-Allyl-2-methoxyphenoxy)ethoxy)-2*H*-chromen-2-one (**60**)

White powder, 280 mg, starting from 300 mg of coumarin **2a**, 72% yield, mp 86–87 °C; IR: 3071, 2930, 1729, 1610, 1508, 1402, 1350, 1207, 1126, 1059, 914, 841, 751 cm^–1^; ^1^H NMR (CDCl_3_, 300 MHz): δ/ppm 3.34 (2H, d, *J* = 6.4 Hz), 3.85 (3H, s), 4.39 (4H, s), 5.05–5.11 (2H, m), 5.91–6.00 (1H, m), 6.25 (1H, d, *J* = 9.0 Hz), 6.70–6.74 (2H, m), 6.88–6.91 (3H, m), 7.37 (1H, d, *J* = 9.6 Hz), 7.64 (1H, d, *J* = 9.3 Hz); ^13^C NMR (CDCl_3_, 75 MHz): δ/ppm 40.0, 56.0, 67.4, 68.0, 101.9, 112.7, 112.9, 113.3, 113.4, 114.9, 116.0, 120.7, 128.9, 134.3, 137.7, 143.6, 146.3, 149.9, 156.0, 161.4, 162.1. HRMS (ESI) *m*/*z*: calcd for C_21_H_20_O_5_ [M + Na]^+^, 375.1208; found, 375.1188.

##### 4.2.4.18 7-(3-(4-Allyl-2-methoxyphenoxy)propoxy)-2*H*-chromen-2-one (**61**)

White powder, 280 mg, starting from 300 mg of coumarin **2b**, 72% yield, mp 70–71 °C; IR: 3072, 2915, 1718, 1608, 1510, 1398, 1251, 1227, 1116, 1026, 991, 832, 614 cm^–1^; ^1^H NMR (CDCl_3_, 300 MHz): δ/ppm 2.28–2.36 (2H, m), 3.33 (2H, d, *J* = 6.4 Hz), 3.84 (3H, s), 4.18–4.26 (4H, m), 5.04–5.10 (2H, m), 5.90–5.99 (1H, m), 6.24 (1H, d, *J* = 9.3 Hz), 6.69–6.73 (2H, m), 6.83–6.86 (3H, m), 7.36 (1H, d, *J* = 9.3 Hz), 7.63 (1H, d, *J* = 9.6 Hz); ^13^C NMR (CDCl_3_, 75 MHz): δ/ppm 29.2, 40.0, 56.0, 65.3, 65.6, 101.6, 112.5, 112.7, 113.1, 113.2, 113.7, 115.9, 120.6, 128.9, 133.5, 137.8, 143.7, 146.6, 149.6, 156.0, 161.5, 162.4. HRMS (ESI) *m*/*z*: calcd for C_22_H_22_O_5_ [M + Na]^+^, 389.1365; found, 389.1344.

##### 4.2.4.19 7-(4-(4-Allyl-2-methoxyphenoxy)butoxy)-2*H*-chromen-2-one (**62**)

Yellow liquid, 314 mg, starting from 300 mg of coumarin **2c**, 82% yield. IR: 3057, 2938, 1726, 1609, 1508, 1396, 1228, 1120, 1033, 831, 615 cm^–1^; ^1^H NMR (CDCl_3_, 300 MHz): δ/ppm 2.01–2.04 (4H, m), 3.33 (2H, d, *J* = 6.3 Hz), 3.85 (3H, s), 4.06–4.13 (4H, m), 5.03–5.11 (2H, m), 5.89–6.00 (1H, m), 6.24 (1H, d, *J* = 9.4 Hz), 6.69–6.72 (2H, m), 6.80–6.85 (3H, m), 7.36 (1H, d, *J* = 8.1 Hz), 7.64 (1H, d, *J* = 9.5 Hz); ^13^C NMR (CDCl_3_, 75 MHz): δ/ppm 26.0, 26.2, 40.0, 56.1, 68.4, 68.8, 101.5, 112.4, 112.6, 113.1, 113.2, 113.3, 115.8, 120.6, 128.9, 133.2, 137.8, 143.7, 146.8, 149.5, 156.1, 161.5, 162.5. HRMS (ESI) *m*/*z*: calcd for C_23_H_24_O_5_ [M + Na]^+^, 403.1521; found, 403.1500.

##### 4.2.4.20 7-((6-(4-Allyl-2-methoxyphenoxy)hexyl)oxy)-2*H*-chromen-2-one (**63**)

White liquid, 340 mg, starting from 300 mg of coumarin **2d**, 90% yield. IR: 3040, 2937, 1726, 1609, 1508, 1258, 1228, 1119, 994, 832, 615 cm^–1^; ^1^H NMR (CDCl_3_, 300 MHz): δ/ppm 1.52–1.57 (4H, m), 1.84–1.87 (4H, m), 3.33 (2H, d, *J* = 6.7 Hz), 3.85 (3H, s), 3.99–4.03 (4H, m), 5.04–5.11 (2H, m), 5.91–6.00 (1H, m), 6.23 (1H, d, *J* = 9.3 Hz), 6.69–6.81 (2H, m), 6.82–6.85 (3H, m), 7.35 (1H, d, *J* = 8.4 Hz), 7.63 (1H, d, *J* = 9.6 Hz); ^13^C NMR (CDCl_3_, 75 MHz): δ/ppm 26.0, 29.1, 29.3, 40.0, 56.1, 68.6, 69.0, 101.4, 112.2, 112.5, 113.0 × 2, 113.2, 115.8, 120.5, 129.0, 132.8, 137.9, 143.8, 146.9, 149.4, 156.0, 161.6, 162.5. HRMS (ESI) *m*/*z*: calcd for C_25_H_28_O_5_ [M + Na]^+^, 431.1834; found, 431.1816.

##### 4.2.4.21 7-((7-(4-Allyl-2-methoxyphenoxy)heptyl)oxy)-2*H*-chromen-2-one (**64**)

White powder, 322 mg, starting from 300 mg of coumarin **2e**, 86% yield, mp 62–63 °C; IR: 3070, 2934, 1722, 1612, 1510, 1297, 1229, 1133, 1009, 836, 569 cm^–1^; ^1^H NMR (CDCl_3_, 300 MHz): δ/ppm 1.46–1.50 (6H, m), 1.80–1.85 (4H, m), 3.33 (2H, d, *J* = 6.4 Hz), 3.85 (3H, s), 3.97–4.08 (4H, m), 5.04–5.11 (2H, m), 5.91–6.02 (1H, m), 6.23 (1H, d, *J* = 9.3 Hz), 6.69–6.71 (2H, m), 6.80–6.84 (3H, m), 7.35 (1H, d, *J* = 8.4 Hz), 7.63 (1H, d, *J* = 9.3 Hz); ^13^C NMR (CDCl_3_, 75 MHz): δ/ppm 26.1, 29.1, 29.3, 40.0, 56.1, 68.7, 69.2, 101.4, 112.4, 112.5, 113.1, 113.2, 113.3, 115.8, 120.6, 128.9, 132.9, 137.9, 143.7, 147.0, 149.4, 156.1, 161.5, 162.6. HRMS (ESI) *m*/*z*: calcd for C_26_H_30_O_5_ [M + Na]^+^, 445.1991; found, 445.1968.

##### 4.2.4.22 7-((9-(4-Allyl-2-methoxyphenoxy)nonyl)oxy)-2*H*-chromen-2-one (**65**)

White powder, 312 mg, starting from 300 mg of coumarin **2f**, 85% yield, mp 56–57 °C; IR: 3085, 2917, 1723, 1613, 1510, 1296, 1227, 1132, 1019, 835, 731, 569 cm^–1^; ^1^H NMR (CDCl_3_, 300 MHz): δ/ppm 1.36–1.46 (10H, m), 1.70–1.87 (4H, m), 3.32 (2H, d, *J* = 6.7 Hz), 3.85 (3H, s), 3.96–4.02 (4H, m), 5.03–5.11 (2H, m), 5.89–6.00 (1H, m), 6.23 (1H, d, *J* = 9.3 Hz), 6.68–6.71 (2H, m), 6.79–6.86 (3H, m), 7.35 (1H, d, *J* = 8.4 Hz), 7.63 (1H, d, *J* = 9.6 Hz); ^13^C NMR (CDCl_3_, 75 MHz): δ/ppm 26.1, 29.1, 29.4 × 2, 29.5, 29.6, 40.0, 56.1, 68.8, 69.3, 101.5, 112.4, 112.5, 113.1 × 2, 113.2, 115.8, 120.6, 128.9, 132.8, 137.9, 143.7, 147.0, 149.4, 156.1, 161.6, 162.6. HRMS (ESI) *m*/*z*: calcd for C_28_H_34_O_5_ [M + Na]^+^, 473.2304; found, 473.2280.

##### 4.2.4.23 7-((10-(4-Allyl-2-methoxyphenoxy)decyl)oxy)-2*H*-chromen-2-one (**66**)

White liquid, 252 mg, starting from 300 mg of coumarin **2g**, 69% yield. IR: 3058, 2924, 1728, 1610, 1509, 139249, 1229, 1120, 1034, 833, 615 cm^–1^; ^1^H NMR (CDCl_3_, 300 MHz): δ/ppm 1.32–1.78 (10H, m), 1.81–1.88 (6H, m), 3.33 (2H, d, *J* = 6.7 Hz), 3.85 (3H, s), 3.87–4.03 (4H, m), 5.04–5.11 (2H, m), 5.89–6.00 (1H, m), 6.24 (1H, d, *J* = 9.3 Hz), 6.67–6.71 (2H, m), 6.79–6.86 (3H, m), 7.37 (1H, d, *J* = 8.4 Hz), 7.63 (1H, d, *J* = 9.6 Hz); ^13^C NMR (CDCl_3_, 75 MHz): δ/ppm 26.1, 29.1, 29.4, 29.5, 29.6 × 2, 40.0, 56.1, 68.8, 69.2, 101.4, 112.3, 112.5, 113.0, 113.2, 115.8, 120.6, 128.9, 132.8, 137.9, 143.8, 147.0, 149.4, 156.1, 161.6, 162.6. HRMS (ESI) *m*/*z*: calcd for C_29_H_36_O_5_ [M + Na]^+^, 487.2460; found, 487.2434.

##### 4.2.4.24 7-((12-(4-Allyl-2-methoxyphenoxy)dodecyl)oxy)-2*H*-chromen-2-one (**67**)

White liquid, 151 mg, starting from 300 mg of coumarin **2h**, 42% yield. IR: 3072, 2917, 1720, 1624, 1511, 1467, 1393, 1233, 1133, 1028, 836, 720 cm^–1^; ^1^H NMR (CDCl_3_, 300 MHz): δ/ppm 1.21–1.28 (12H, m), 1.41–1.46 (4H, m), 1.76–1.85 (4H, m), 3.33 (2H, d, *J* = 6.7 Hz), 3.85 (3H, s), 3.95–4.02 (4H, m), 5.03–5.11 (2H, m), 5.91–6.00 (1H, m), 6.24 (1H, d, *J* = 9.3 Hz), 6.68–6.71 (2H, m), 6.79–6.85 (3H, m), 7.36 (1H, d, *J* = 8.4 Hz), 7.63 (1H, d, *J* = 9.6 Hz); ^13^C NMR (CDCl_3_, 75 MHz): δ/ppm 26.0, 26.2, 28.7, 29.2, 29.4, 29.5, 29.6, 29.8, 40.0, 56.1, 68.8, 69.2, 101.4, 112.2, 112.5, 112.9, 113.0, 113.2, 115.8, 120.5, 128.9, 132.7, 137.9, 143.8, 146.9, 149.3, 156.0, 161.6, 162.6. HRMS (ESI) *m*/*z*: calcd for C_31_H_40_O_5_ [M + Na]^+^, 515.2773; found, 515.2754.

##### 4.2.4.25 7-(2-((2-Isopropyl-5-methylcyclohexyl)oxy)ethoxy)-2*H*-chromen-2-one (**68**)

White liquid, 40 mg, starting from 200 mg of coumarin **2a**, 16% yield. IR: 3052, 2958, 1725, 1609, 1508, 1400, 1229, 1120, 995, 831, 751 cm^–1^; ^1^H NMR (CDCl_3_, 300 MHz): δ/ppm 0.78 (3H, d, *J* = 7.0 Hz), 0.80–0.96 (5H, m), 1.12–1.28 (4H, m), 1.45–1.62 (4H, m), 1.98–2.21 (2H, m), 3.10 (1H, td, *J* = 10.5, 4.1 Hz), 3.60–3.70 (1H, m), 3.80–3.97 (1H, m), 4.10–4.20 (2H, m), 6.18 (1H, d, *J* = 9.3 Hz), 6.78–6.84 (2H, m), 7.36 (1H, d, *J* = 8.4 Hz), 7.60 (1H, d, *J* = 9.3 Hz); ^13^C NMR (CDCl_3_, 75 MHz): δ/ppm 16.1, 21.0, 22.2, 23.1, 25.8, 31.6, 34.5, 45.0, 64.6, 65.6, 104.2, 113.7, 114.3, 129.0, 143.1, 146.3, 155.5, 159.6, 160.8. HRMS (ESI) *m*/*z*: calcd for C_21_H_28_O_4_ [M + Na]^+^, 367.1885; found, 367.1868.

##### 4.2.4.26 7-(3-((2-Isopropyl-5-methylcyclohexyl)oxy)propoxy)-2*H*-chromen-2-one (**69**)

White liquid, 91 mg, starting from 200 mg of coumarin **2b**, 36% yield. IR: 3072, 2956, 2917, 1720, 1600, 1510, 1460, 1233, 1133, 1025, 840, 720 cm^–1^; ^1^H NMR (CDCl_3_, 300 MHz): δ/ppm 0.81 (3H, d, *J* = 6.7 Hz), 0.83–0.94 (7H, m), 1.09–1.30 (3H, m), 1.50–1.66 (3H, m), 1.95–2.11 (4H, m), 2.97 (1H, td, *J* = 10.5, 4.1 Hz), 3.36–3.43 (1H, m), 3.70–3.77 (1H, m), 4.01–4.10 (2H, m), 6.17 (1H, d, *J* = 9.6 Hz), 6.75–6.79 (2H, m), 7.30 (1H, d, *J* = 8.4 Hz), 7.58 (1H, d, *J* = 9.6 Hz); ^13^C NMR (CDCl_3_, 75 MHz): δ/ppm 16.3, 21.1, 22.5, 23.5, 25.8, 30.0, 31.7, 34.7, 40.6, 48.4, 64.5, 65.7, 79.7, 101.6, 112.6, 113.0, 113.1, 128.9, 143.7, 156.1, 161.5, 162.5. HRMS (ESI) *m*/*z*: calcd for C_22_H_30_O_4_ [M + Na]^+^, 381.2042; found, 381.2021.

##### 4.2.4.27 7-(4-((2-Isopropyl-5-methylcyclohexyl)oxy)butoxy)-2*H*-chromen-2-one (**70**)

White liquid, 80 mg, starting from 200 mg of coumarin **2d**, 32% yield. IR: 3060, 2976, 1718, 1610, 1500, 1209, 1130, 1028, 840, 720 cm^–1^; ^1^H NMR (CDCl_3_, 300 MHz): δ/ppm 0.70 (3H, d, *J* = 7.0 Hz), 0.80–0.92 (8H, m), 1.11–1.28 (3H, m), 1.51–1.69 (4H, m), 1.78–1.88 (2H, m), 2.01–2.16 (2H, m), 2.95 (1H, td, *J* = 10.5, 4.1 Hz), 3.22–3.29 (1H, m), 3.60–3.67 (1H, m), 3.97 (2H, t, *J* = 6.1 Hz), 6.17 (1H, d, *J* = 9.3 Hz), 6.73–6.78 (2H, m), 7.30 (1H, d, *J* = 8.4 Hz), 7.56 (1H, d, *J* = 9.3 Hz); ^13^C NMR (CDCl_3_, 75 MHz): δ/ppm 16.4, 21.2, 22.6, 23.5, 25.8, 26.2, 27.0, 31.7, 34.8, 40.6, 48.5, 68.1, 68.6, 79.5, 101.5, 112.6, 113.1, 113.2, 128.9, 143.7, 156.1, 161.6, 162.5. HRMS (ESI) *m*/*z*: calcd for C_23_H_32_O_4_ [M + H]^+^, 372.2301; found, 373.2359.

##### 4.2.4.28 7-((6-((2-Isopropyl-5-methylcyclohexyl)oxy)hexyl)oxy)-2*H*-chromen-2-one (**71**)

White liquid, 49 mg, starting from 200 mg of coumarin **2e**, 20% yield. IR: 3072, 2950, 1700, 1610, 1500, 1450, 1230, 1109, 1020, 832, 720 cm^–1^; ^1^H NMR (CDCl_3_, 300 MHz): δ/ppm 0.69 (3H, d, *J* = 7.0 Hz), 0.73–0.91 (10H, m), 1.09–1.45 (5H, m), 1.47–1.57 (4H, m), 1.60–1.99 (2H, m), 2.00–2.17 (2H, m), 2.91 (1H, td, *J* = 10.5, 4.1 Hz), 3.16–3.23 (1H, m), 3.53–3.60 (1H, m), 3.91–3.96 (2H, m), 6.17 (1H, d, *J* = 9.3 Hz), 6.72–6.78 (2H, m), 7.28 (1H, d, *J* = 8.4 Hz), 7.56 (1H, d, *J* = 9.3 Hz); ^13^C NMR (CDCl_3_, 75 MHz): δ/ppm 16.4, 21.2, 22.6, 23.5, 25.8, 26.0, 26.2, 29.1, 30.4, 31.7, 34.8, 40.7, 48.5, 68.5, 68.7, 79.4, 101.5, 112.5, 113.1, 113.2, 128.9, 143.7, 156.1, 161.6, 162.6. HRMS (ESI) *m*/*z*: calcd for C_25_H_36_O_4_ [M + Na]^+^, 423.2511; found, 423.2489.

### 4.3 CA Inhibition Assays

In order to determine the CA inhibition of the compounds, the method mentioned in the previous studies was used, and inhibition results were obtained.^24^

### 4.4 Cell Cytotoxicity Assay

The cytotoxicity of the test compounds on the human colorectal adenocarcinoma cell line (HT-29; HTB-38), human breast adenocarcinoma cell line (MCF7; HTB-22), human prostate adenocarcinoma cell line (PC3; CRL-1435), and human healthy kidney fibroblast cell line (HEK293T; CRL-3216) were evaluated by MTT (3-(4,5 dimethylthiazol-2-yl)-2,5- diphenyltetrazolium bromide) assay according to described methods.^8^ Briefly, the cell line was seeded in a flat-bottomed 96-well plate at a density of 5 × 10^3^ cells/well in DMEM F12/DMEM containing 10% FBS. The plate was incubated at 37 °C with 5% CO_2_ for 24 h, and then compounds were dissolved in DMSO and added to the medium to make final concentrations of 80, 40, 20, 10, 5, 2.5, 1.25, 0.62, 0.31, 0.16, and 0.08 μM. Cells were further incubated for 24 h at 37 °C with 5% CO_2_; then, medium-containing compounds were replaced with the fresh medium. 10 μL of filter-sterilized MTT solution (5 mg/mL in PBS) was added to each well and further incubated at 37 °C with 5% CO_2_ for 4 h. At the end of incubation, media was aspirated from the wells, and 100 μL of DMSO was added to dissolve the insoluble formazan crystals that formed. The absorbance was measured at 540 nm using a microtiter plate reader. The relative % cell viability was calculated from the following equation: relative percent cell viability = (*A*_test_ – *A*_blank_/*A*_control_ – *A*_blank_) × 100%. (*A*_test_ is the absorbance of the sample-treated cells, *A*_control_ is the absorbance of the untreated cells, and *A*_blank_ is the absorbance of the cell-free wells. Each absorbance was taken to be the mean of triplicate measurements). The cell viability was represented as a percentage relative to untreated cells as a control.

### 4.5 Western Blotting Assay

CA IX and CA XII protein expressions were evaluated in treated and control HT-29 and MCF7 cells after hypoxia induction. Cells were seeded in a 6-well plate in DMEM/F12 (normoxic cells) or DMEM/F12 containing 200 μM CoCl_2_ (hypoxic and treated cells) in order to create hypoxia.^25^ HT29 cells were treated with **14** (40, 10, and 1.25 μM), **23** (80, 20, and 1.25 μM), and **66** (80, 20, and 1.25 μM), and MCF7 cells were treated with **14** (40, 10, and 1.25 μM), **23** (80, 20, and 1.25 μM), and **63** (80, 20, and 1.25 μM) for 24 h at three different doses. After the treatment period, cells were washed with cold PBS and homogenized into Ripa cell lysis buffer (Santa Cruz, USA). Samples were prepared, electrophoresed, and transferred according to the literature.^25^ The membranes were blocked in 5% fat-free milk (w/v) for 1 h at RT, incubated with the corresponding antibody at 4 °C overnight, and then incubated with the horseradish peroxidase (HRP)-labeled secondary antibody for 1 h at RT. The following antibodies were used: anti-CA IX (ab107257, Abcam), anti-CA XII (sc-374314, Santa Cruz), and anti-GAPDH (A19056, Abclonal). Finally, the membranes were stained with ECL reagents (LumiGLO, CST, USA), and then, imaging was performed with the Bio-Rad Chemidoc Imaging System. The bands were calculated with the Image Lab (Bio-Rad, USA) and analyzed with Graphpad Prism 9.00 (San Diego, CA, USA).

### 4.6 Apoptosis Assay

Apoptosis assay was performed using the Muse Annexin V & Dead Cell Assay kit (Merck Millipore, Germany) in Muse Cell Analyzer (Merck Millipore, Germany). HT29 cells were treated with **14** (40, 10, and 1.25 μM), **23** (80, 20, and 1.25 μM), and **66** (80, 20, and 1.25 μM), and MCF7 cells were treated with **14** (40, 10, and 1.25 μM), **23** (80, 20, and 1.25 μM), and **63** (80, 20, and 1.25 μM) for 24 h at three different doses. After the treatment period, cells were collected and resuspended in PBS with 1% FBS, mixed with the Muse Annexin V and Dead Cell reagents. Samples were incubated for 20 min at room temperature in the dark. Apoptotic cell ratios were analyzed by flow cytometry using the Muse cell analyzer system, and gating was adjusted according to the untreated sample. Results were presented as the percentage of cells that were viable (Ann-V– 7-AAD−), early apoptotic (Ann-V+ 7-AAD−), late apoptotic (Ann-V+ 7-AAD−), or dead (Ann-V– 7-AAD+).^26^

### 4.7 Microscopy

HT-29 cells were stained with propidium iodide (PI) and Hoechst 33342 for observation of cell viability under the microscope. HT29 cells were treated with **14**, **23,** and **66** for 24 h at 1.25 μM concentration. After the treatment period cells were rinsed with PBS, fixed with ice-cold ethanol (95%) at −20 °C for 2 h, and then rinsed with PBS again. Cells were incubated with PI and Hoechst for 10 min and observed by using a fluorescence microscope (Zeiss Axio Observer Z1) with the appropriate excitation/detection filters.

### 4.8 Molecular Modeling

#### 4.8.1 Preparation of Protein Structures

The crystal structure of hCA IX and XII in complex with acetazolamide (pdb: 3iai and 1jd0) was obtained from the RCSB Protein Data Bank. Subsequently, the structures were prepared using the protein preparation tool of Schrödinger (v2021-1, Schrödinger, Inc., New York, USA). All water and buffer molecules were omitted. Subunit A was retained, and all other subunits, if present, were omitted. Subsequently, hydrogen atoms were added, and the system was minimized using the OPLS4 forcefield.

#### 4.8.2 Docking Studies

Compounds **23** and **70** were prepared in the open and closed coumarin forms using the LigPrep tool of Schrödinger and minimized with the OPLS4 force field. Subsequently, both stereoisomers were docked into the active sites of hCA IX and hCA XII using the Glide tool of Schrödinger with the XP settings. The three highest-scoring poses were obtained for each ligand, and the poses were subsequently minimized using the Prime tool and MM–GBSA forcefield. To this end, the ligand and all residues within 4 Å were unrestrained, except the zinc ion and zinc-binding residues.

#### 4.8.3 Molecular Dynamics Simulations

The ligand-enzyme complexes obtained with the docking procedure were subjected to a 50 ns MD simulation using Desmond. The complex was first placed in an orthorhombic box (at least 10 Å between the complex and boundary) and then filled with Tip5P water molecules and 0.15 M NaCl. The amount of Na or Cl atoms were adjusted to create a neutral system. Afterward, all heavy atoms were restrained, and the system was minimized for 100 ps using the OPLS4 forcefield. Finally, the system was simulated for 50 ns under isothermic (Nose–Hoover chain, 1 ps relaxation time) and isobaric (Martyna–Tobial–Klein, 2 ps relaxation time, isotropic coupling) conditions without restraints. Snapshots were saved every 100 ps.

## Supporting Information Available

^1^H, ^13^C NMR, and MS spectra of selected compounds and viability (%)—log concentration (μM) curves for the cytotoxic effects and graphs showing the CA inhibition of lead molecules and representative IR spectra (PDF)

## References

[ref1] Wang L. H.; Jiang X. R.; Yang J. Y.; Bao X. F.; Chen J. L.; Liu X.; Chen G. L.; Wu C. F. SYP-5, a novel HIF-1 inhibitor, suppresses tumor cells invasion and angiogenesis. Eur. J. Pharmacol. 2016, 791, 560–568. 10.1016/j.ejphar.2016.09.027. 27664769

[ref2] Peerlings J.; Van De Voorde L.; Mitea C.; Larue R.; Yaromina A.; Sandeleanu S.; Spiegelberg L.; Dubois L.; Lambin P.; Mottaghy F. M. Hypoxia and hypoxia response-associated molecular markers in esophageal cancer: A systematic review. Methods 2017, 130, 51–62. 10.1016/j.ymeth.2017.07.002. 28705470

[ref3] Yusuf Z. S.; Uysal T. K.; Simsek E.; Nocentini A.; Osman S. M.; Supuran C. T.; Özensoy Güler O. The inhibitory effect of boric acid on hypoxia-regulated tumour-associated carbonic anhydrase IX. J. Enzyme Inhib. Med. Chem. 2022, 37, 1340–1345. 10.1080/14756366.2022.2072837. 35535546 PMC9103596

[ref4] Supuran C. T. Carbonic anhydrase inhibitors. Bioorg. Med. Chem. Lett. 2010, 20, 3467–3474. 10.1016/j.bmcl.2010.05.009. 20529676

[ref5] Petreni A.; Osman S. M.; Alasmary F. A.; Almutairi T. M.; Nocentini A.; Supuran C. T. Binding site comparison for coumarin inhibitors and amine/amino acid activators of human carbonic anhydrases. Eur. J. Med. Chem. 2021, 226, 113875 10.1016/j.ejmech.2021.113875. 34634741

[ref6] Biernacki K.; Ciupak O.; Daśko M.; Rachon J.; Kozak W.; Rak J.; Kubiński K.; Masłyk M.; Martyna A.; Śliwka-Kaszyńska M.; et al. Development of Sulfamoylated 4-(1-Phenyl-1H-1,2,3-triazol-4-yl)phenol Derivatives as Potent Steroid Sulfatase Inhibitors for Efficient Treatment of Breast Cancer. J. Med. Chem. 2022, 65, 5044–5056. 10.1021/acs.jmedchem.1c02220. 35235747 PMC8958511

[ref7] Ilies M. A.; Winum J.-Y. Carbonic anhydrase inhibitors for the treatment of tumors: therapeutic, immunologic, and diagnostic tools targeting isoforms IX and XII. Carbonic Anhydrases 2019, 2019, 331–365. 10.1016/B978-0-12-816476-1.00016-2.

[ref8] Zengin Kurt B.; Sonmez F.; Ozturk D.; Akdemir A.; Angeli A.; Supuran C. T. Synthesis of coumarin-sulfonamide derivatives and determination of their cytotoxicity, carbonic anhydrase inhibitory and molecular docking studies. Eur. J. Med. Chem. 2019, 183, 111702 10.1016/j.ejmech.2019.111702. 31542715

[ref9] Tawfik H. O.; Petreni A.; Supuran C. T.; El-Hamamsy M. H. Discovery of new carbonic anhydrase IX inhibitors as anticancer agents by toning the hydrophobic and hydrophilic rims of the active site to encounter the dual-tail approach. Eur. J. Med. Chem. 2022, 232, 114190 10.1016/j.ejmech.2022.114190. 35182815

[ref10] Ewies E. F.; Sabry E.; Bekheit M. S.; Fouad M. A.; Vullo D.; Supuran C. T. Click chemistry-based synthesis of new benzenesulfonamide derivatives bearing triazole ring as selective carbonic anhydrase II inhibitors. Drug Dev. Res. 2022, 83, 1281 10.1002/ddr.21957. 35706360

[ref11] Mahon B. P.; Lomelino C. L.; Ladwig J.; Rankin G. M.; Driscoll J. M.; Salguero A. L.; Pinard M. A.; Vullo D.; Supuran C. T.; Poulsen S. A.; et al. Mapping Selective Inhibition of the Cancer-Related Carbonic Anhydrase IX Using Structure-Activity Relationships of Glucosyl-Based Sulfamates. J. Med. Chem. 2015, 58, 6630–6638. 10.1021/acs.jmedchem.5b00845. 26203869

[ref12] Giovannuzzi S.; De Luca V.; Nocentini A.; Capasso C.; Supuran C. T. Coumarins inhibit eta-class carbonic anhydrase from Plasmodium falciparum. J. Enzyme Inhib. Med. Chem. 2022, 37, 680–685. 10.1080/14756366.2022.2036986. 35139744 PMC8843172

[ref13] Maresca A.; Temperini C.; Pochet L.; Masereel B.; Scozzafava A.; Supuran C. T. Deciphering the Mechanism of Carbonic Anhydrase Inhibition with Coumarins and Thiocoumarins. J. Med. Chem. 2010, 53, 335–344. 10.1021/jm901287j. 19911821

[ref14] Supuran C. T. Exploring the multiple binding modes of inhibitors to carbonic anhydrases for novel drug discovery. Expert Opin. Drug Discovery 2020, 15, 671–686. 10.1080/17460441.2020.1743676. 32208982

[ref15] Bonardi A.; Nocentini A.; Bua S.; Combs J.; Lomelino C.; Andring J.; Lucarini L.; Sgambellone S.; Masini E.; McKenna R.; et al. Sulfonamide Inhibitors of Human Carbonic Anhydrases Designed through a Three-Tails Approach: Improving Ligand/Isoform Matching and Selectivity of Action. J. Med. Chem. 2020, 63, 7422–7444. 10.1021/acs.jmedchem.0c00733. 32519851 PMC8008423

[ref16] Andring J. T.; Fouch M.; Akocak S.; Angeli A.; Supuran C. T.; Ilies M. A.; McKenna R. Structural Basis of Nanomolar Inhibition of Tumor-Associated Carbonic Anhydrase IX: X-Ray Crystallographic and Inhibition Study of Lipophilic Inhibitors with Acetazolamide Backbone. J. Med. Chem. 2020, 63, 13064–13075. 10.1021/acs.jmedchem.0c01390. 33085484

[ref17] Kumar K. S.; Siwach K.; Rom T.; Kumar R.; Angeli A.; Kumar Paul A.; Supuran C. T.; Sharma P. K. Tail-approach based design and synthesis of Arylthiazolylhydrazono-1,2,3-triazoles incorporating sulfanilamide and metanilamide as human carbonic anhydrase I, II, IV and IX inhibitors. Bioorg. Chem. 2022, 123, 105764 10.1016/j.bioorg.2022.105764. 35366582

[ref18] Grande R.; Carradori S.; Puca V.; Vitale I.; Angeli A.; Nocentini A.; Bonardi A.; Gratteri P.; Lanuti P.; Bologna G.; et al. Selective Inhibition of Helicobacter pylori Carbonic Anhydrases by Carvacrol and Thymol Could Impair Biofilm Production and the Release of Outer Membrane Vesicles. Int. J. Mol. Sci. 2021, 22, 11583 10.3390/ijms222111583. 34769015 PMC8584244

[ref19] Supuran C. T. Coumarin carbonic anhydrase inhibitors from natural sources. J. Enzyme Inhib. Med. Chem. 2020, 35, 1462–1470. 10.1080/14756366.2020.1788009. 32779543 PMC7470141

[ref20] Kurt B. Z.; Dag A.; Doğan B.; Durdagi S.; Angeli A.; Nocentini A.; Supuran C. T.; Sonmez F. Synthesis, biological activity and multiscale molecular modeling studies of bis-coumarins as selective carbonic anhydrase IX and XII inhibitors with effective cytotoxicity against hepatocellular carcinoma. Bioorg. Chem. 2019, 87, 838–850. 10.1016/j.bioorg.2019.03.003. 31003041

[ref21] Hogendorf W. F. J.; Verhagen C. P.; Malta E.; Goosen N.; Overkleeft H. S.; Filippov D. V.; Van der Marel G. A. The synthesis of a menthol derivative of 2-aminopurine as a fluorescent DNA lesion. Tetrahedron 2009, 65, 10430–10435. 10.1016/j.tet.2009.10.023.

[ref22] Dag A.; Sahin H.; Durmaz H.; Hizal G.; Tunca U. Block-Brush Copolymers via ROMP and Sequential Double Click Reaction Strategy. J. Polym. Sci., Polym. Chem. 2011, 49, 886–892. 10.1002/pola.24499.

[ref23] Chen Y.; Wang S. L.; Xu X. Q.; Liu X.; Yu M. Q.; Zhao S.; Liu S. C.; Qiu Y. L.; Zhang T.; Liu B. F.; et al. Synthesis and Biological Investigation of Coumarin Piperazine (Piperidine) Derivatives as Potential Multireceptor Atypical Antipsychotics. J. Med. Chem. 2013, 56, 4671–4690. 10.1021/jm400408r. 23675993

[ref24] Khalifah R. G. The carbon dioxide hydration activity of carbonic anhydrase. I. Stop-flow kinetic studies on the native human isoenzymes B and C. J. Biol. Chem. 1971, 246, 2561–2573. 10.1016/s0021-9258(18)62326-9. 4994926

[ref25] Wu D. L.; Yotnda P. Induction and Testing of Hypoxia in Cell Culture. J. Visualized Exp. 2011, 12, 2899 10.3791/2899. PMC3217626 21860378

[ref26] Goncu B.; Sevgi E.; Hancer C. K.; Gokay G.; Ozten N. Correction: Differential anti-proliferative and apoptotic effects of lichen species on human prostate carcinoma cells. PLoS One 2020, 15, e0244831 10.1371/journal.pone.0244831. 33370387 PMC7769444

