# Supplementary material for: Tail-Approach-Based Design and Synthesis of Coumarin-Monoterpenes as Carbonic Anhydrase Inhibitors and Anticancer Agents
Source: ACS Omega. 2023 Feb 3;8(6):5787–807. doi: 10.1021/acsomega.2c07459 (PMC9933483; doi:10.1021/acsomega.2c07459)
Supplement: Supplementary file 1 — ao2c07459_si_001.pdf [file ao2c07459_si_001.pdf]

Supporting Information for the Paper

Tail-Approach Based Design and Synthesis of Coumarin-Monoterpenes as Carbonic Anhydrase Inhibitors and Anticancer Agents

Belma Zengin Kurt[1,*], Gulsen Celebi[2], Dilek Öztürk Civelek[3], Andrea Angeli[4], Atilla Akdemir[5], Fatih Sonmez[6], Claudiu T. Supuran[4]

[1]Bezmialem Vakif University, Faculty of Pharmacy, Department of Pharmaceutical Chemistry, 34093, Istanbul, Türkiye
[2]Kocaeli University, Institute of Medicine, 41001, Kocaeli, Türkiye
[3]Bezmialem Vakif University, Faculty of Pharmacy, Department of Pharmacology, 34093, Istanbul, Türkiye
[4]Università degli Studi di Firenze, Dipartimento Neurofarba, Sezione di Scienze Farmaceutiche e Nutraceutiche, Via U. Schiff 6, 50019 Sesto Fiorentino, Florence, Italy
[5]Bezmialem Vakif University, Faculty of Pharmacy, Department of Pharmacology, Computer-aided drug discovery laboratory, 34093, Istanbul, Türkiye
[6]Sakarya University of Applied Sciences, Pamukova Vocational School, 54055, Sakarya, Türkiye

*Corresponding author: Belma Zengin Kurt; e-mail: bzengin@bezmialem.edu.tr

Table of Contents

^{1}H NMR, ^{13}C NMR, and MS spectrums of some selected compounds **S2**
IC_{50} curves of cytotoxicity **S49**
The HPLC trace **S62**
IR spectra for **3b**, **3c**, **3g** and **3h** **S65**
Graphs for CA inhibition of lead molecules; **14**, **23**, **63** and **66** **S67**

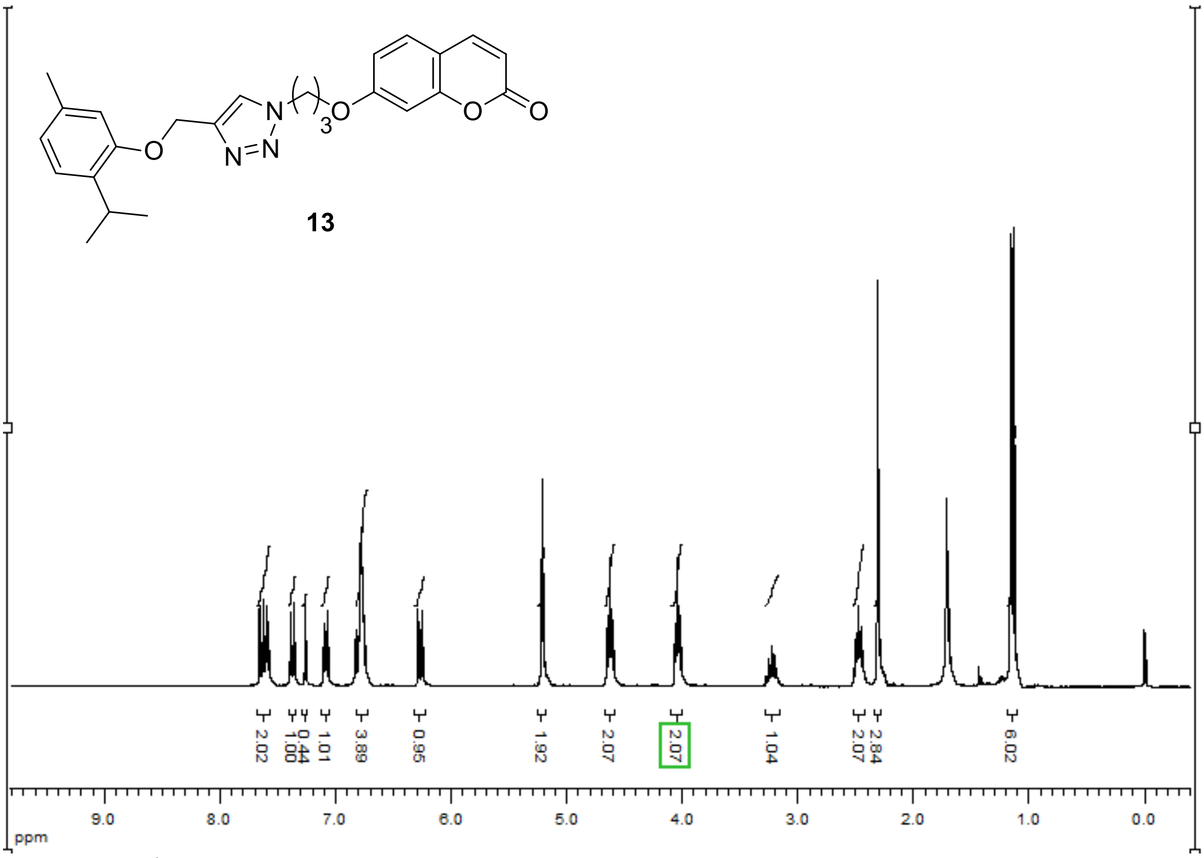

Figure S1. ^{1}H NMR (300 MHz, $CDCl_3$) spectrum of **13**

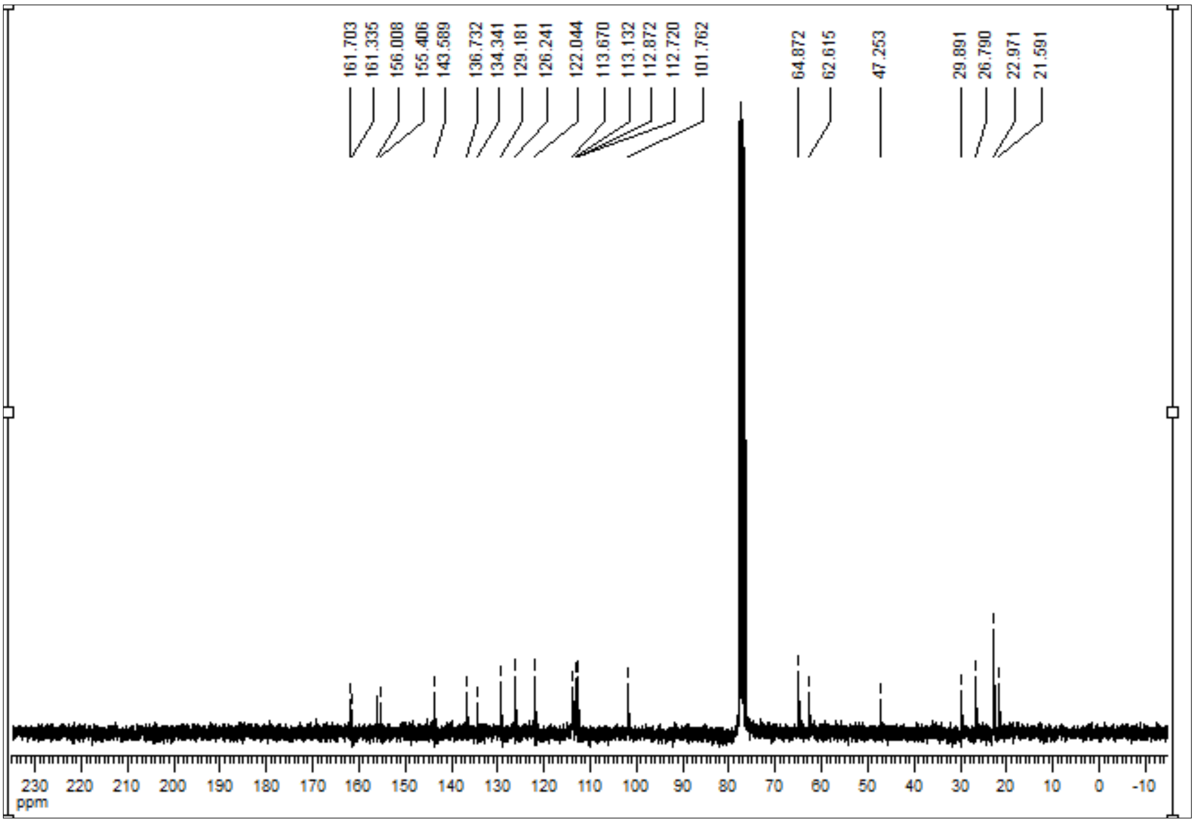

Figure S2. ^{13}C NMR (75 MHz, $CDCl_3$) spectrum of **13**

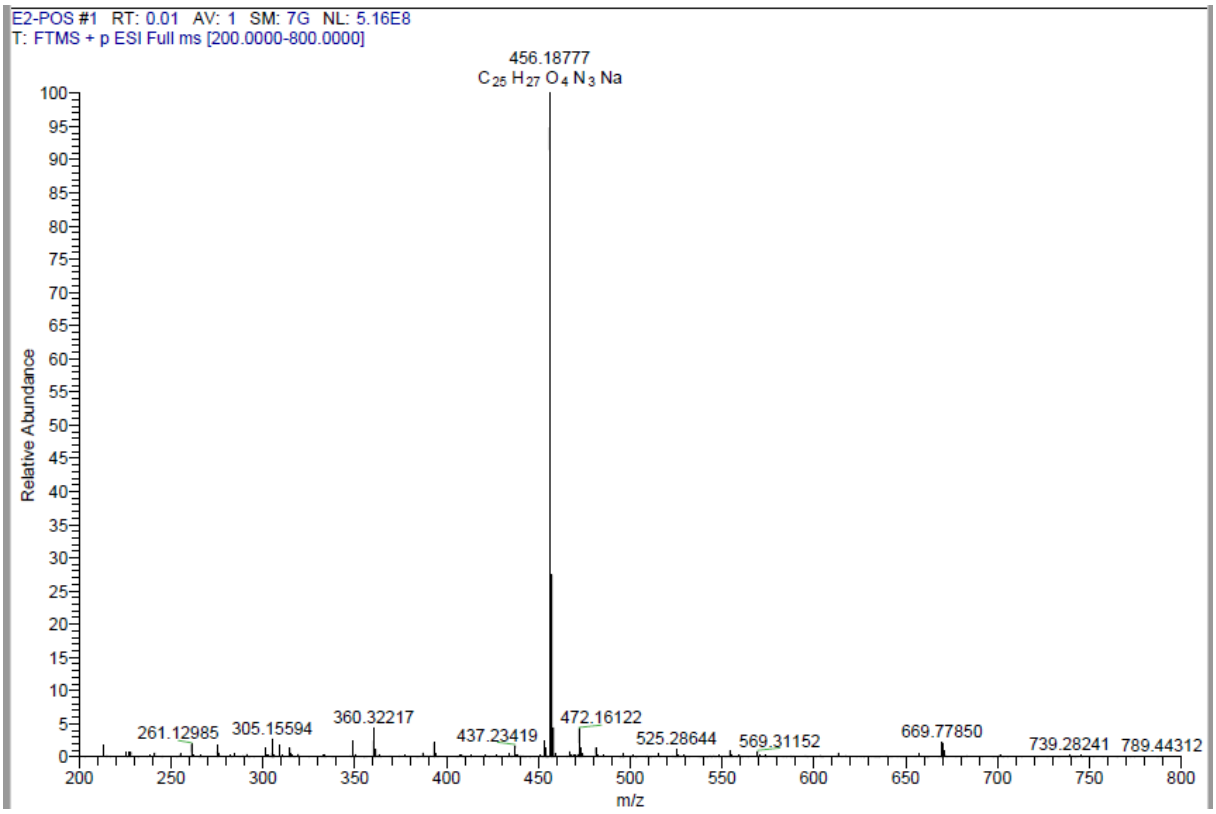

Figure S3. Mass spectrum of **13**

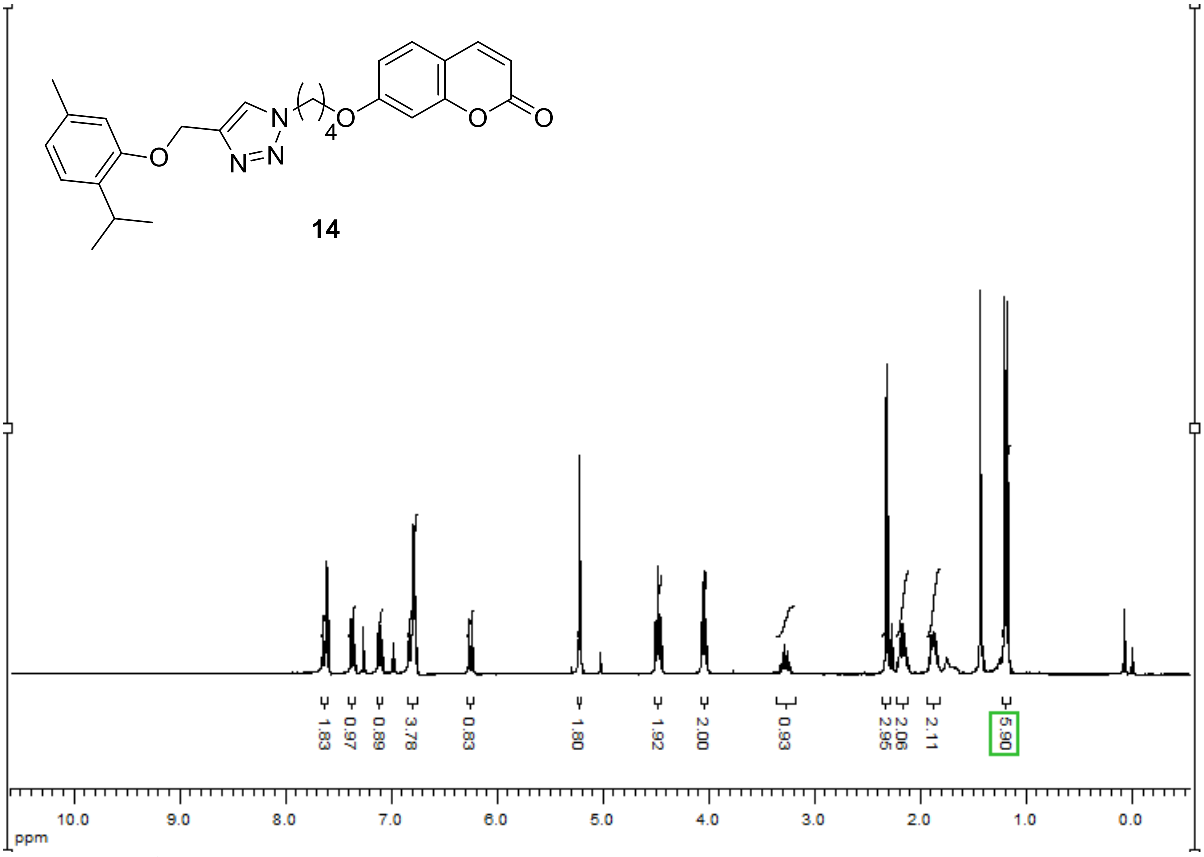

Figure S4. ^{1}H NMR (300 MHz, $CDCl_3$) spectrum of **14**

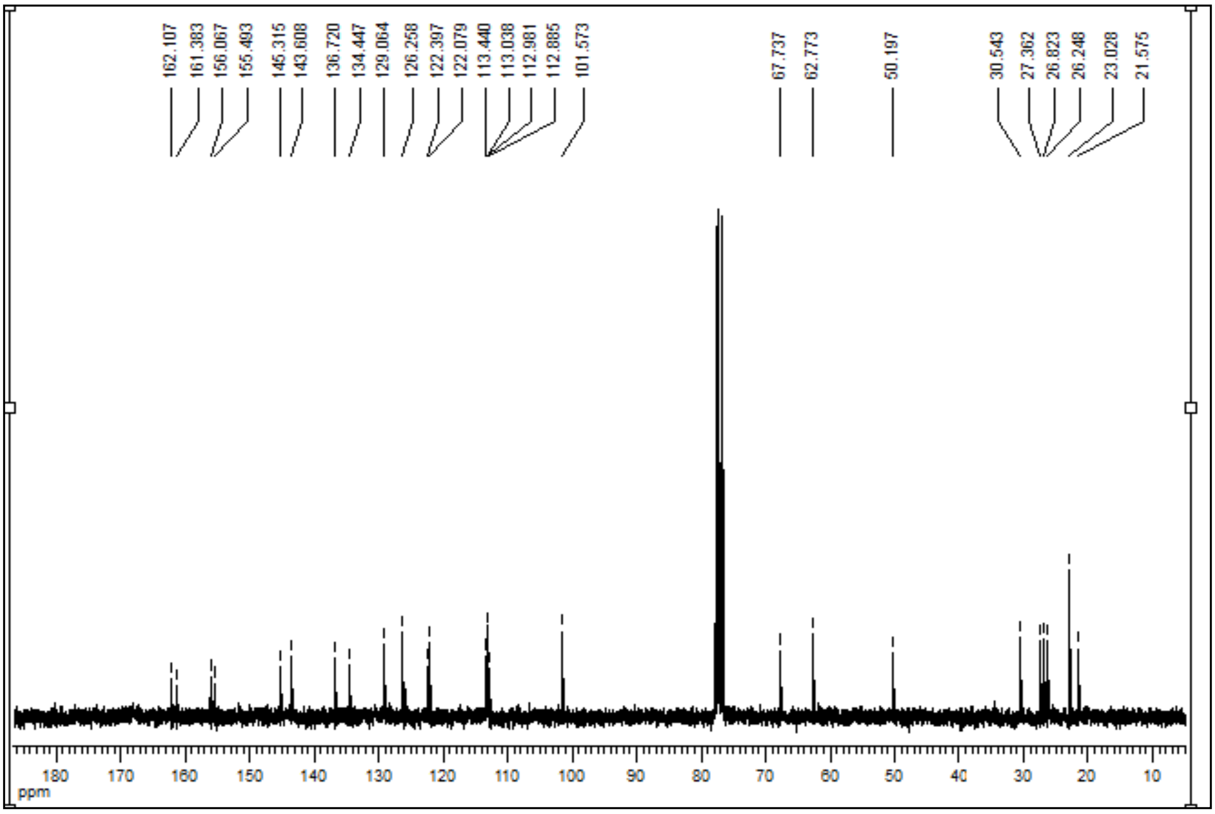

Figure S5. ^{13}C NMR (75 MHz, $CDCl_3$) spectrum of **14**

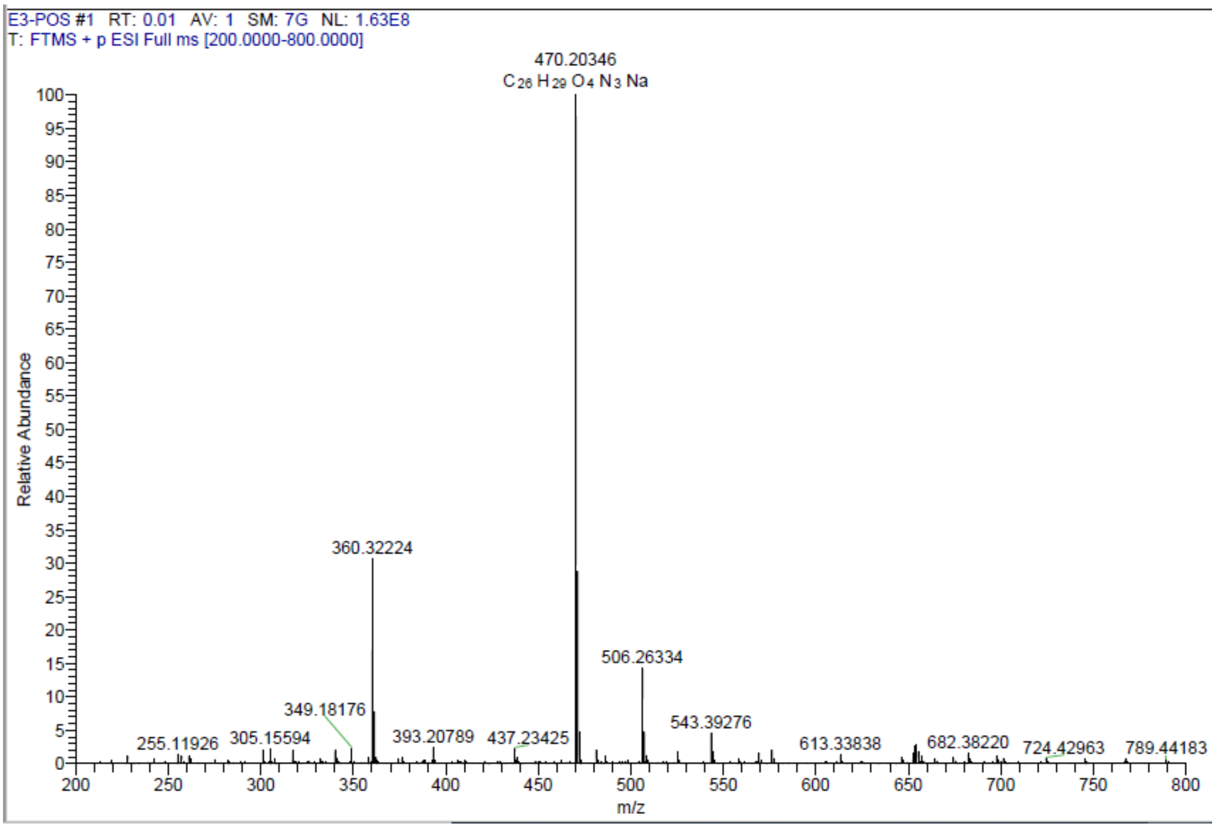

Figure S6. Mass spectrum of **14**

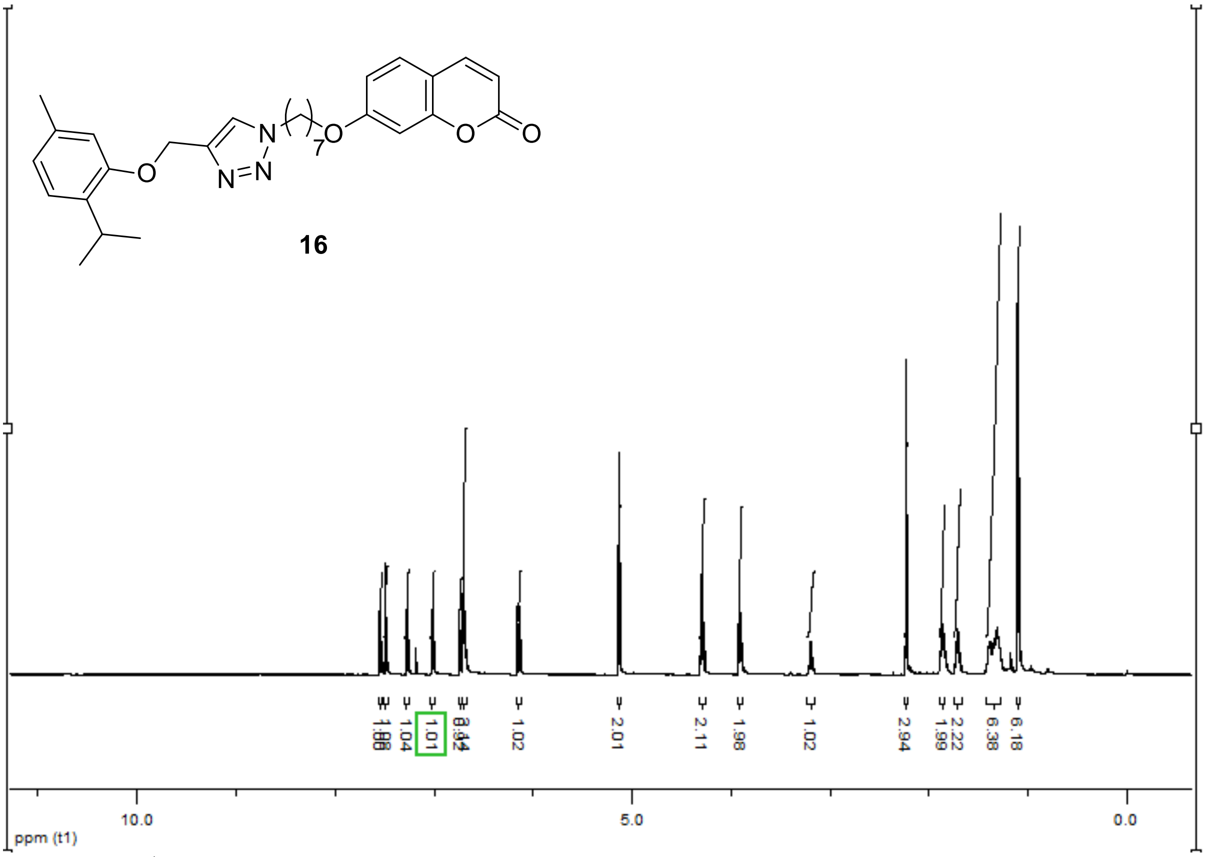

Figure S7. ^{1}H NMR (300 MHz, $CDCl_3$) spectrum of **16**

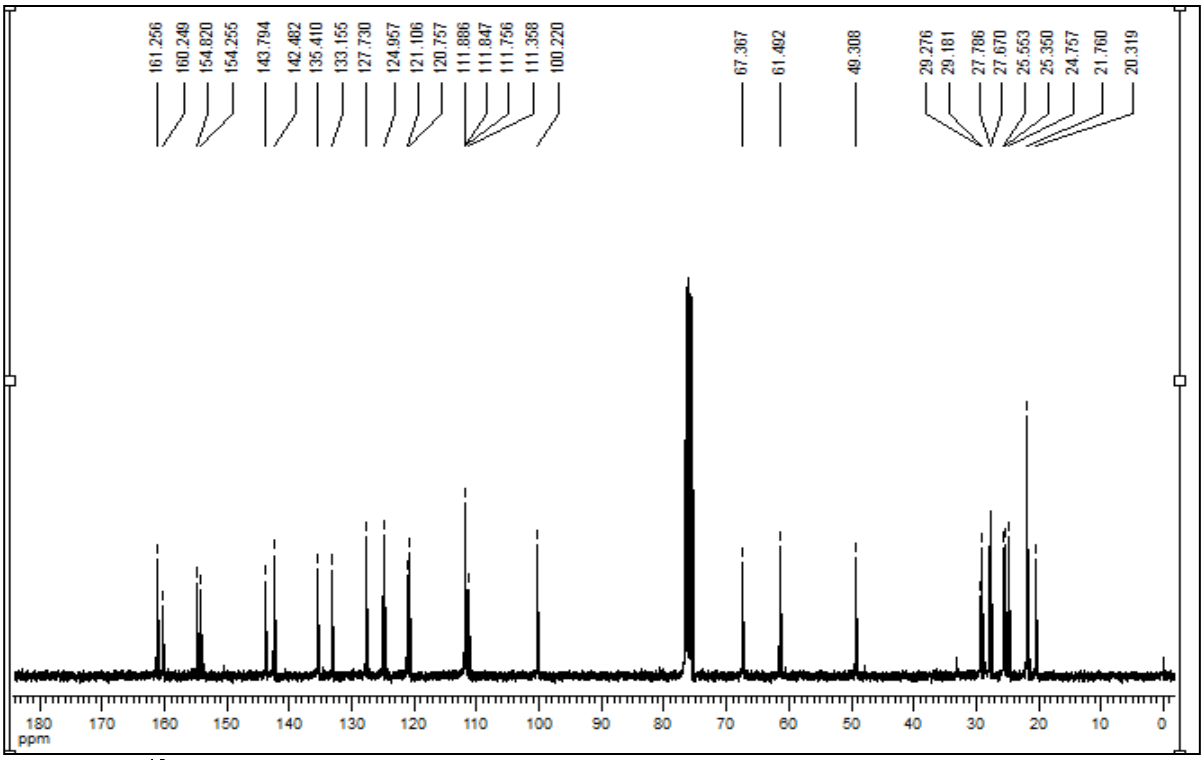

Figure S8. ^{13}C NMR (75 MHz, $CDCl_3$) spectrum of **16**

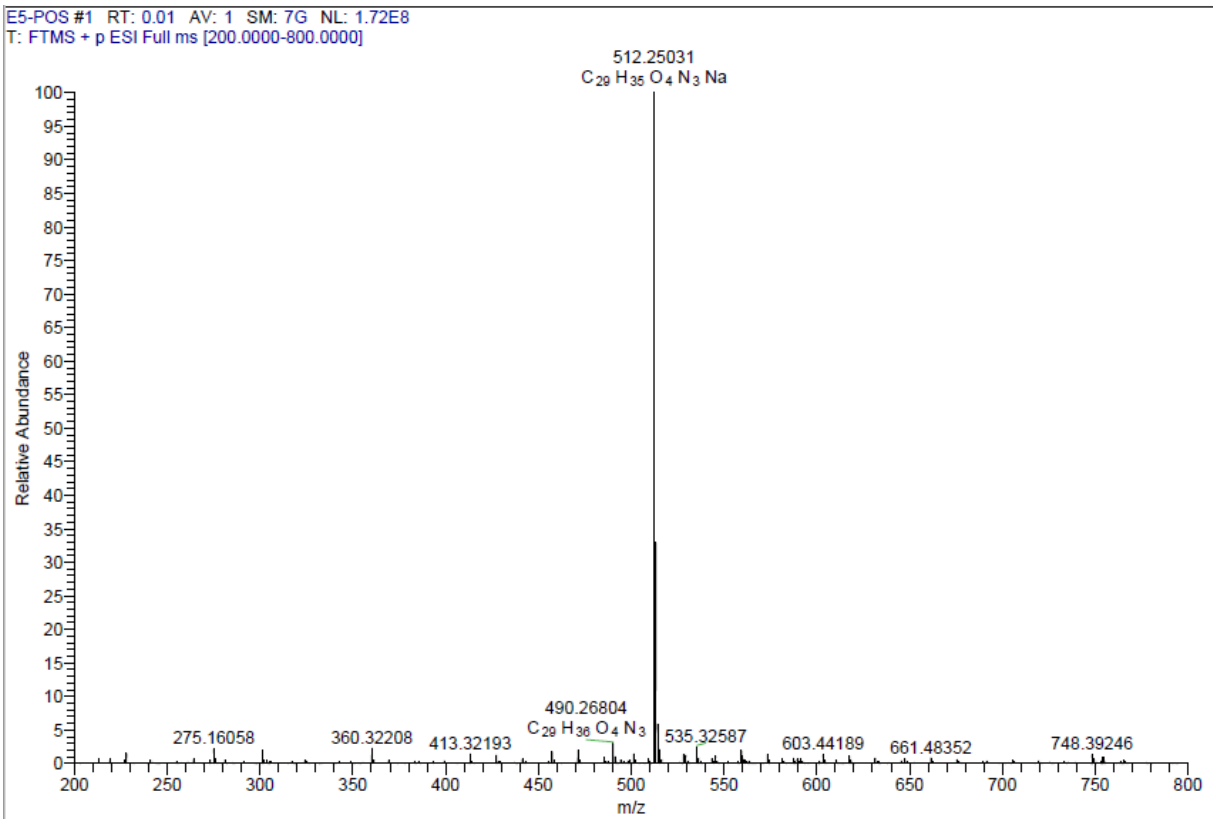

Figure S9. Mass spectrum of **16**

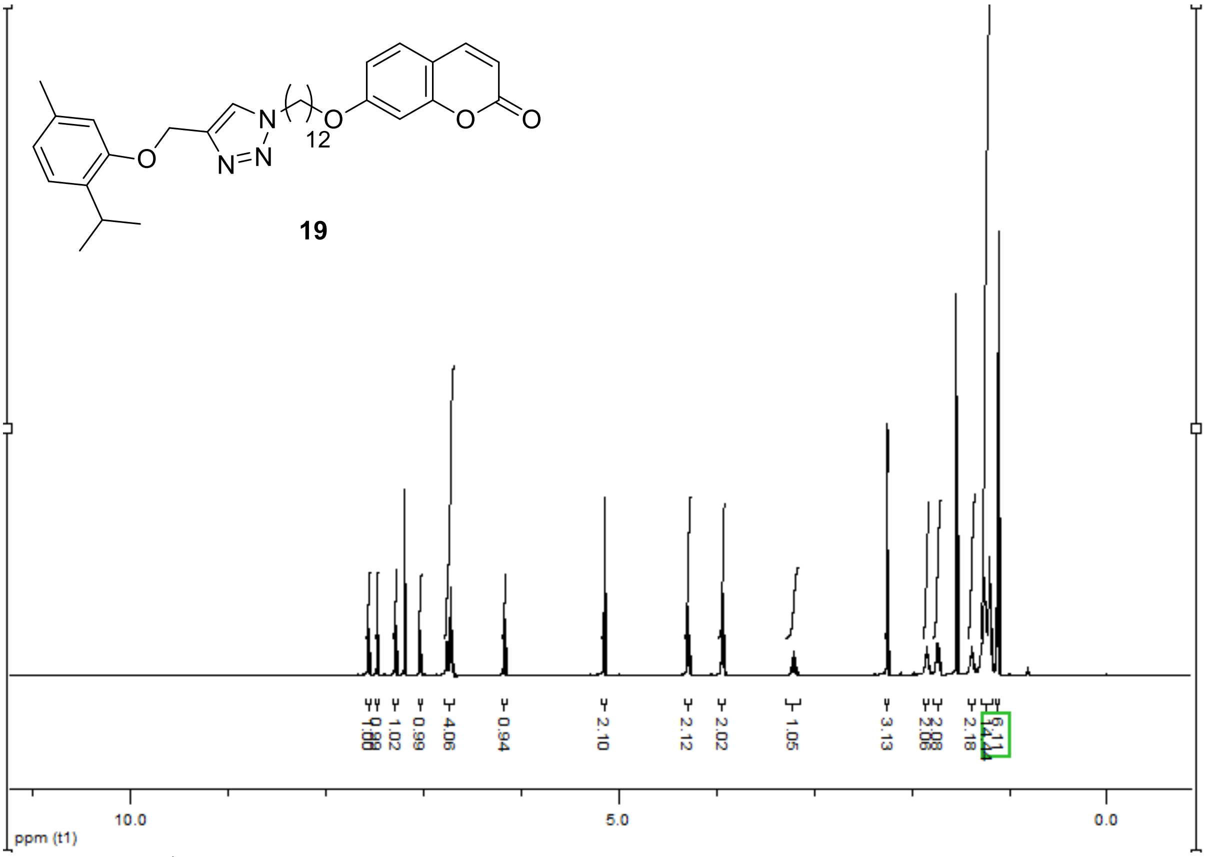

Figure S10. ^{1}H NMR (500 MHz, $CDCl_3$) spectrum of **19**

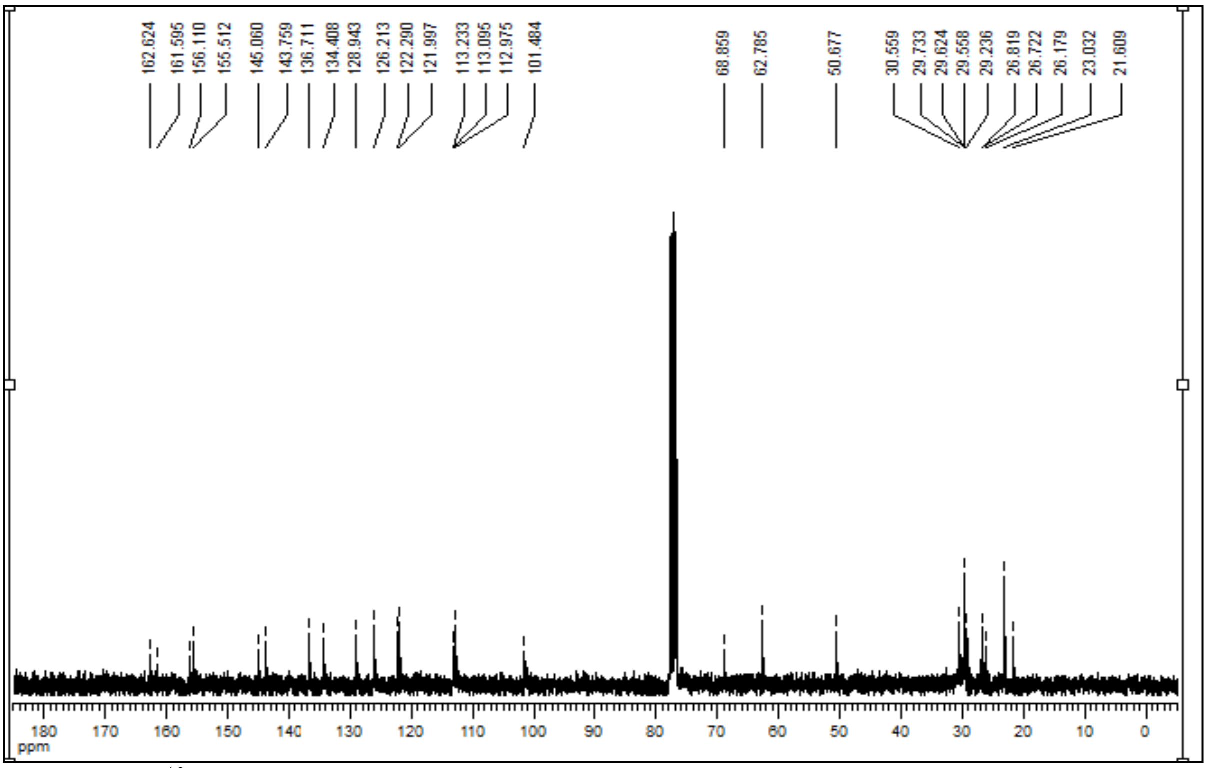

Figure S11. ^{13}C NMR (75 MHz, $CDCl_3$) spectrum of **19**

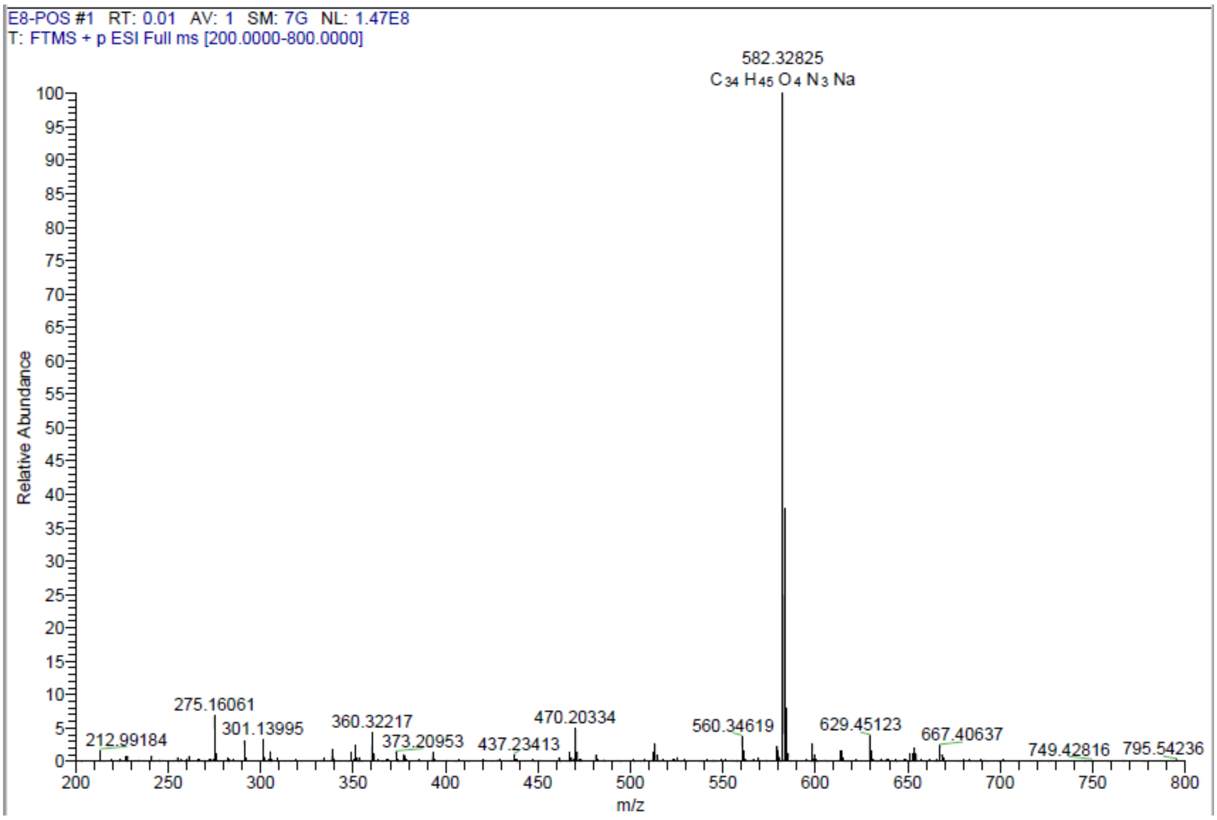

Figure S12. Mass spectrum of **19**

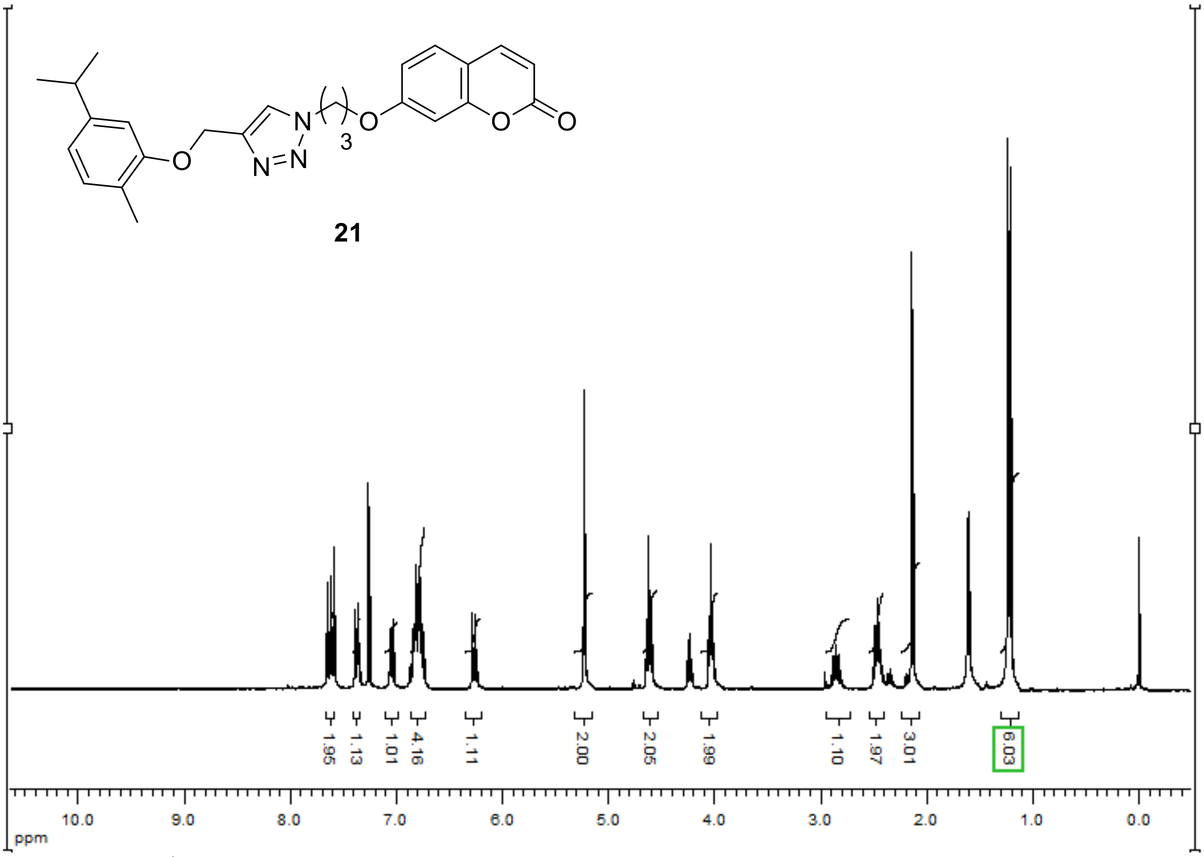

Figure S13. ^{1}H NMR (300 MHz, $CDCl_3$) spectrum of **21**

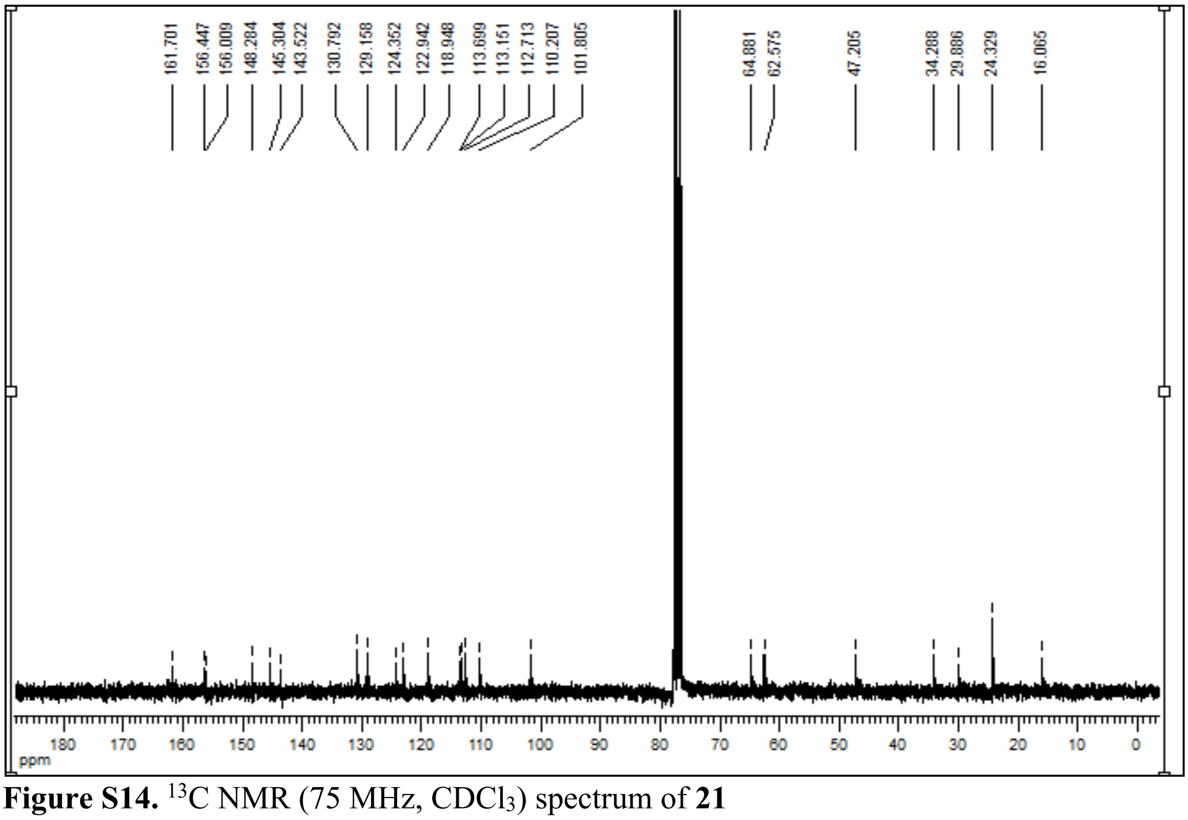

Figure S14. ^{13}C NMR (75 MHz, $CDCl_3$) spectrum of **21**

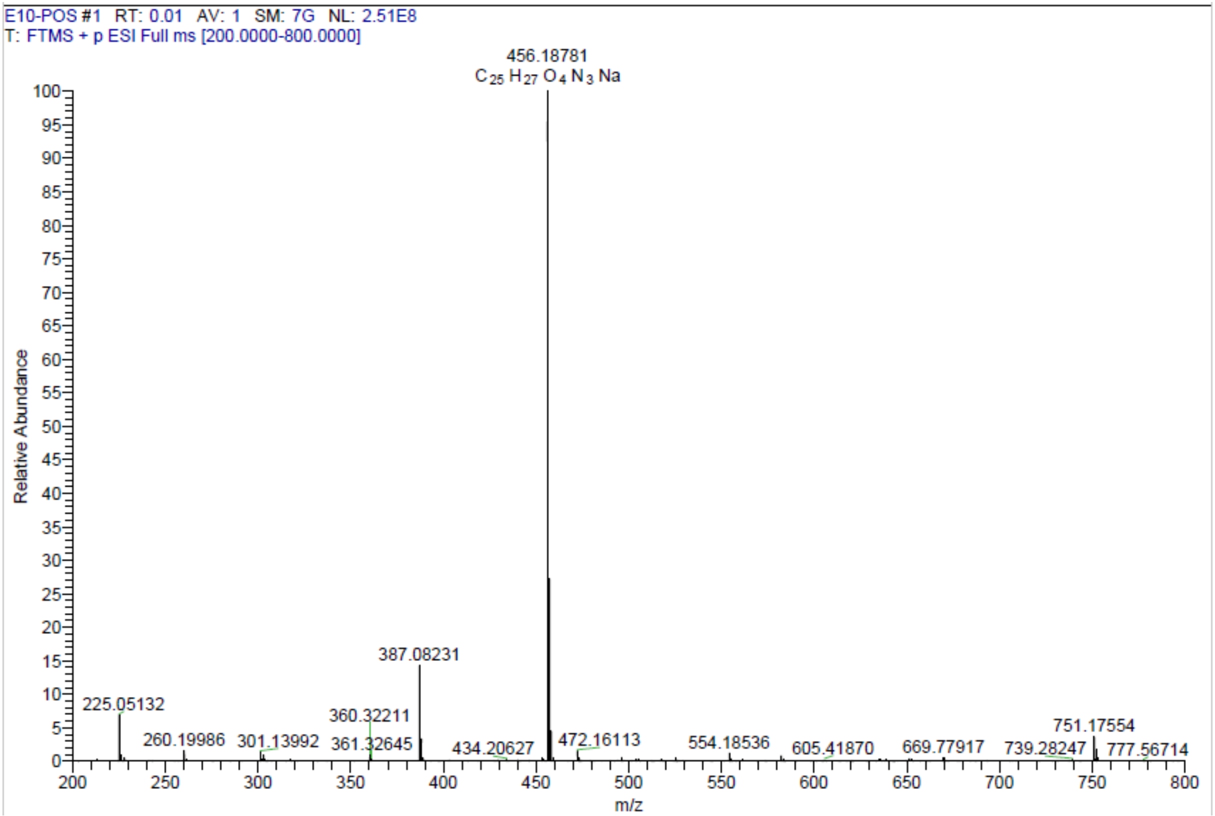

Figure S15. Mass spectrum of **21**

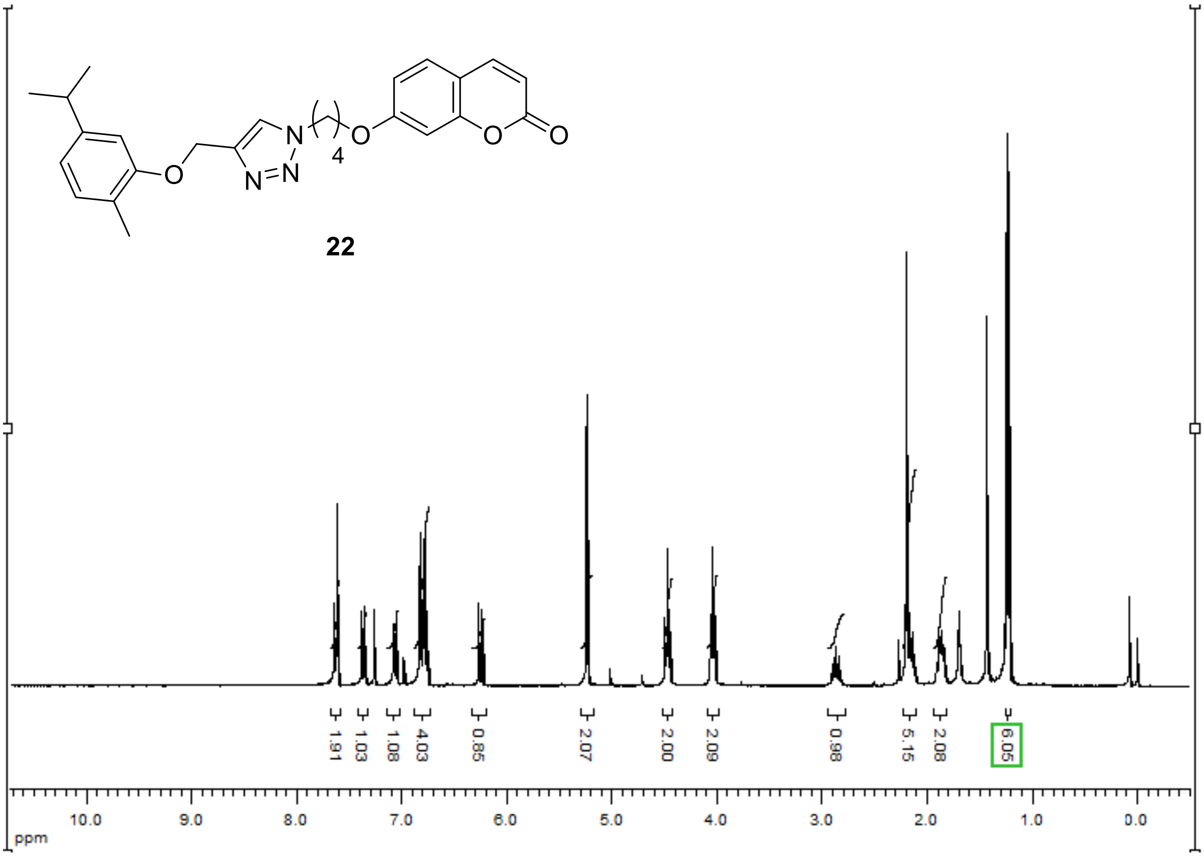

Figure S16. ^{1}H NMR (300 MHz, $CDCl_3$) spectrum of **22**

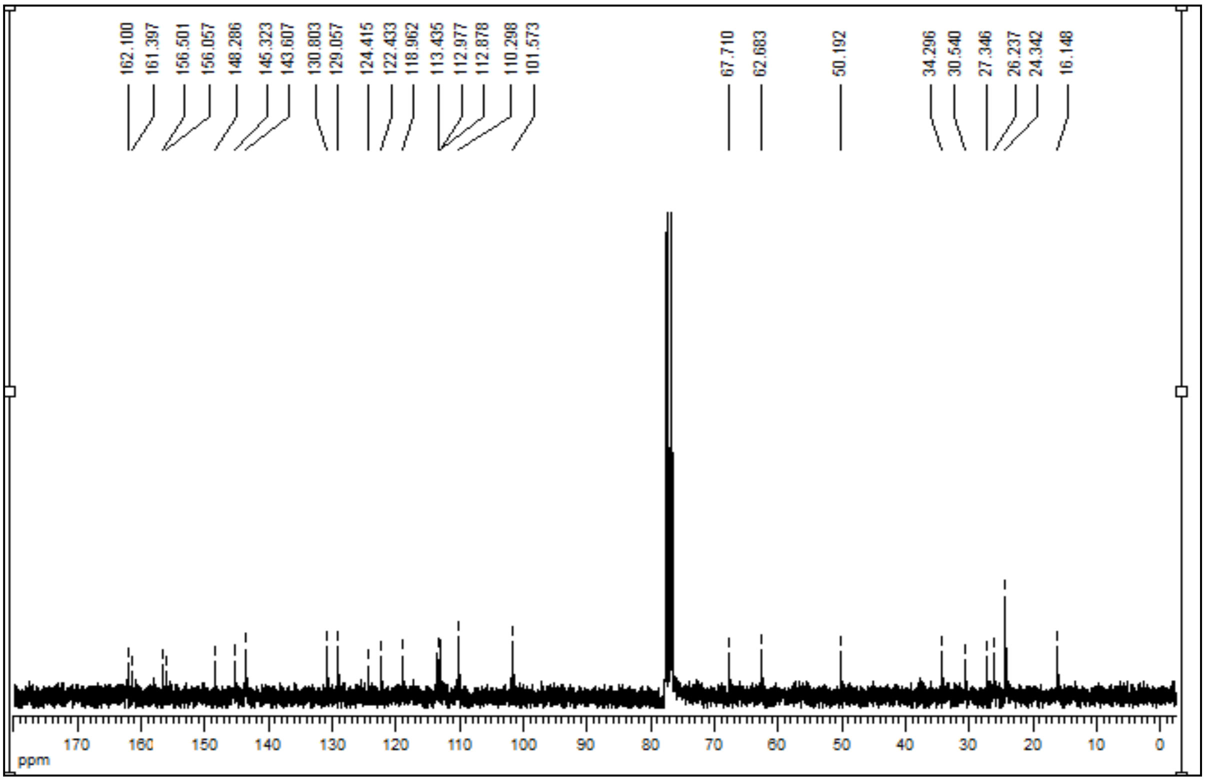

Figure S17. ^{13}C NMR (75 MHz, $CDCl_3$) spectrum of **22**

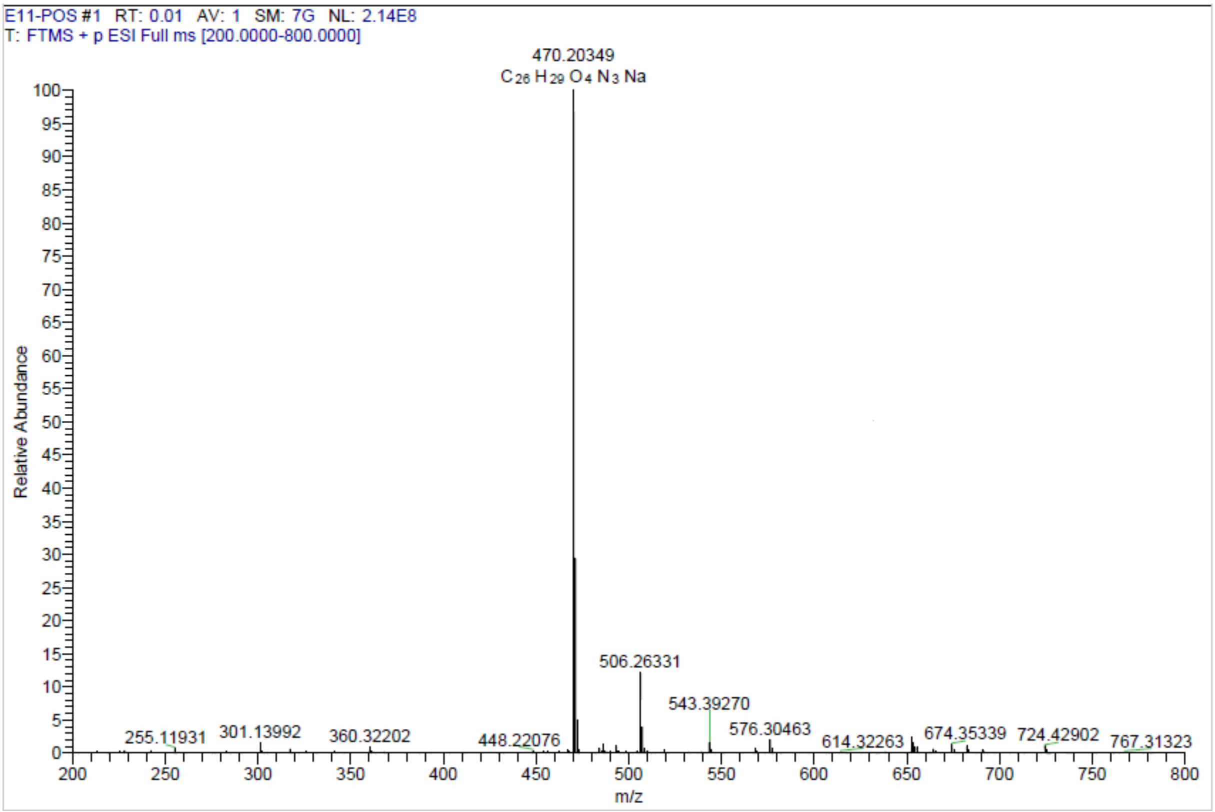

Figure S18. Mass spectrum of **22**

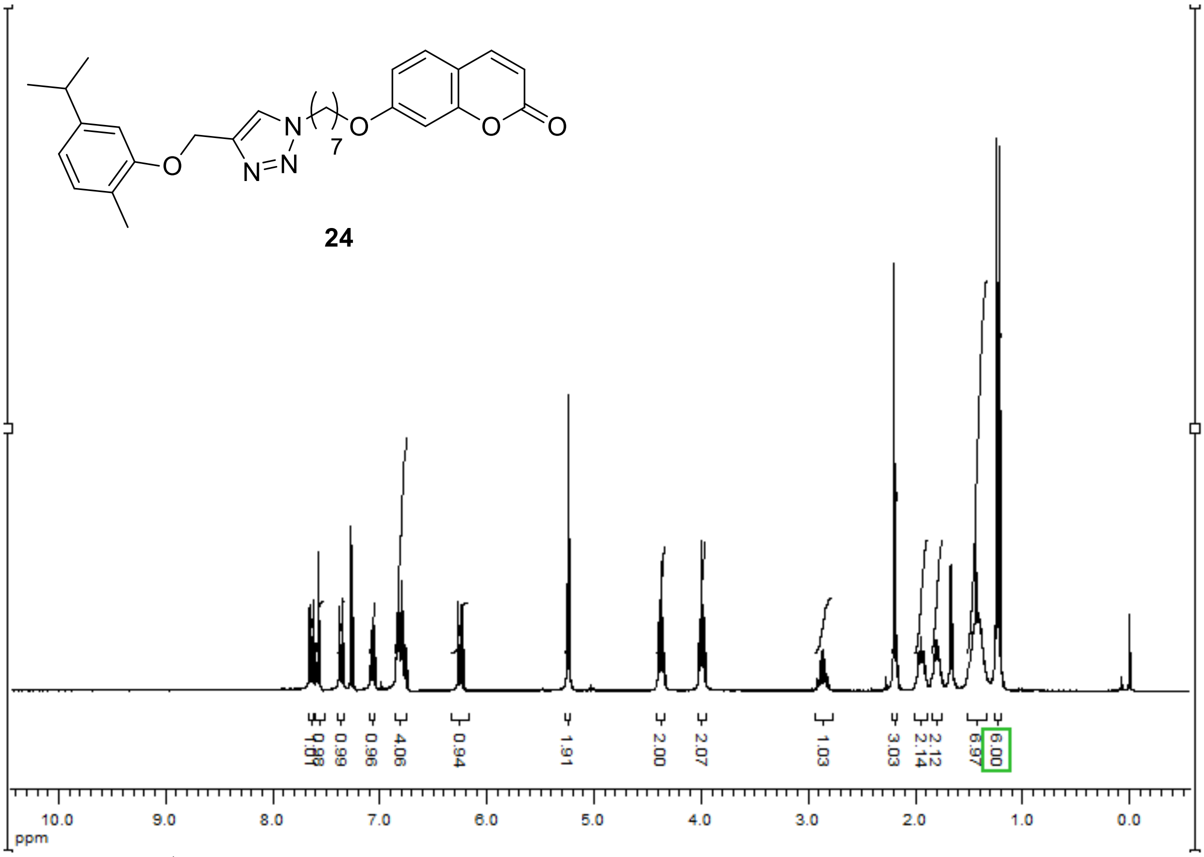
Figure S19. ^{1}H NMR (300 MHz, $CDCl_3$) spectrum of **24**

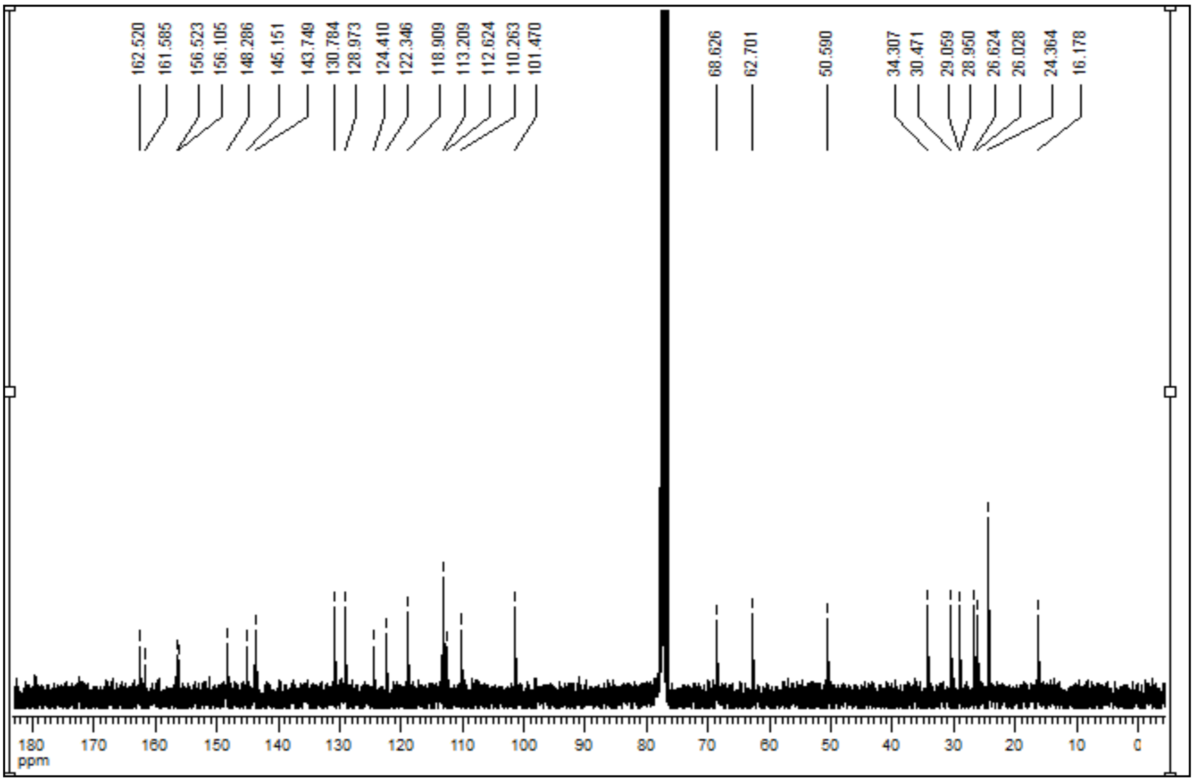
Figure S20. ^{13}C NMR (75 MHz, $CDCl_3$) spectrum of **24**

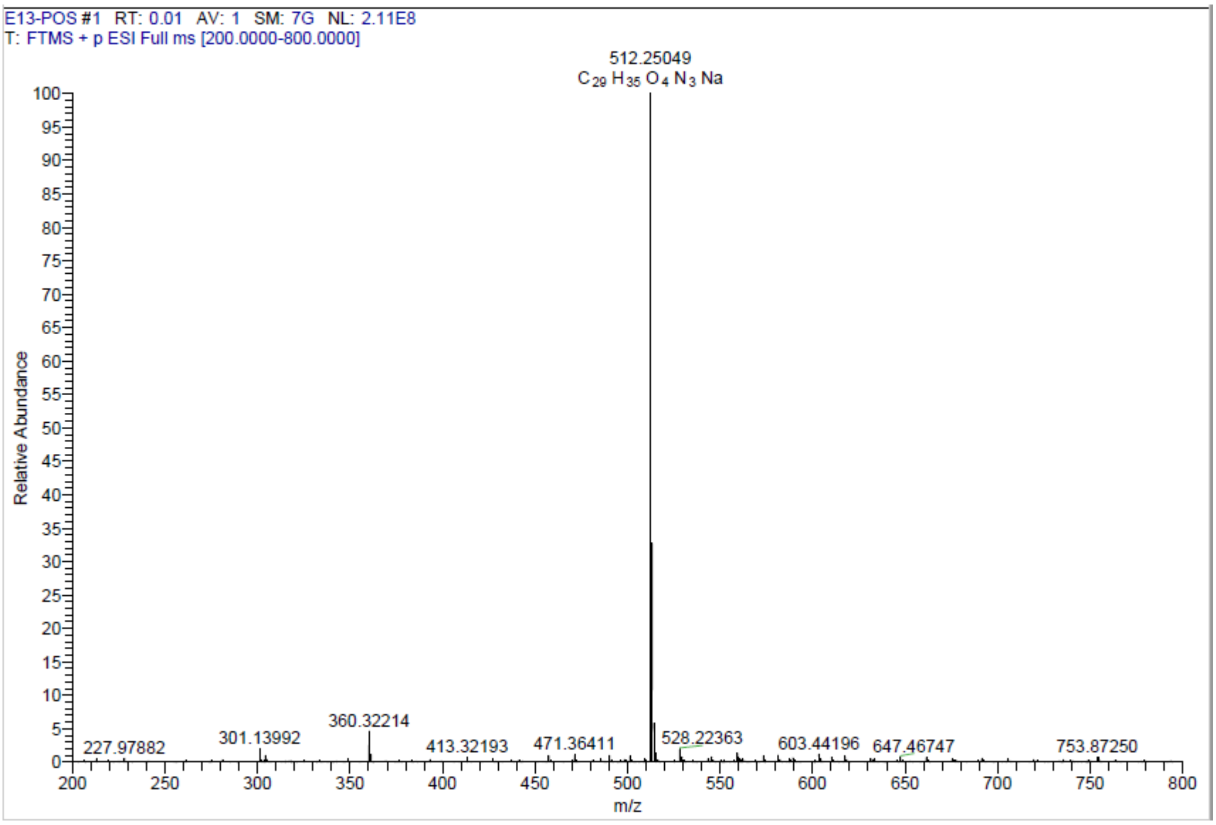

Figure S21. Mass spectrum of **24**

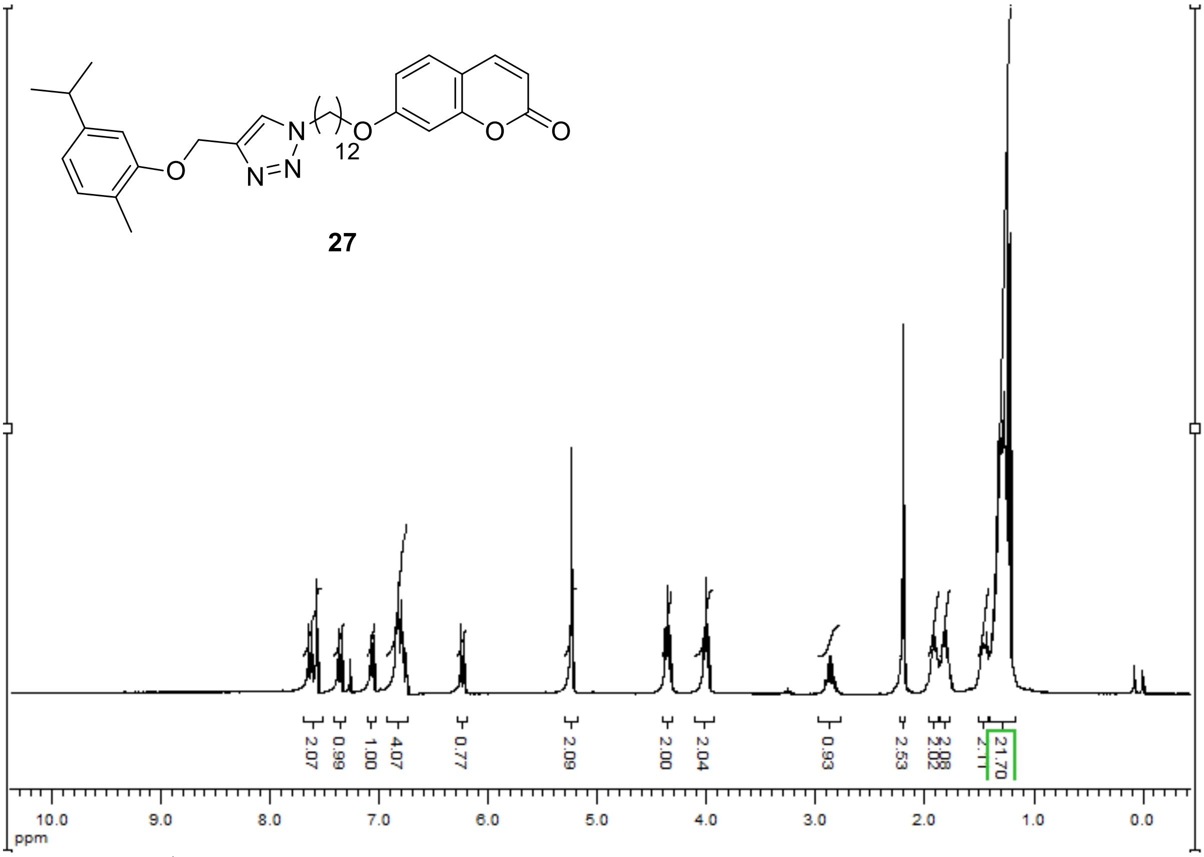

Figure S22. ^{1}H NMR (300 MHz, $CDCl_3$) spectrum of **27**

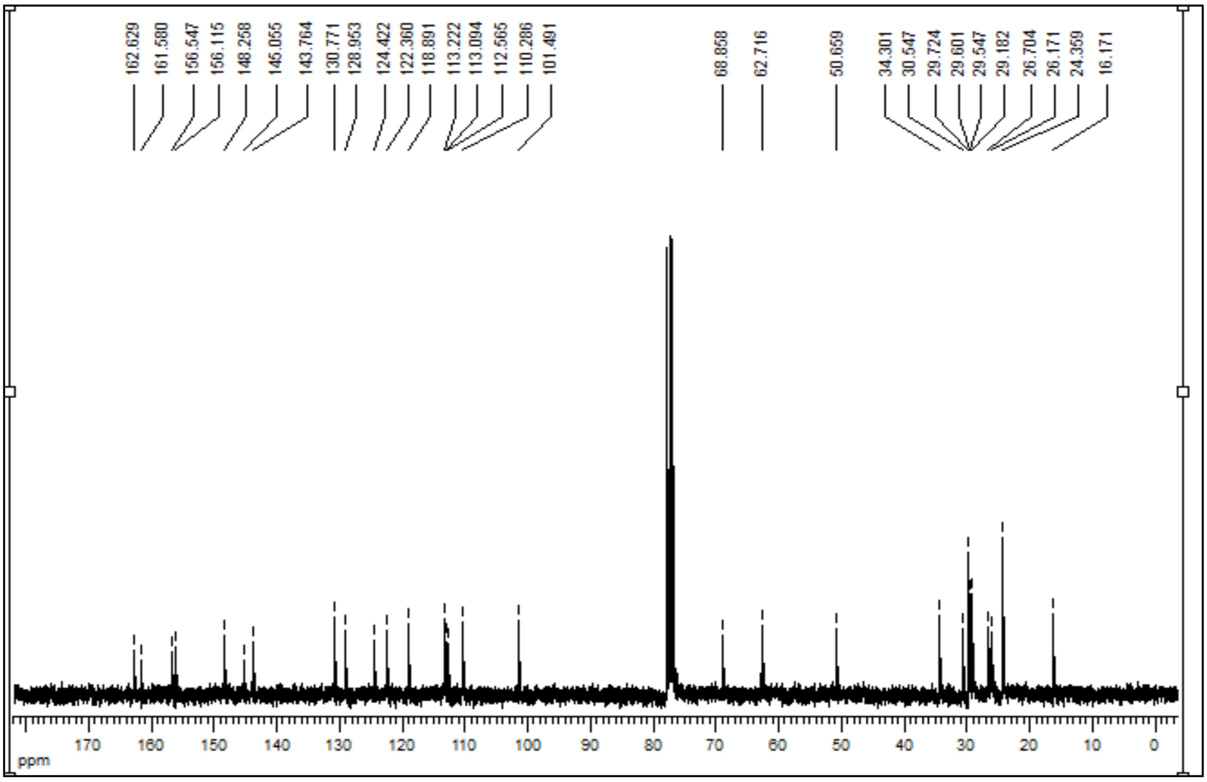

Figure S23. ^{13}C NMR (75 MHz, $CDCl_3$) spectrum of **27**

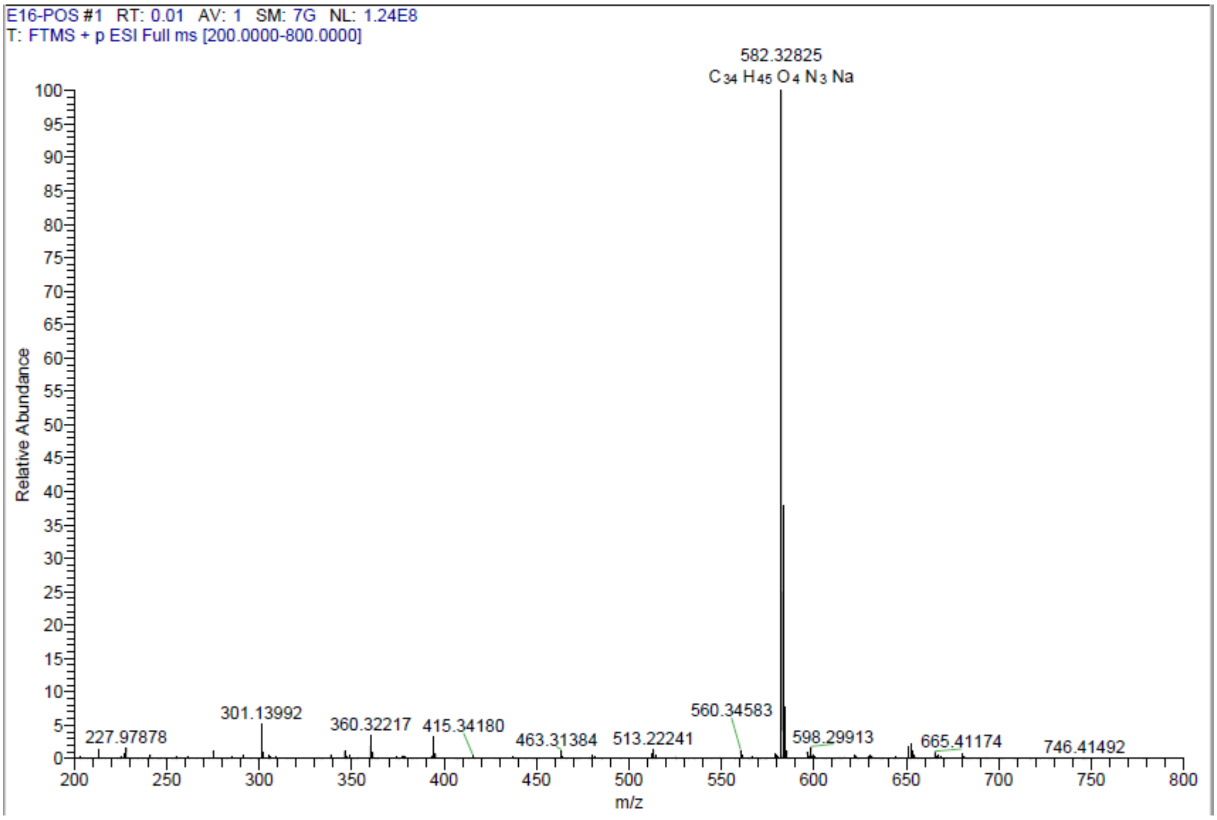

Figure S24. Mass spectrum of **27**

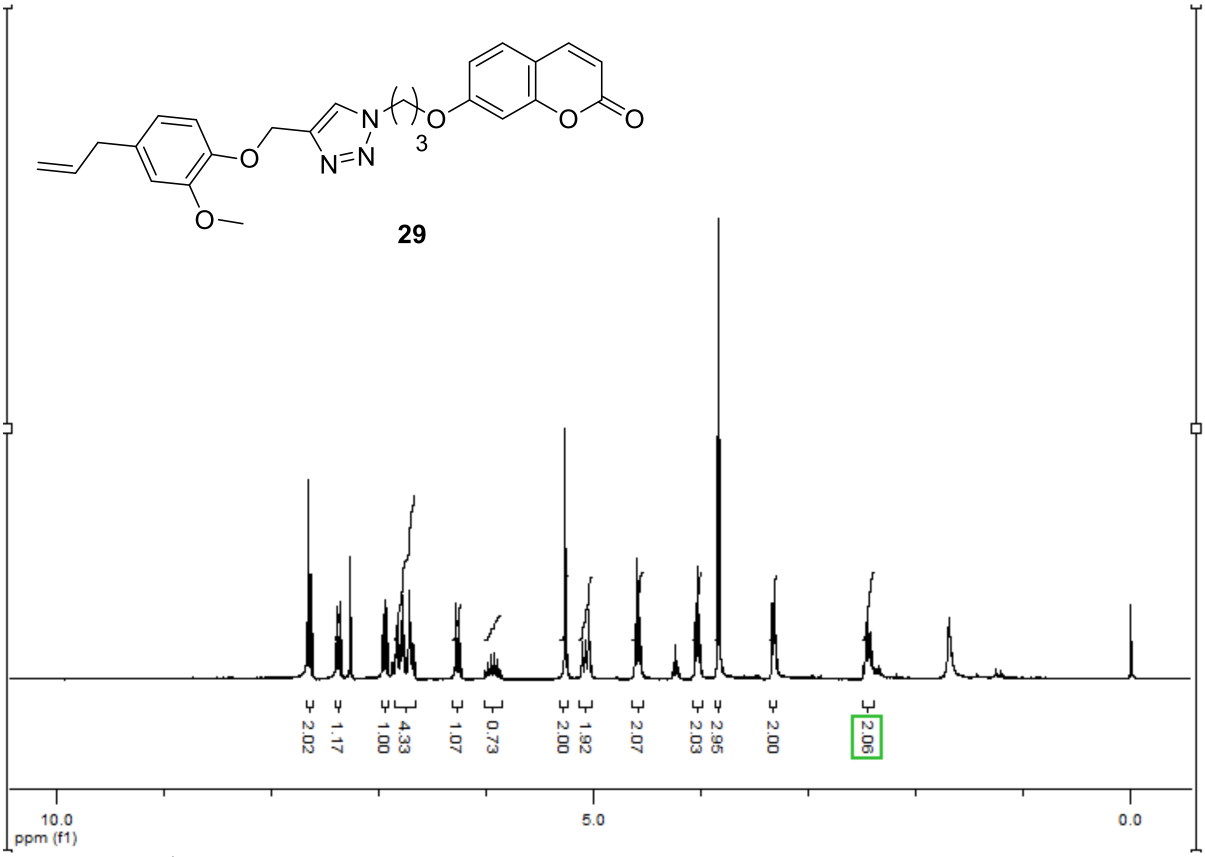

Figure S25. ^{1}H NMR (300 MHz, $CDCl_3$) spectrum of **29**

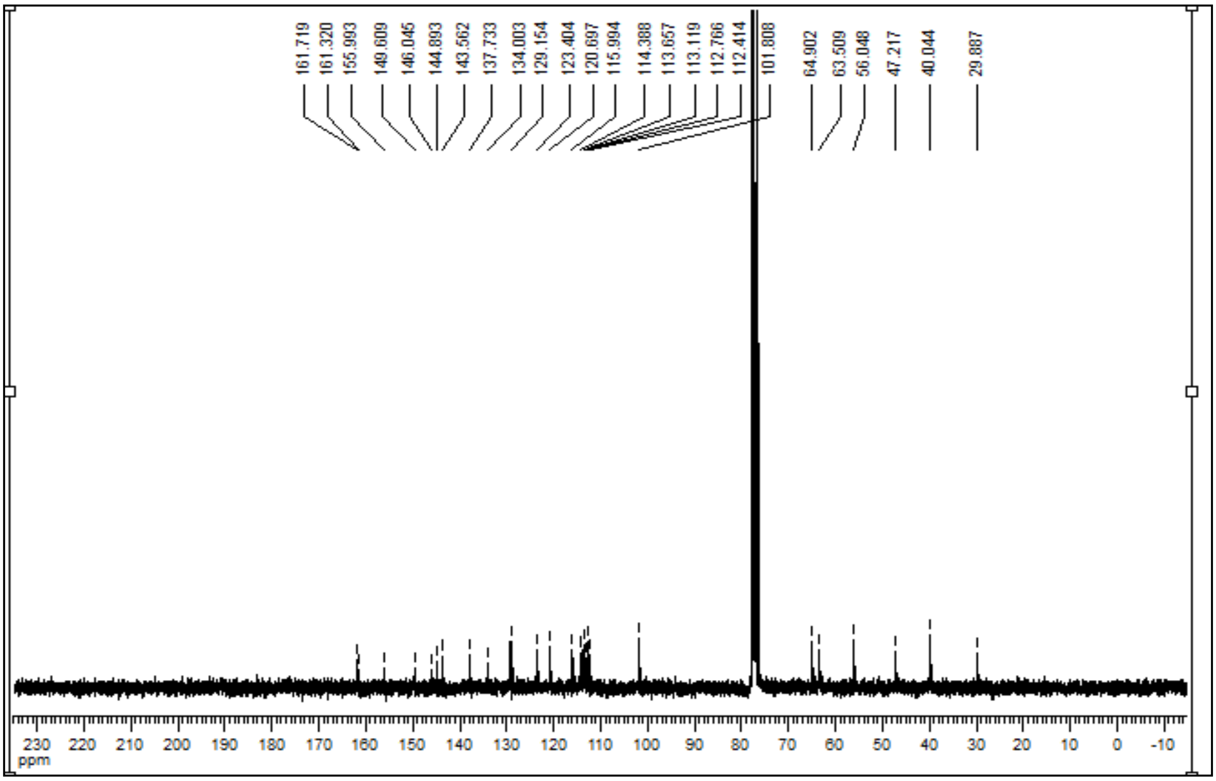

Figure S26. ^{13}C NMR (75 MHz, $CDCl_3$) spectrum of **29**

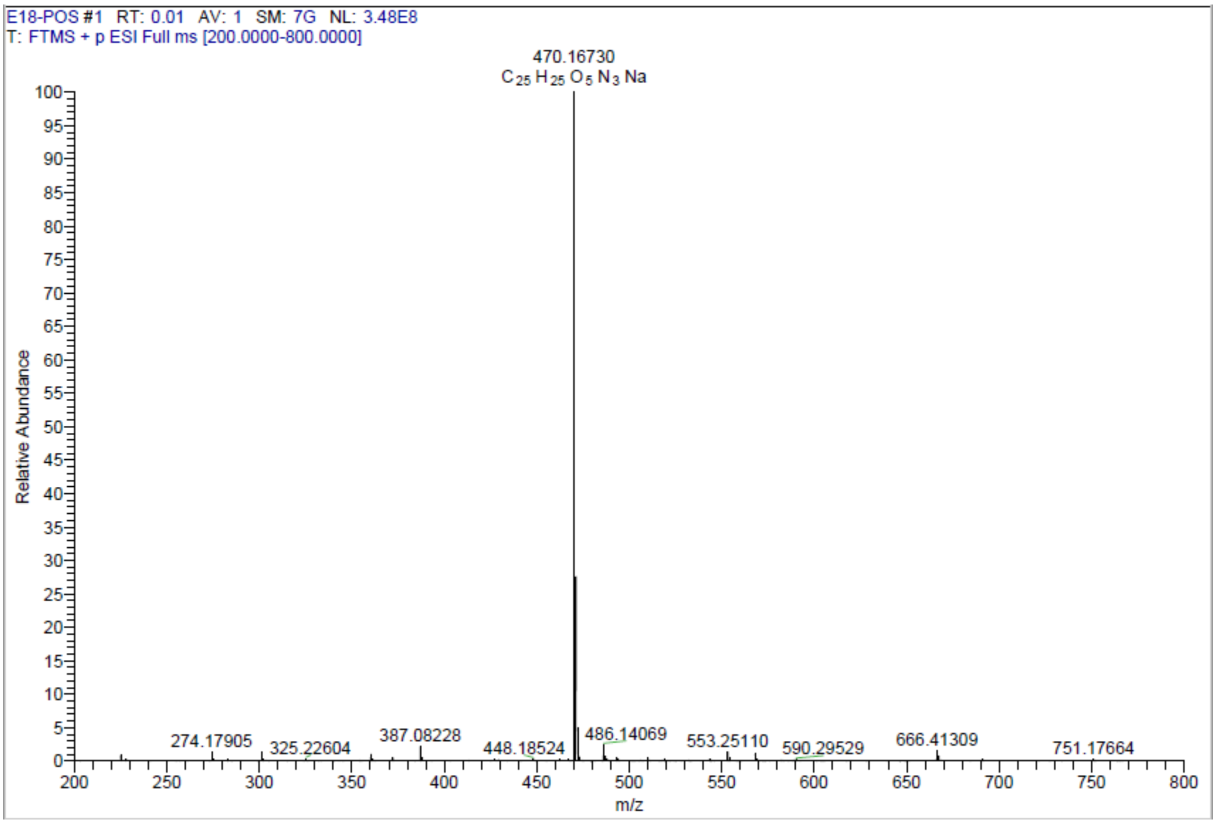

Figure S27. Mass spectrum of **29**

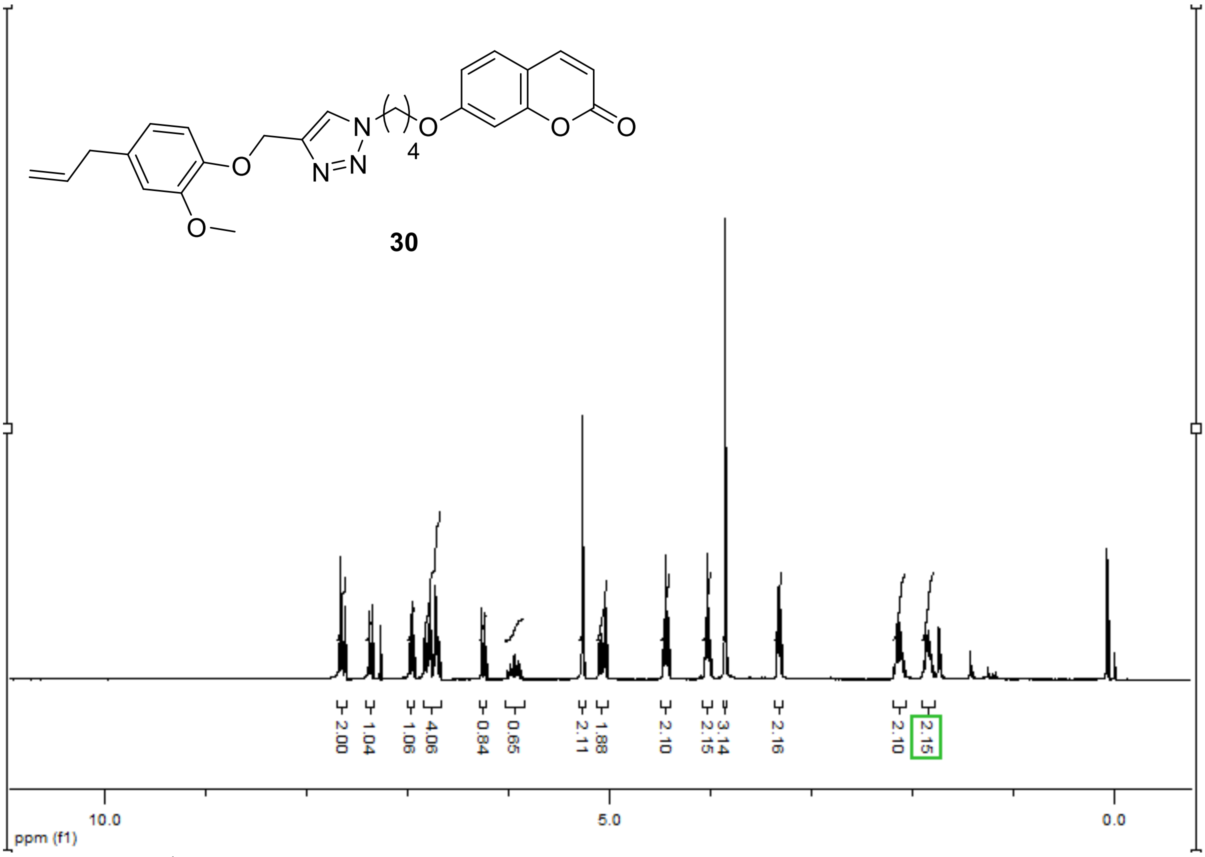

Figure S28. ^{1}H NMR (300 MHz, $CDCl_3$) spectrum of **30**

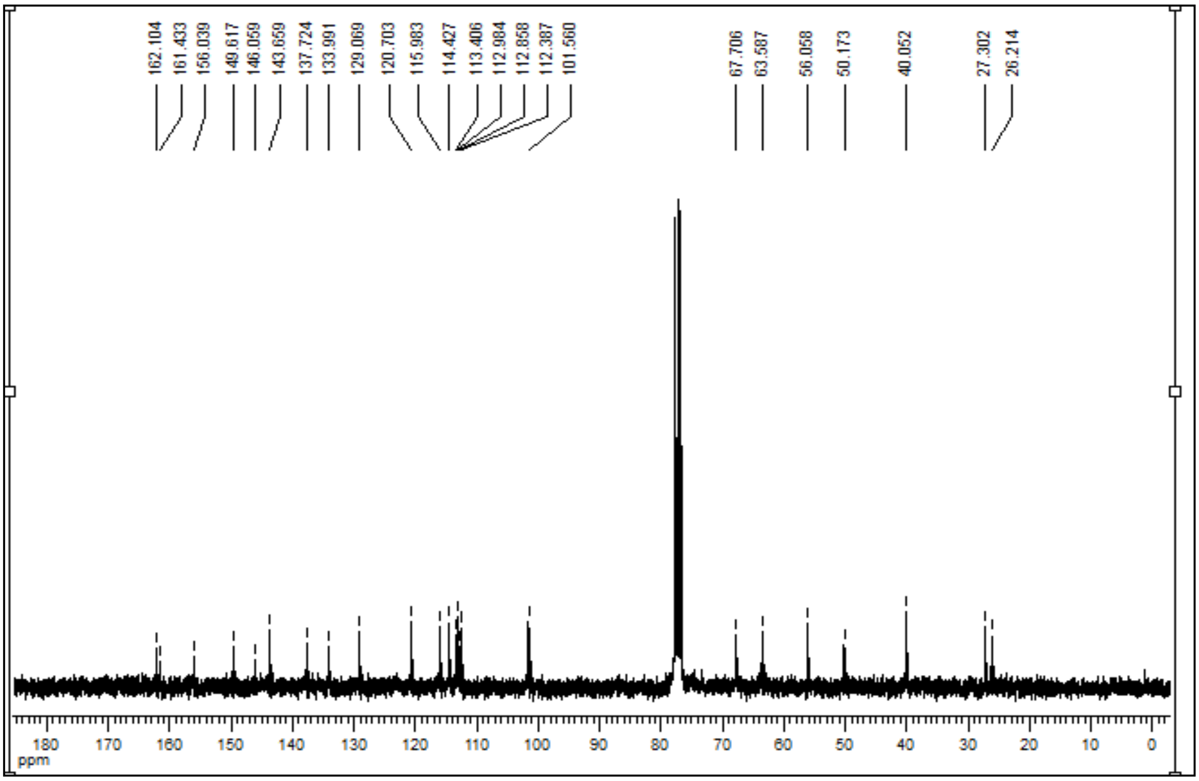

Figure S29. ^{13}C NMR (75 MHz, $CDCl_3$) spectrum of **30**

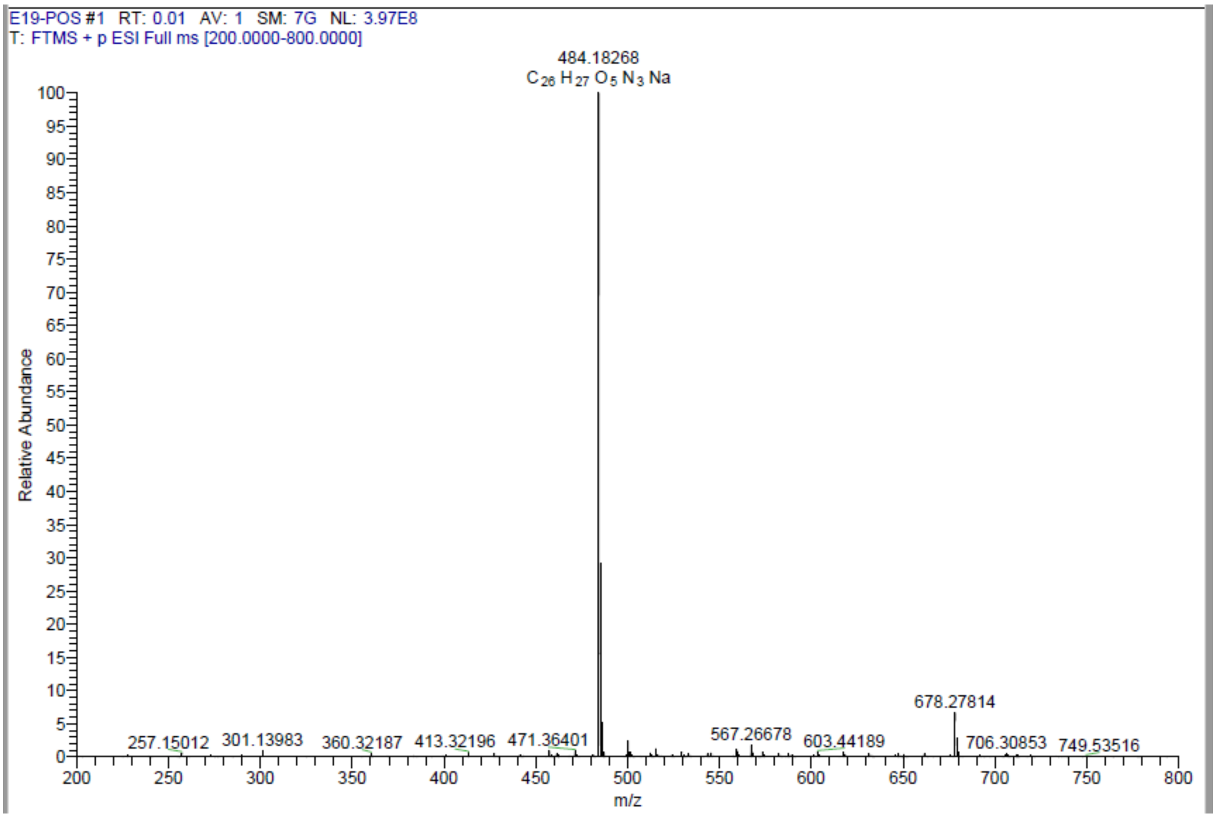

Figure S30. Mass spectrum of **30**

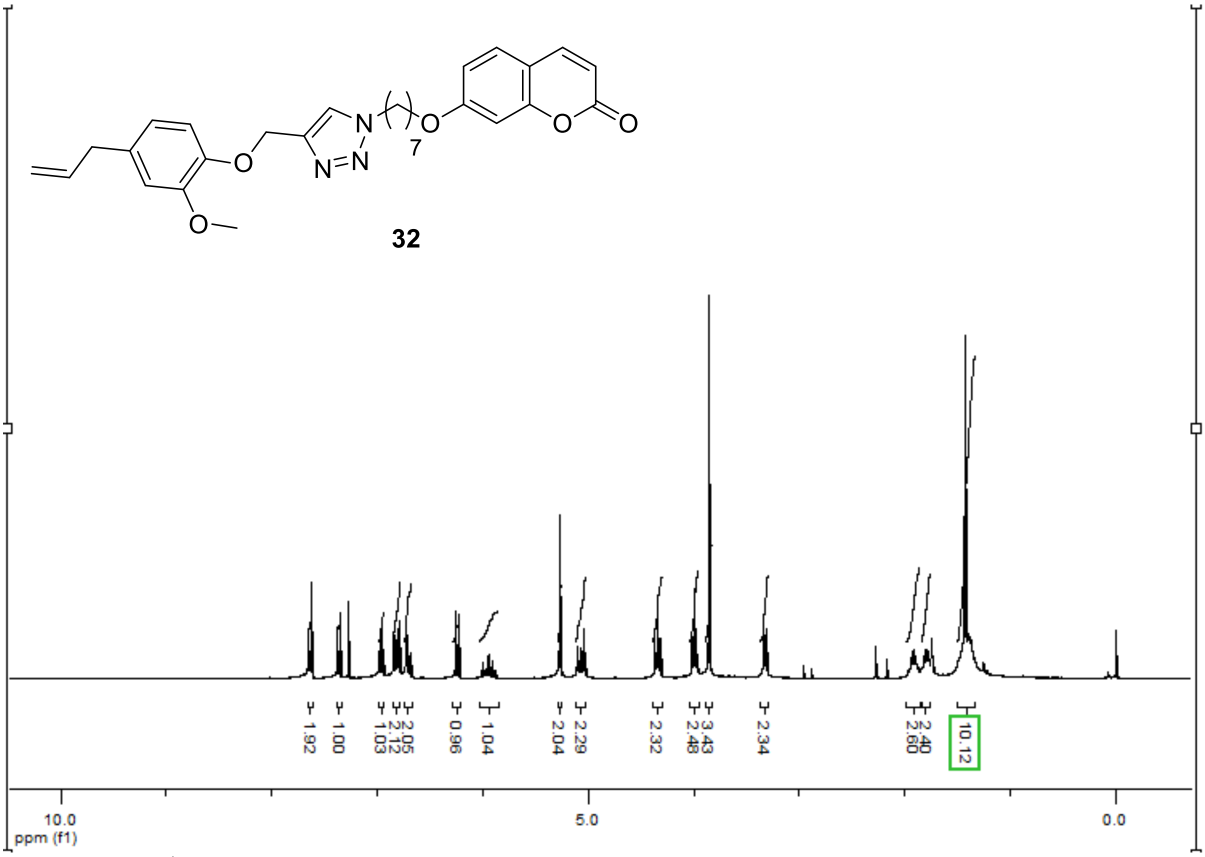

Figure S31. ^{1}H NMR (300 MHz, $CDCl_3$) spectrum of **32**

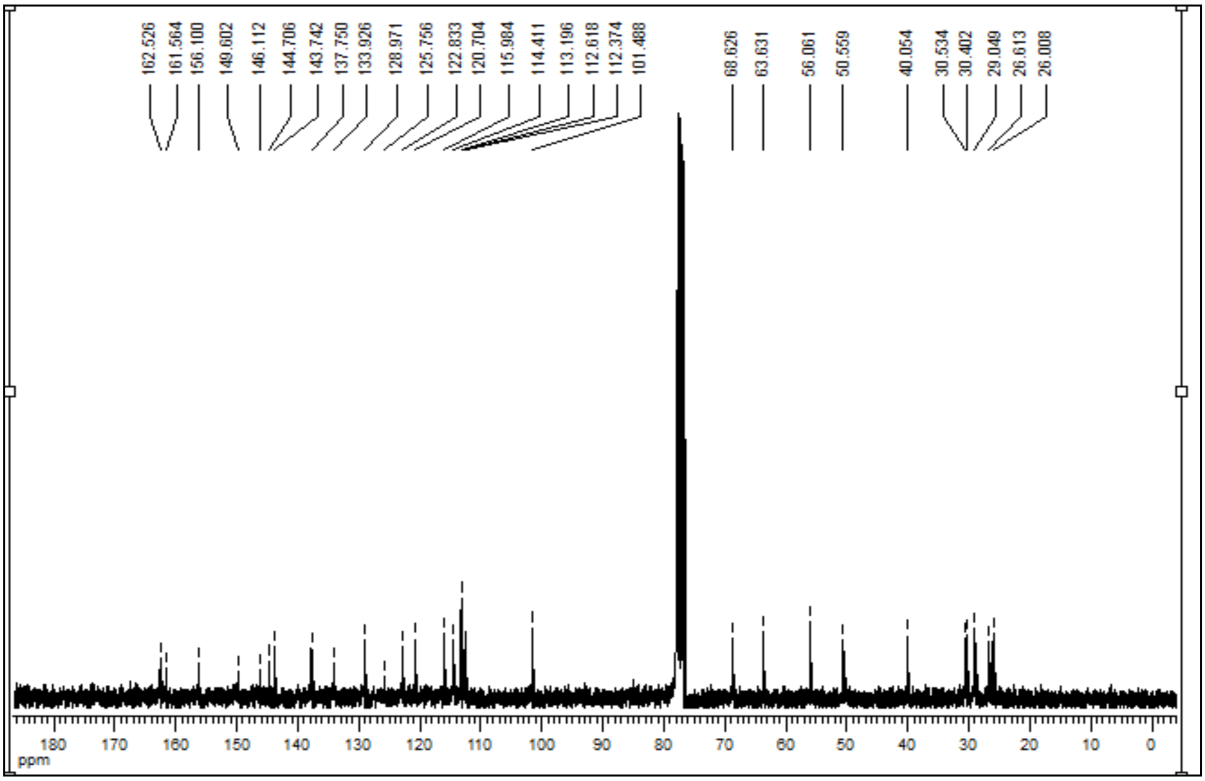

Figure S32. ^{13}C NMR (75 MHz, $CDCl_3$) spectrum of **32**

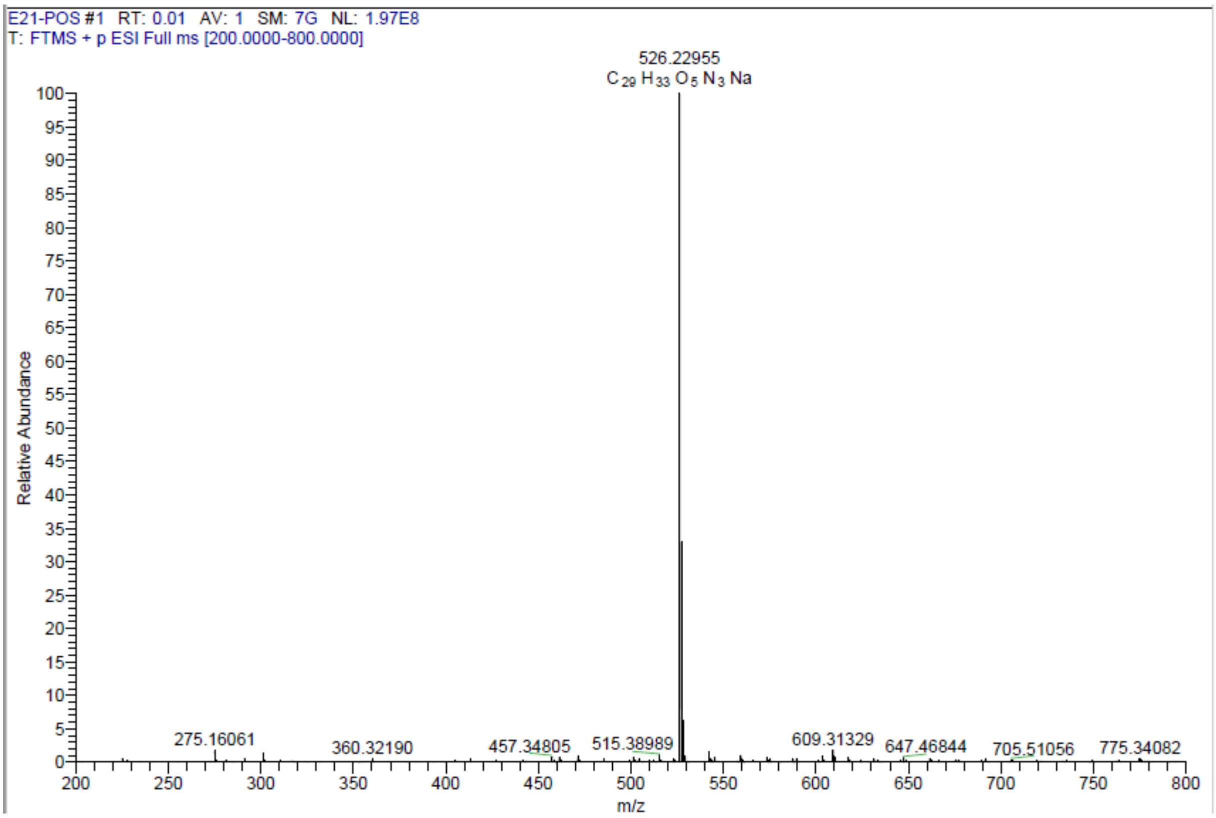

Figure S33. Mass spectrum of **32**

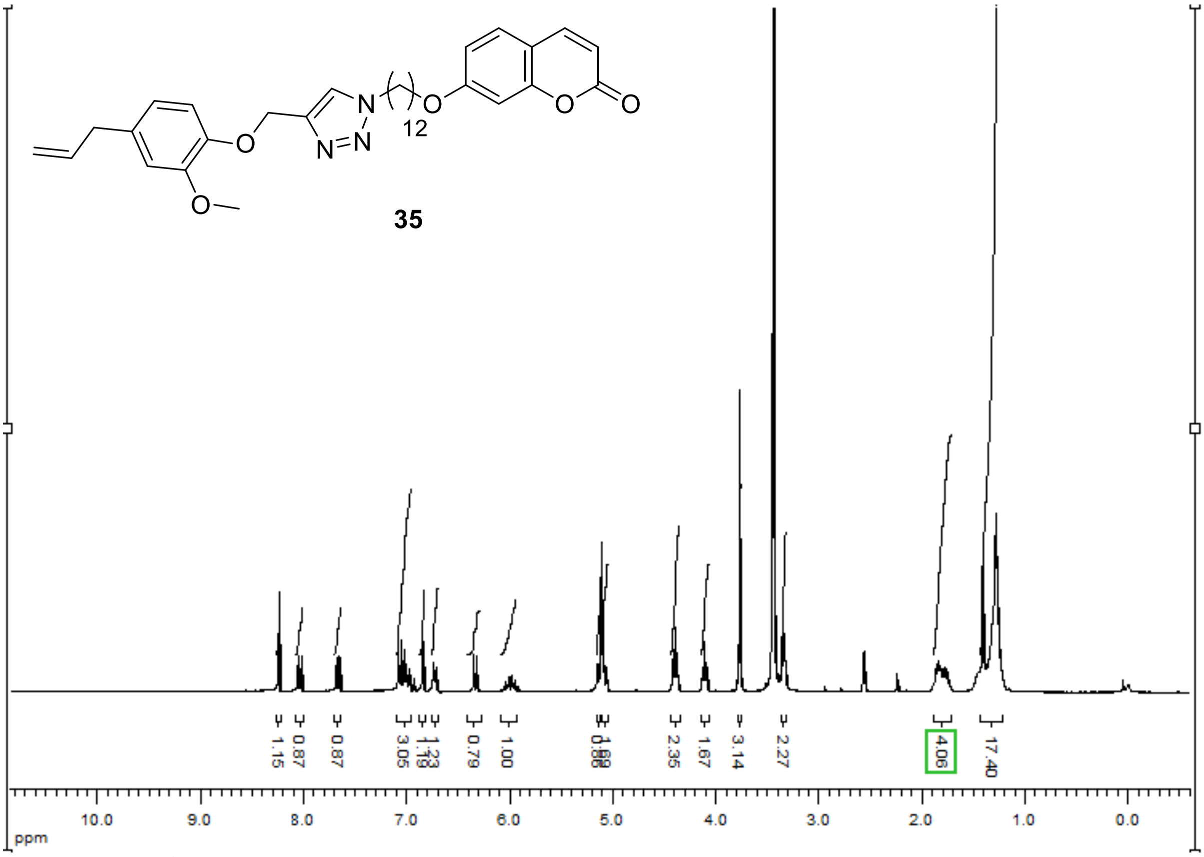

Figure S34. ^{1}H NMR (300 MHz, DMSO-d_6) spectrum of **35**

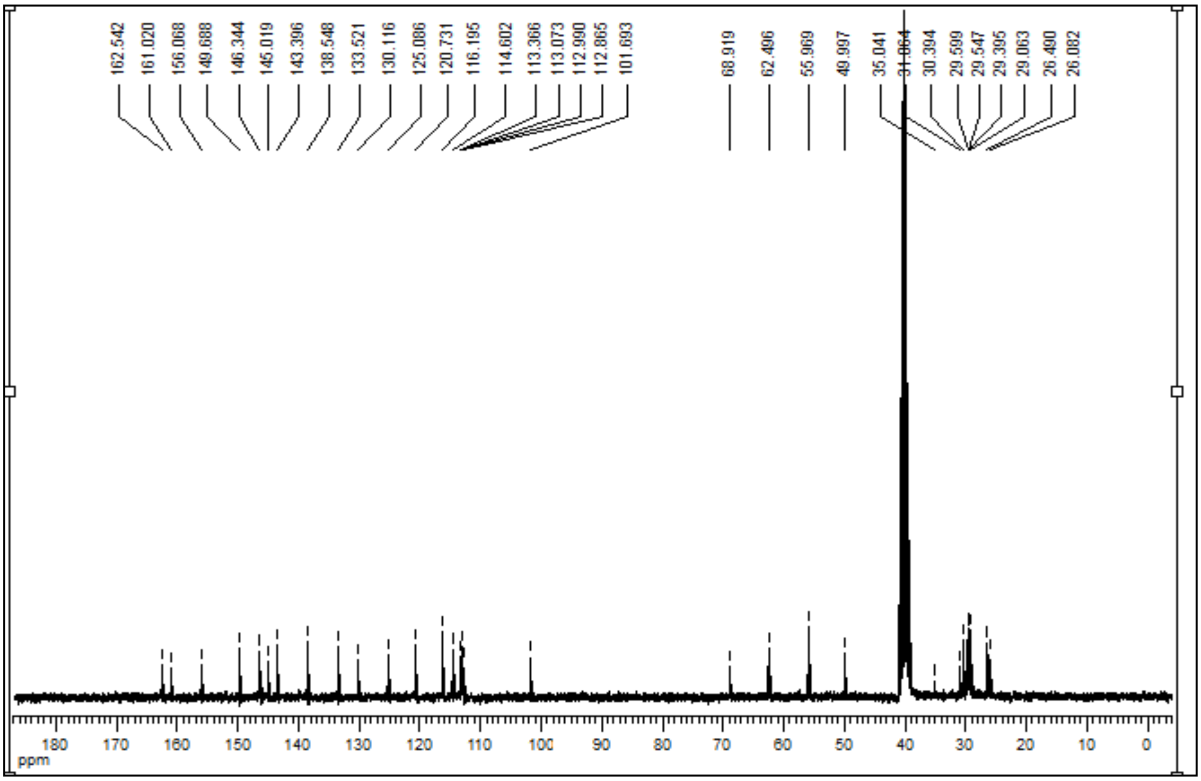

Figure S35. ^{13}C NMR (75 MHz, DMSO-d_6) spectrum of **35**

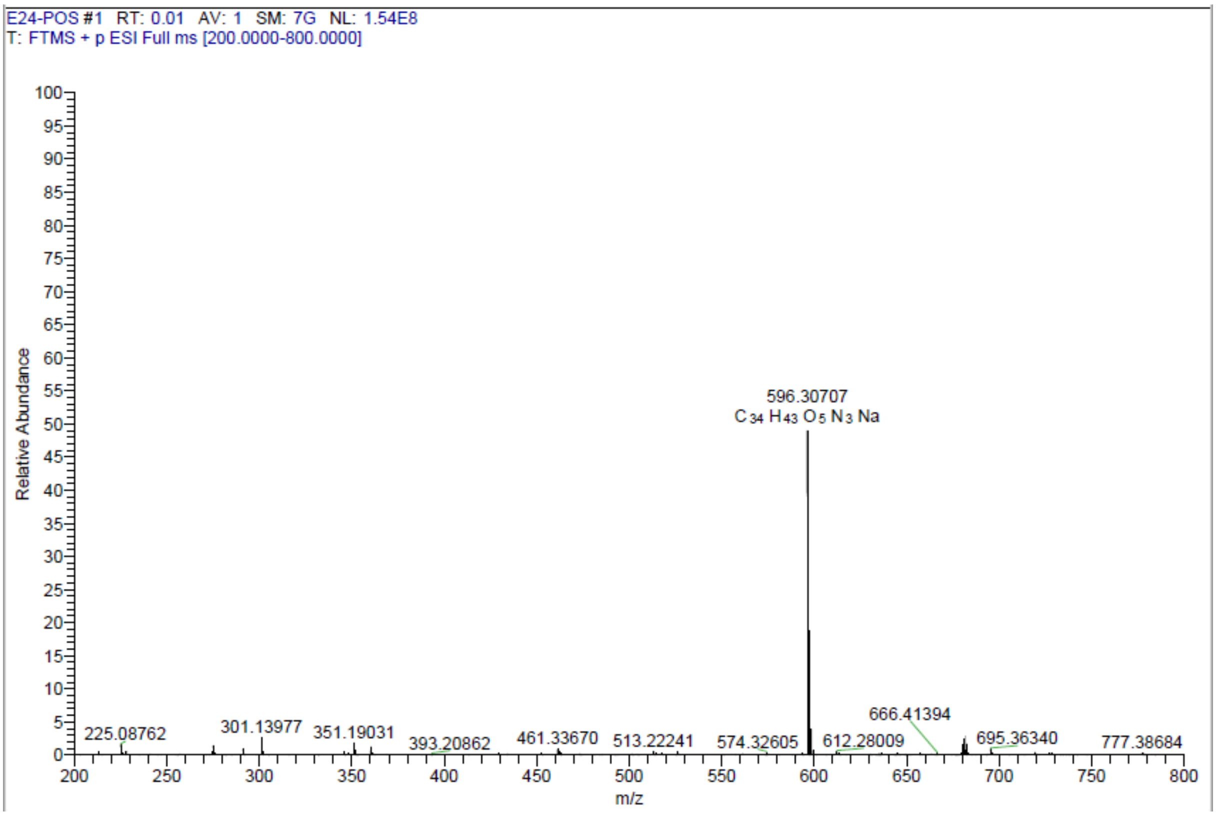

Figure S36. Mass spectrum of **35**

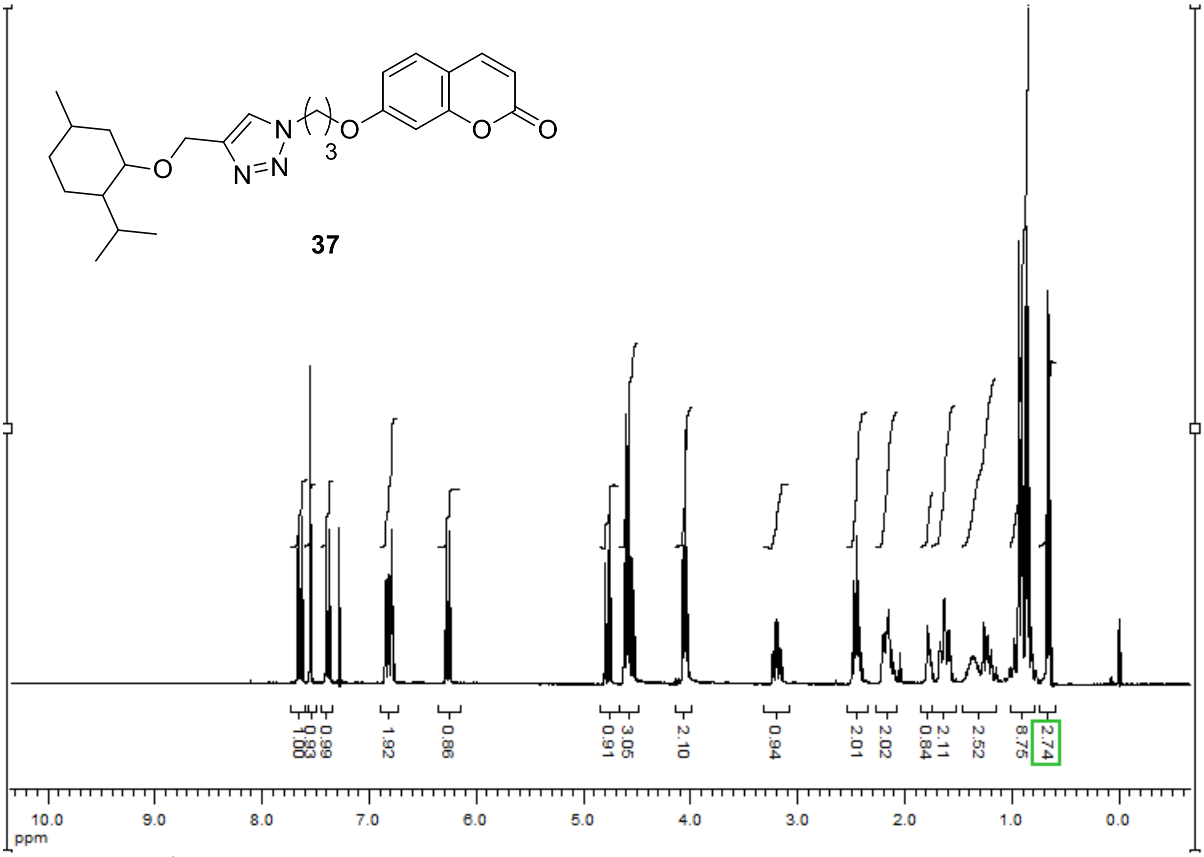

Figure S37. ^{1}H NMR (300 MHz, $CDCl_3$) spectrum of **37**

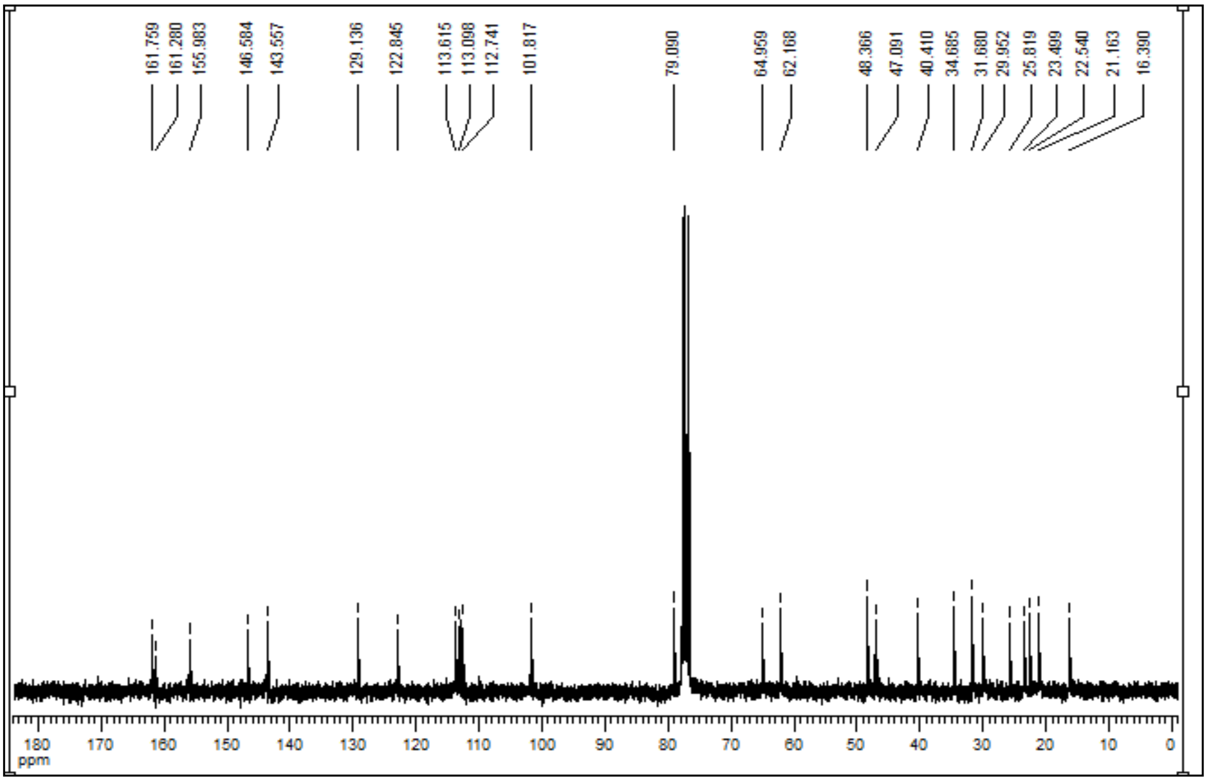

Figure S38. ^{13}C NMR (75 MHz, $CDCl_3$) spectrum of **37**

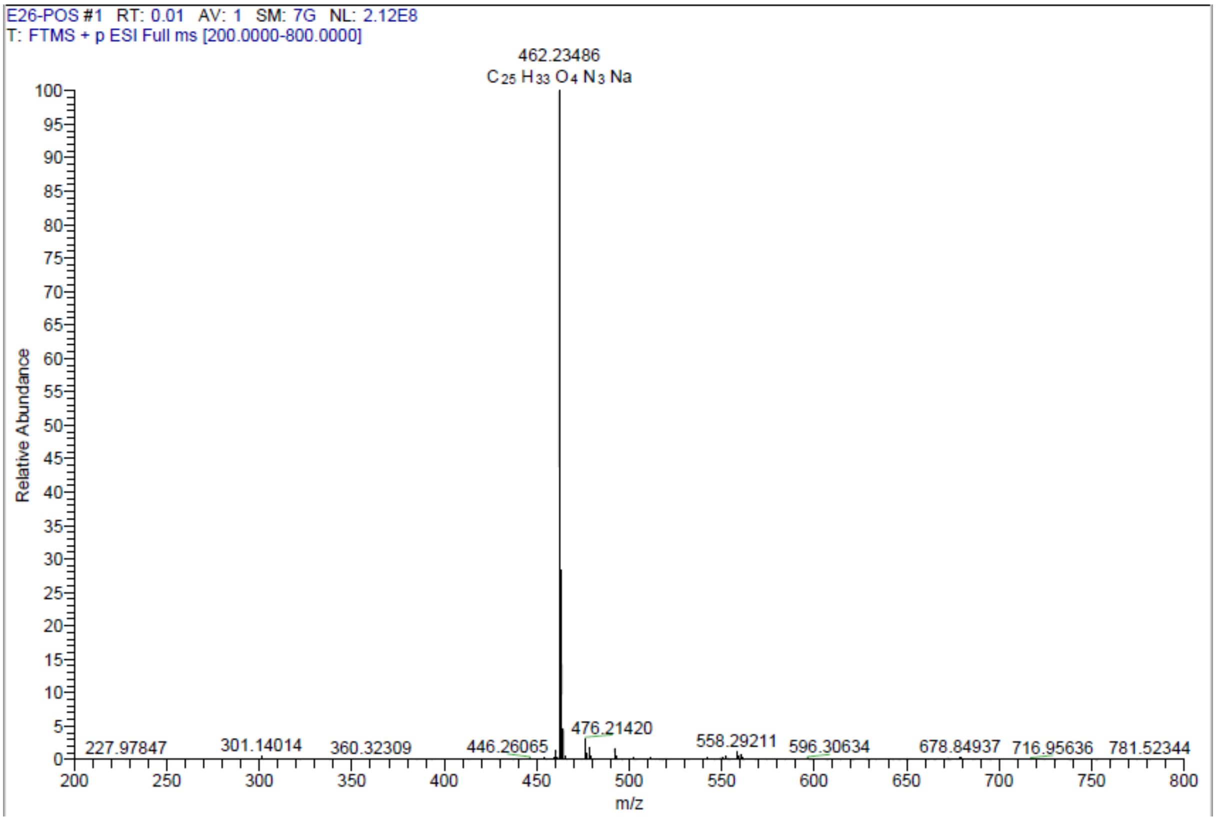

Figure S39. Mass spectrum of **37**

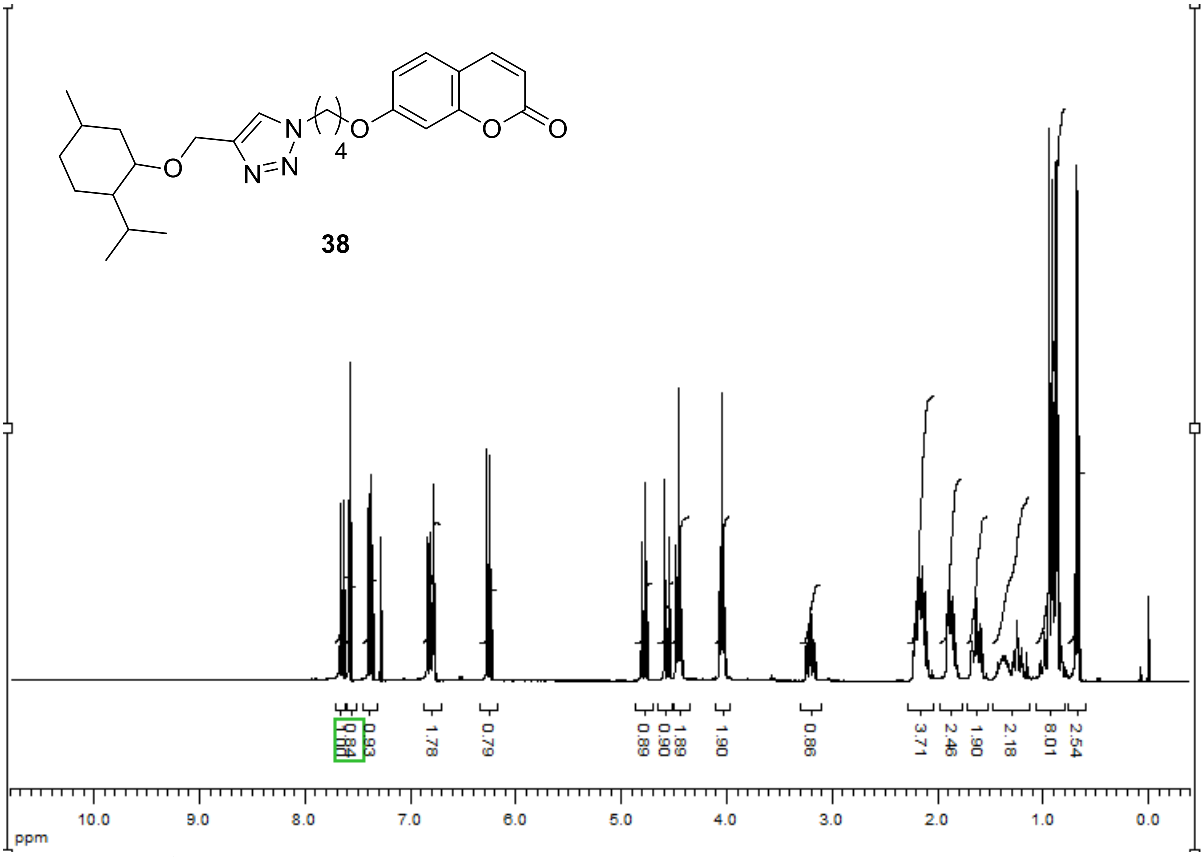

Figure S40. ^{1}H NMR (300 MHz, $CDCl_3$) spectrum of **38**

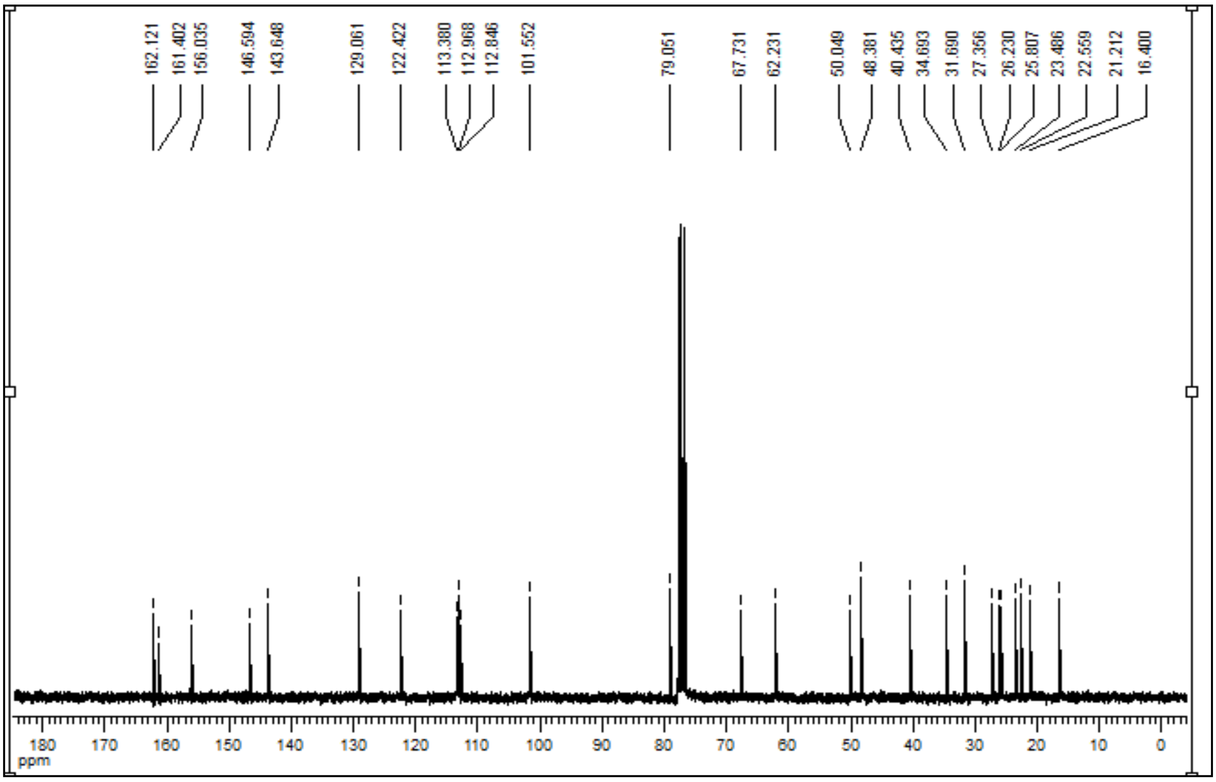

Figure S41. ^{13}C NMR (75 MHz, $CDCl_3$) spectrum of **38**

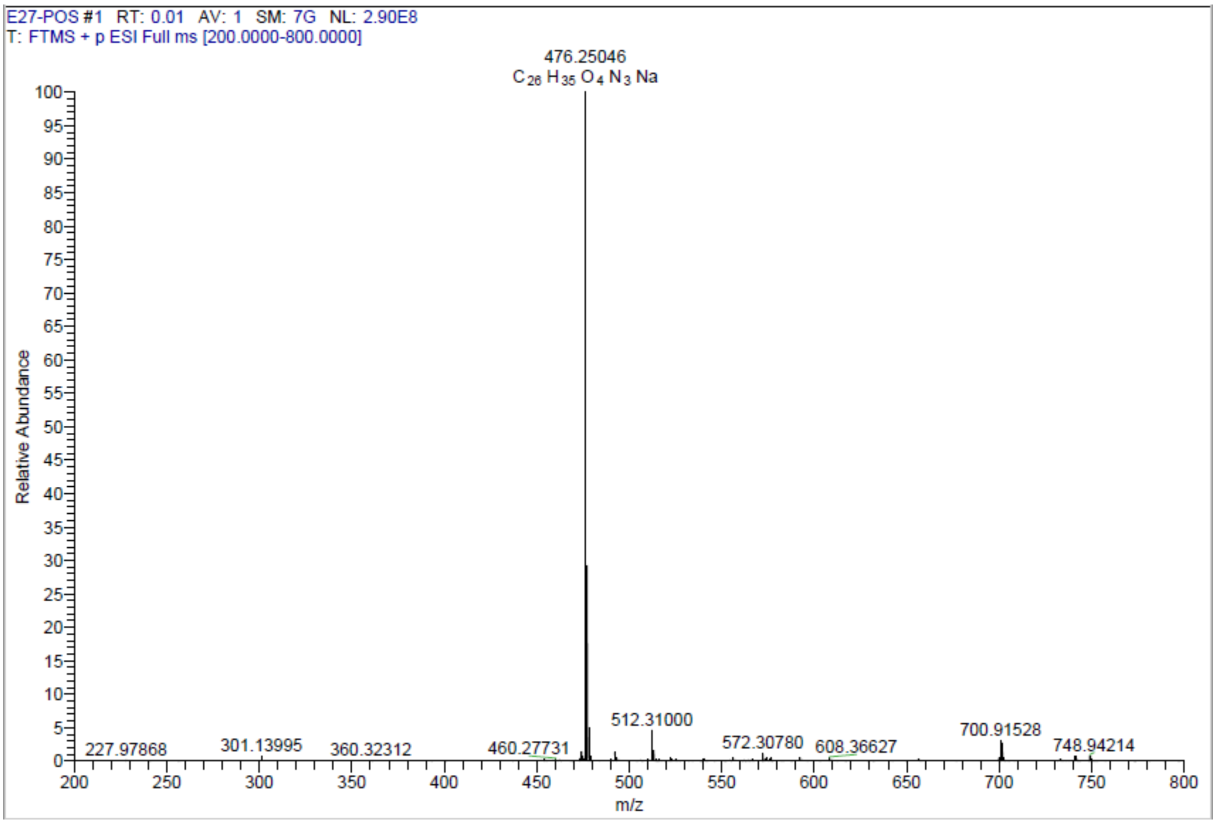

Figure S42. Mass spectrum of **38**

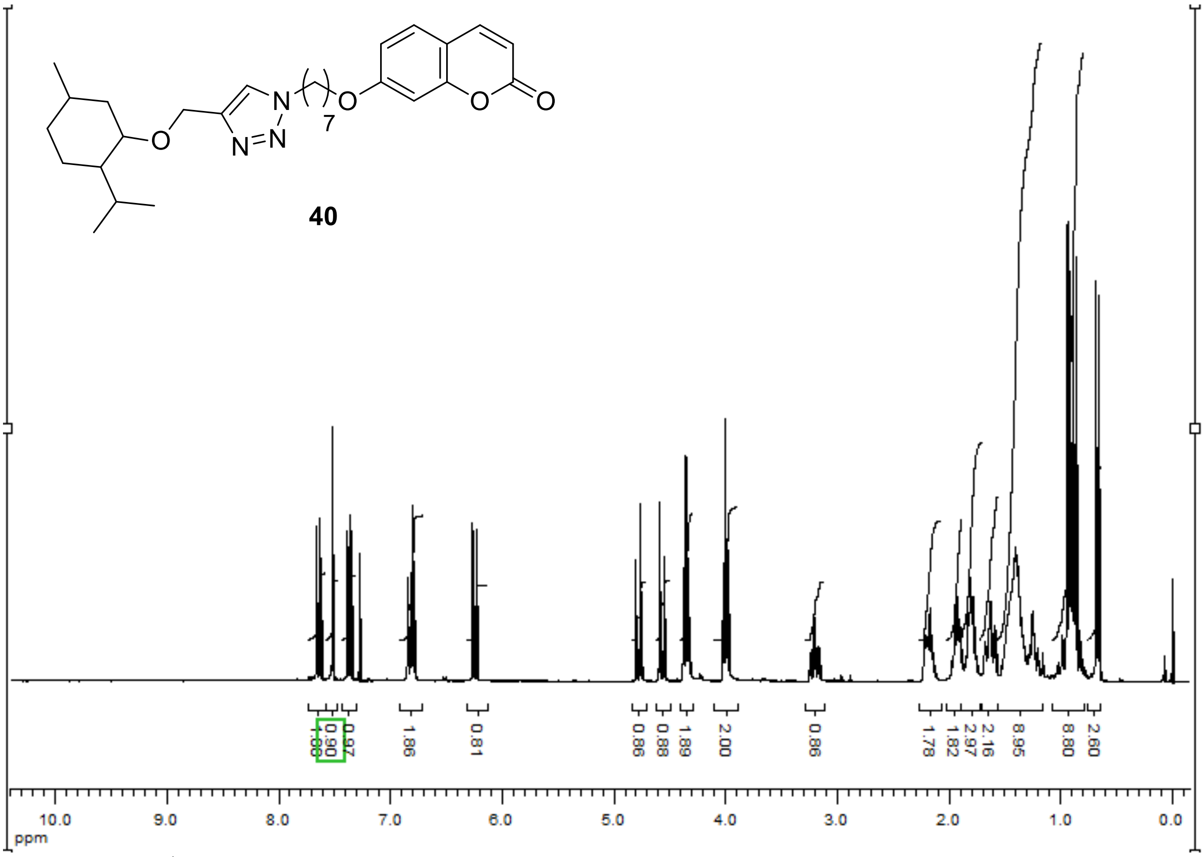

Figure S43. ^{1}H NMR (300 MHz, $CDCl_3$) spectrum of **40**

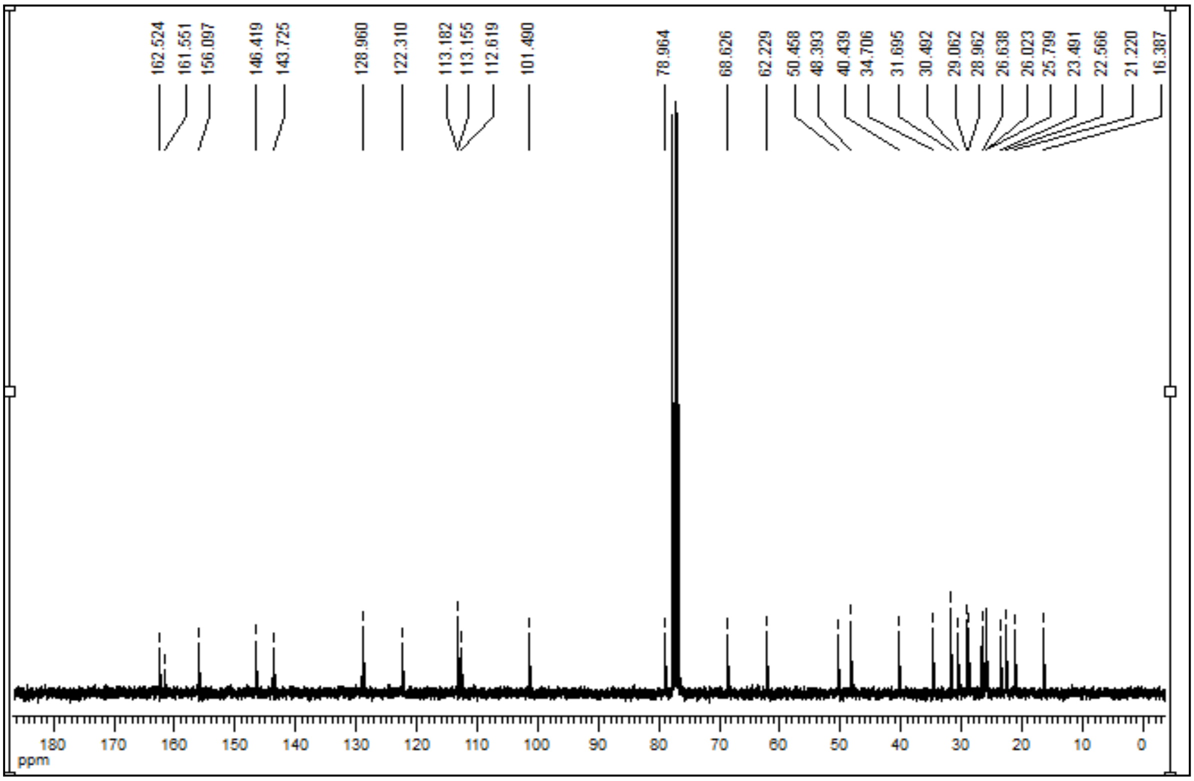

Figure S44. ^{13}C NMR (75 MHz, $CDCl_3$) spectrum of **40**

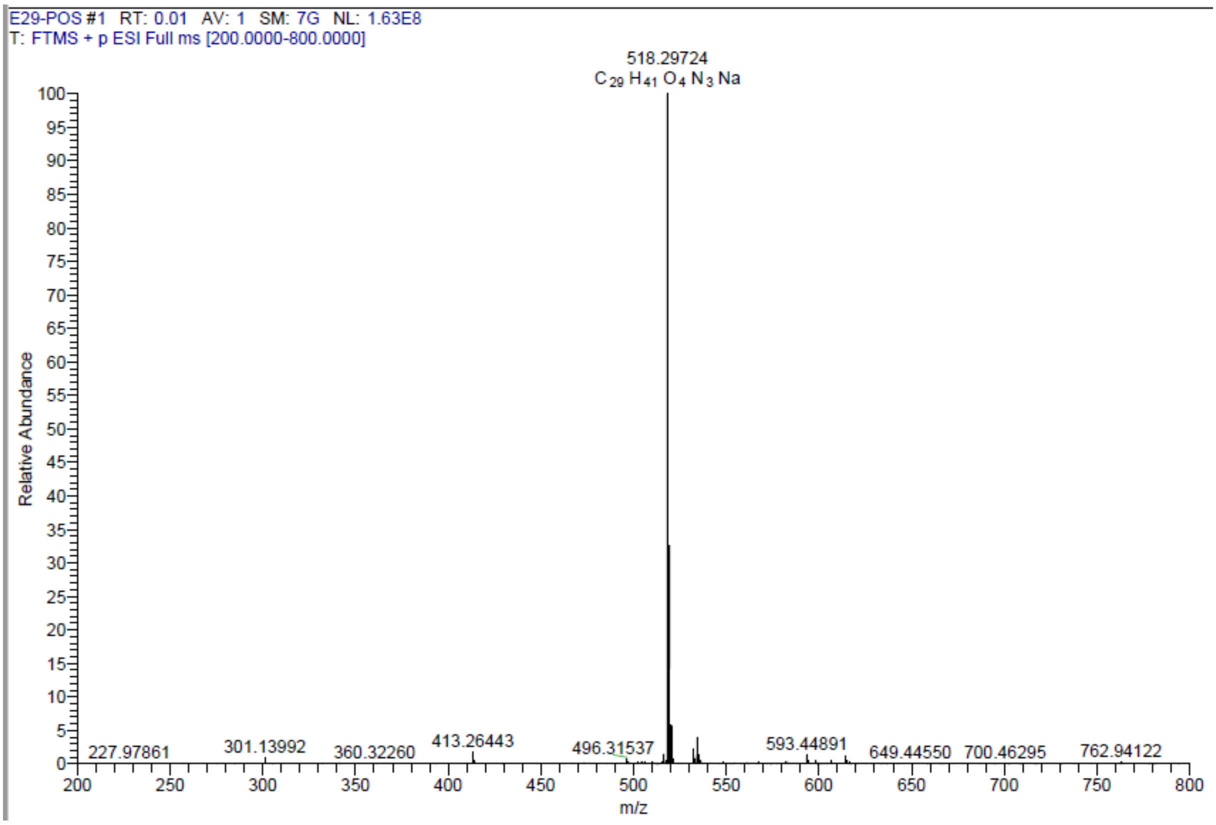

Figure S45. Mass spectrum of **40**

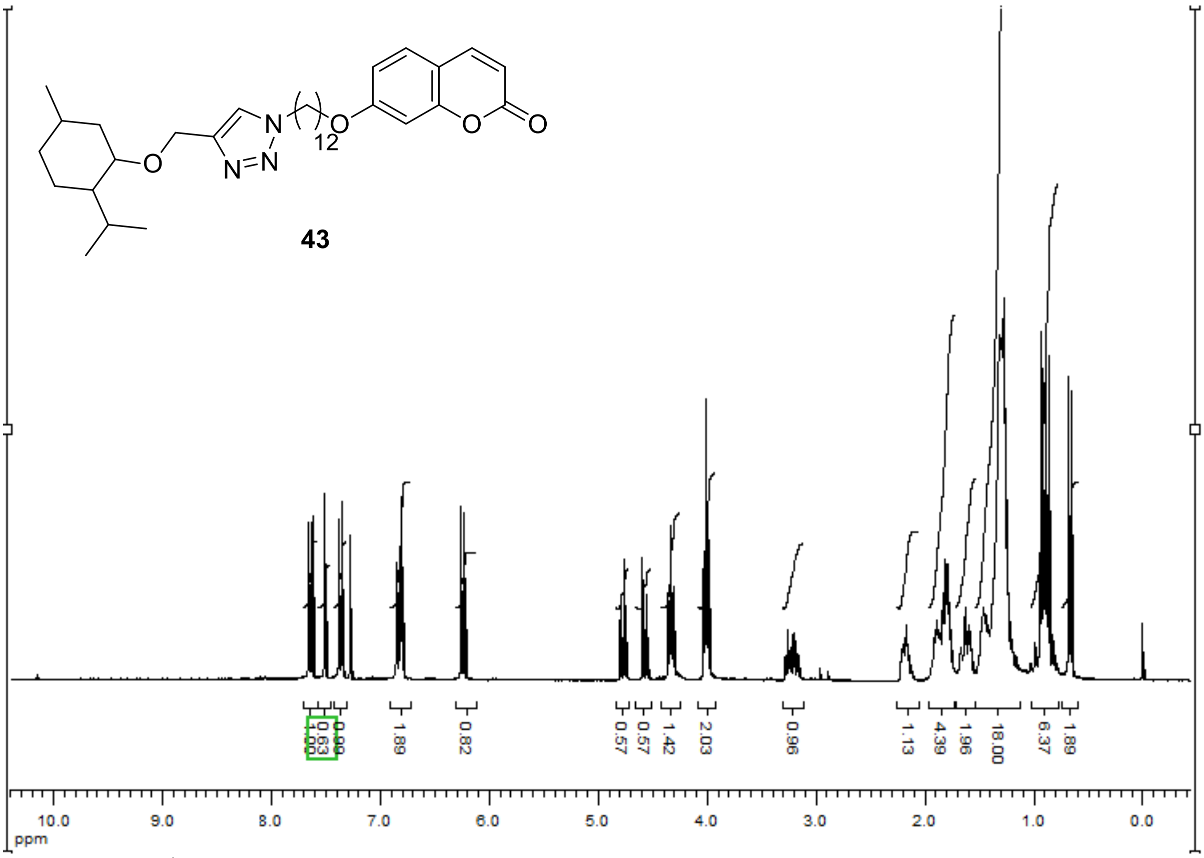

Figure S46. ^{1}H NMR (300 MHz, $CDCl_3$) spectrum of **43**

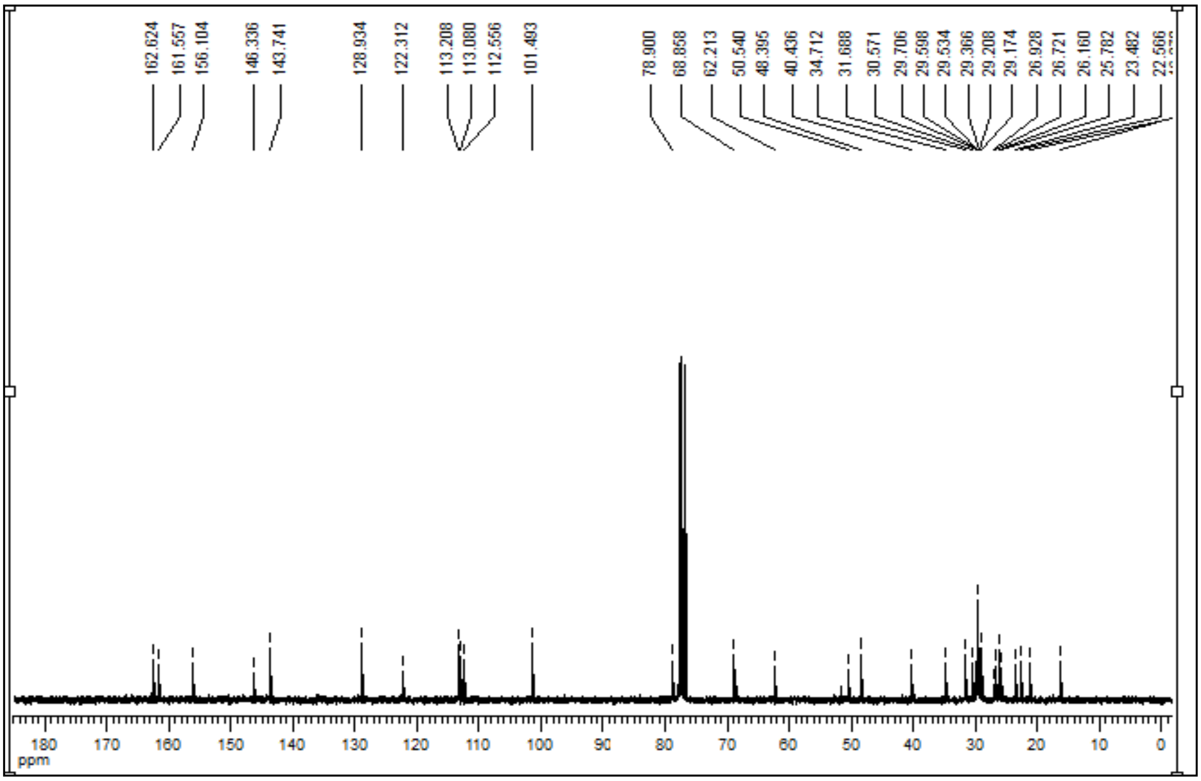

Figure S47. ^{13}C NMR (75 MHz, $CDCl_3$) spectrum of **43**

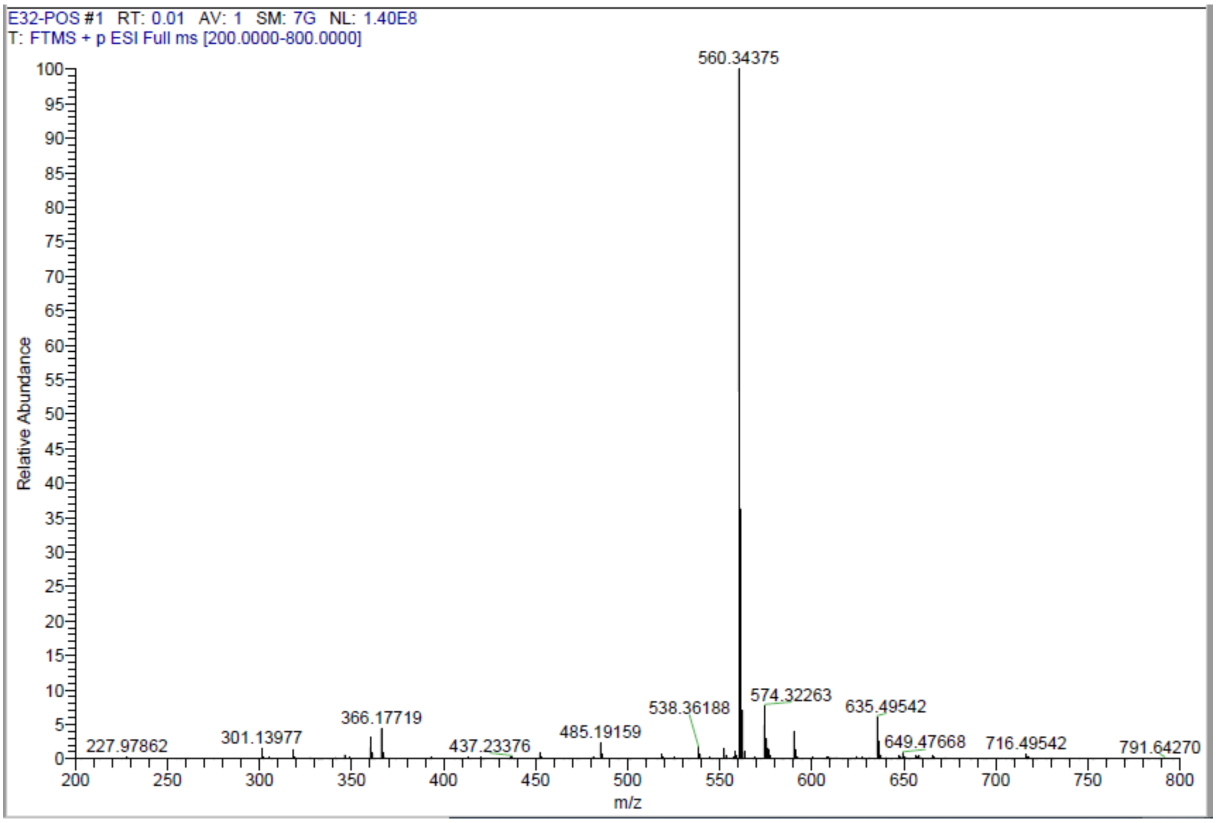

Figure S48. Mass spectrum of **43**

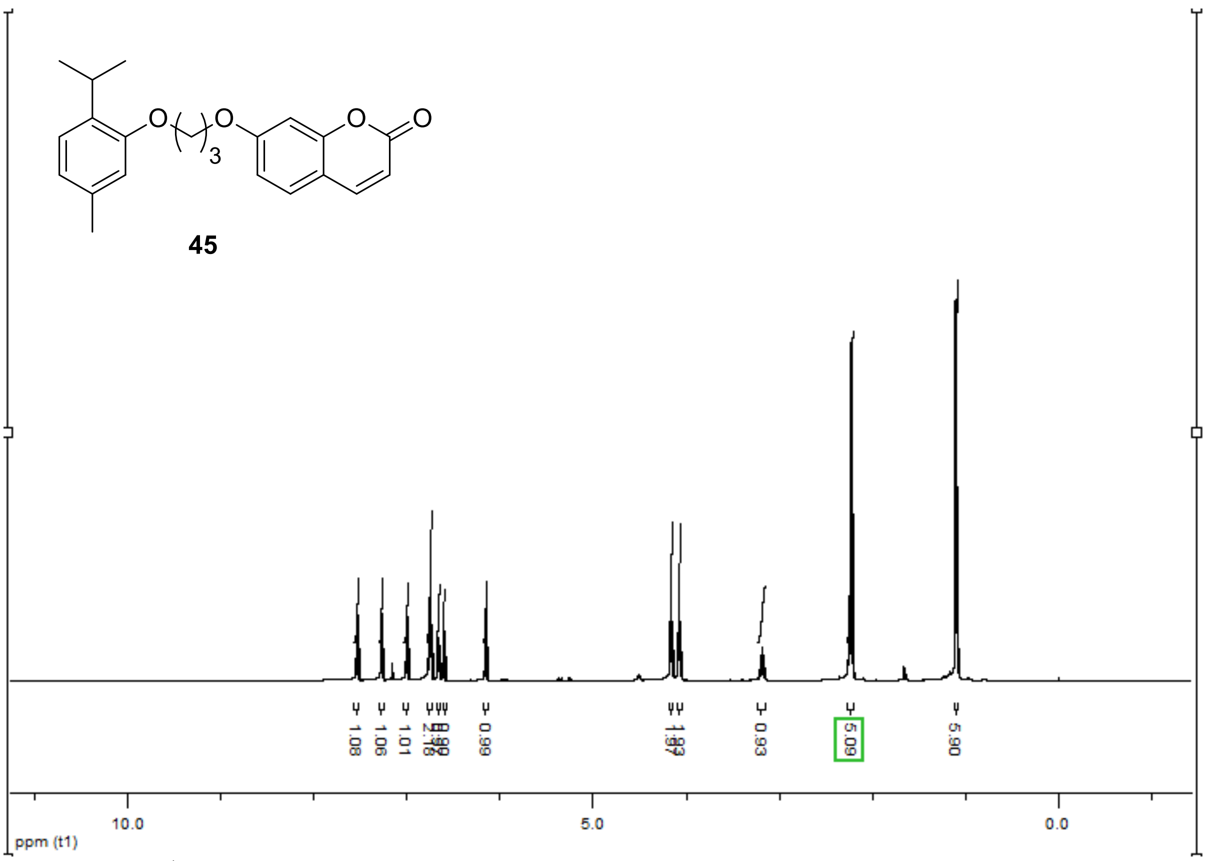

Figure S49. ^{1}H NMR (300 MHz, $CDCl_3$) spectrum of **45**

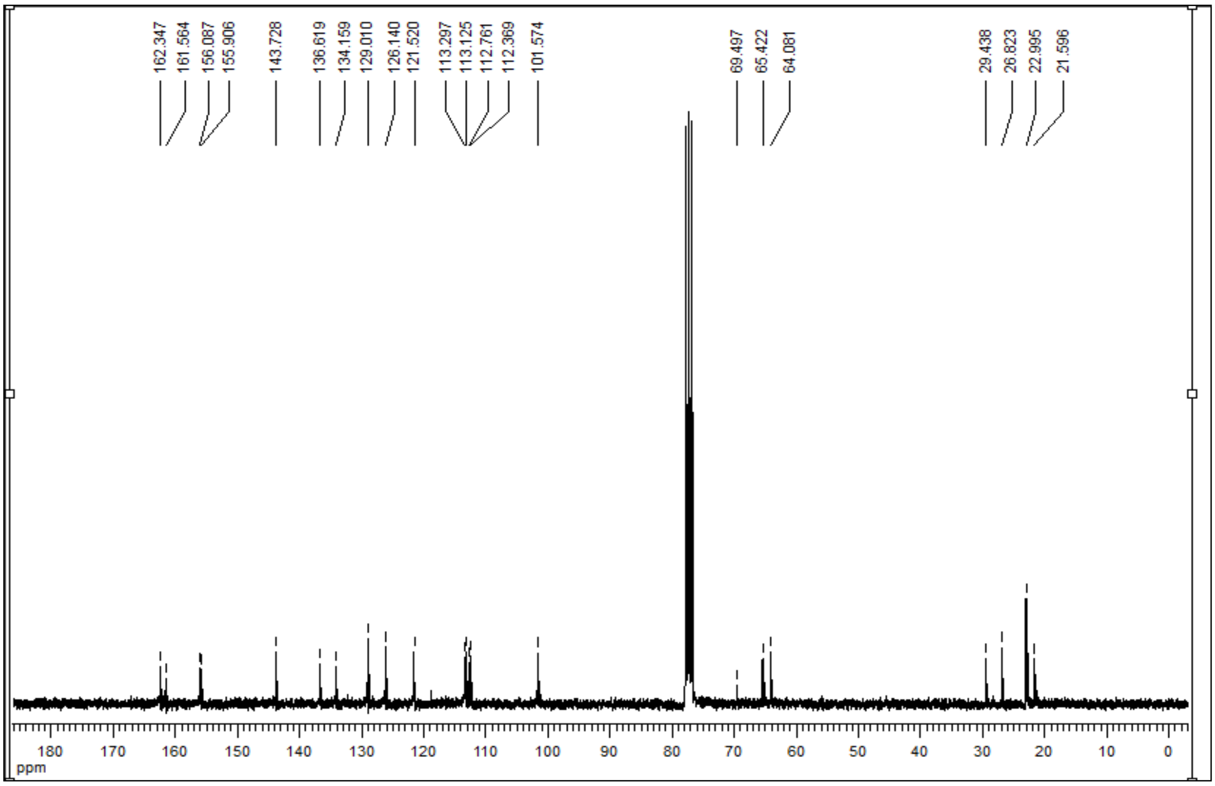

Figure S50. ^{13}C NMR (75 MHz, $CDCl_3$) spectrum of **45**

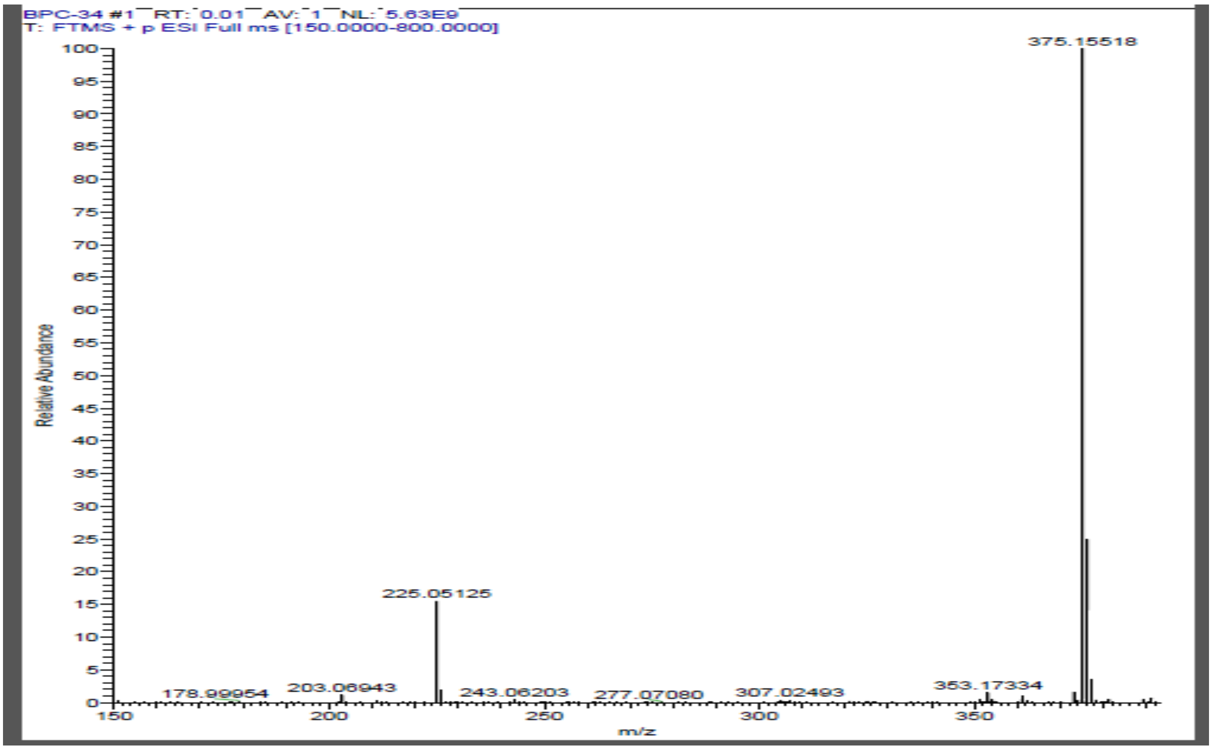

Figure S51. Mass spectrum of **45**

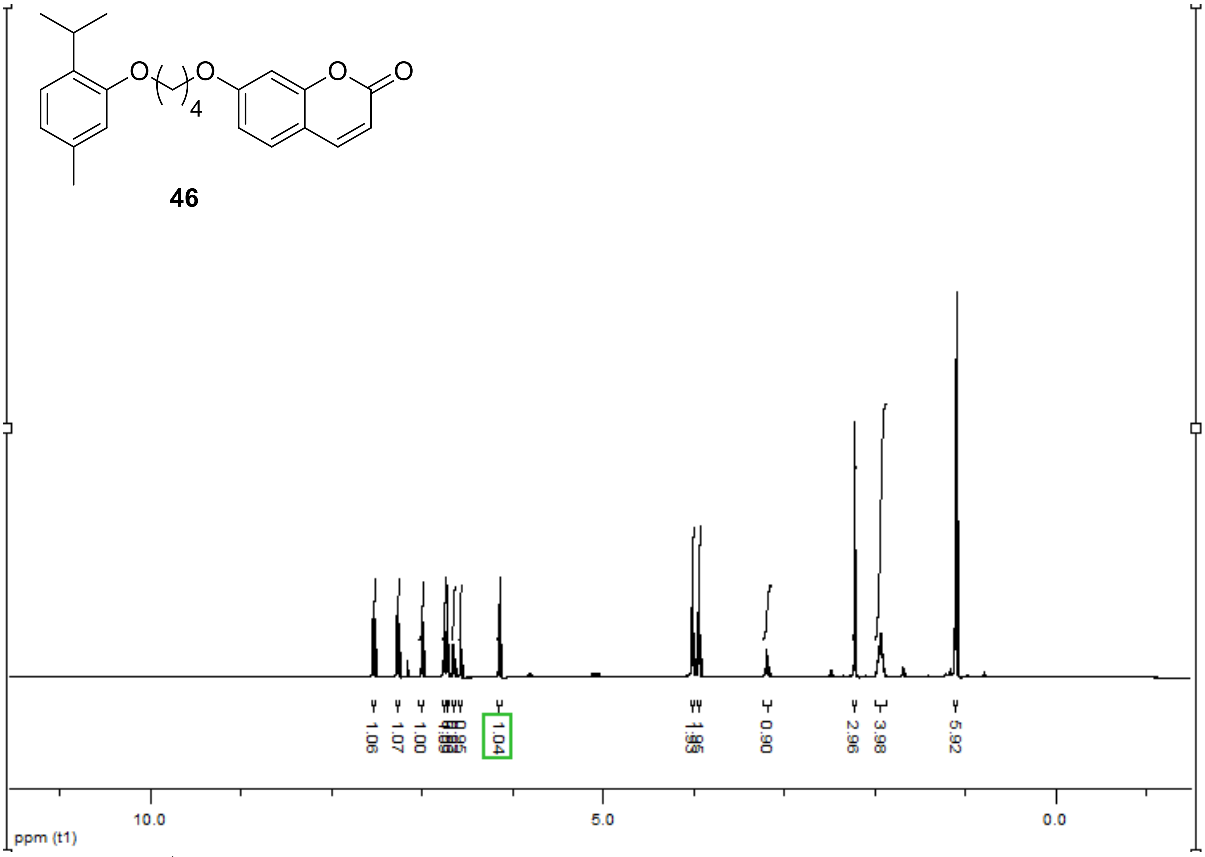

Figure S52. ^{1}H NMR (300 MHz, $CDCl_3$) spectrum of **46**

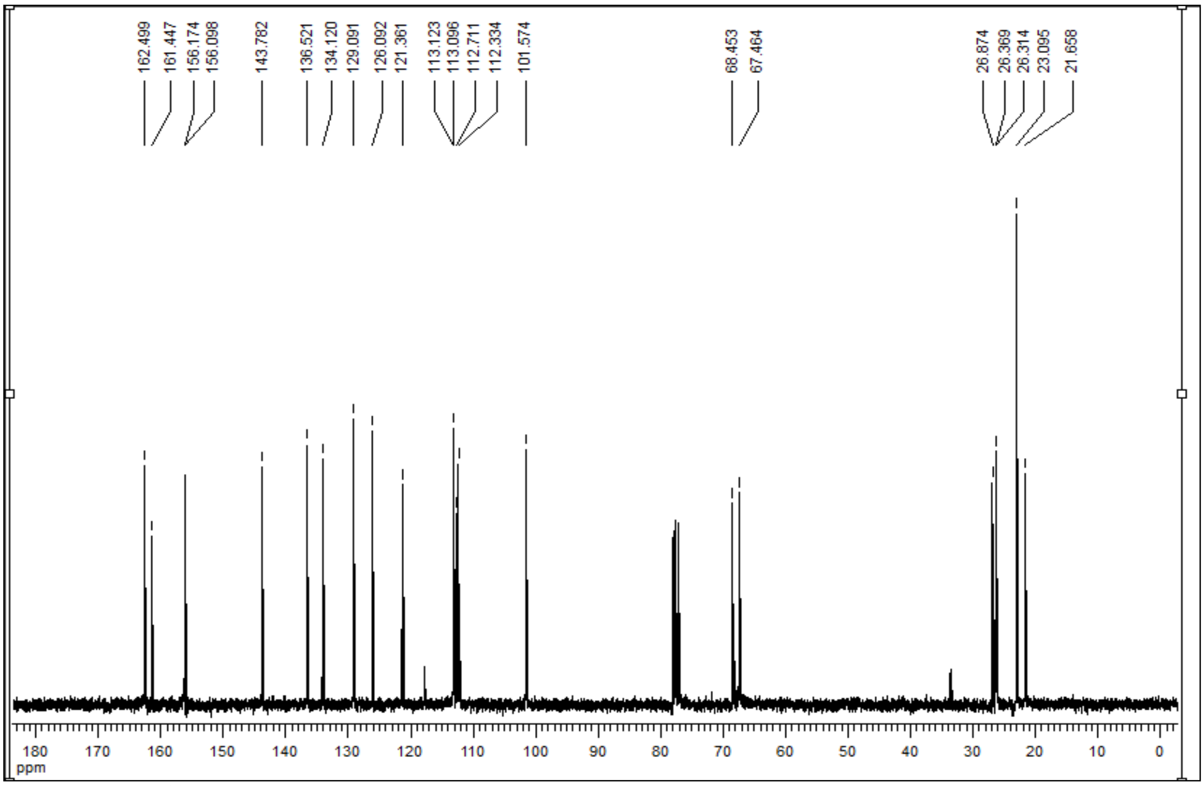

Figure S53. ^{13}C NMR (75 MHz, $CDCl_3$) spectrum of **46**

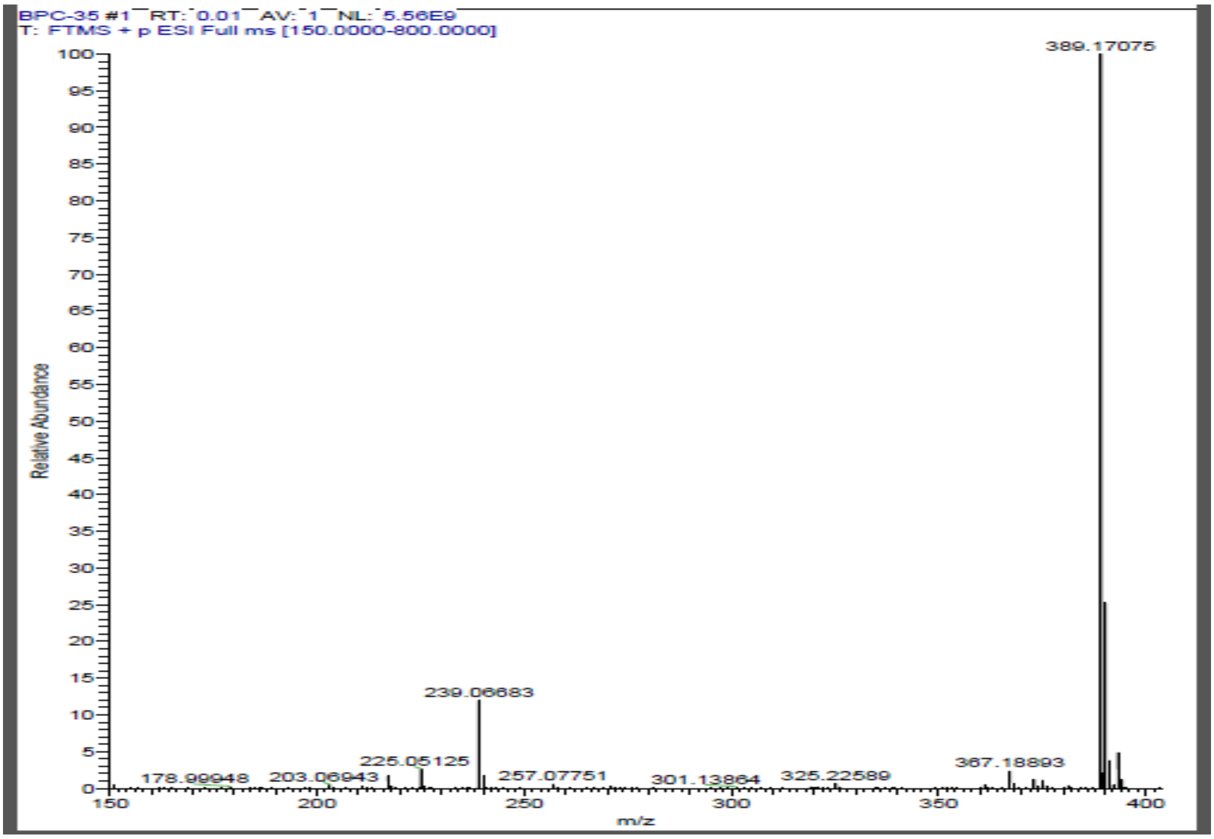

Figure S54. Mass spectrum of **46**

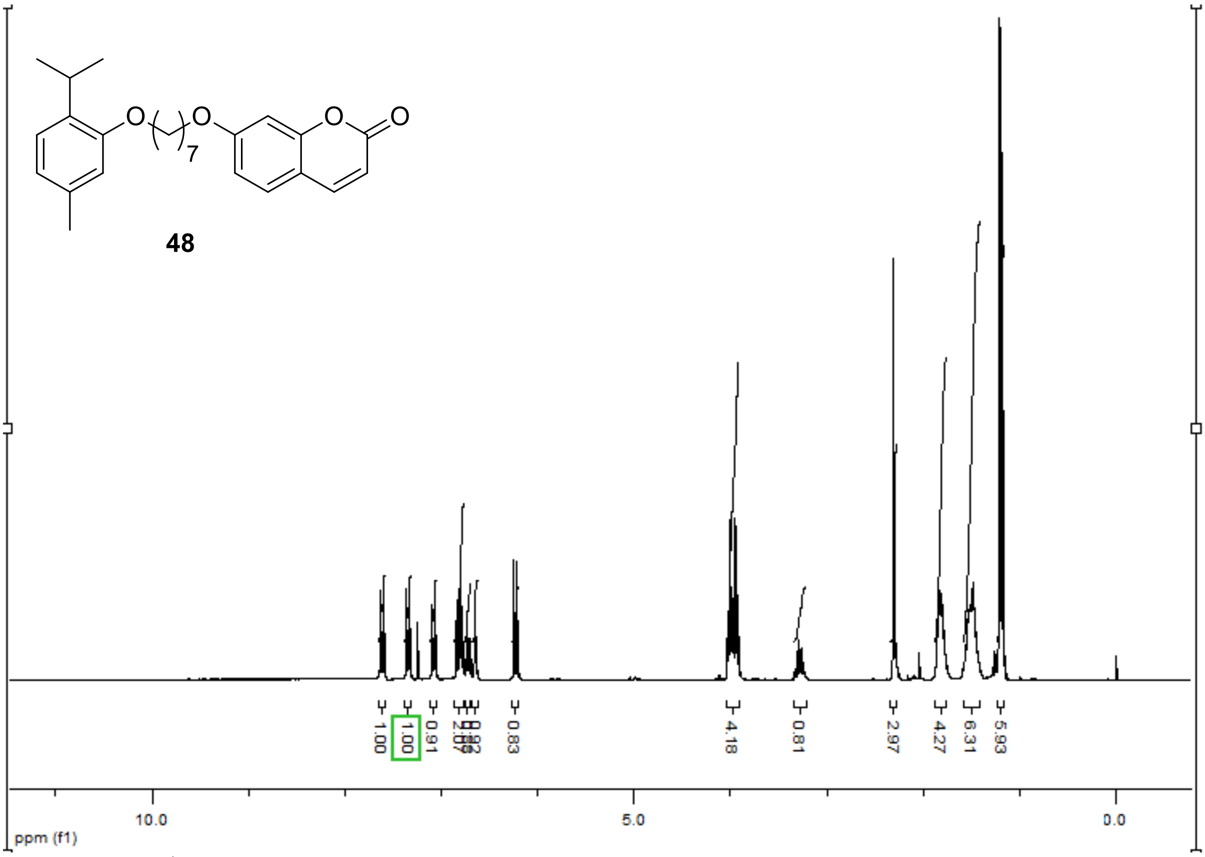

Figure S55. ^{1}H NMR (300 MHz, $CDCl_3$) spectrum of **48**

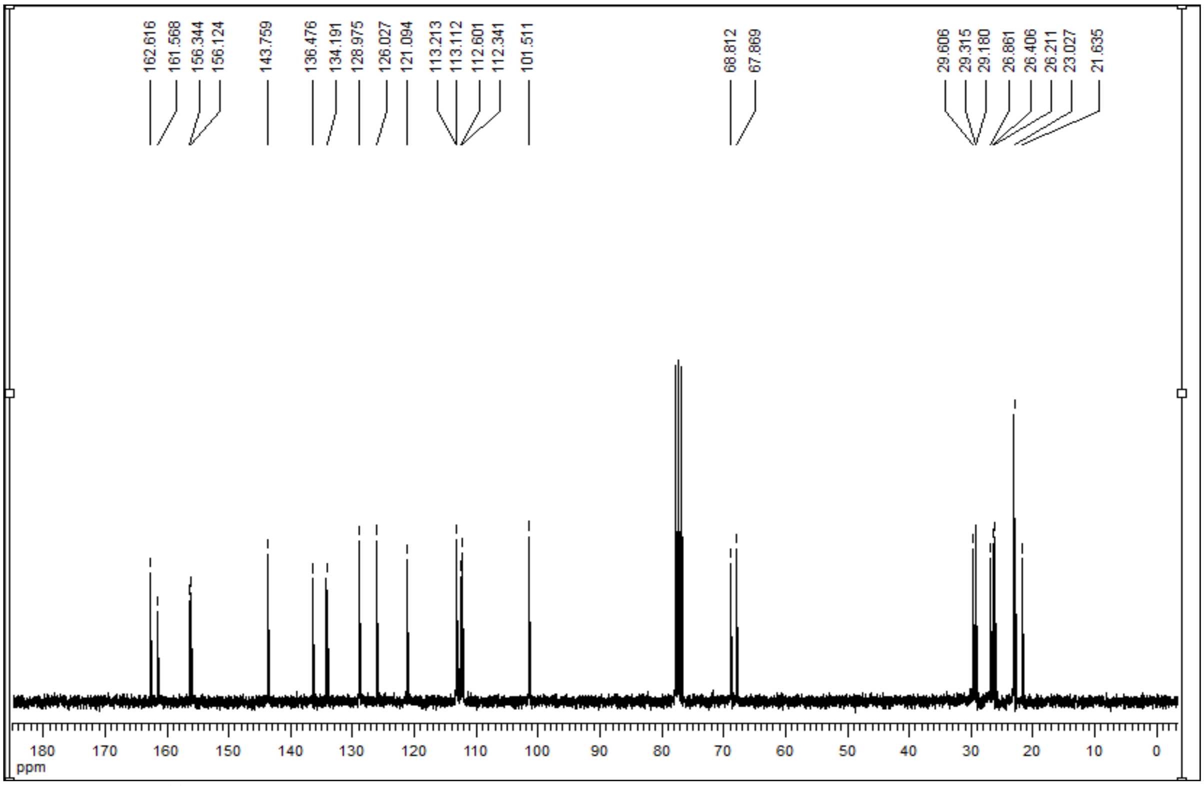

Figure S56. ^{13}C NMR (75 MHz, $CDCl_3$) spectrum of **48**

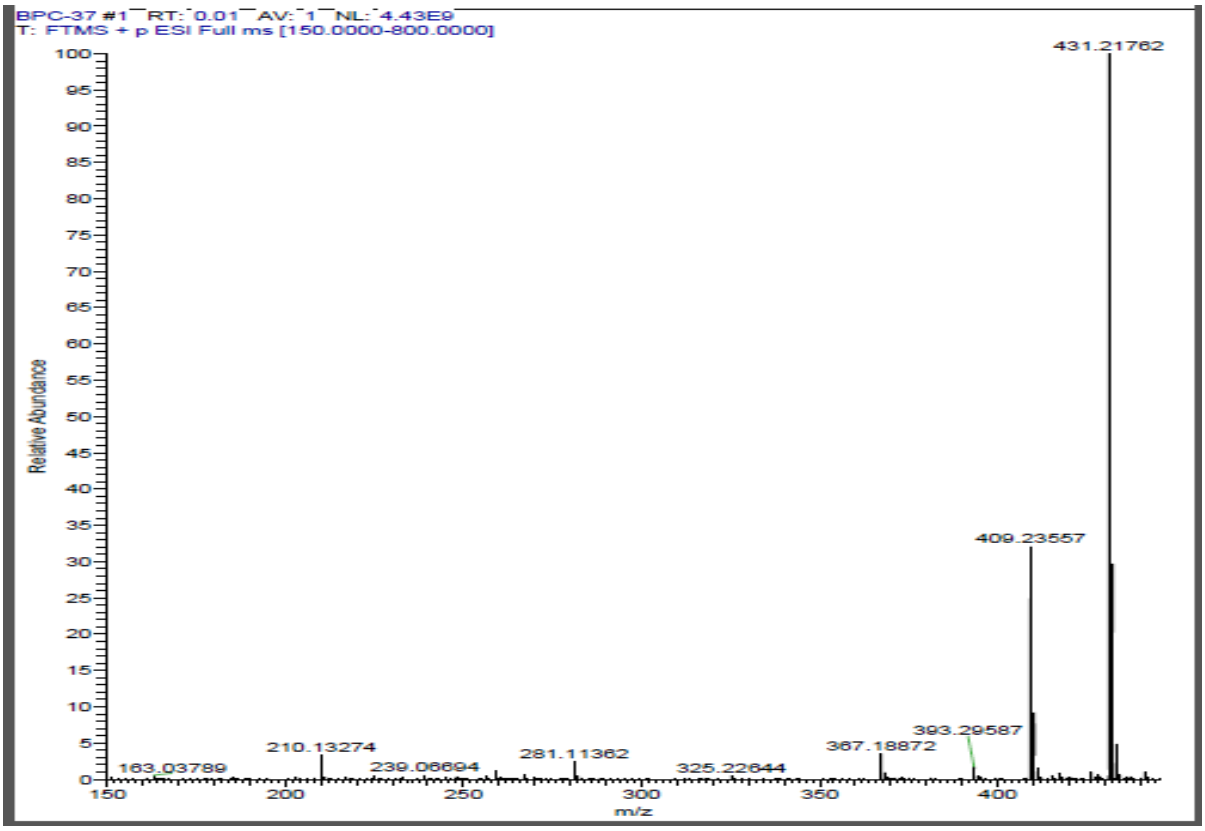

Figure S57. Mass spectrum of **48**

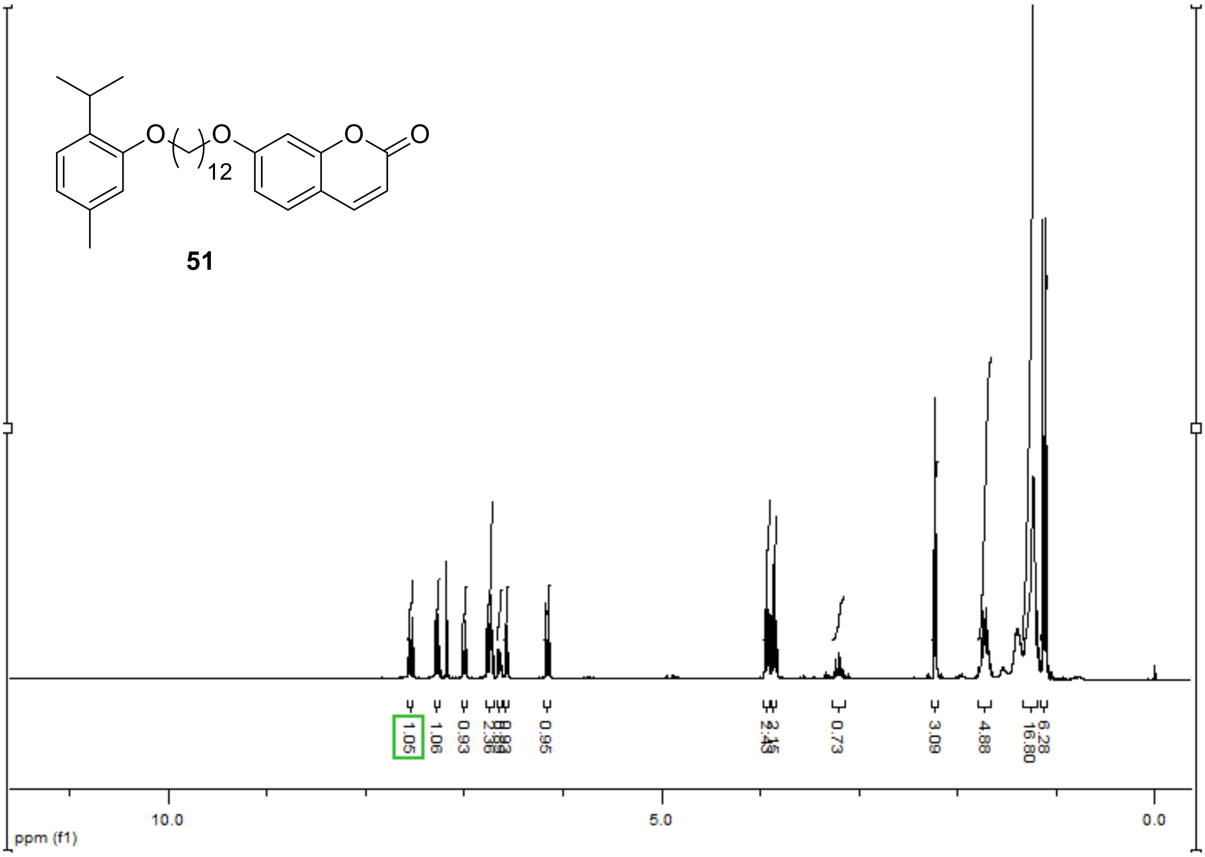

Figure S58. ^{1}H NMR (300 MHz, $CDCl_3$) spectrum of **51**

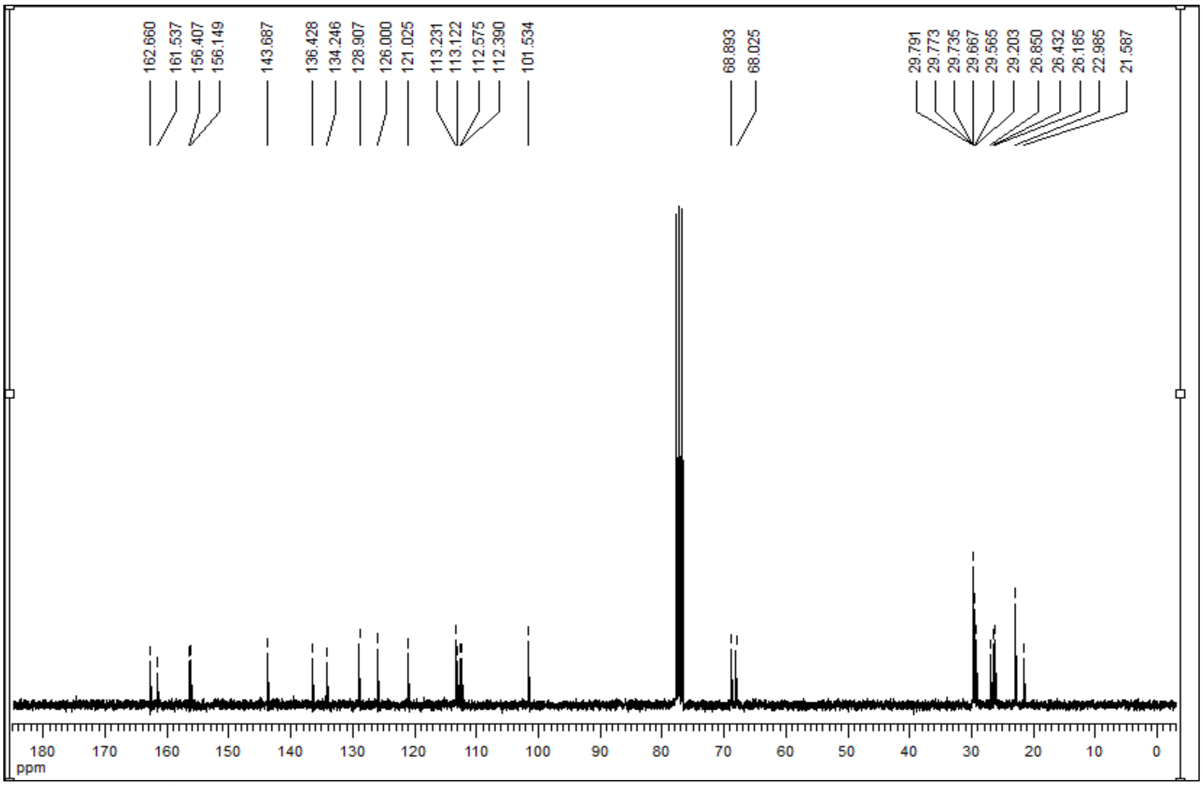

Figure S59. ^{13}C NMR (75 MHz, $CDCl_3$) spectrum of **51**

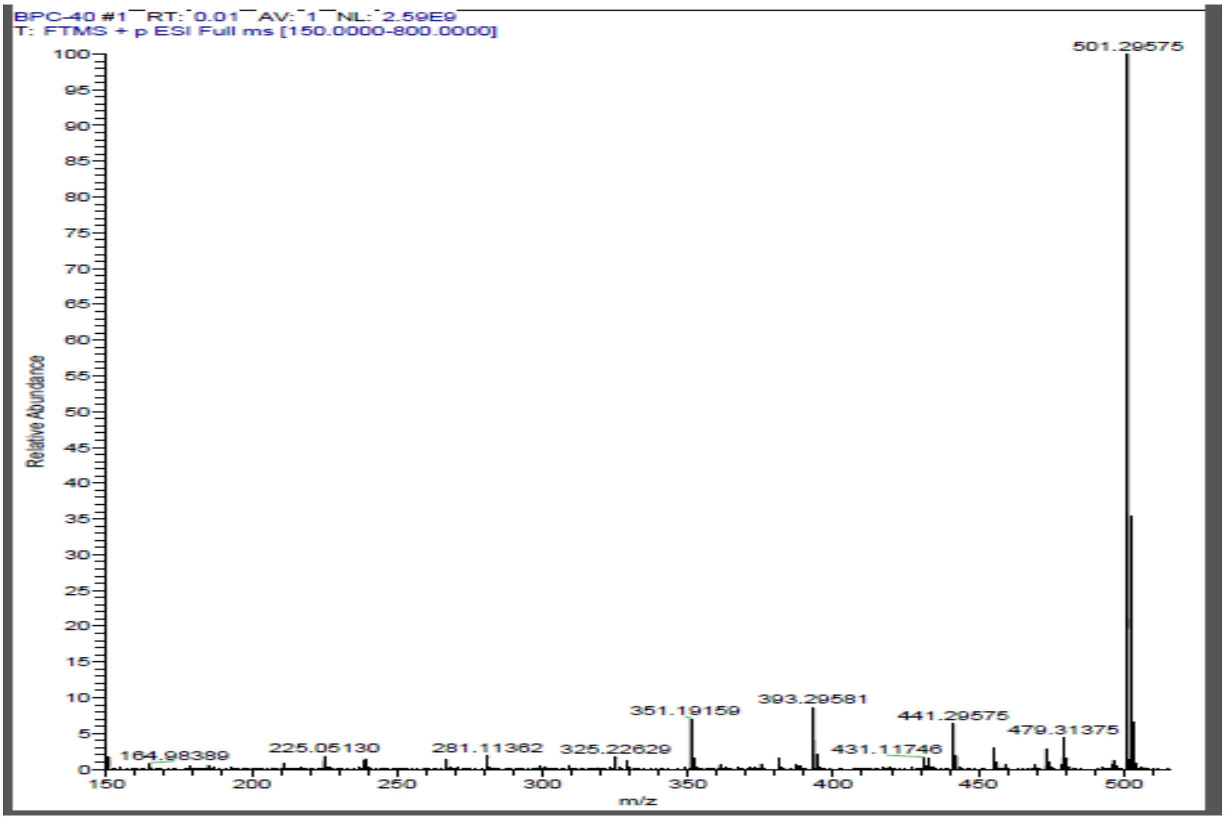

Figure S60. Mass spectrum of **51**

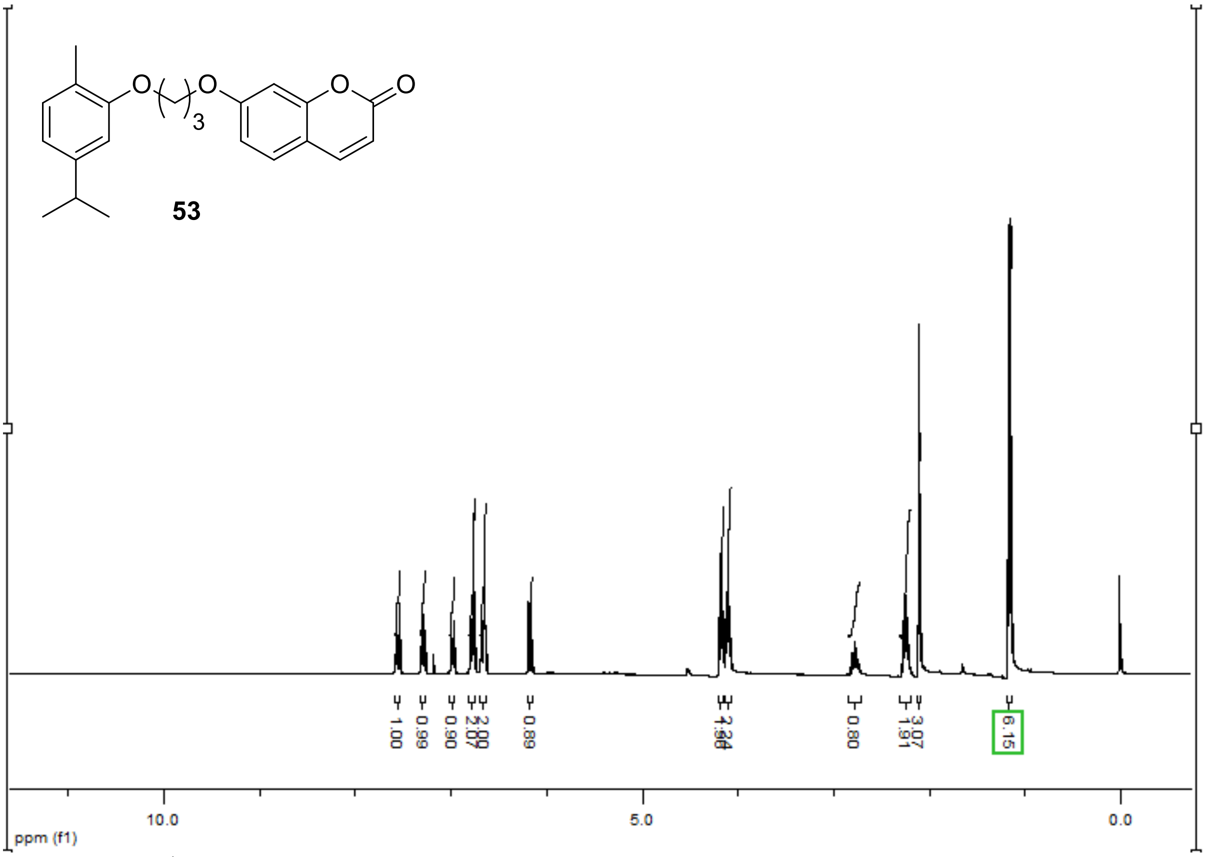

Figure S61. ^{1}H NMR (300 MHz, $CDCl_3$) spectrum of **53**

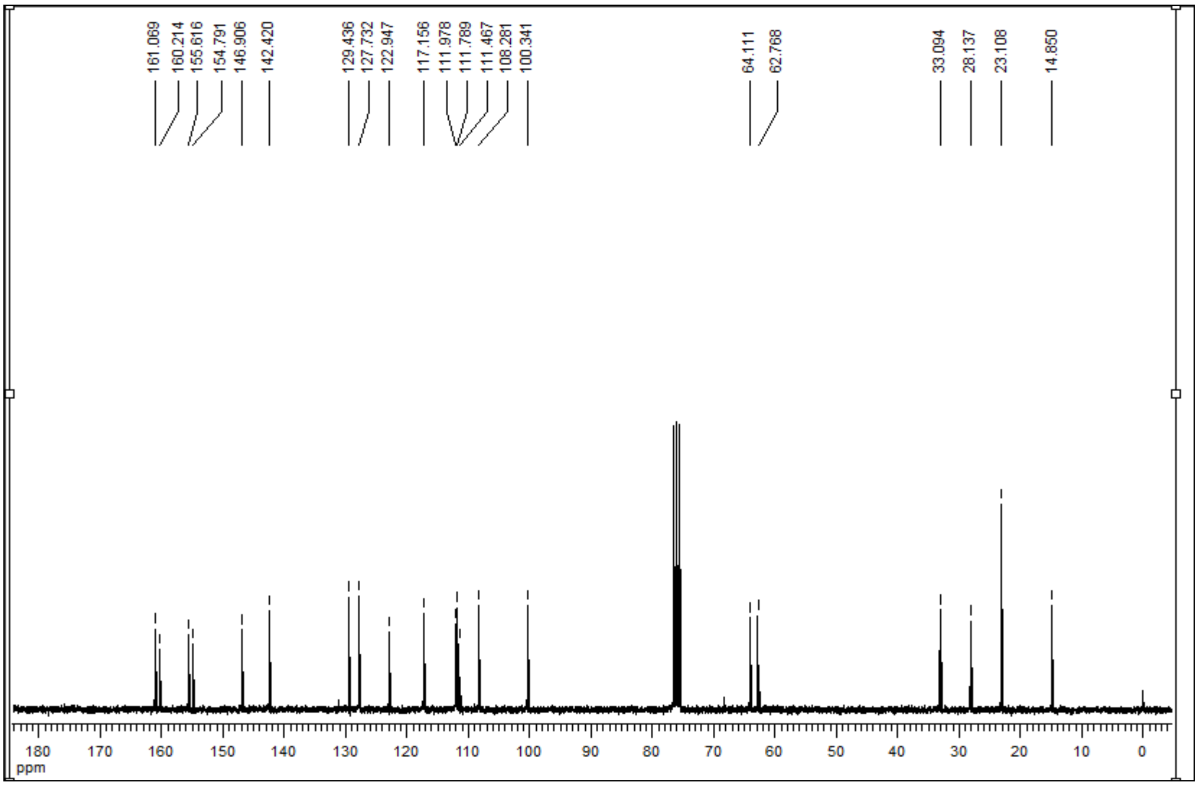

Figure S62. ^{13}C NMR (75 MHz, $CDCl_3$) spectrum of **53**

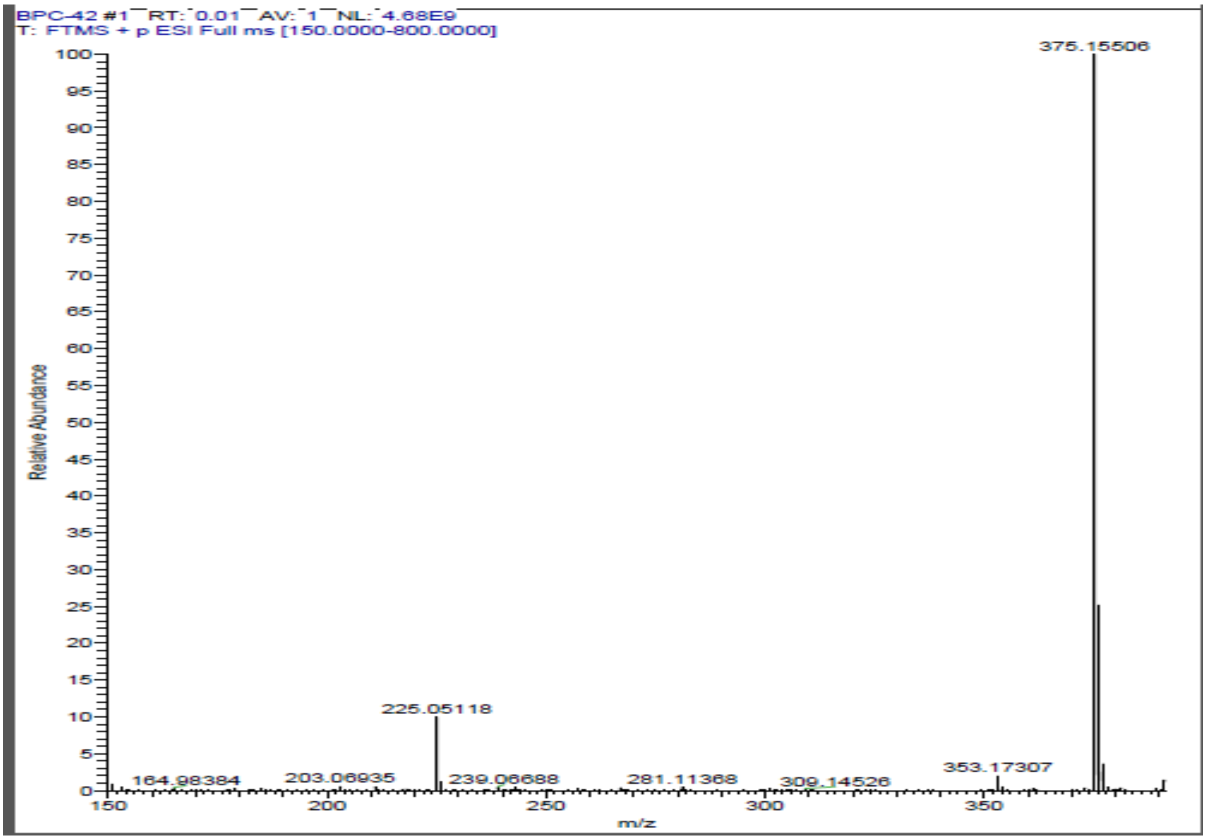

Figure S63. Mass spectrum of **53**

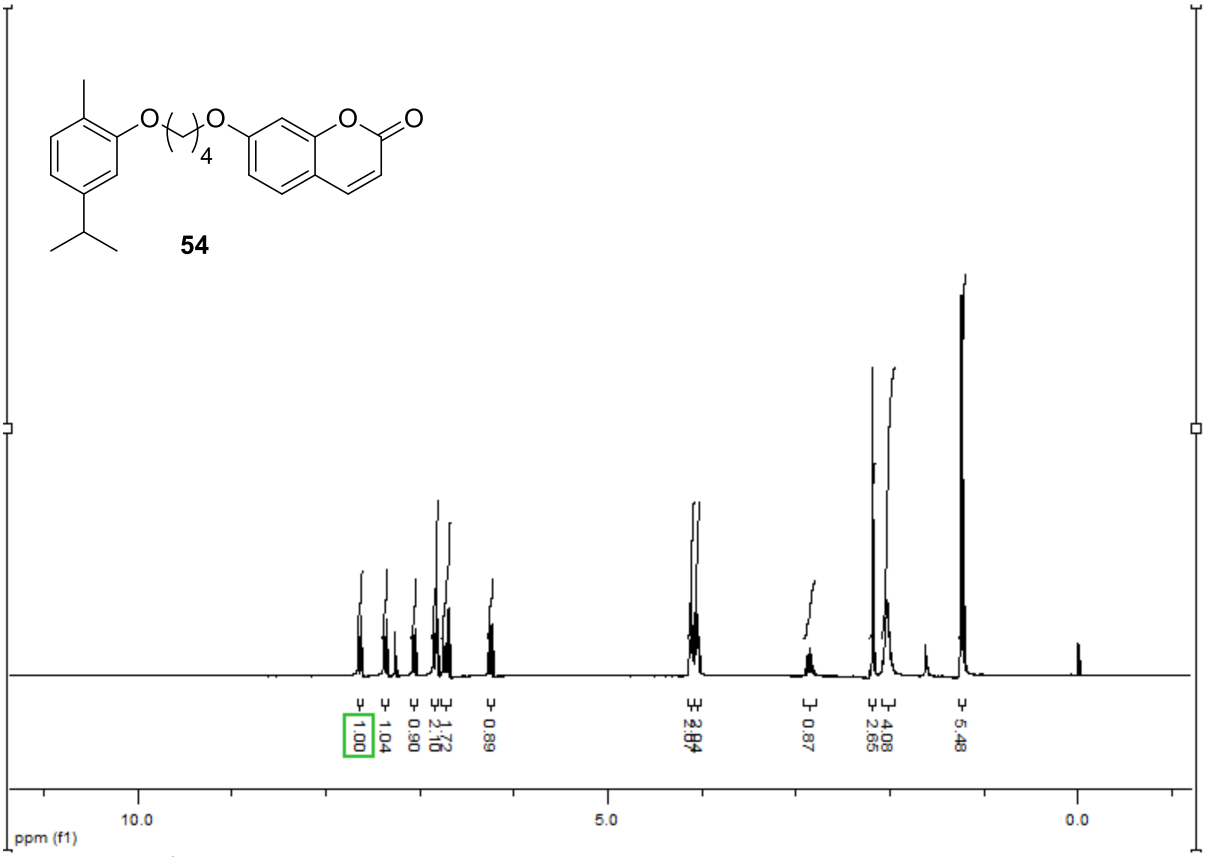

Figure S64. ^{1}H NMR (300 MHz, $CDCl_3$) spectrum of **54**

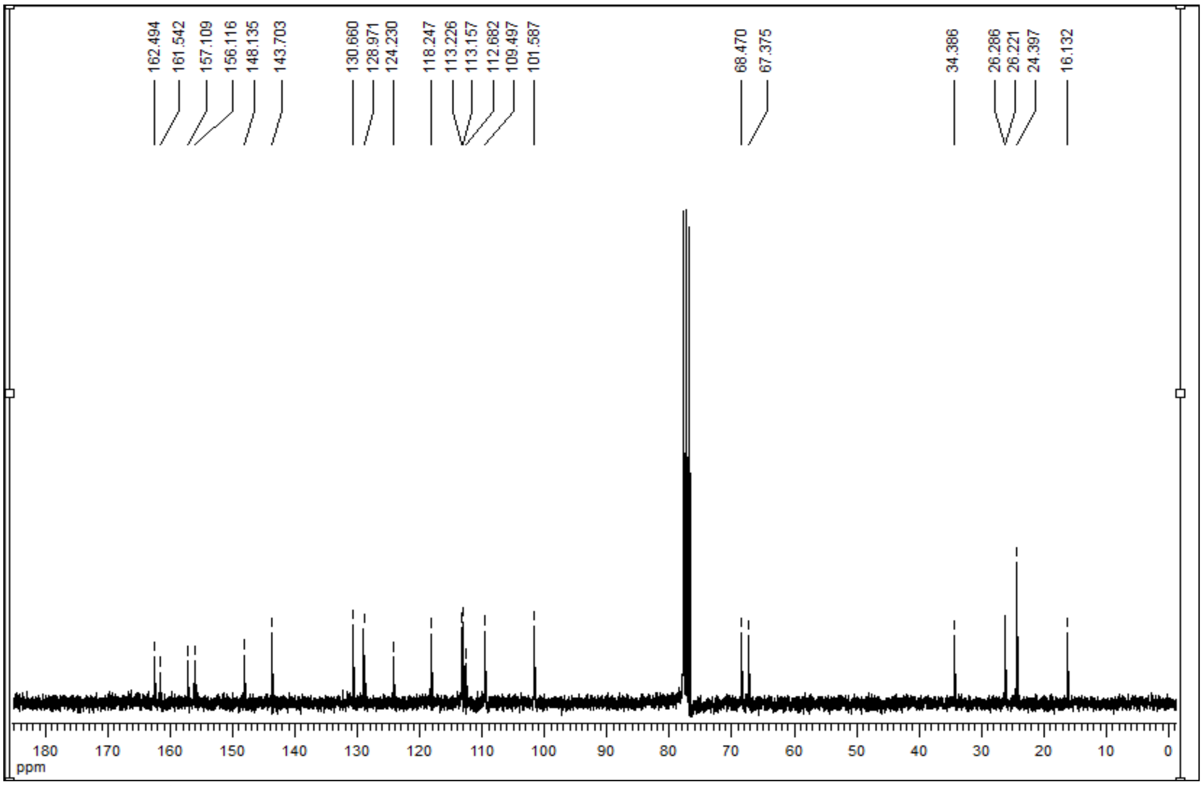

Figure S65. ^{13}C NMR (75 MHz, $CDCl_3$) spectrum of **54**

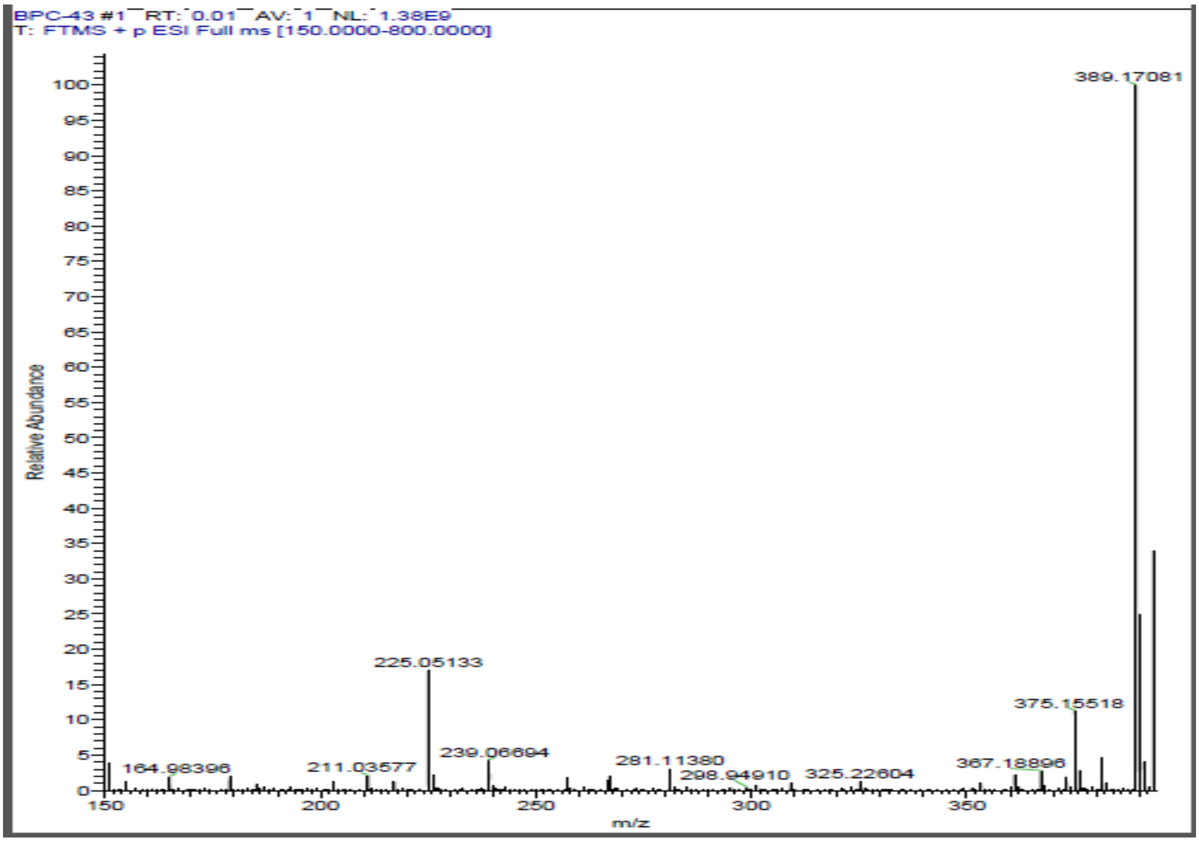

Figure S66. Mass spectrum of **54**

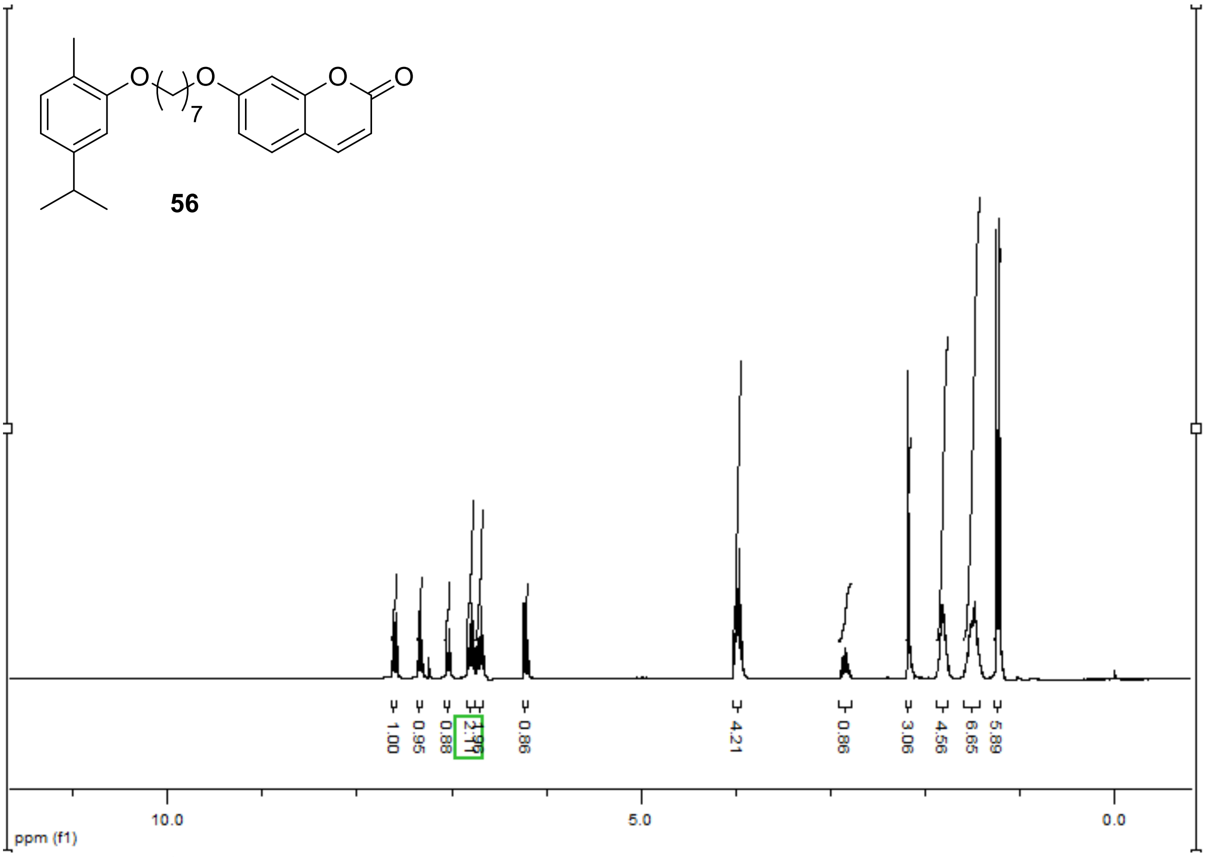

Figure S67. ^{1}H NMR (300 MHz, $CDCl_3$) spectrum of **56**

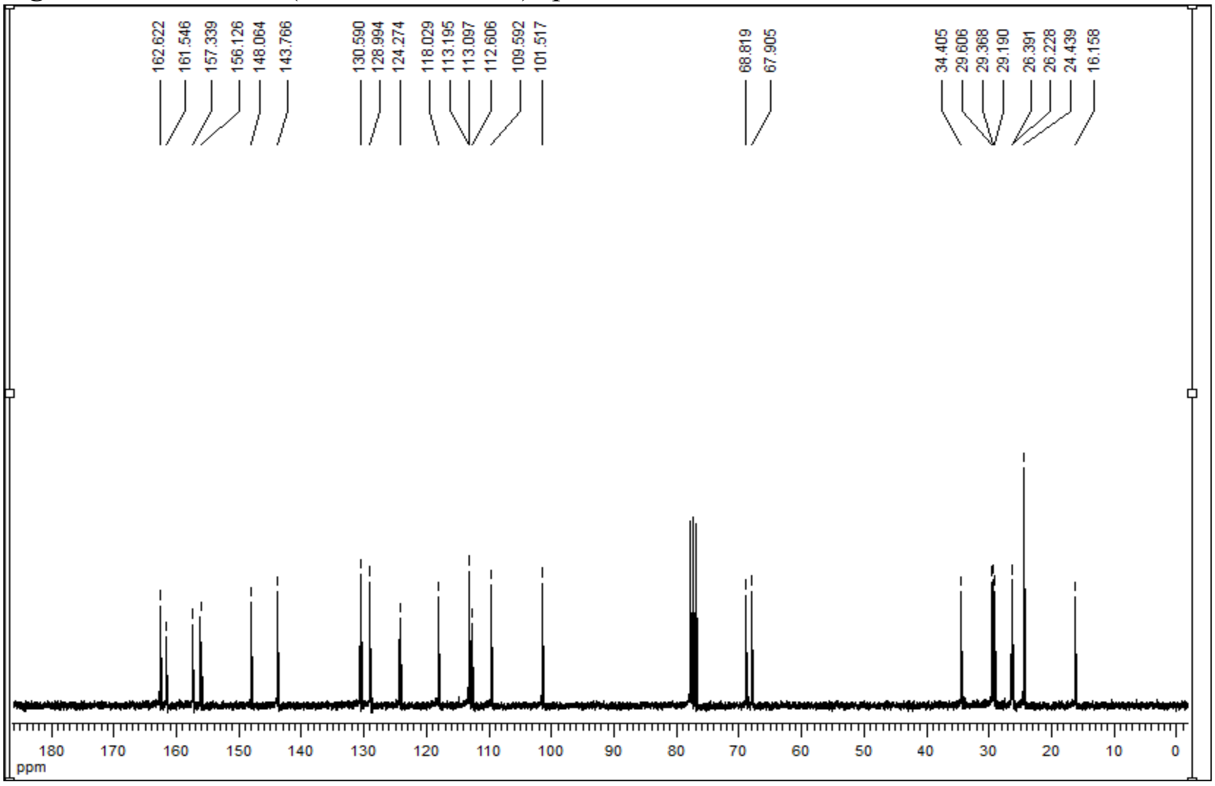

Figure S68. ^{13}C NMR (75 MHz, $CDCl_3$) spectrum of **56**

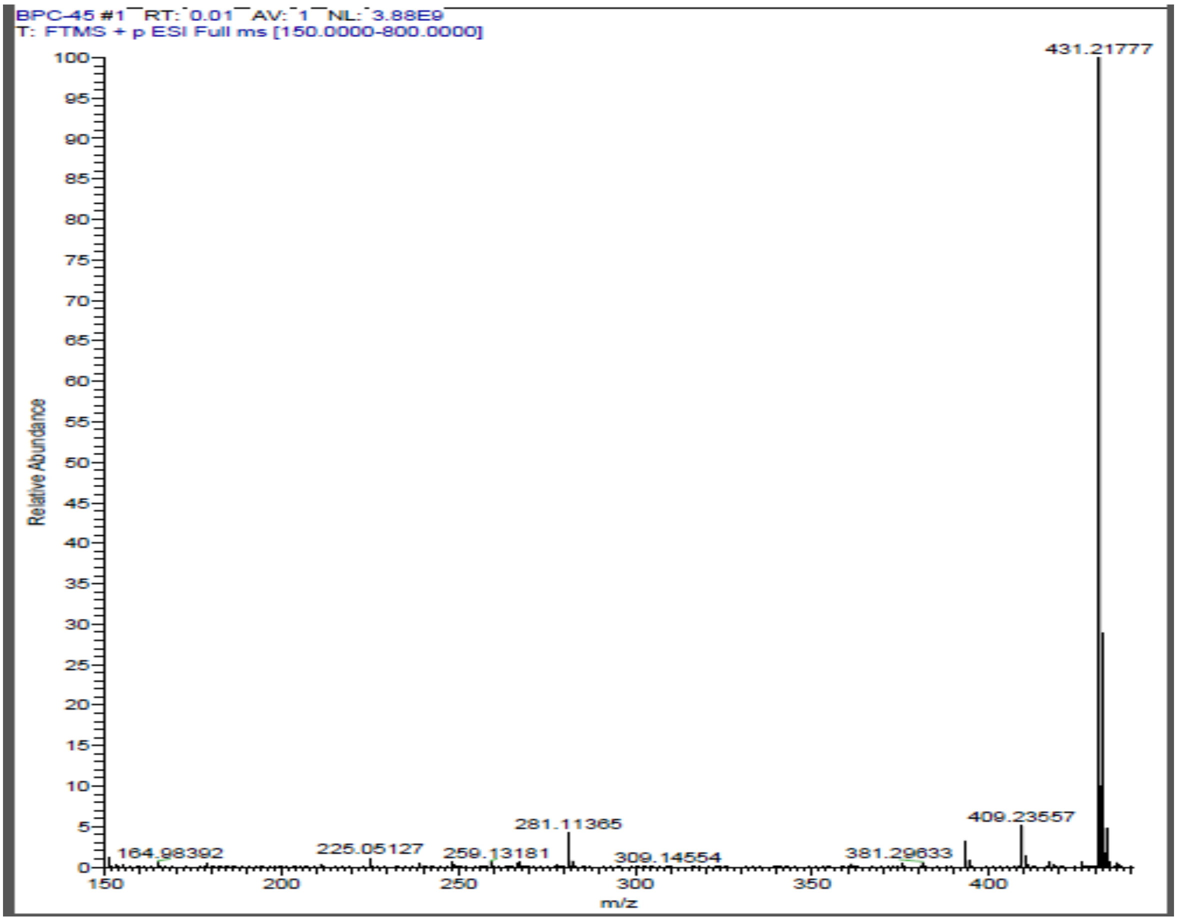

Figure S69. Mass spectrum of **56**

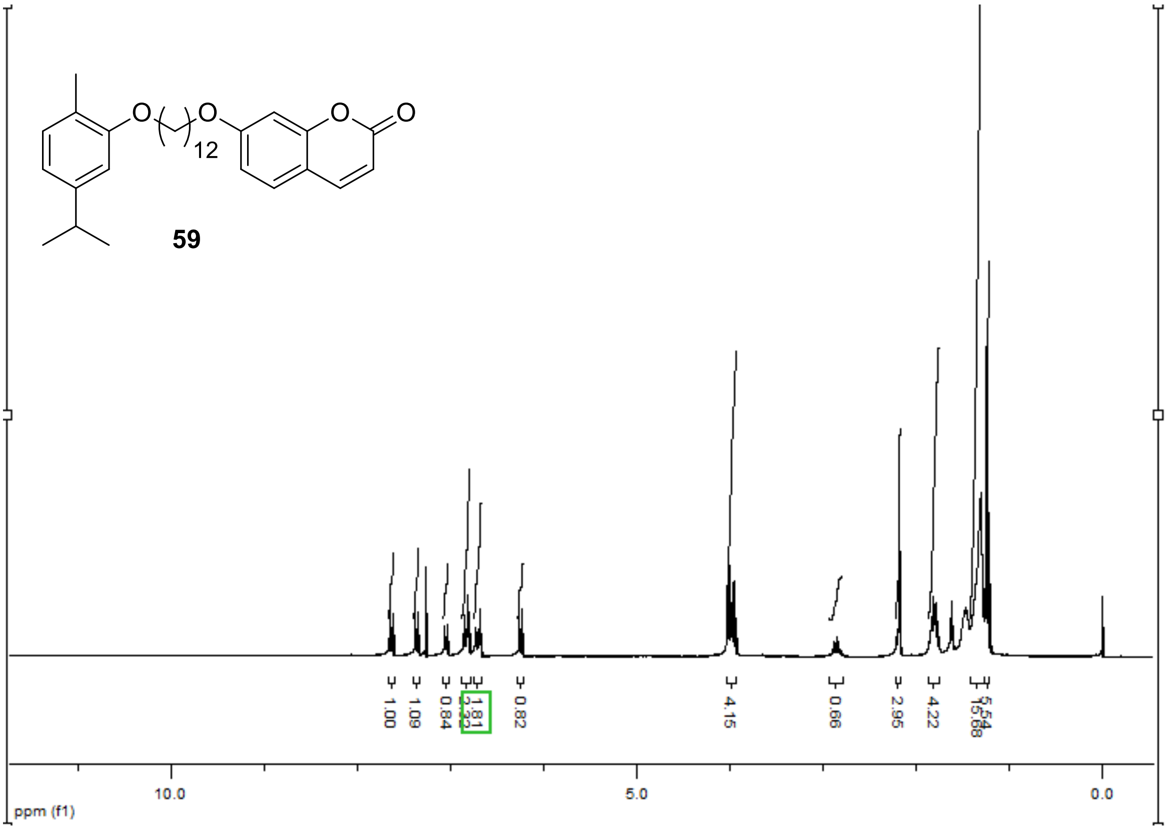

Figure S70. ^{1}H NMR (300 MHz, $CDCl_3$) spectrum of **59**

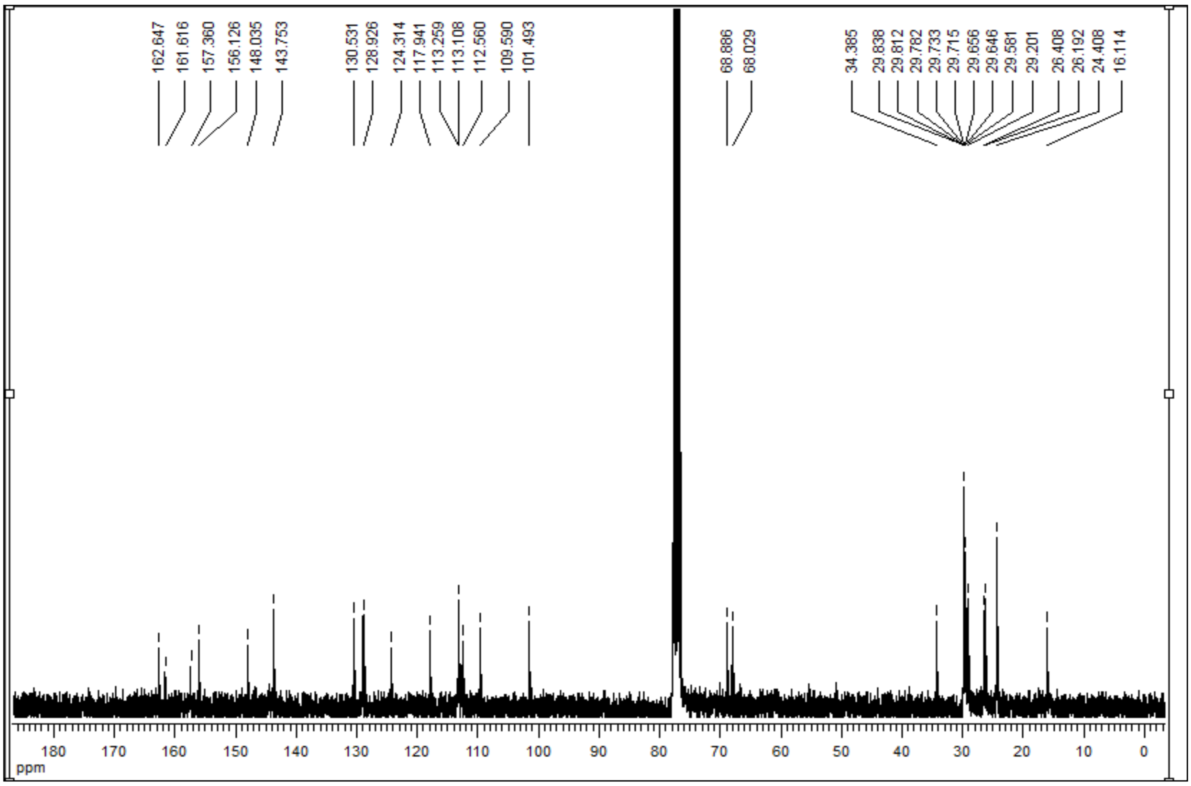

Figure S71. ^{13}C NMR (75 MHz, $CDCl_3$) spectrum of **59**

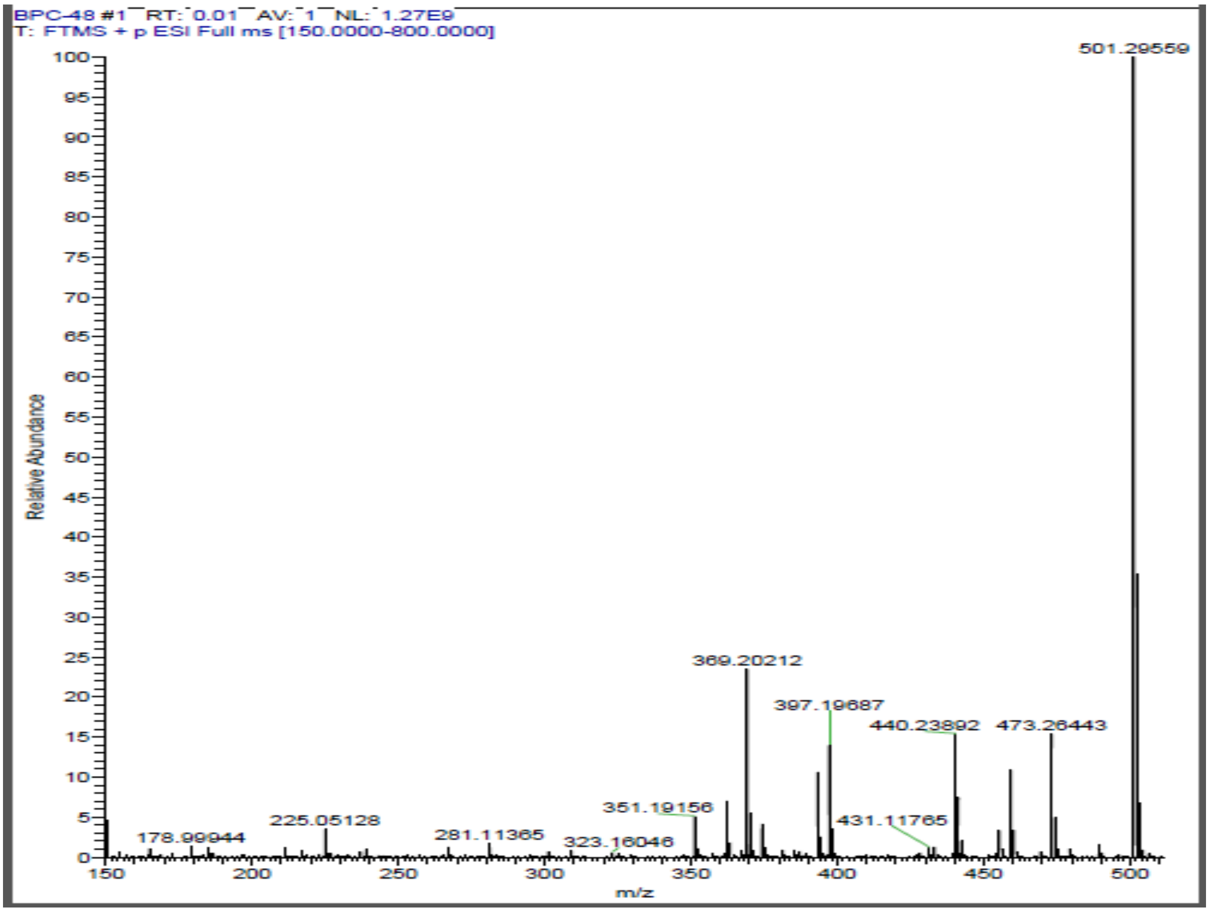

Figure S72. Mass spectrum of **59**

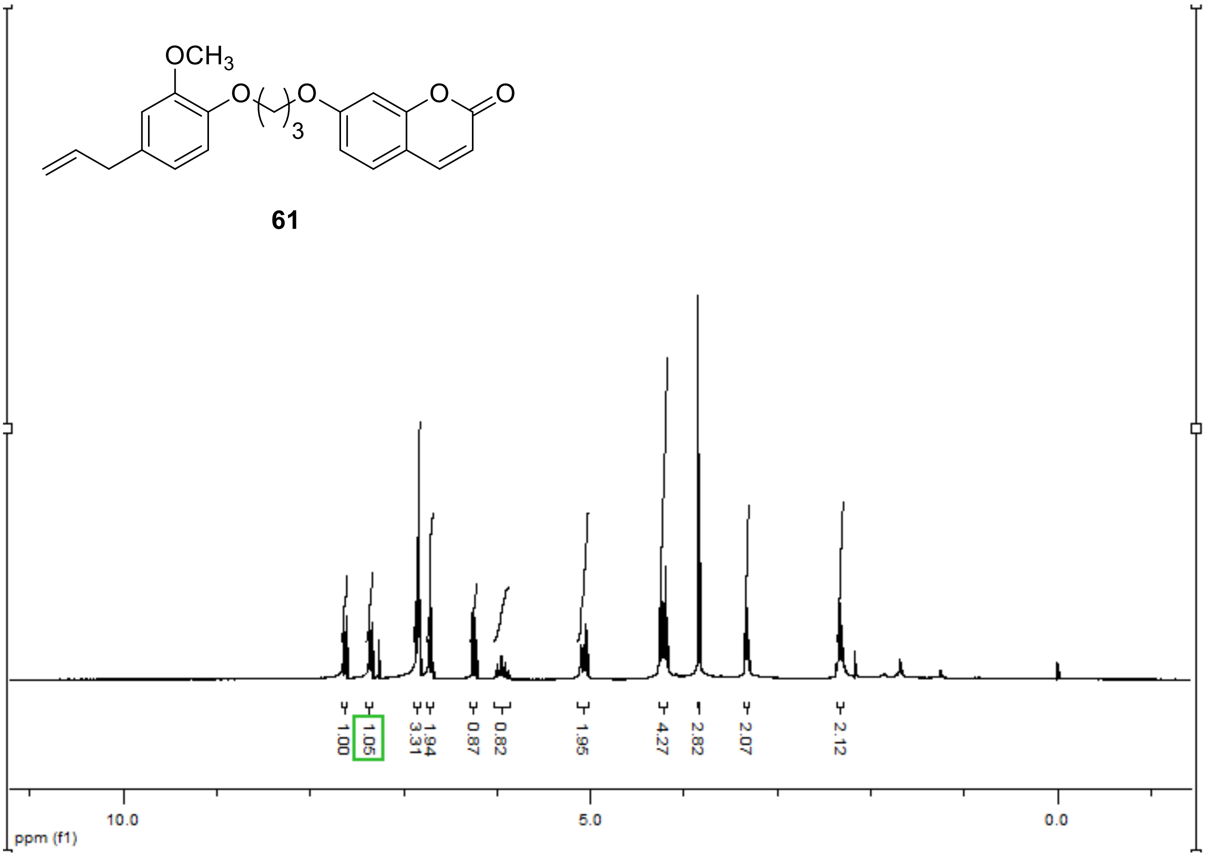

Figure S73. ^{1}H NMR (300 MHz, $CDCl_3$) spectrum of **61**

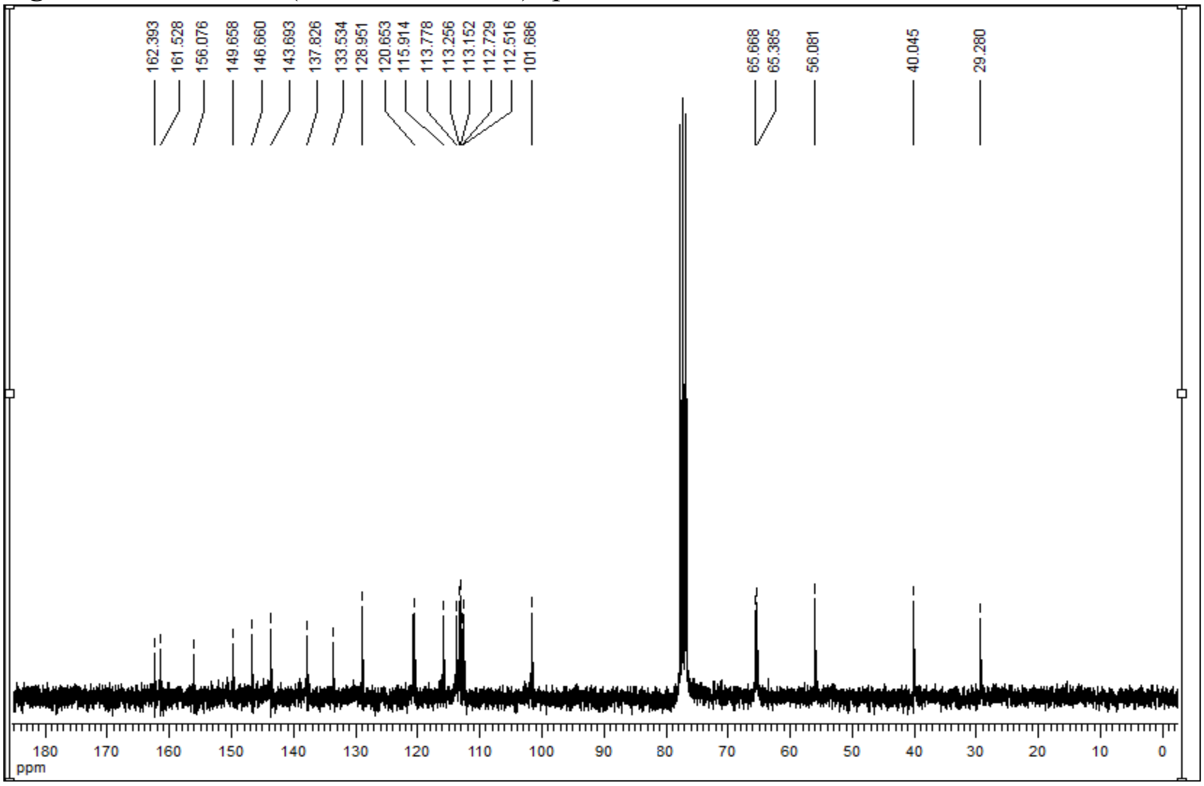

Figure S74. ^{13}C NMR (75 MHz, $CDCl_3$) spectrum of **61**

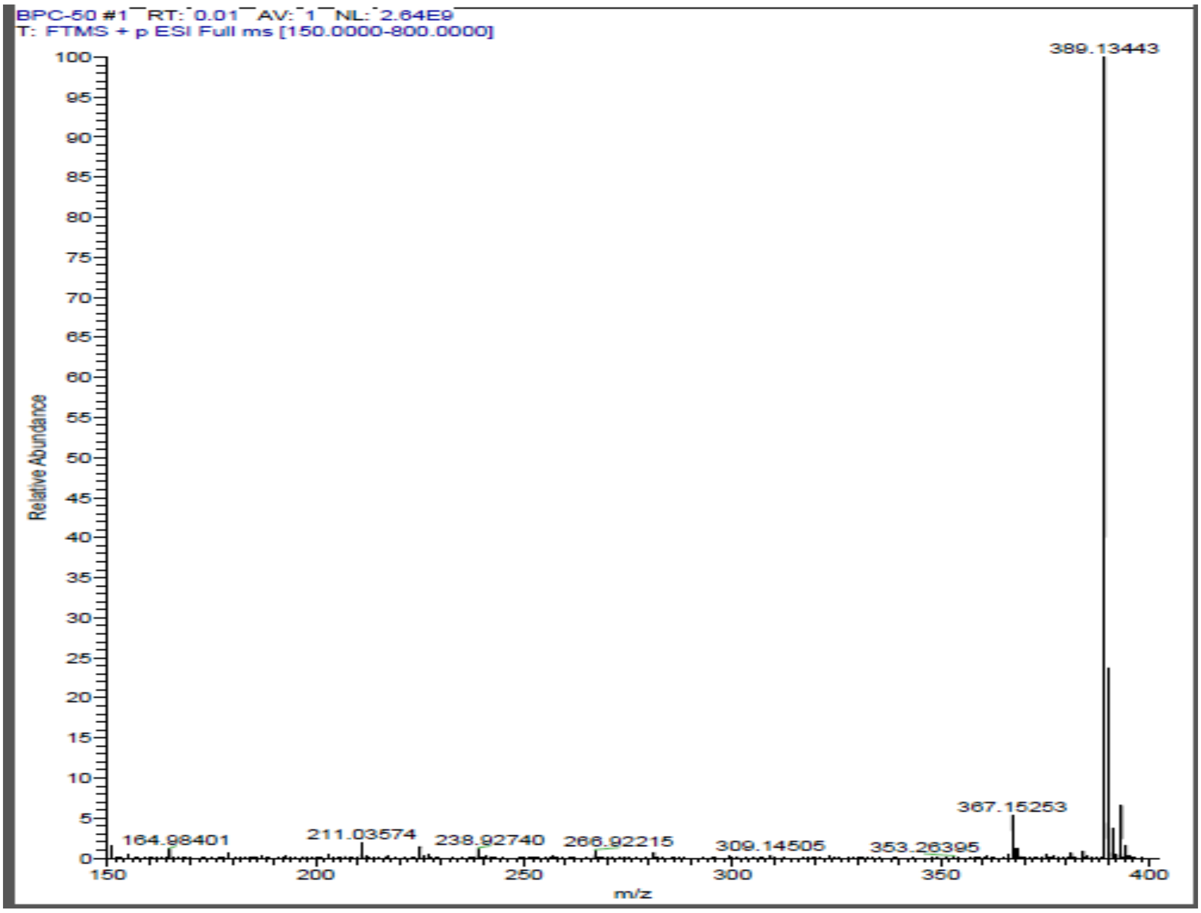

Figure S75. Mass spectrum of **61**

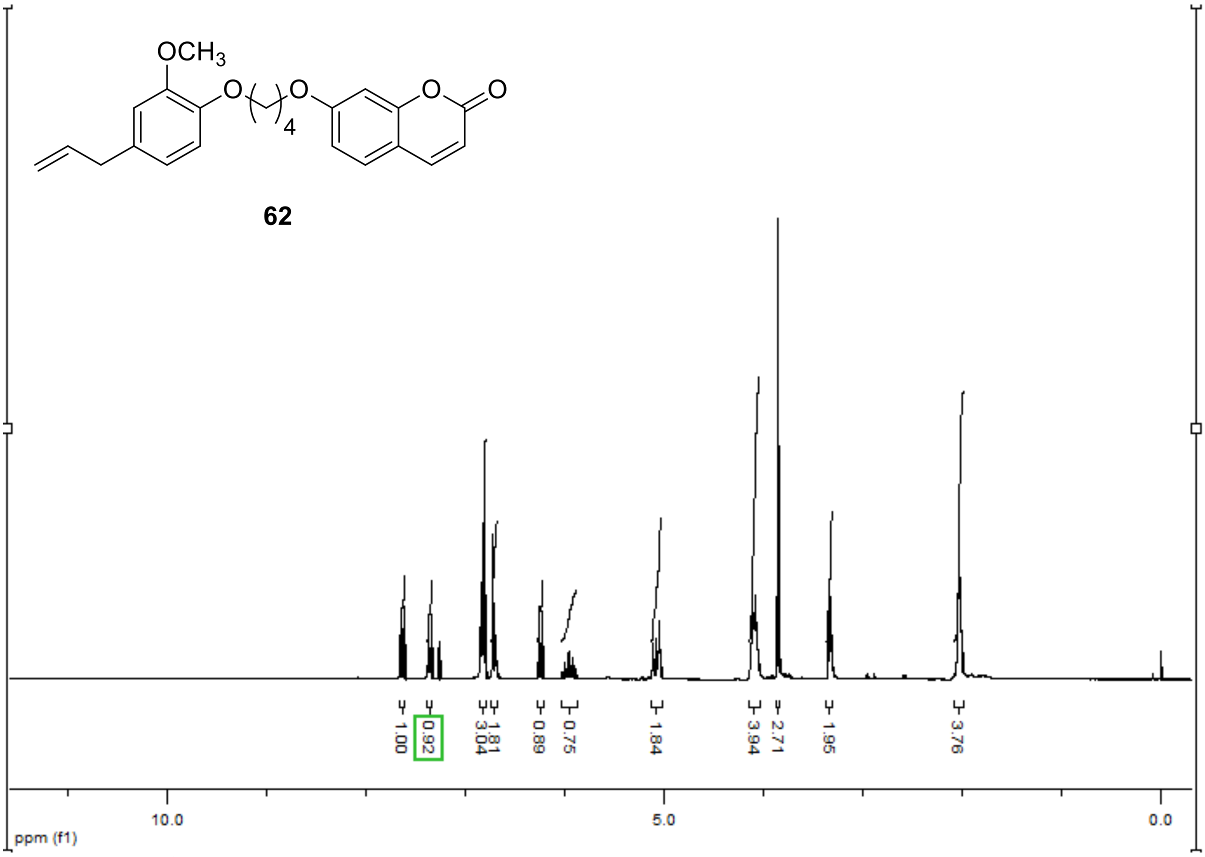

Figure S76. ^{1}H NMR (300 MHz, $CDCl_3$) spectrum of **62**

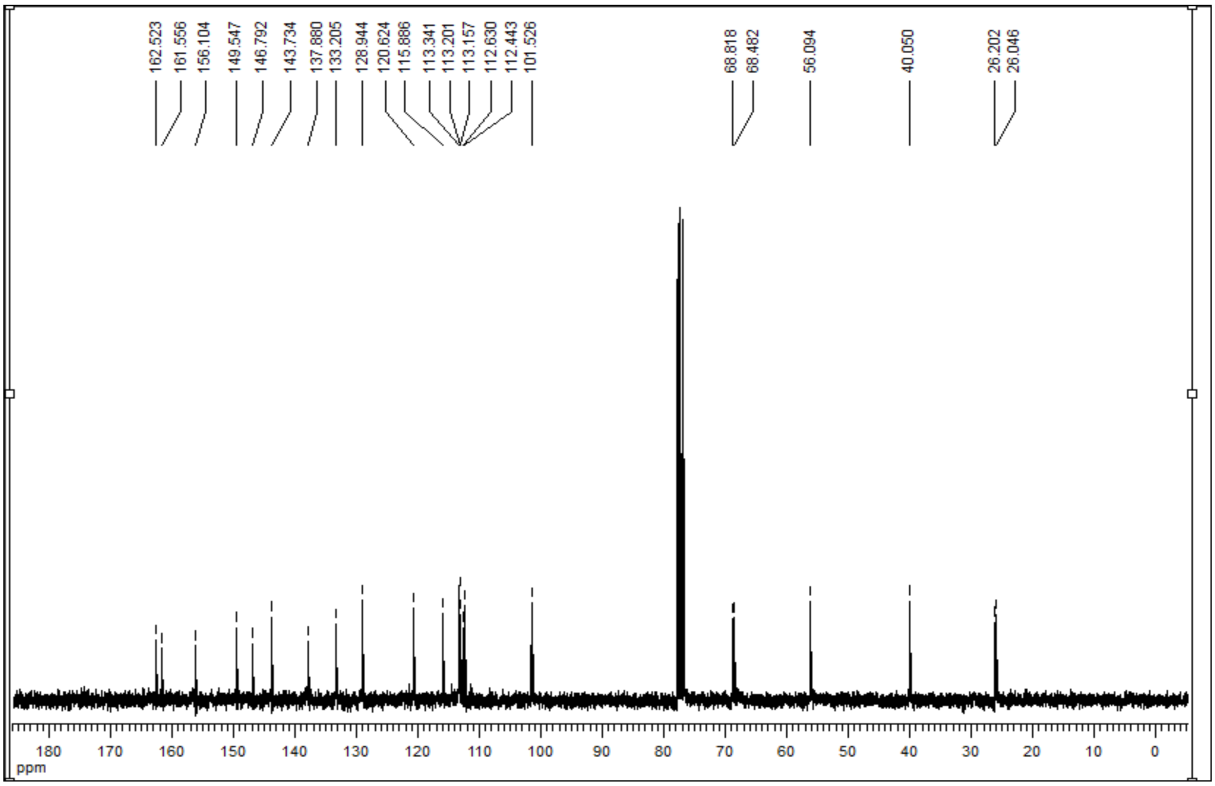

Figure S77. ^{13}C NMR (75 MHz, $CDCl_3$) spectrum of **62**

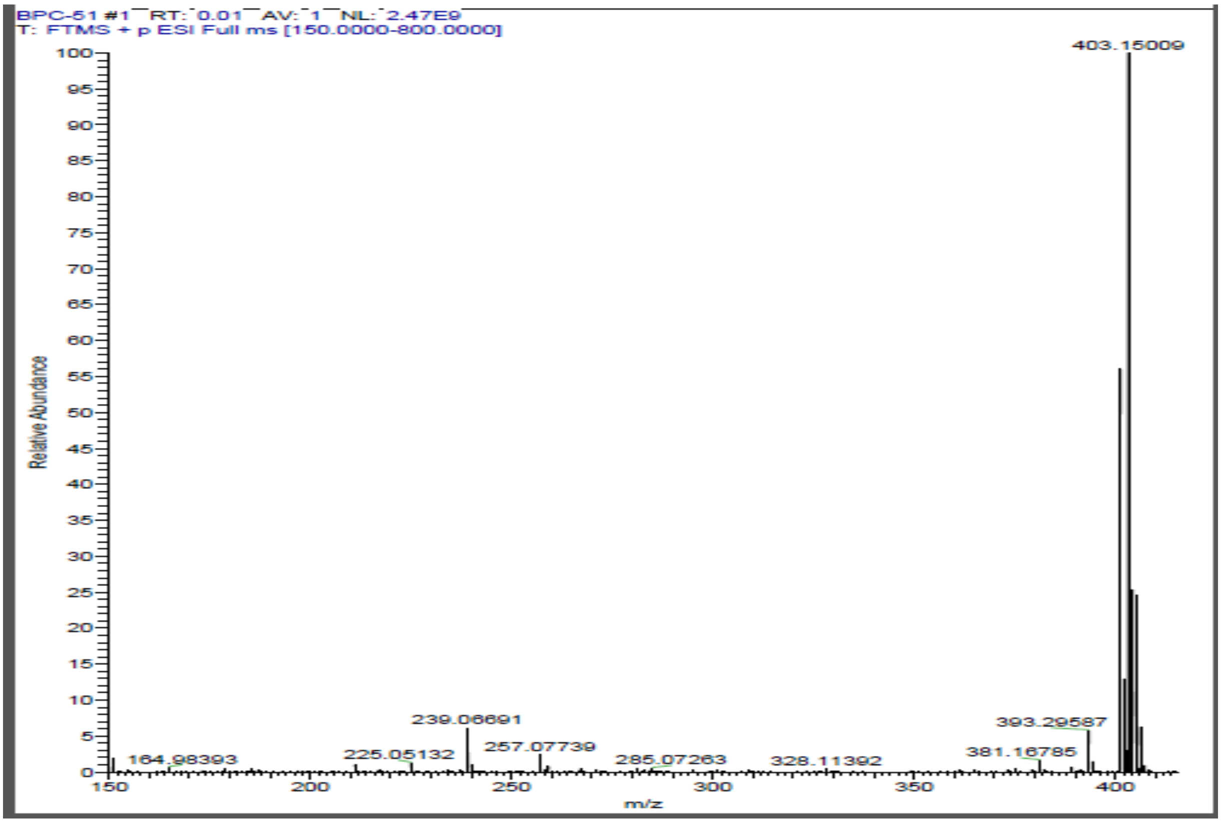

Figure S78. Mass spectrum of **62**

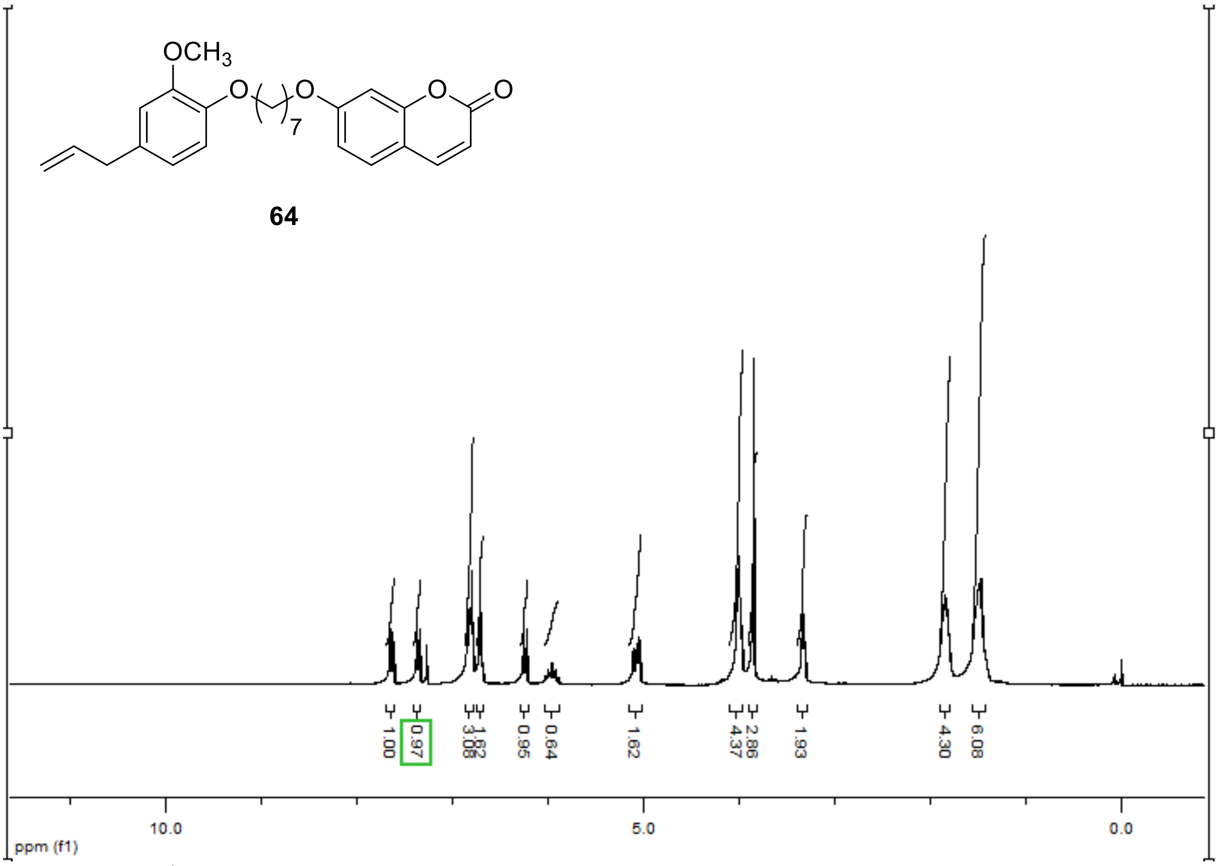

Figure S79. ^{1}H NMR (300 MHz, $CDCl_3$) spectrum of **64**

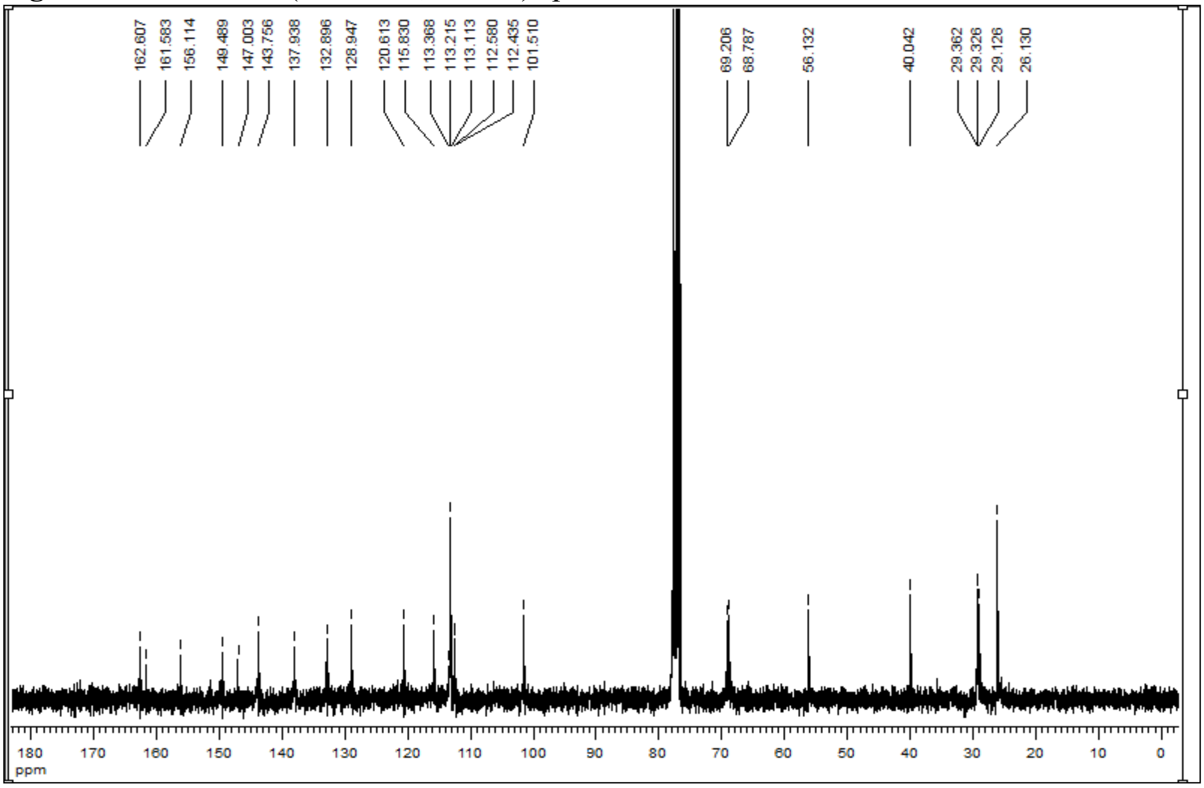

Figure S80. ^{13}C NMR (75 MHz, $CDCl_3$) spectrum of **64**

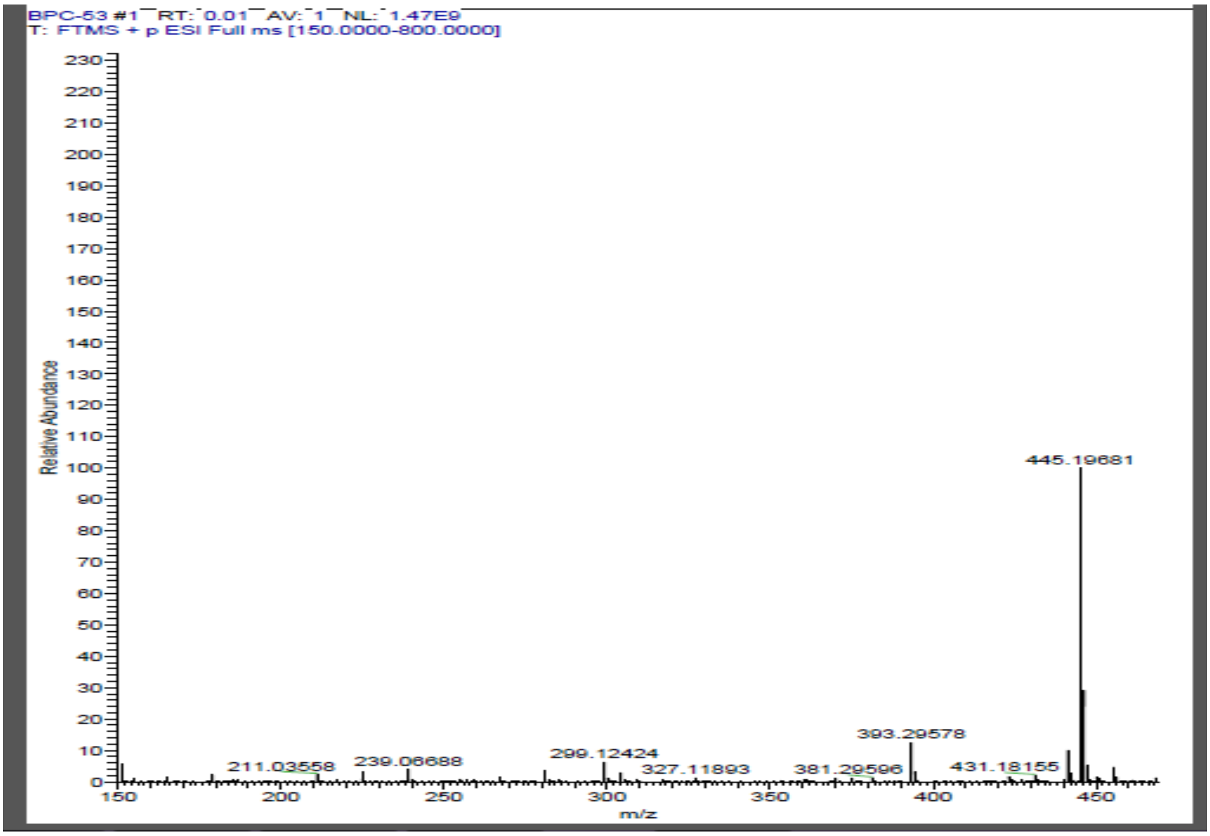

Figure S81. Mass spectrum of **64**

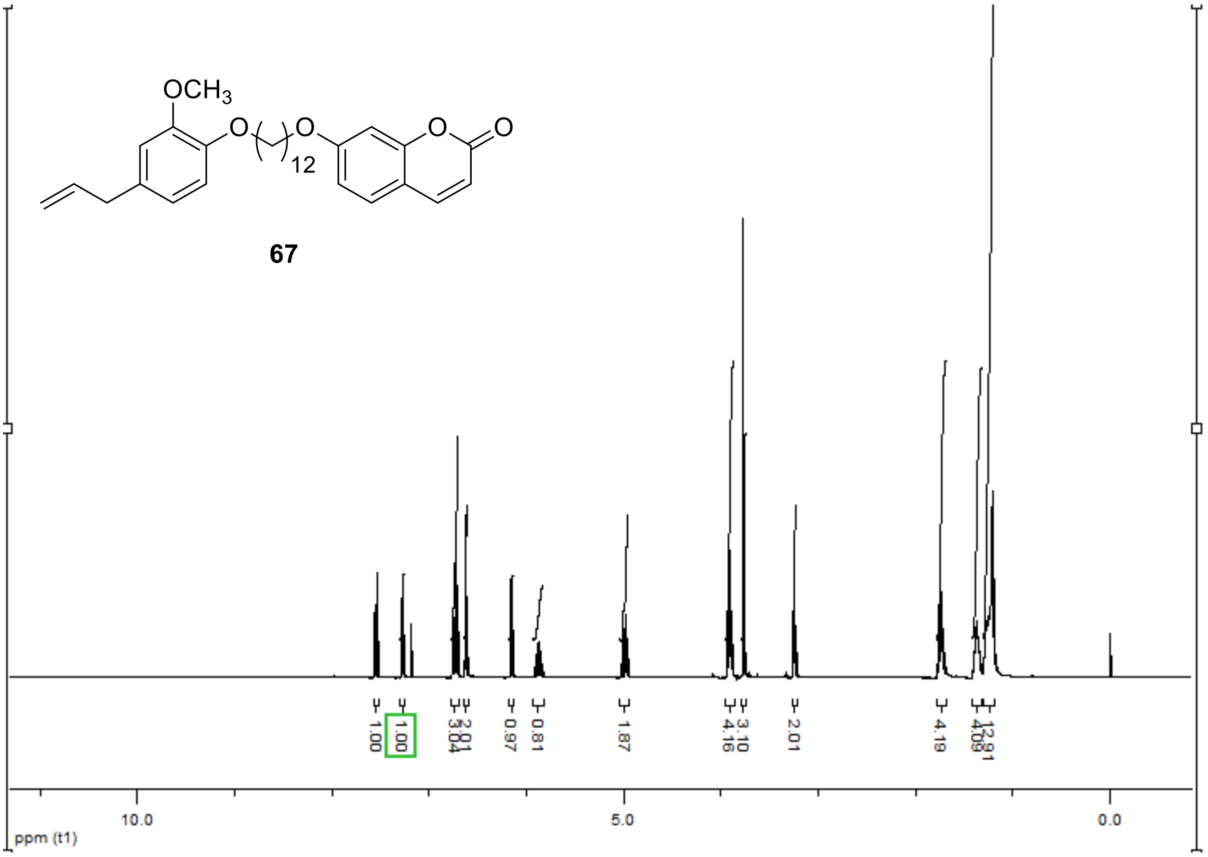

Figure S82. ^{1}H NMR (300 MHz, $CDCl_3$) spectrum of **67**

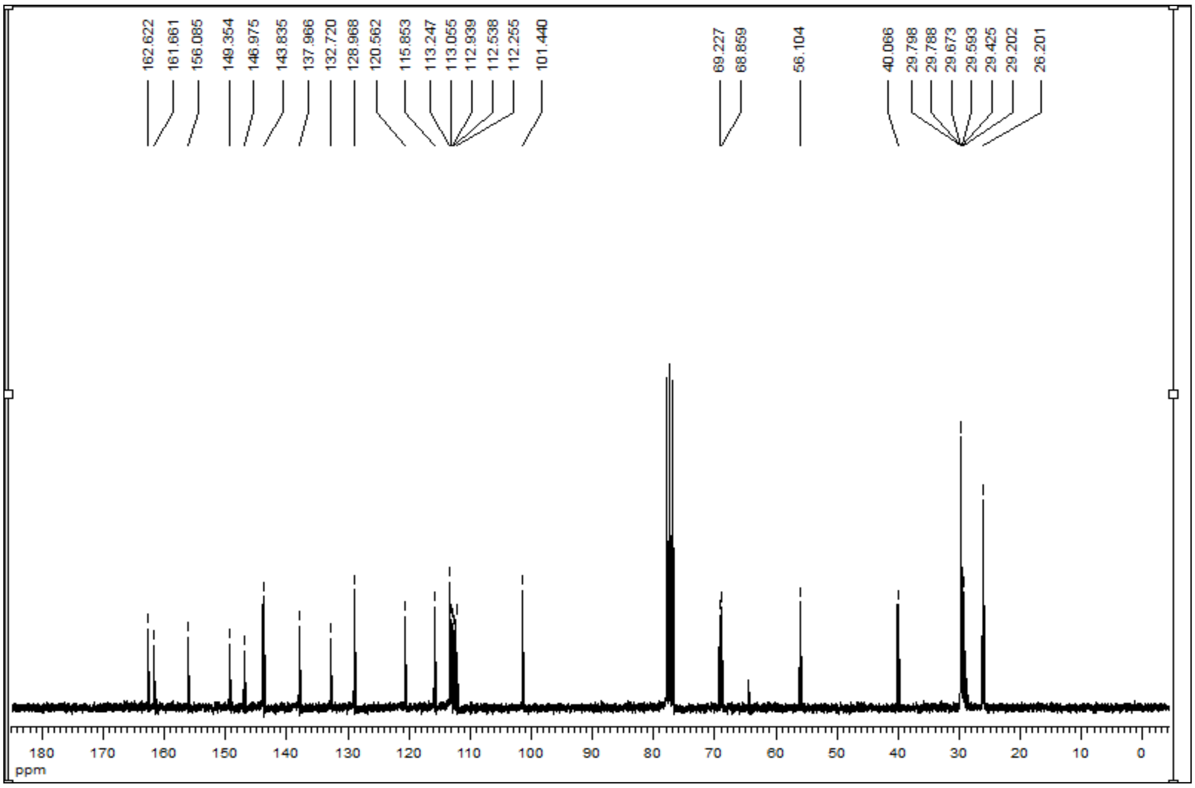

Figure S83. ^{13}C NMR (75 MHz, $CDCl_3$) spectrum of **67**

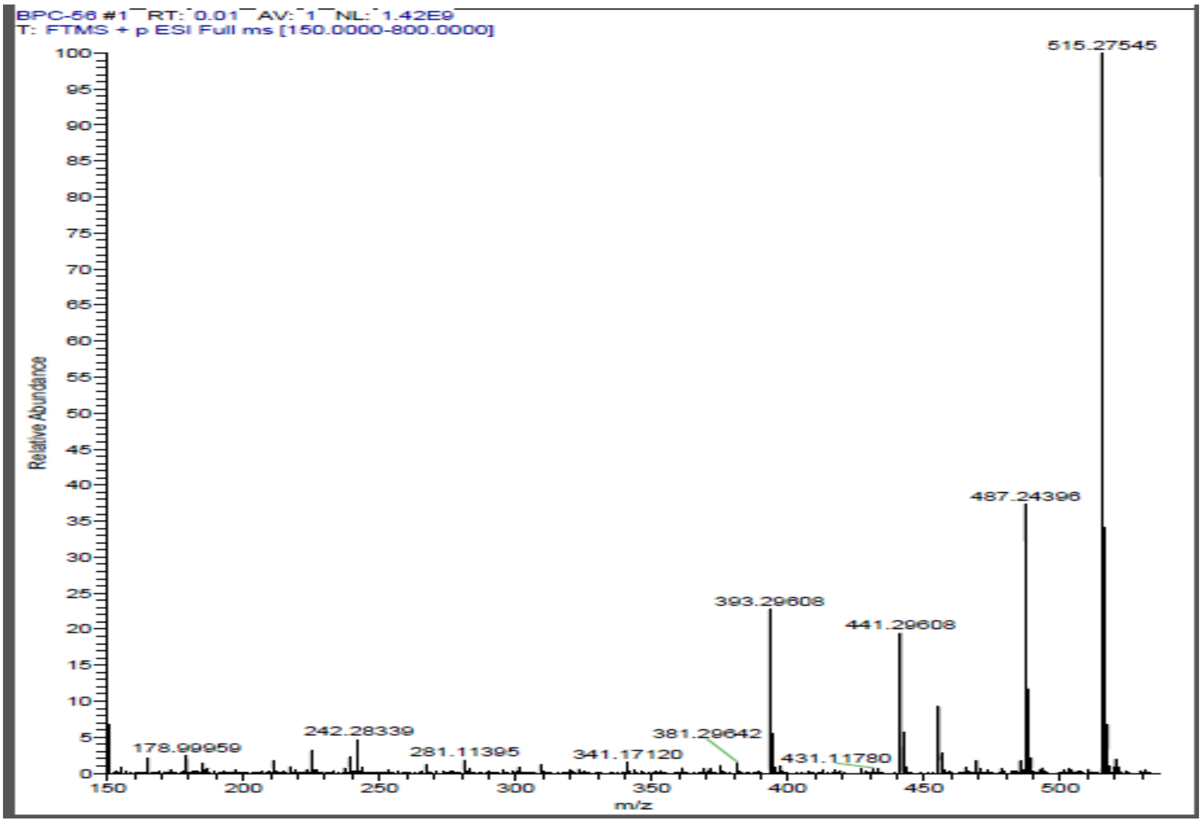

Figure S84. Mass spectrum of **67**

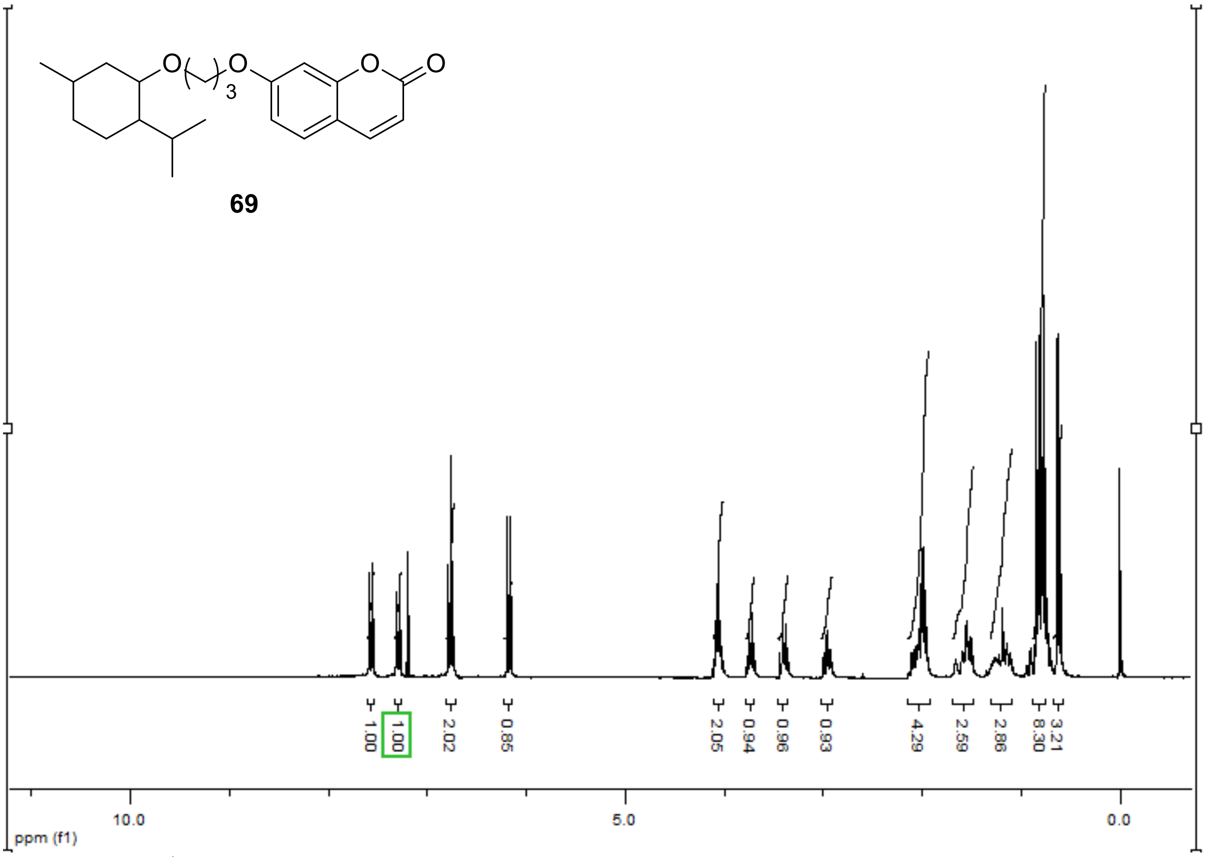

Figure S85. ^{1}H NMR (300 MHz, $CDCl_3$) spectrum of **69**

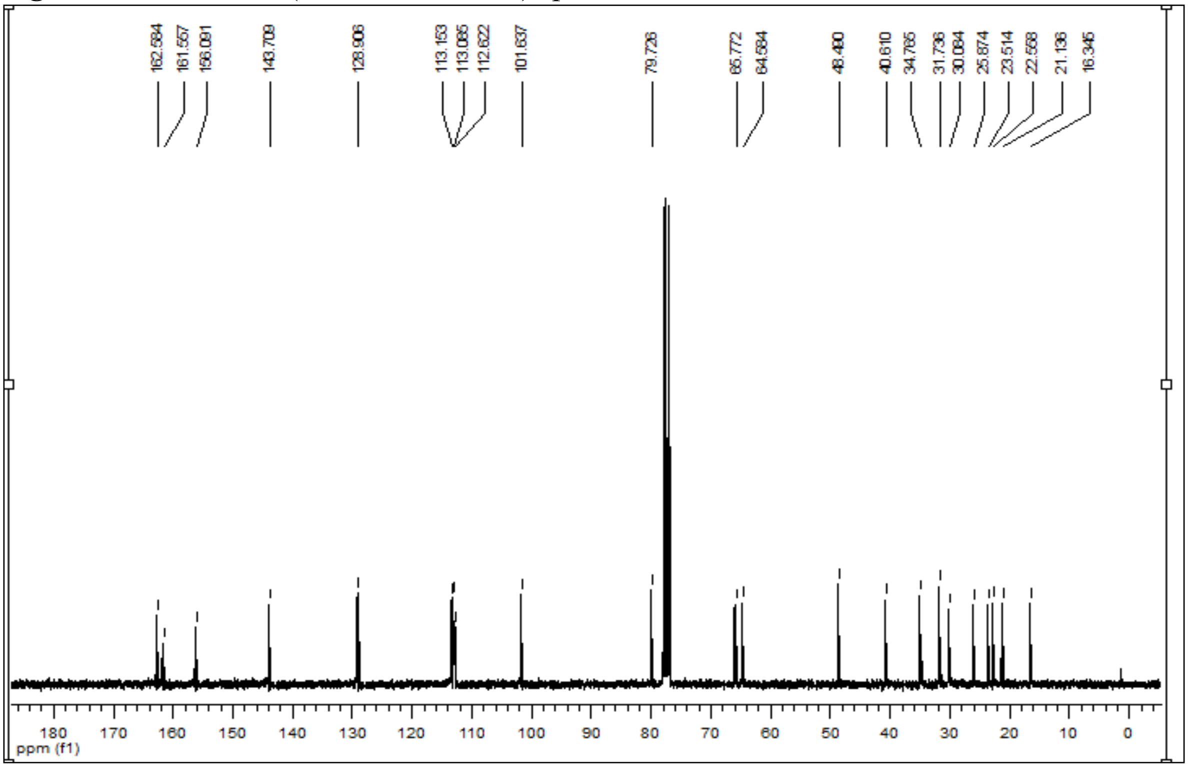

Figure S86. ^{13}C NMR (75 MHz, $CDCl_3$) spectrum of **69**

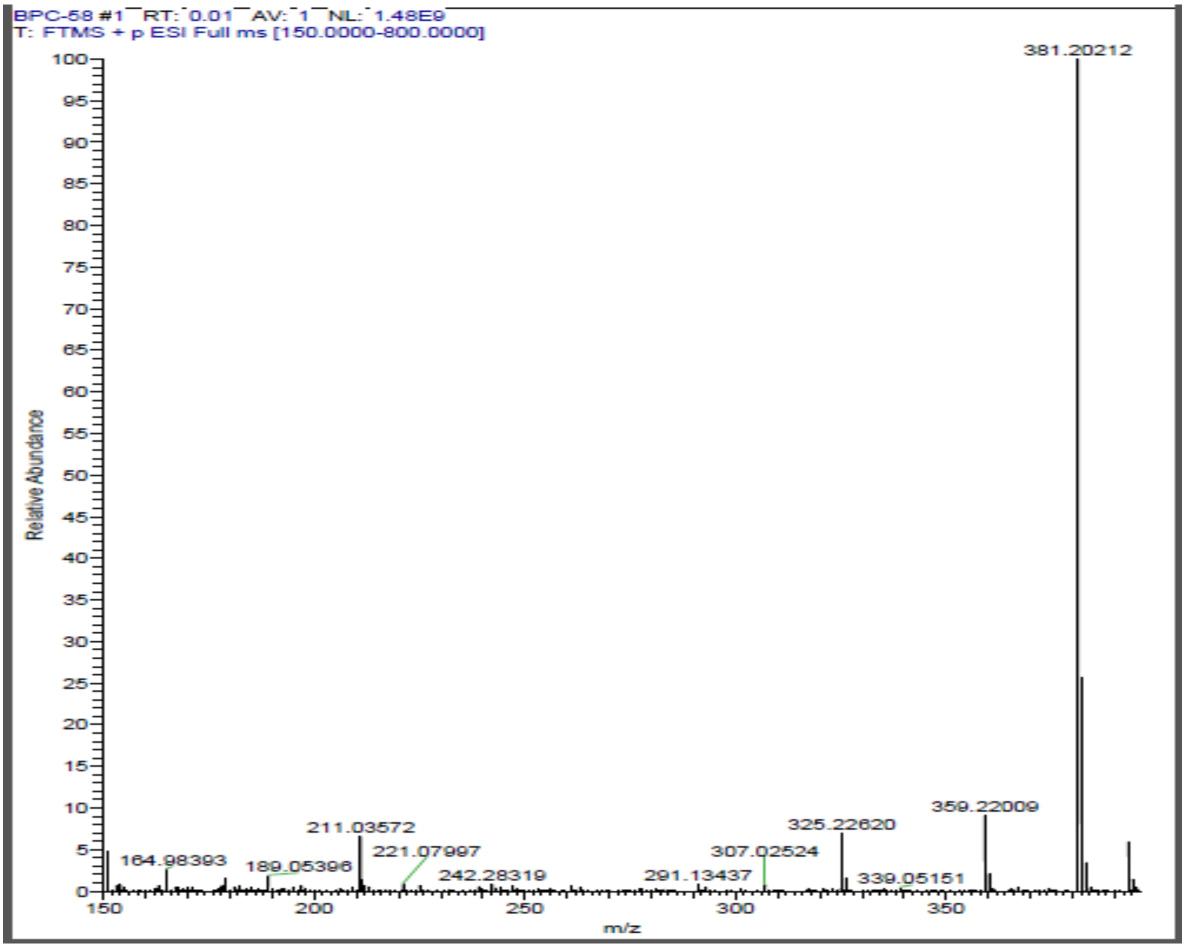

Figure S87. Mass spectrum of **69**

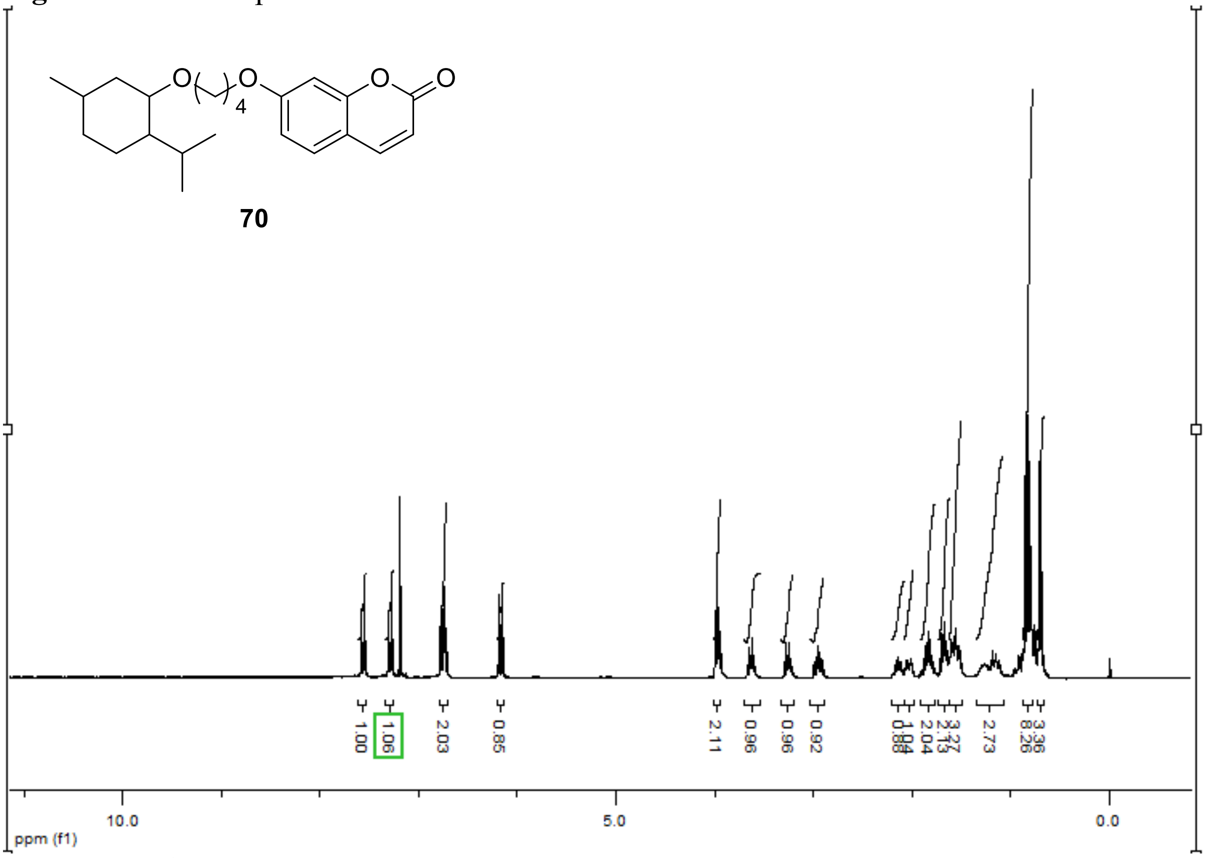

Figure S88. ^{1}H NMR (300 MHz, $CDCl_3$) spectrum of **70**

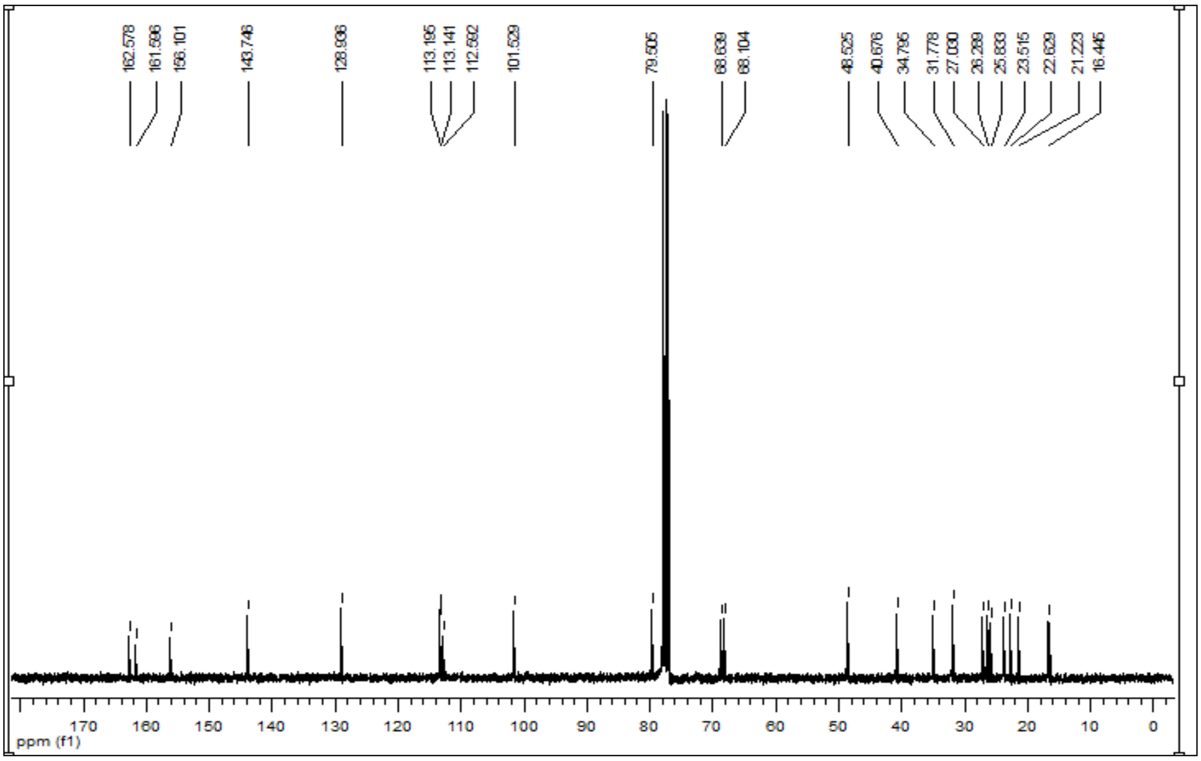

Figure S89. ^{13}C NMR (75 MHz, $CDCl_3$) spectrum of **70**

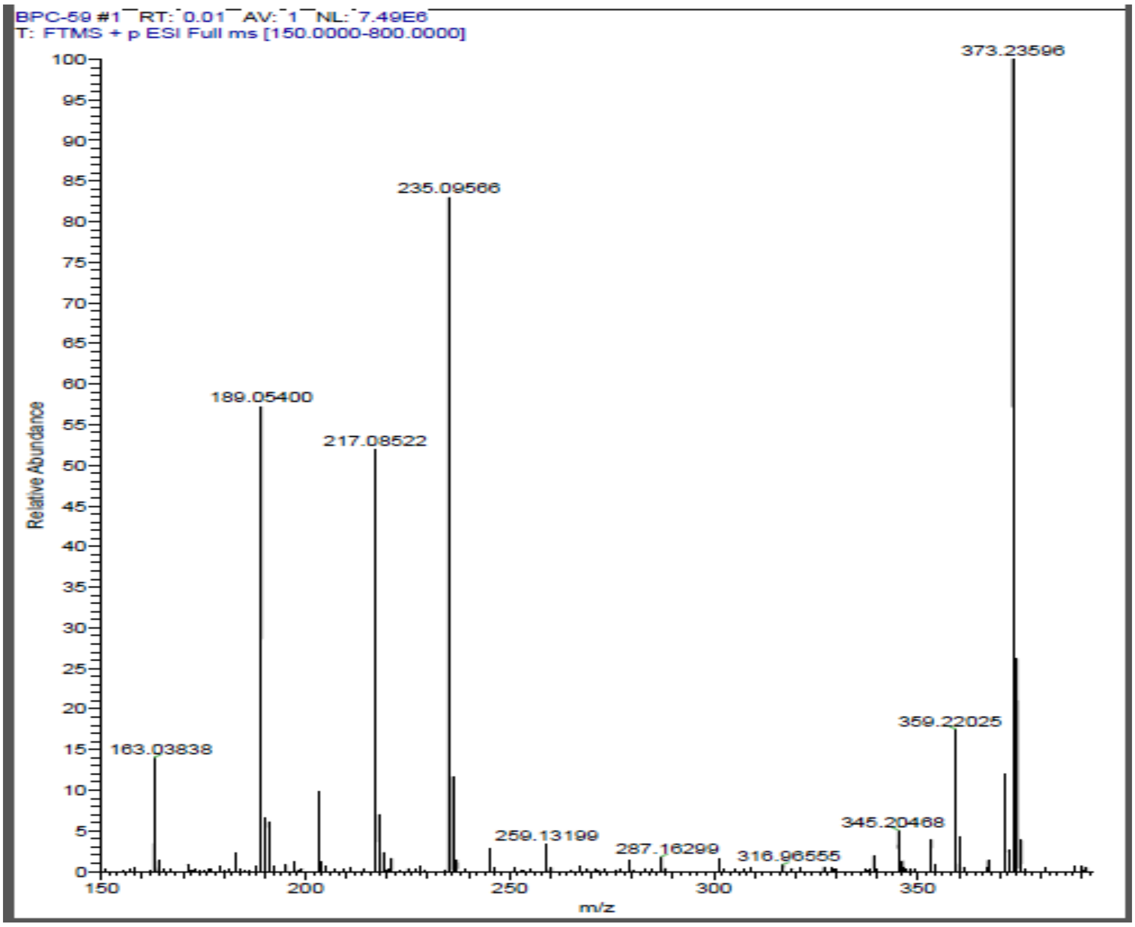

Figure S90. Mass spectrum of **70**

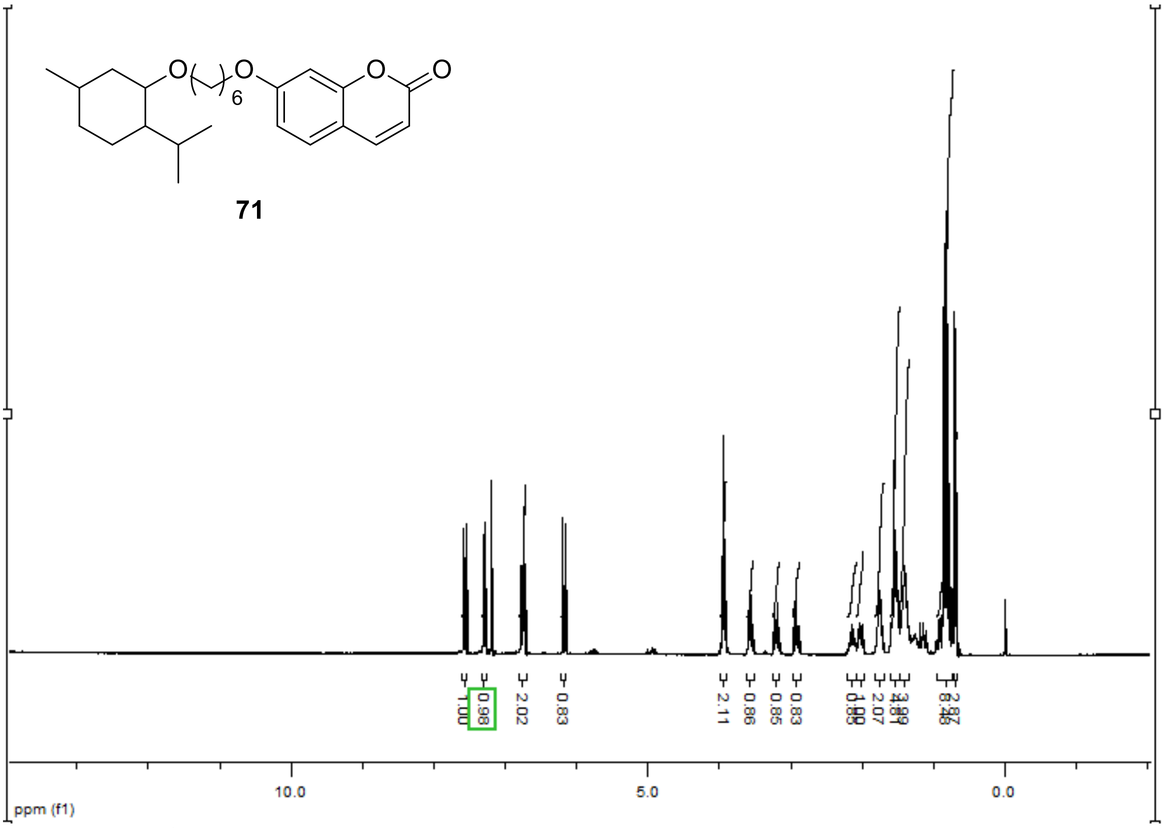

Figure S91. ^{1}H NMR (300 MHz, $CDCl_3$) spectrum of **71**

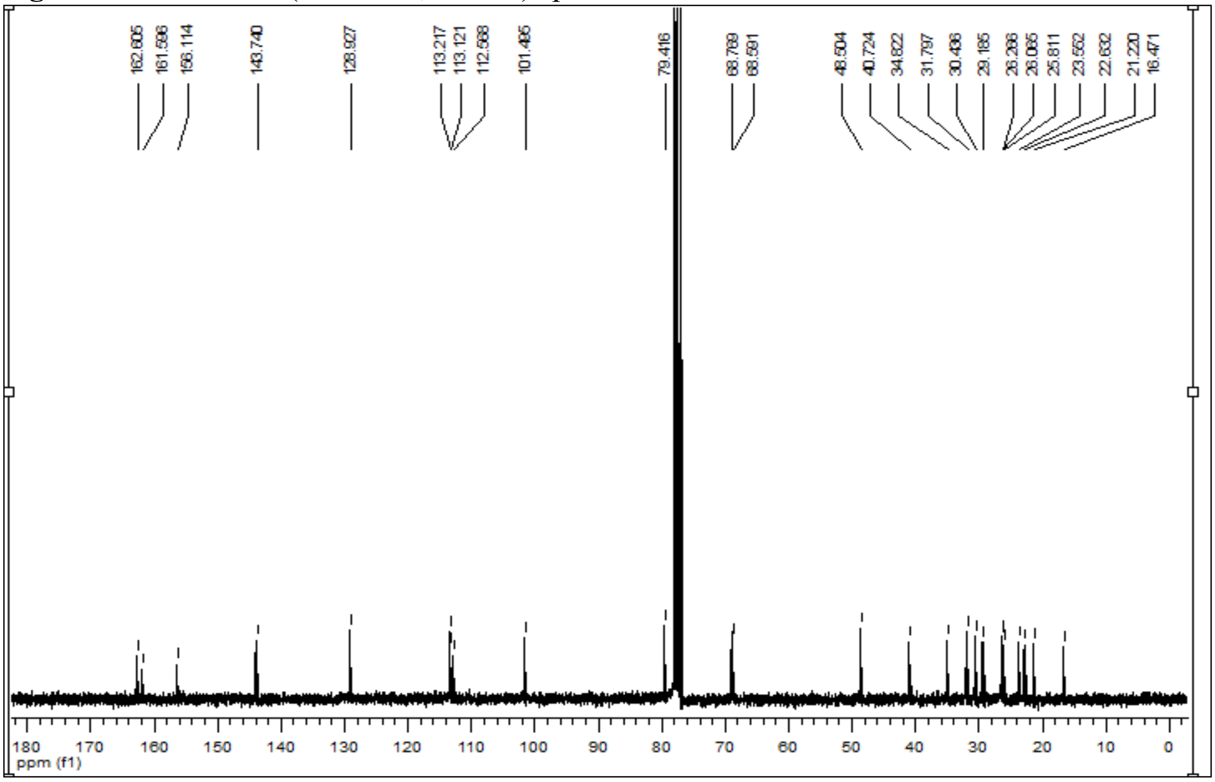

Figure S92. ^{13}C NMR (75 MHz, $CDCl_3$) spectrum of **71**

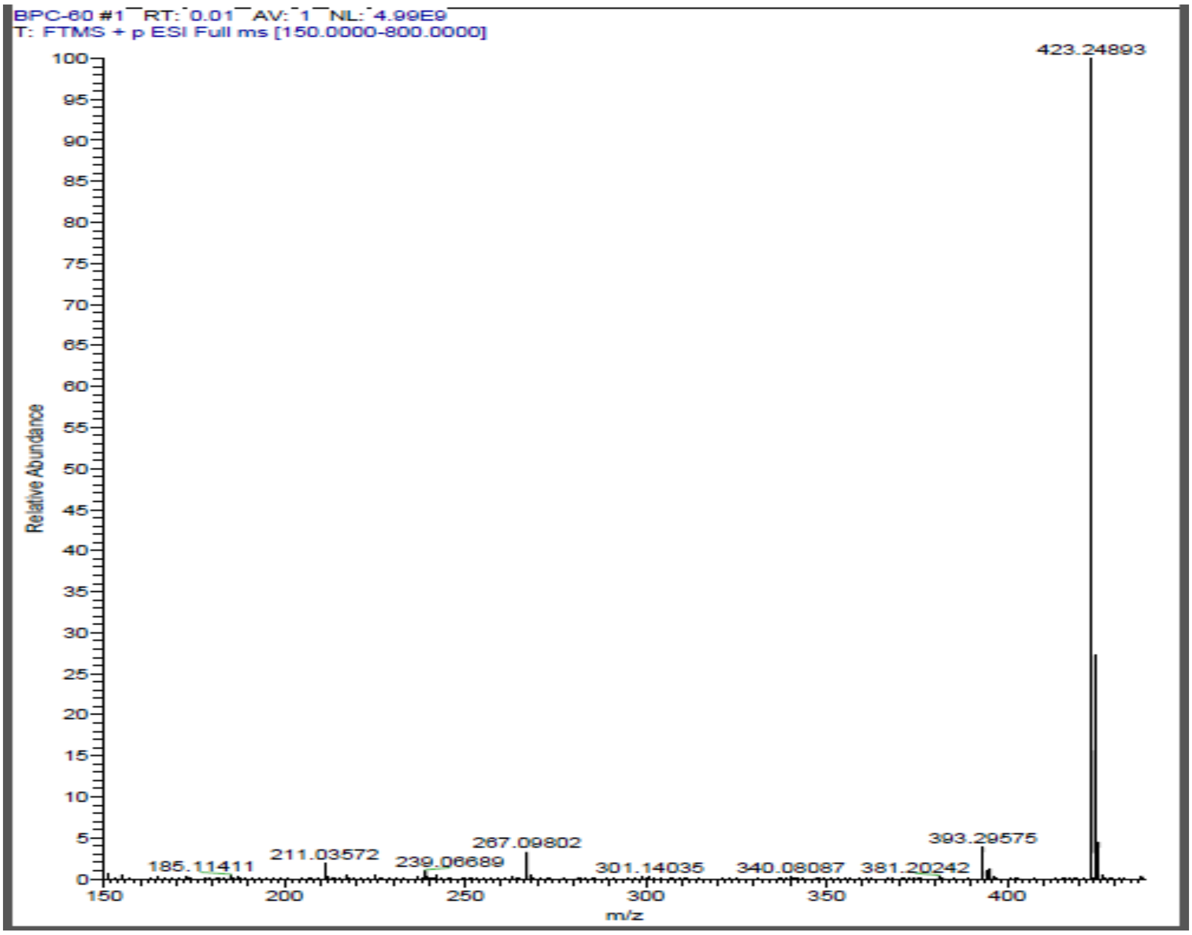

Figure S93. Mass spectrum of **71**

12

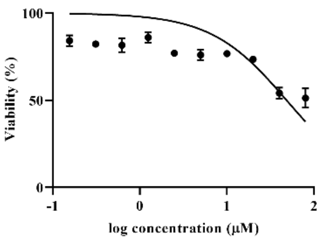

13

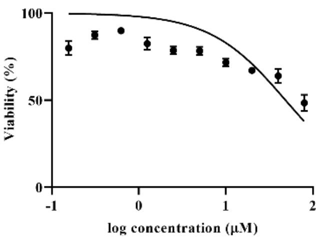

14

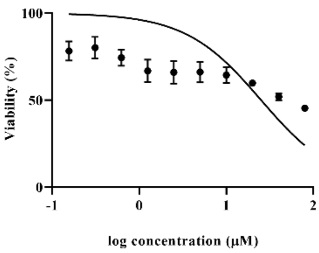

15

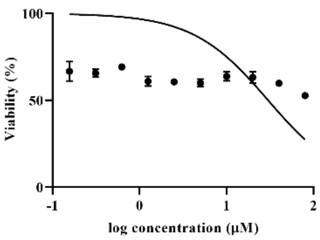

16

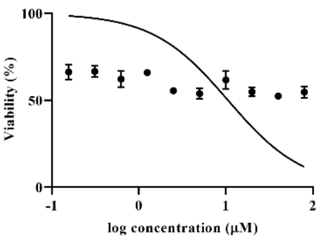

17

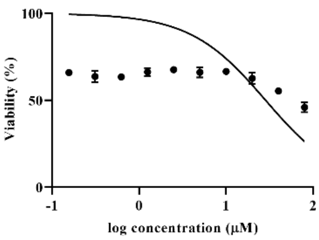

18

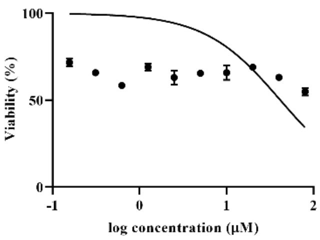

19

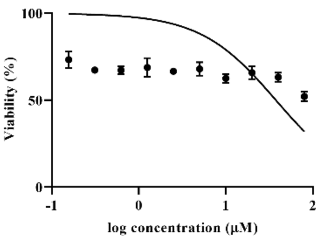

20

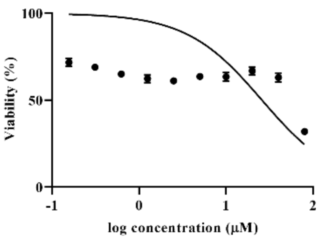

21

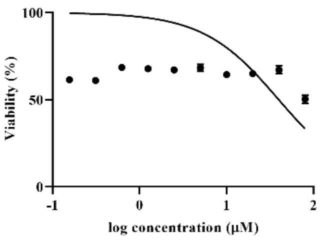

22

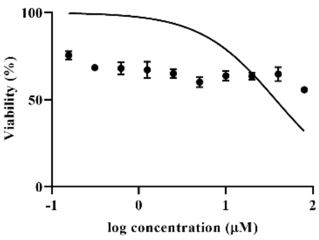

23

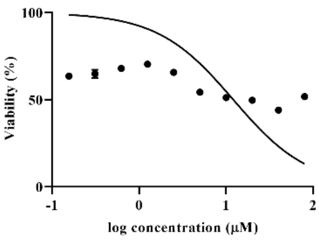

24

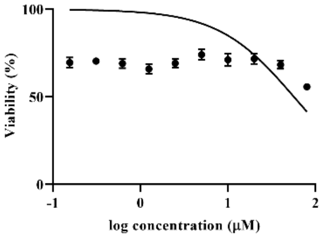

25

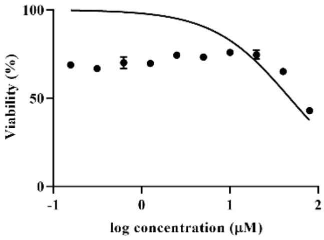

26

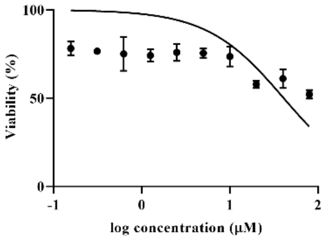

27

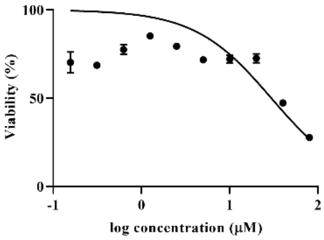

28

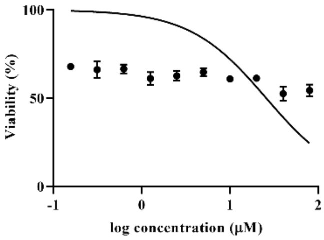

29

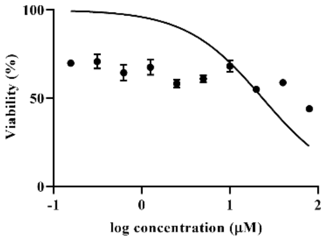

30

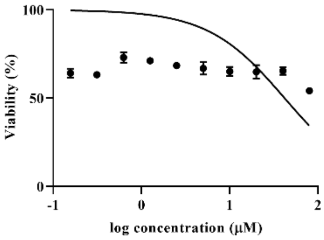

31

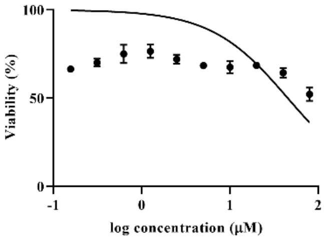

32

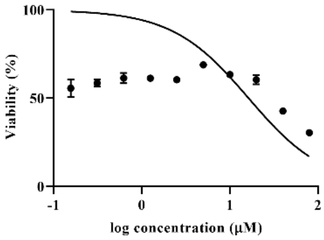

33

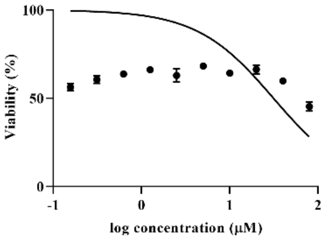

34

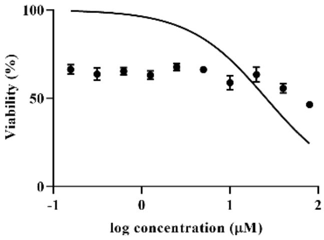

35

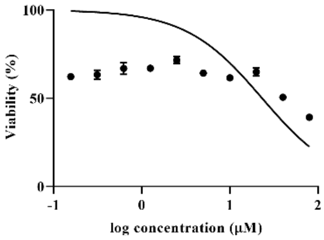

36

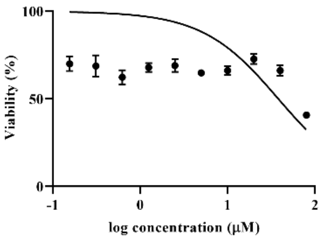

37

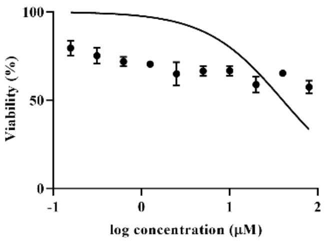

38

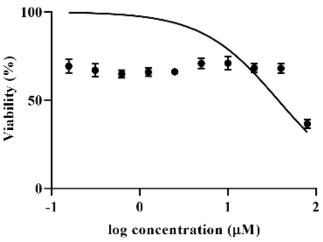

39

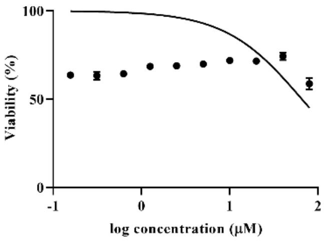

40

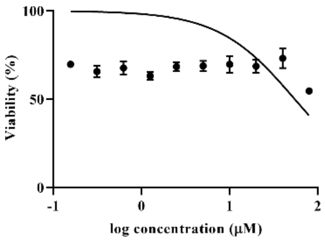

41

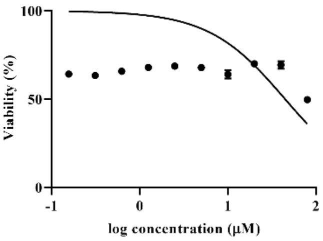

42

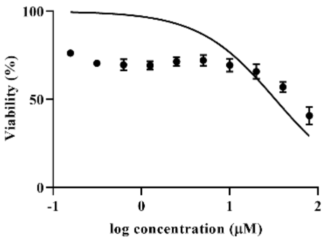

43

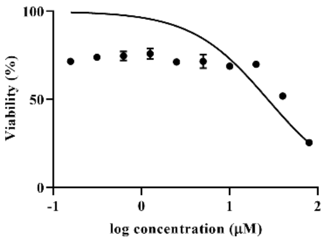

44

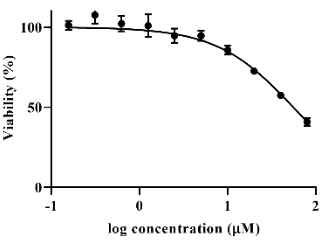

45

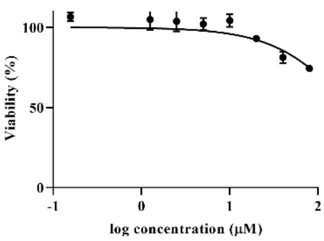

46

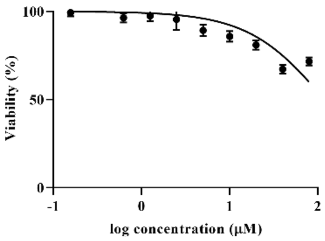

47

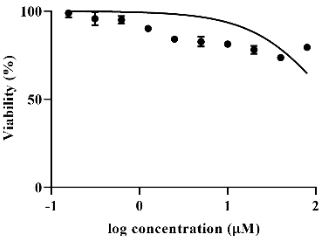

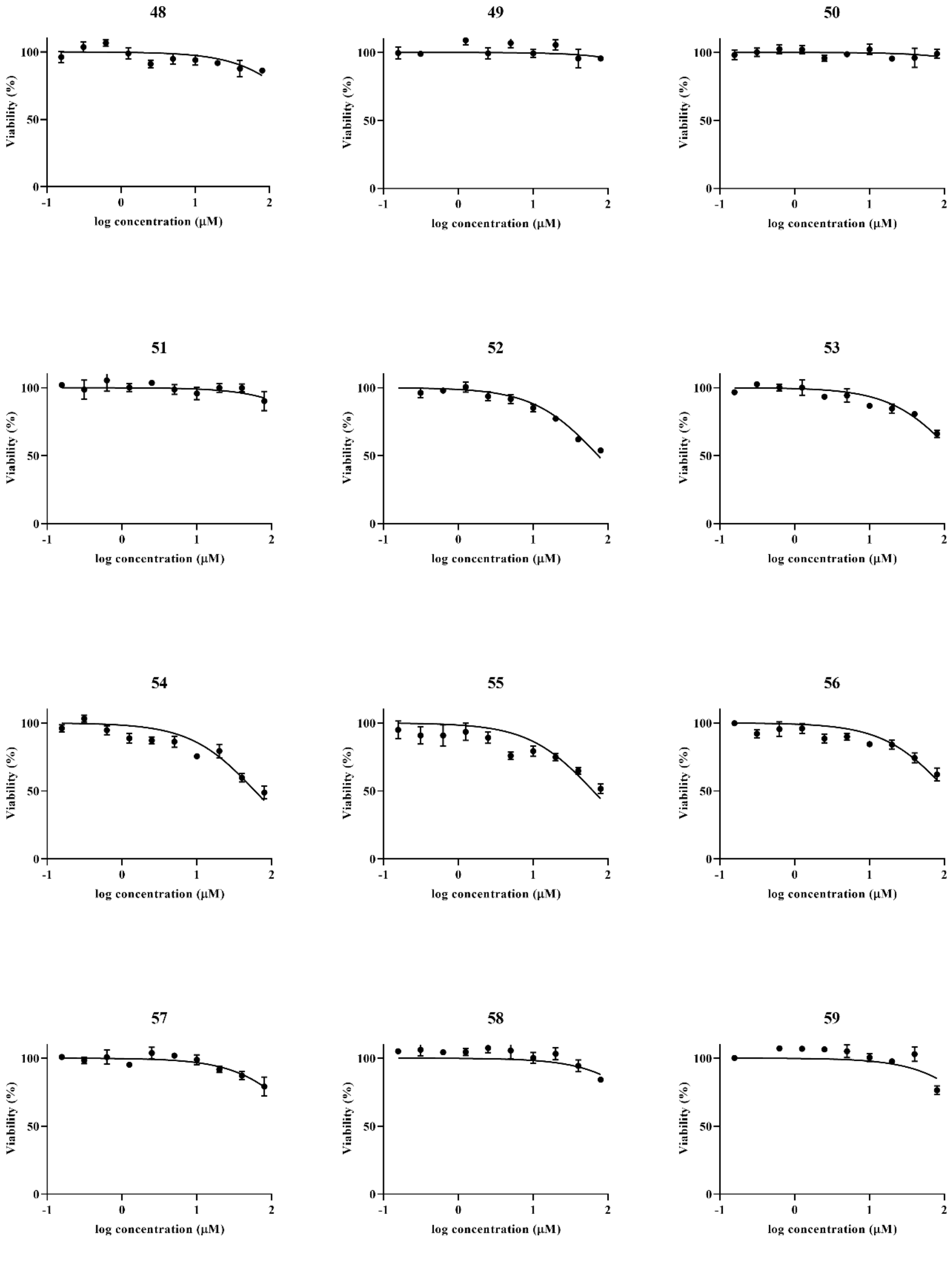

Figure S94. Viability (%) – log concentration (µM) curves showing the cytotoxic effects the compounds on HT-29 cell lines. Mean ± SEM, n=3.

12

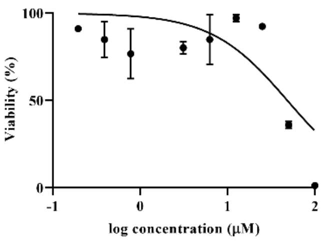

14

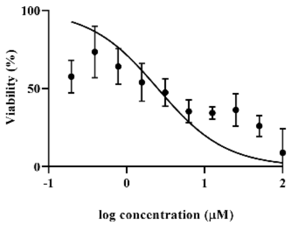

15

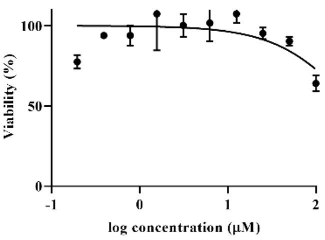

16

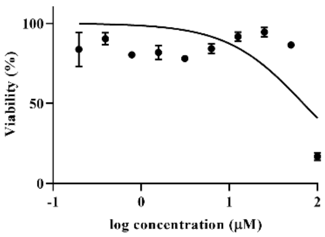

17

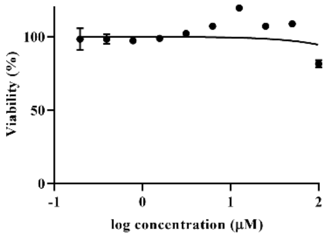

23

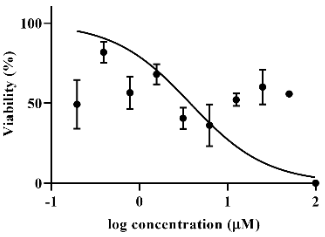

24

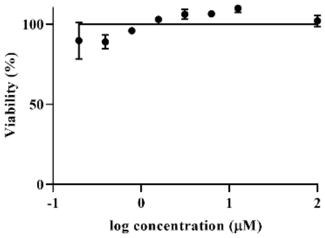

29

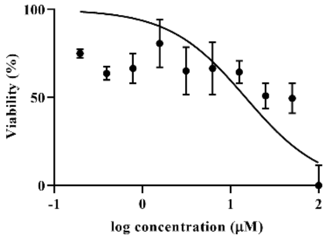

30

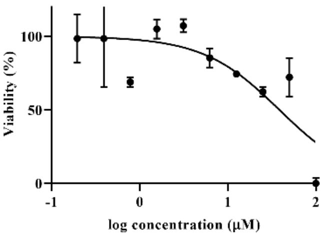

31

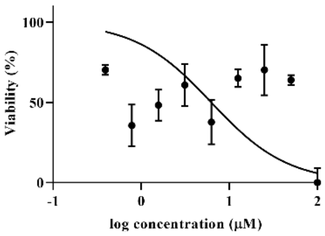

32

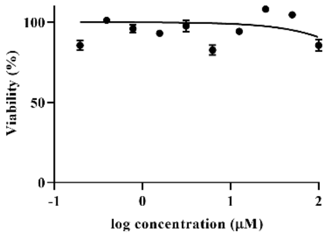

37

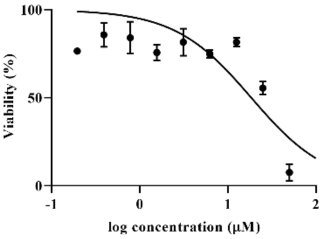

42

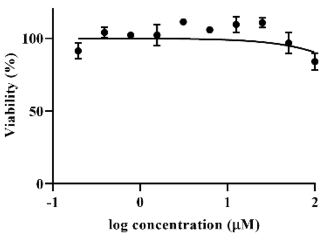

43

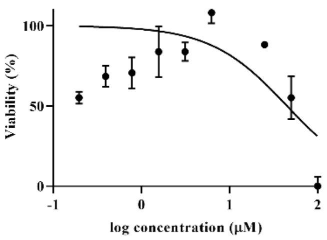

45

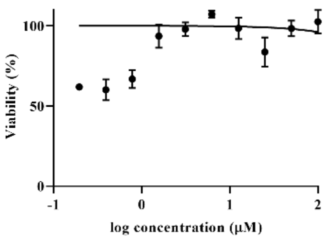

46

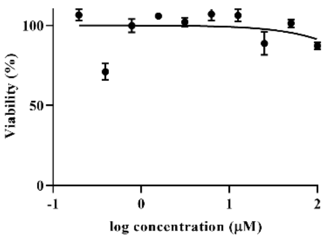

47

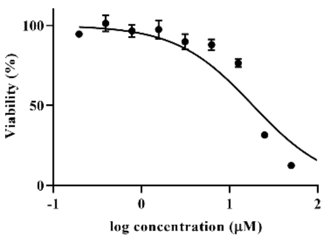

51

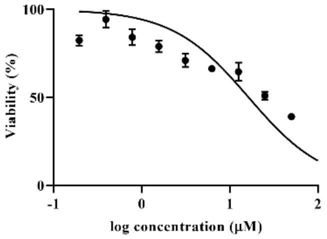

53

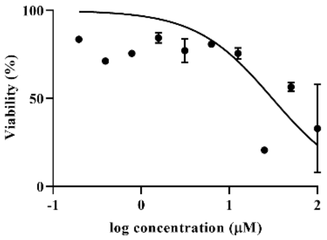

55

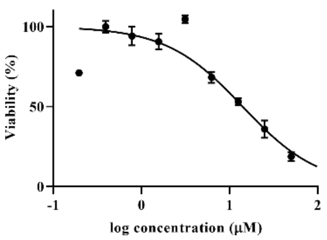

56

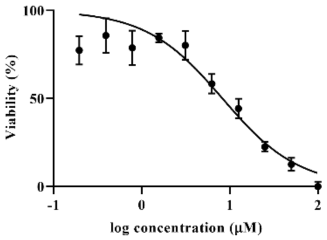

60

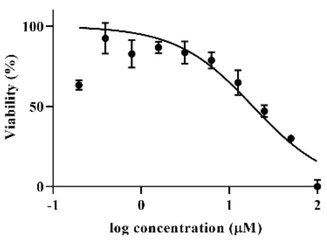

62

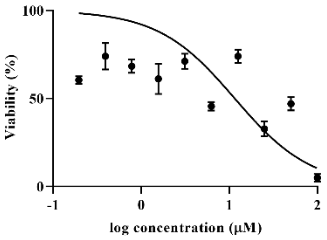

63

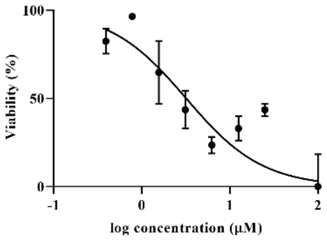

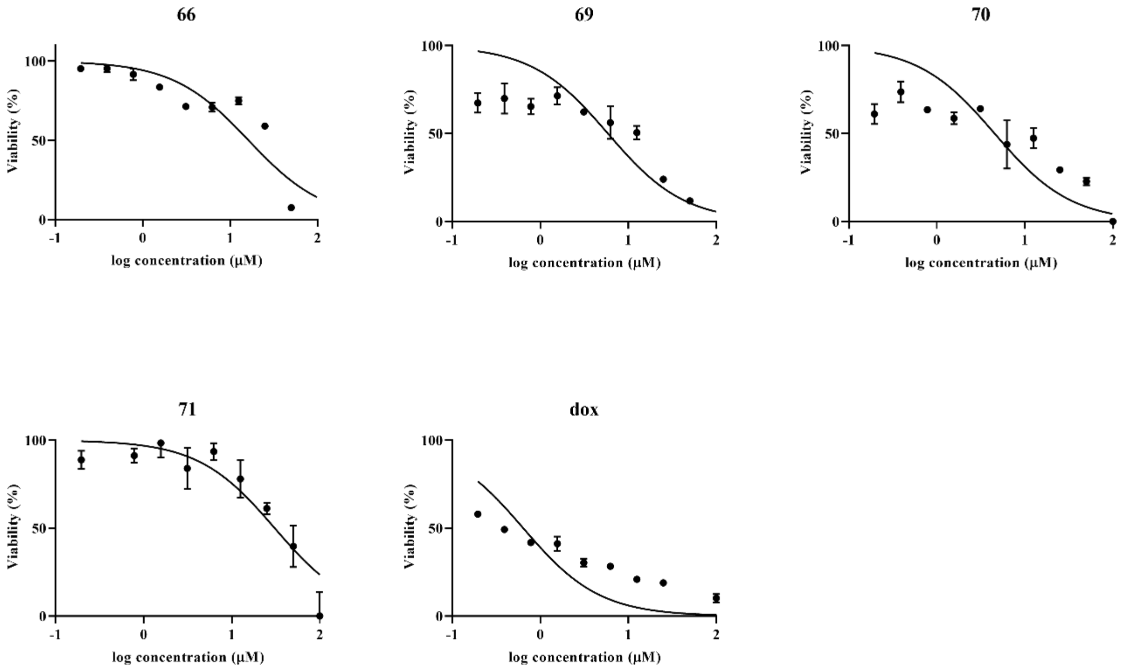

Figure S95. Viability (%) – log concentration (μM) curves showing the cytotoxic effects the compounds on MCF-7 cell lines. Mean ± SEM, n=3.

12

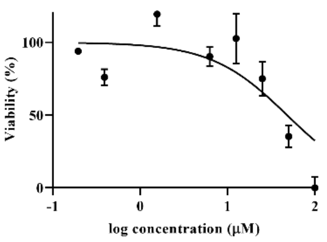

14

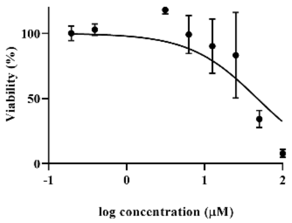

15

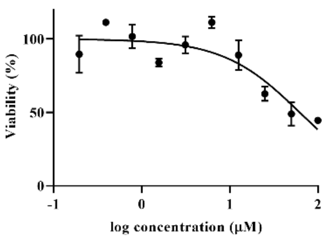

16

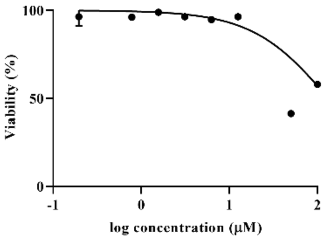

17

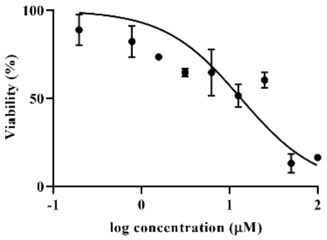

23

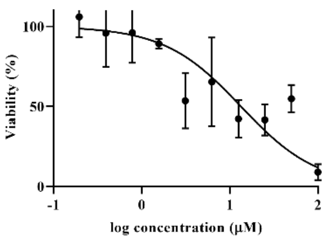

24

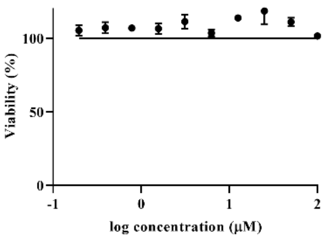

29

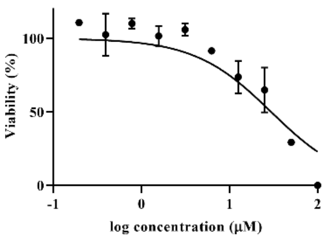

30

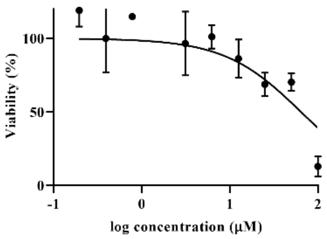

31

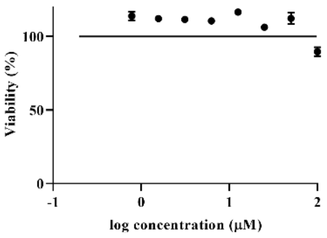

32

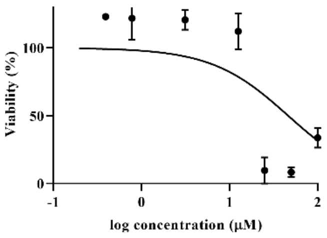

37

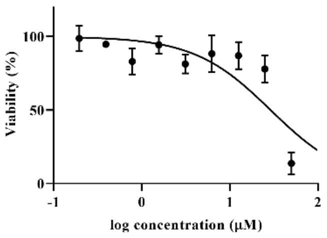

42

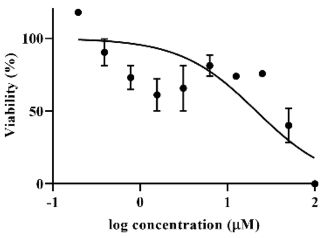

43

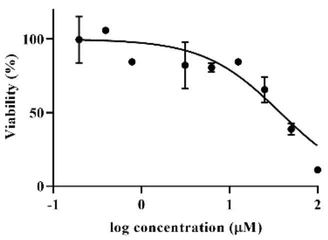

45

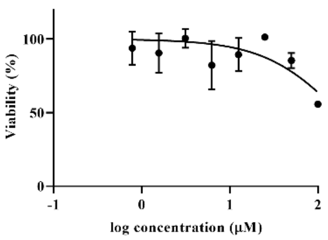

46

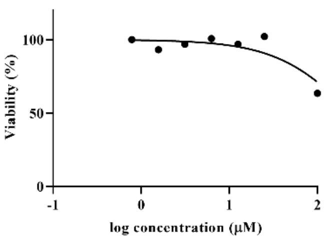

47

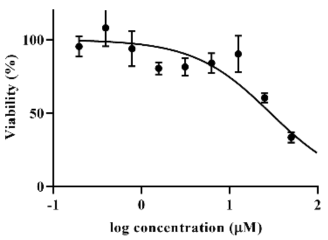

51

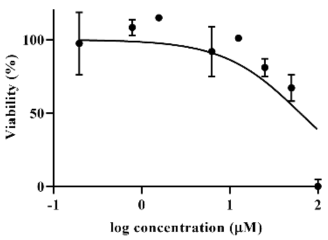

53

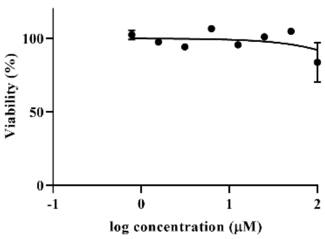

55

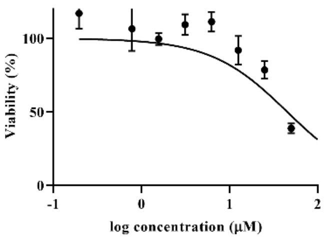

56

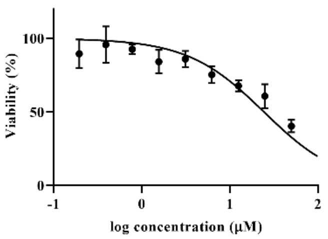

60

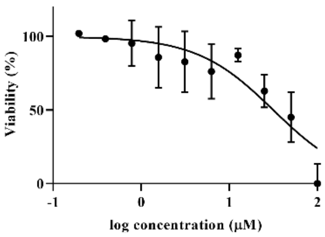

62

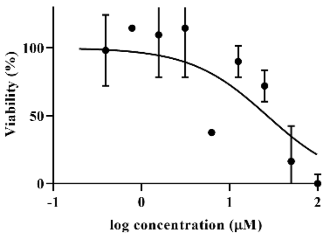

63

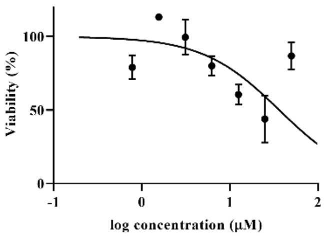

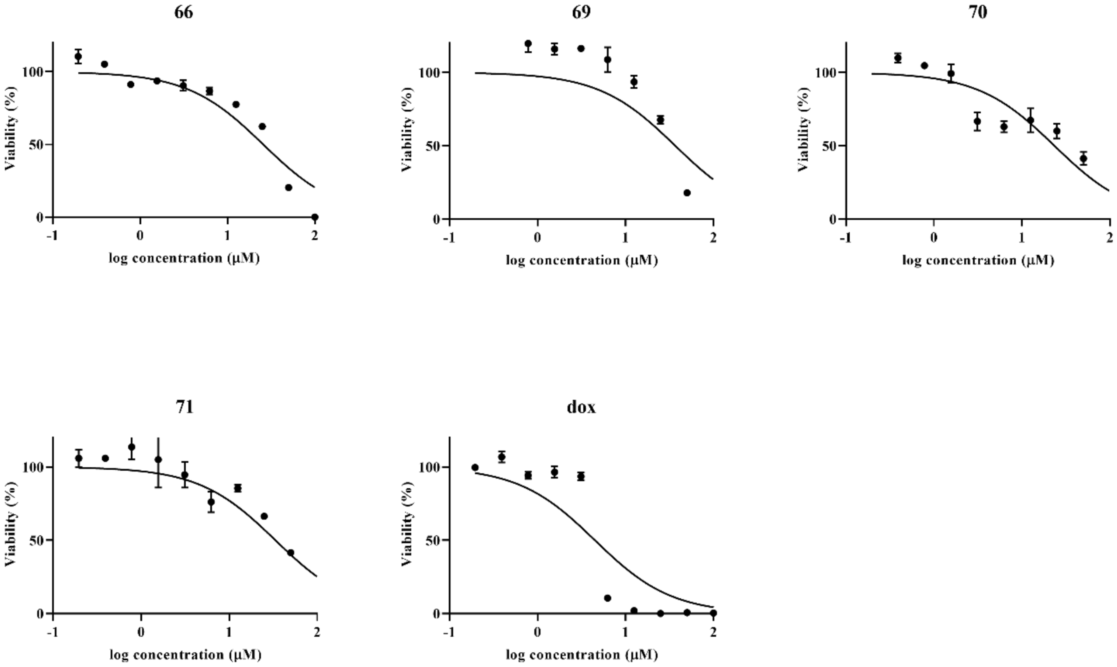

Figure S96. Viability (%) – log concentration (μM) curves showing the cytotoxic effects the compounds on PC3 cell lines. Mean ± SEM, n=3.

12

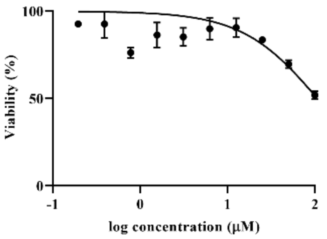

14

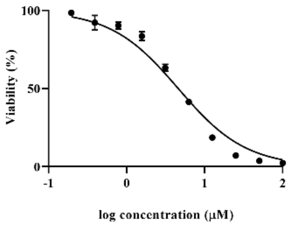

15

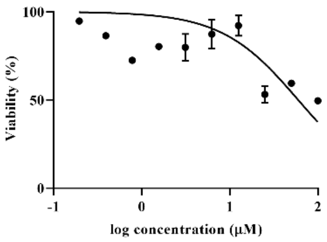

16

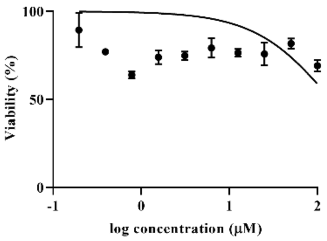

17

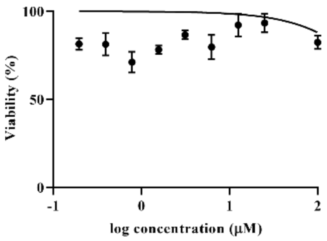

23

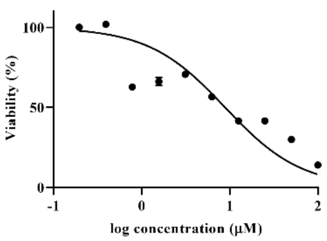

24

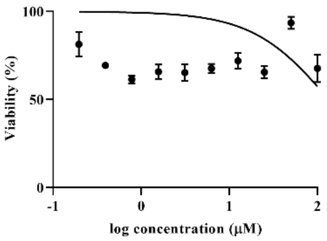

29

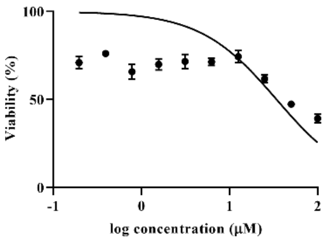

30

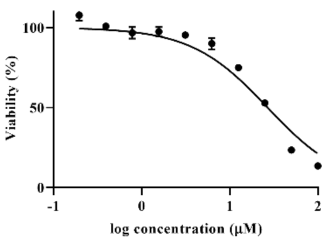

31

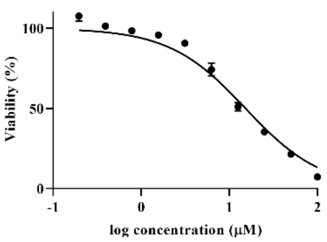

32

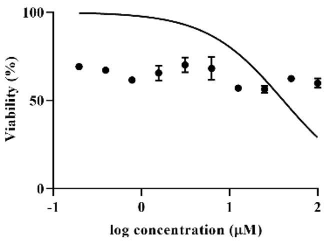

37

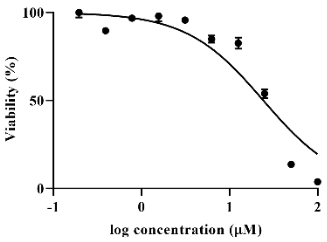

42

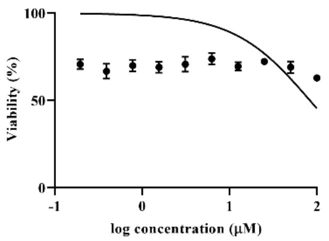

43

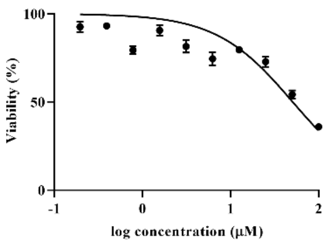

45

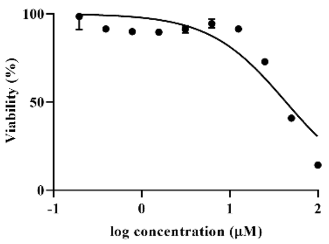

46

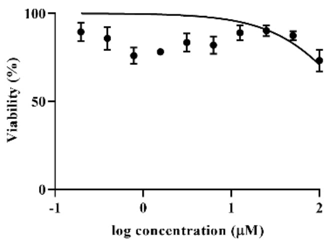

47

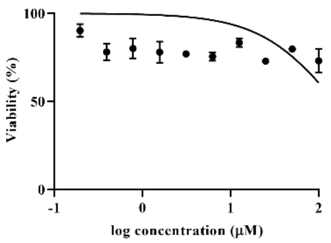

51

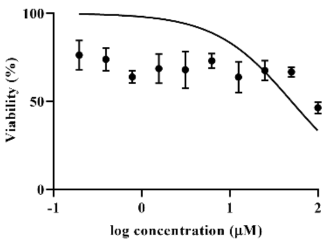

53

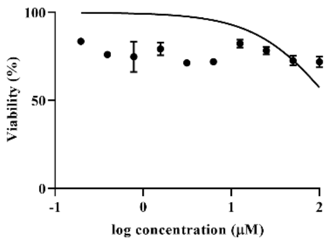

55

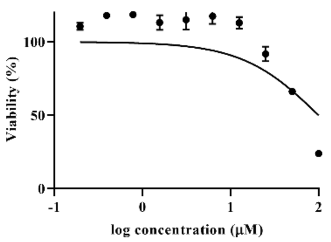

56

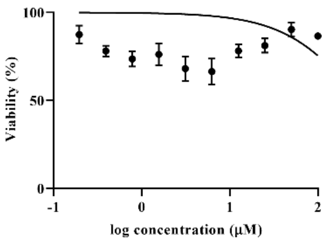

60

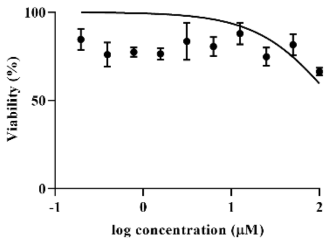

62

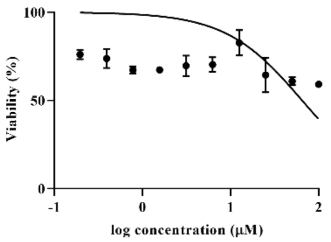

63

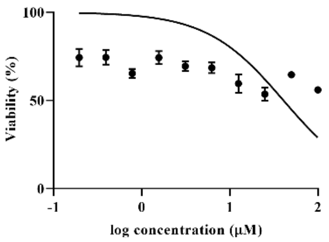

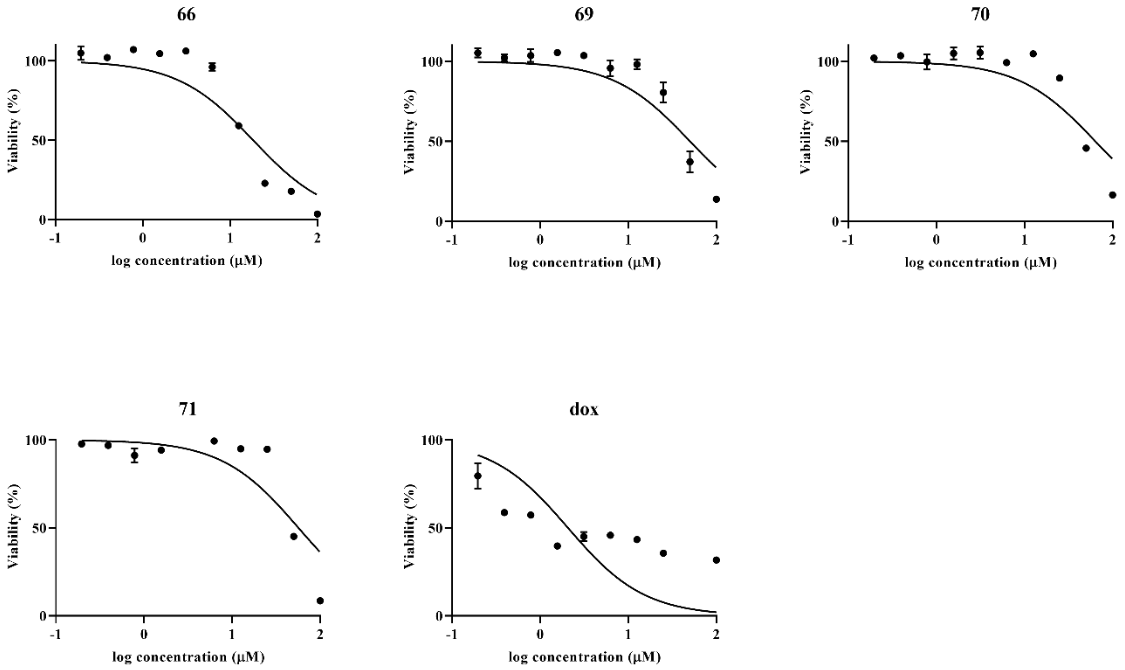

Figure S97. Viability (%) – log concentration (µM) curves showing the cytotoxic effects the compounds on Hek293T cell lines. Mean ± SEM, n=3.

The HPLC trace

Method: Shimadzu HPLC system (Kyoto, Japan) with LC-30AD pump and SPD-20A UV/VIS detector was used for the determination of the compounds. Chromatographic separation was carried out on Phenomenex C18 column (250×4.6 mm, 5 µm) at 25 °C using acetonitrile: 0.5% formic acid in water (70:30, v/v) isocratically at a flow rate of 0.8 ml/min. The chromatographic peaks were detected via a UV detector at 280 nm.

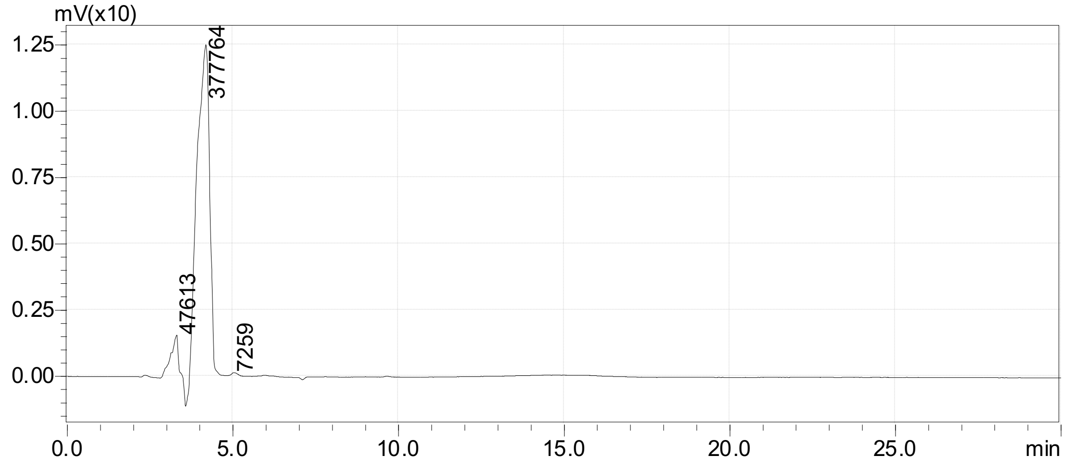

Figure S98. Methanol HPLC trace

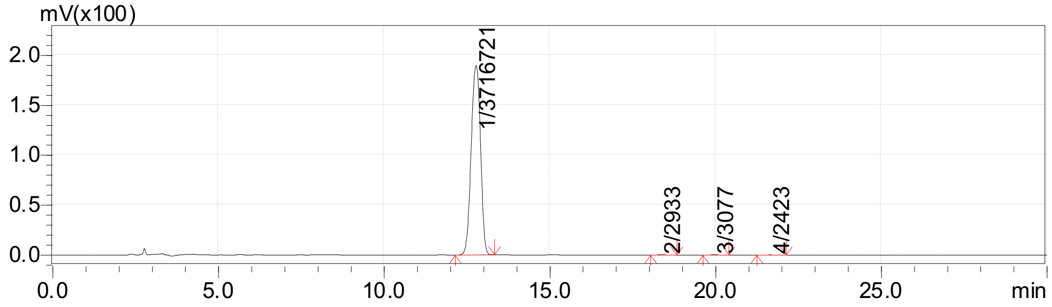

	Select	Ret. Time	Area	Height
1	☑	12.766	3716720.9	189801.6
2	☑	18.357	2932.6	128.5
3	☑	19.952	3076.7	129.3
4	☑	21.649	2423.0	103.8

Figure S99. Compound **22** HPLC trace**;** % Purity=99.77%

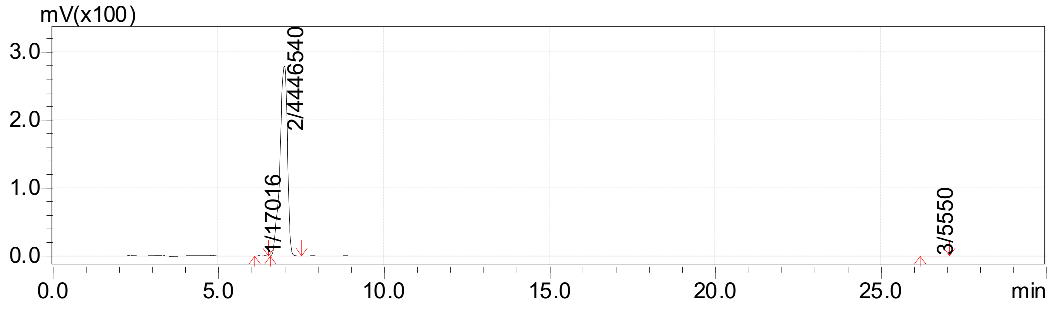

	Select	Ret. Time	Area	Height
1	☑	6.295	17016.3	1641.2
2	☑	6.979	4446540.3	278751.4
3	☑	26.593	5550.0	198.4

Figure S100. Compound **29** HPLC trace; % Purity =99.50%

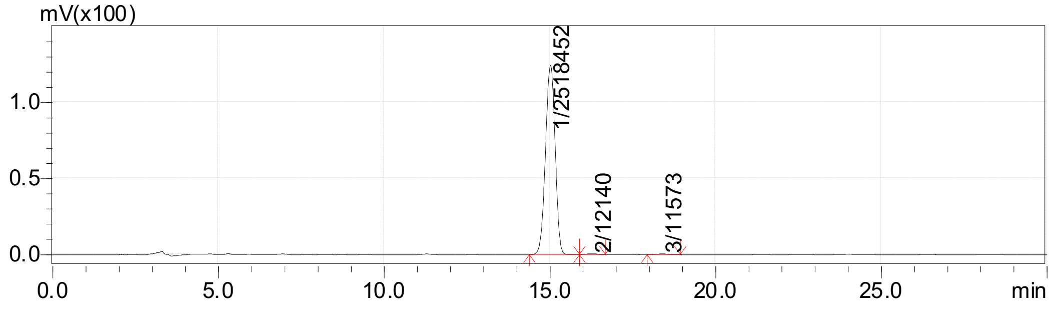

	Select	Ret. Time	Area	Height
1	☑	15.011	2518451.9	124463.3
2	☑	16.270	12139.9	556.5
3	☑	18.391	11573.2	474.5

Figure S101. Compound **37** HPLC trace; % Purity =99.06%

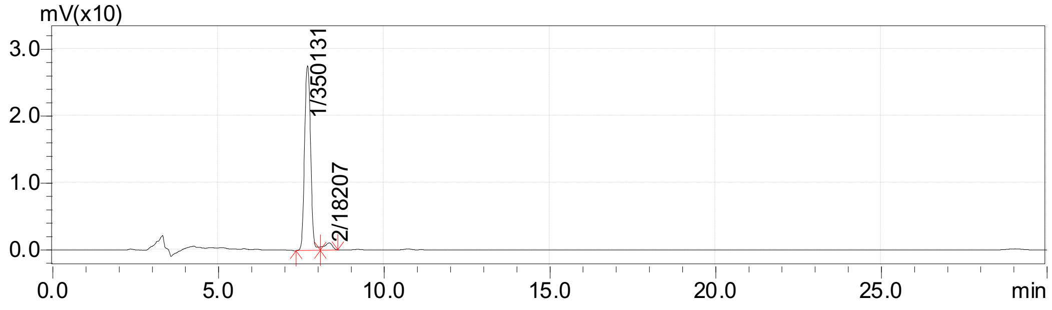

	Select	Ret. Time	Area	Height
1	☑	7.680	350130.7	27600.0
2	☑	8.333	18207.2	1057.5

Figure S102. Compound **46** HPLC trace; % Purity =95.05%

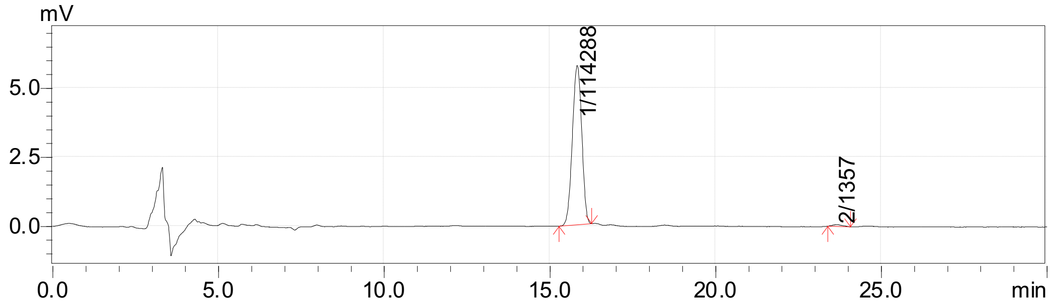

	Select	Ret. Time	Area	Height
1	☑	15.818	114288.3	5770.8
2	☑	23.621	1357.0	63.0

Figure S103. Compound **56** HPLC trace**;** % Purity =98.8%

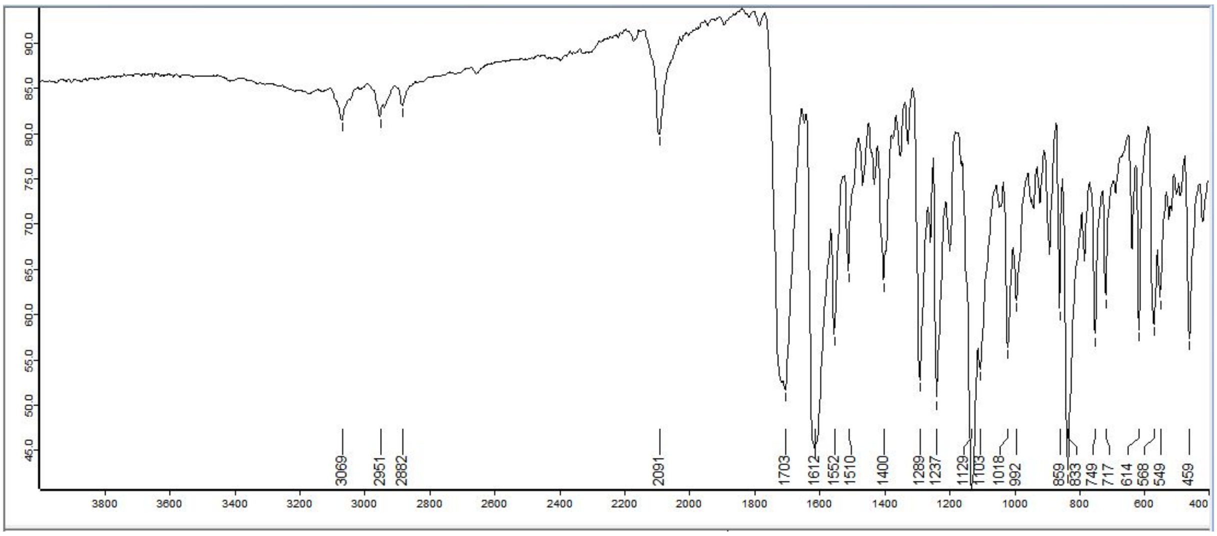

Figure S104. IR spectra for **3b**

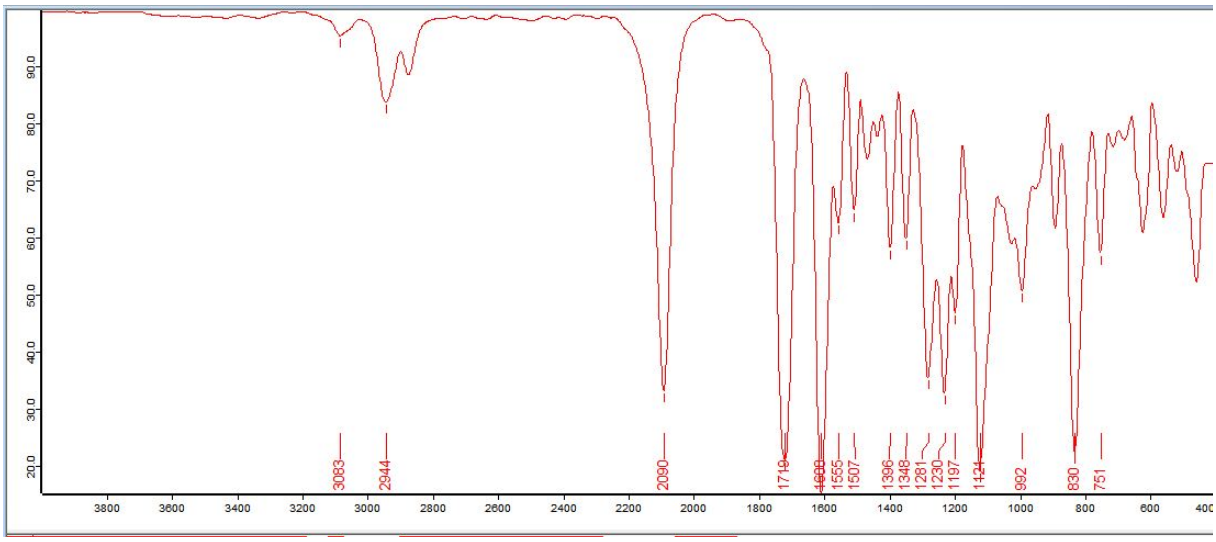

Figure S105. IR spectra for **3c**

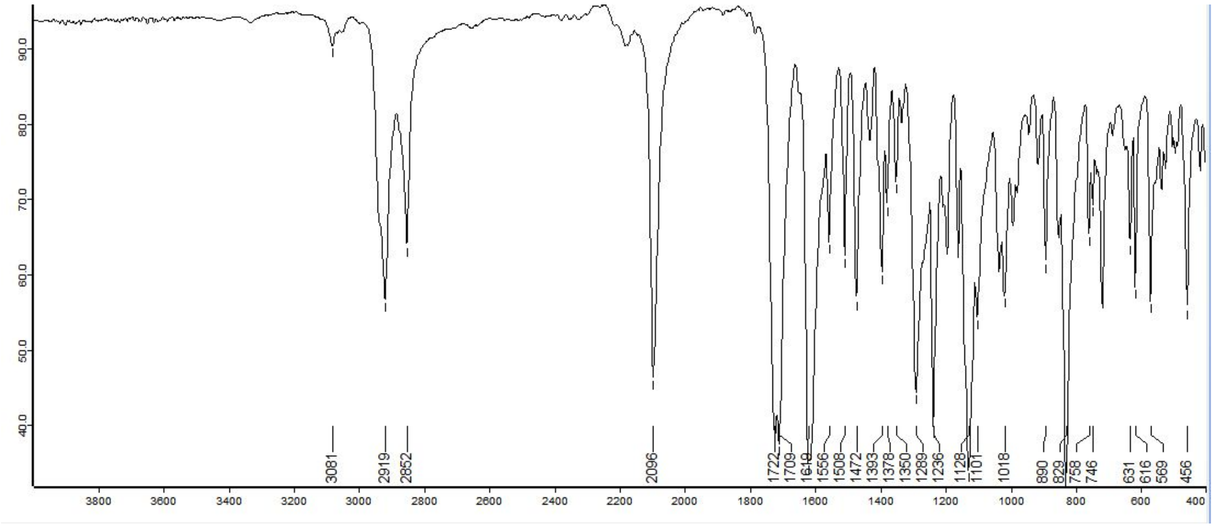

Figure S106. IR spectra for **3g**

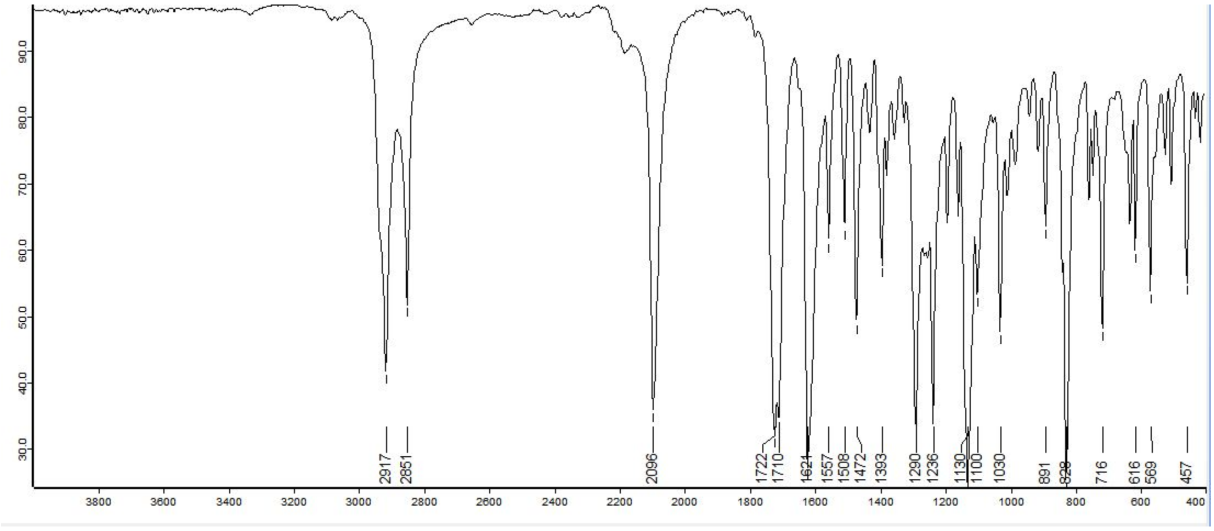

Figure S107. IR spectra for **3h**

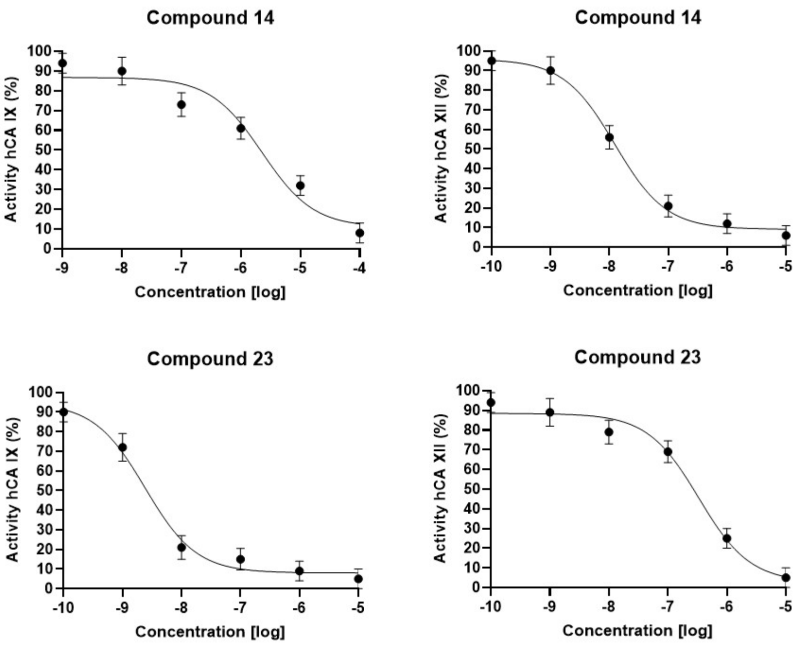

Figure S108. Graphs for CA inhibition of lead molecules; **14** and **23**

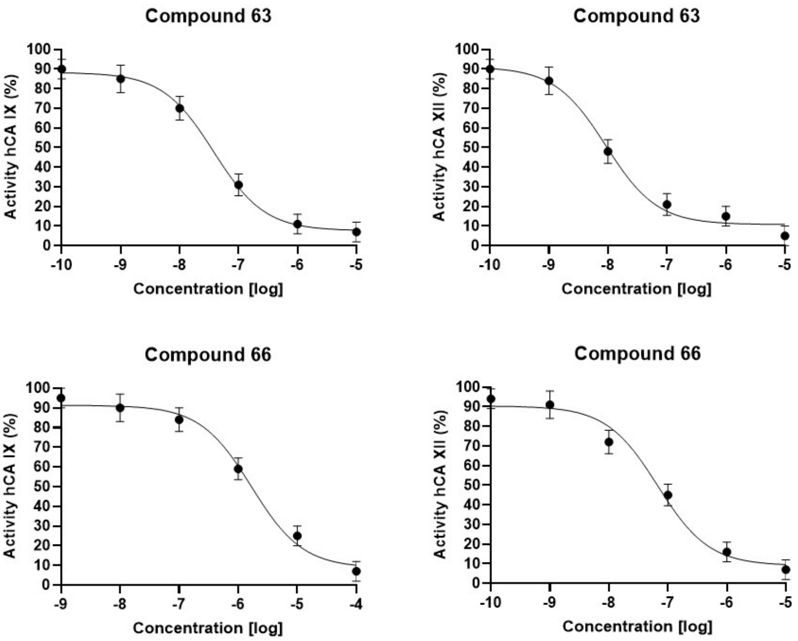

Figure S109. Graphs for CA inhibition of lead molecules; **63** and **66**
